# Design, Synthesis and Biological Activity Testing of Library of Sphk1 Inhibitors

**DOI:** 10.3390/molecules27062020

**Published:** 2022-03-21

**Authors:** Shuangshuang Geng, Haijiao Chen, Yan Li, Ying Li, Jingxiang Pang, Feipeng Zhang, Zhiqiang Qu, Mengjun Li, Na Liu, Qingqiang Yao, Yanling Mu, Bo Liu

**Affiliations:** 1 Institute of Materia Medica, Shandong First Medical University & Shandong Academy of Medical Sciences, Jinan 250117, China; ashandongshuang@163.com (S.G.); chj_jer@163.com (H.C.); liyan091022@126.com (Y.L.); liying@sdfmu.edu.cn (Y.L.); blakez1000@163.com (F.Z.); iverson13675362703@163.com (Z.Q.); 15234697337@163.com (M.L.); ln17ccc@163.com (N.L.); yao_imm@163.com (Q.Y.); 2 Biomedical Sciences College & Shandong Medicinal Biotechnology Centre, Shandong First Medical University & Shandong Academy of Medical Sciences, Jinan 250117, China; jxpang@sdfmu.edu.cn

**Keywords:** SphK1 inhibitor, melanoma, PI3K/NF-κB signaling pathway, xenograft tumor

## Abstract

Our team discovered a moderate SphK1 inhibitor, **SAMS10** (IC_50_ = 9.8 μM), which was screened by computer-assisted screening. In this study, we developed a series of novel diaryl derivatives with improved antiproliferative activities by modifying the structure of the lead compound **SAMS10**. A total of 50 new compounds were synthesized. Among these compounds, the most potent compound, named **CHJ04022Rb,** has significant anticancer activity in melanoma A375 cell line (IC_50_ = 2.95 μM). Further underlying mechanism studies indicated that **CHJ04022R** exhibited inhibition effect against PI3K/NF-κB signaling pathways, inhibited the migration of A375 cells, promoted apoptosis and exerted antiproliferative effect by inducing G2/M phase arrest in A375 cells. Furthermore, acute toxicity experiment indicated **CHJ04022R** exhibited good safety in vivo. Additionally, it showed a dose-dependent inhibitory effect on the growth of xenograft tumor in nude mice. Therefore, **CHJ04022R** may be a potential candidate for the treatment of melanoma.

## 1. Introduction

Sphingomyelin is an important component of eukaryotic cell membranes. It can be metabolized by various enzymes in turn to produce ceramide (Cer), sphingosine (Sp), and sphingosine-1-phosphate (S1P). As biological effector molecules, these three metabolites play different roles in biology [1]. Cer and Sp have been associated with growth arrest and apoptosis. In contrast, S1P has been demonstrated to play pro-survival roles. The dynamic balance between them is called a sphingolipid rheostat. Sphingolipid rheostat is complex, and many enzymes have been shown to be involved in its regulation, including ceramidase, ceramide synthase, sphingosine kinases (SphKs) and phosphorylase [2,3]. SphKs convert sphingosine into S1P, which are critical regulators of cell fate. Intracellularly generated S1P acts as a proproliferative effect in two ways: (1) directly binding to intracytoplasmic TRAF2 and activating the transcription factor NF-κB; (2) secreted into the cell in the form of autocrine or paracrine, binding to the corresponding S1P receptors (S1PRs) on the cell membrane G protein-coupled receptors, activating the pro-viability PI3K/Akt, proliferation-promoting Ras/ERK pathways, and Rac, PLC and other related pathway signals associated with cell migration movement, thereby regulating cell survival, proliferation and migration [4]. According to previous reports, there are two distinct isoforms of SphK enzymes with different functions, SphK1 and SphK2 [5,6]. As for SphK1, it has been proved to be highly expressed in various cancer cells, such as ovarian [7], cervical, colon, stomach [8], lung and brain cancers [9]. It is generally believed that the main biological function of SphK1 is to promote cell survival and proliferation, while SphK2 is mainly related to cell apoptosis [10]. Although this view has been controversial, there is no doubt that inhibition of SphK1 activity plays an important role in the treatment of cancer, inflammation and other diseases [11]. In previous studies, we found that SphK1 inhibitors have anti-inflammatory and anticancer activities, which makes the discovery of novel drugs more promising [12,13,14].

Melanoma, also known as malignant melanoma, is a malignant tumor caused by abnormally rapid proliferation of melanocytes [15]. It is more common in skin, pia meninga, choroid and other parts of the eye [16]. Melanoma accounts for only 1% of all skin cancers, but it is the most invasive and dangerous one, accounting for 90% of all skin cancer deaths [17]. Melanoma is a disease of old age in which a random accumulation of acquired mutations in melanocytes leads to the transformation of melanocytes into melanoma. Melanoma is more common in people aged 16–60 years, with an average survival time of 30.3 months. At present, the main treatment for melanoma is surgery, but there are poor prognosis, high invasion and metastasis [18]. With further research, it has been found that melanoma is caused by complex changes in multiple signaling pathways that control cell proliferation and escape the process of cell death. Damage or over activation of certain components of these pathways may lead to malignant transformation and the development of cancer. In the present study, we consider that signaling pathways such as cyclin/CDK, Ras/Raf/MEK/MAPK [19], JNK/C-Jun/AP-1, PI3K/Akt/PTEN/mTOR [20], IKK/I-κB/NF-κB [21], Wnt/β-catenin [22], Notch, Jak/STAT [15], MITF and some growth factors regulate the cycle progression, apoptosis and development of melanoma cells. However, no ideal inhibitor has appeared thus far, and there are still no inhibitors approved for marketing. It is highly necessary to find new selective inhibitors against melanoma with strong affinity, metabolic stability, high selectivity and low toxicity.

It has been reported that elevated sphingosine-1-phosphate (S-1-P) levels resulting from increased activity of sphingosine kinase-1 (SPHK1) occur in melanomas. Our research team has long been committed to the research and development of SphK1 inhibitors. In this study, starting from the crystal structure of SphK1, computer-aided drug design was used to screen out substituted diaryl compound with novel structure and ideal SphK1 inhibitory activity (named **SAMS10**), which has been synthesized by our group as a promising lead compound for the development of antitumor drugs. In total, 50 derivatives were designed and synthesized with **SAMS10** as the lead compound against A549, SKOV3, A375 and LOVO cancer cells evaluated by the MTT method, followed by evaluating their antitumor activities in vitro and in vivo.

## 2. Results and Discussion

### 2.1. Design and Synthesis

**Figure 1:**
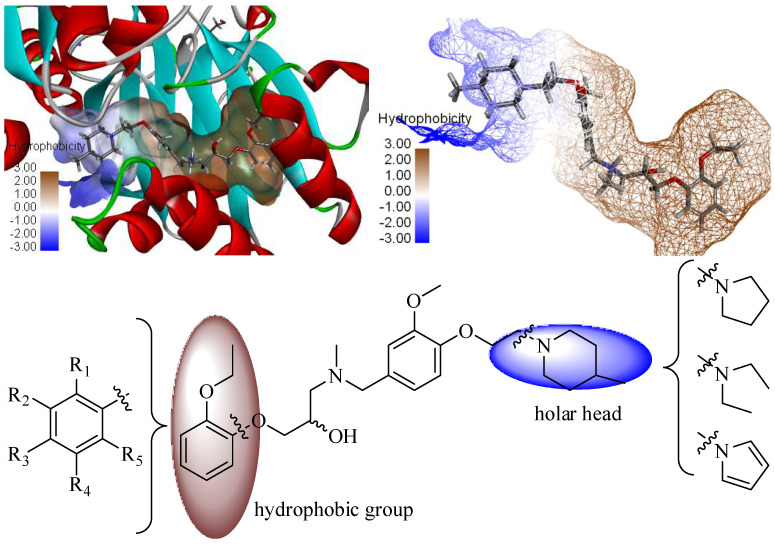
The structure of **SAMS10** and the docking of **SAMS10** (**SAMS10**, this racemate was used for molecular docking, the R configuration of **SAMS10** was used for the analysis) with a SphK1 crystal structure (**3vzb**).

**Scheme 1:**
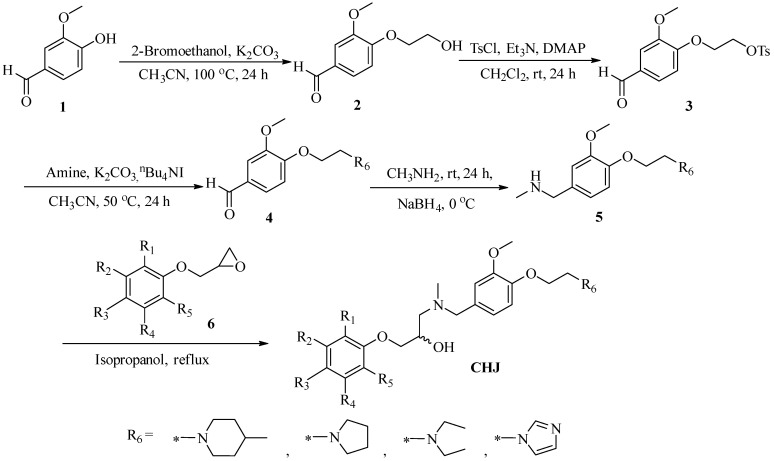
The structures and synthetic route of designed compounds.

The beginning of the subject was that our team used computer-aided drug design software (Discovery Studio 2020) to screen out a SphK1 inhibitor **SAMS10** with moderate inhibitory activity. The IC_50_ of **SASM10** against SphK1 is about 9.8 μM (manuscript on **SAMS10** has been accepted by the Journal of Chemical Research). The structure of **SAMS10** is shown in Figure 1. It can be seen from the figure that a 4-methyl-piperidine group of **SAMS10** is located at the polar head (hydrophilic end) of the protein, and the terminal aromatic ring is located at the hydrophobic end of the protein cavity. Thus, in order to obtain SphK1 inhibitors with high activity, we purposefully modified the two ends. When designing the compound, in the polar head position, the 4-methyl-piperidine group is replaced by pyrrolidine and diethylamine. At the same time, SphK1 inhibitors may be found by changing the substitution of hydrophobic aromatic rings. The general formula for the synthesis of the lead compound and their derivatives is shown in the Scheme 1. Firstly, intermediate compounds **5** were synthesized from raw material Vanillin (**1**) by some nucleophilic reactions and reductive amination reactions. Then, a series of target compounds were obtained by the ring-opening reaction between intermediate compounds **5** and phenoxy-2,3-epoxypropane compounds **6**.

### 2.2. Synthesis of Intermediate Compounds (***5***)

Scheme 1 shows the synthesis of intermediate compounds (**5**). The intermediate compound (**5**) is synthesized from vanillin (**1**) through a five-step reaction. First, compound **4** was obtained by substituting *p*-toluenesulfonyloxy of compound **3** with amines. Then, compounds **5** were obtained by reductive amination reaction of compounds **4**. The four intermediate compounds obtained are shown in the Table 1. The specific operation is described in the experimental section.

### 2.3. Synthesis of Phenoxy-2,3-Epoxypropane Compounds (***6***)

Scheme 1 shows the general formula of compound **6**. The synthesis of phenoxy-2,3-epoxypropane compounds (**6**) is derived from the nucleophilic substitution reaction between phenols with different substitutions on the benzene ring and epibromide. Supporting information provides detailed structure of 37 compound **6**.

### 2.4. Synthesis and Kinase Inhibitory Activities of the Designed Compounds (CHJ)

The designed **CHJ** series compounds are obtained by the nucleophilic substitution reaction between the intermediate compounds (**5**) and the 1-phenoxy-2,3-epoxypropanes (**6**) with different substitutions on the benzene ring. The structures of **CHJ** compounds and the preliminary results of kinase screening are shown in the Table 2. It can be seen from the data that **CHJ04022**, **CHJ04082** and **CHJ04083**, with the best kinase activity, had only about 30% inhibition on SphK1 at the concentration of 10 μM. However, their inhibitory effects on SphK1 are not as strong as **SAMS10** (**SAMS10** had about 60% inhibition on SphK1 at the concentration of 10 μM). Therefore, the change of the polar head amine group and the hydrophobic end substituent of the compound did not significantly improve the compound’s inhibitory effect on SphK1. It is worth noting that the compounds **CHJ03011** and **CHJ03012**, containing long alkyl side chains on the aromatic ring, still show signs of an inhibitory effect on SphK1, indicating that the hydrophobic part still has the possibility for structural modification.

### 2.5. 3-(4,5-Dimethylthiazol-2-yl)-2,5-Diphenytetrazolium Bromide (MTT) Assay

Most of the compounds have significant inhibitory effects on the proliferation of human lung cancer (A549), human ovarian cancer (SKOV3), human melanoma (A375) and human colon cancer (LOVO) cells. In addition, it can be seen from Table 3 that compounds with 4-methyl-piperidinyl in polar head usually have better anticancer activity than those with pyrrolidine in polar head, compounds with a longer alkyl at the hydrophobic end (**CHJ03011** and **CHJ03012**) still showed good anticancer activity, but for compounds with larger polar groups at the hydrophobic end (**CHJ04068** and **CHJ04061**), the anticancer activity is significantly reduced.

It is worth noting that the compounds with three bromine atoms on the aromatic ring (**CHJ04022** and **CHJ04068**) have the best anticancer activity, and their anticancer activity is even better than that of cisplatin. Two isomers, **CHJ04022R** and **CHJ04022S**, were obtained by chemical synthesis. Supporting information provides their synthetic methods. Given the interesting results, we next investigated the effect of **CHJ04022R** and **CHJ04022S** on human melanoma (A375). The results are shown in Table 4. **CHJ04022R** exhibited obvious inhibitory activities against A375. The most potent **CHJ04022R** was then used to explore the further biological mechanism.

### 2.6. CHJ04022R Blocked the Migration of A375 Cells

**Figure 2:**
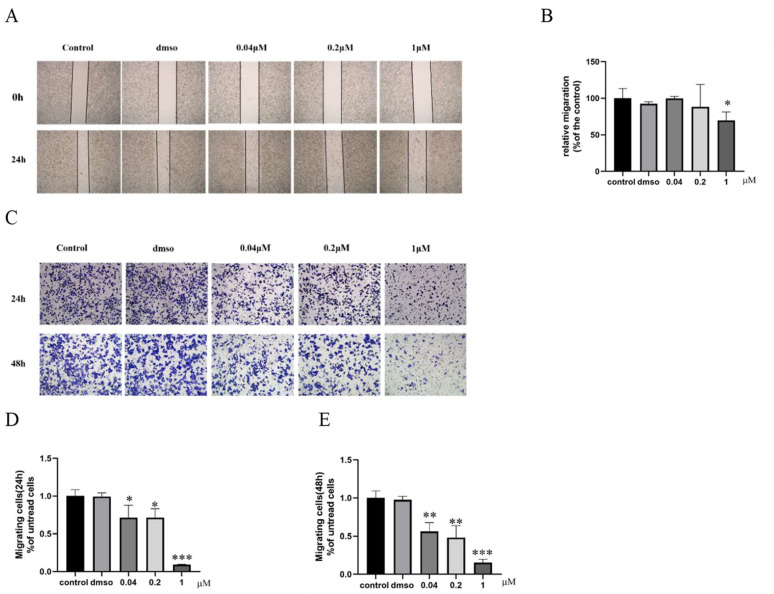
**CHJ04022R** inhibited the migration of A375 cells (**A**,**B**). The effect and statistical analysis of wound healing in A375 cells treated with indicated concentrations of **CHJ04022R** for 24 h in wound healing assay (**C**–**E**). The effect and statistical analysis of migration in A375 cells treated with **CHJ04022R** for 24 h and 48 h in transwell assay. Three independent experiments were performed. All data were expressed as the mean ± SD. * *p* < 0.05, ** *p* < 0.01, *** *p* < 0.001 compared to the control group.

Tumor metastasis often occurs in the advanced stage of malignant tumor and differs from clinical treatment. As an essential step in tumor metastasis, cell migration is worth exploring. Therefore, we investigated the effect of **CHJ04022R** on A375 cell migration. Wound healing and transwell assay showed that **CHJ04022R** effectively inhibited A375 cell migration in a dose-dependent manner (Figure 2A–E).

### 2.7. CHJ04022R Inhibited A375 Cells through G2/M Cell Cycle Arrest

**Figure 3:**
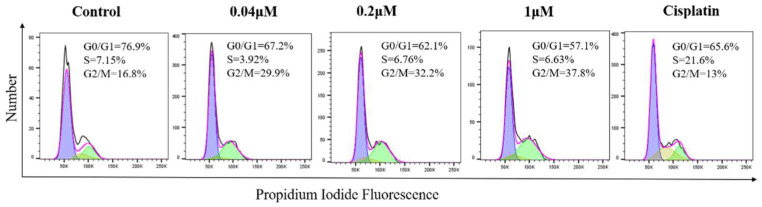

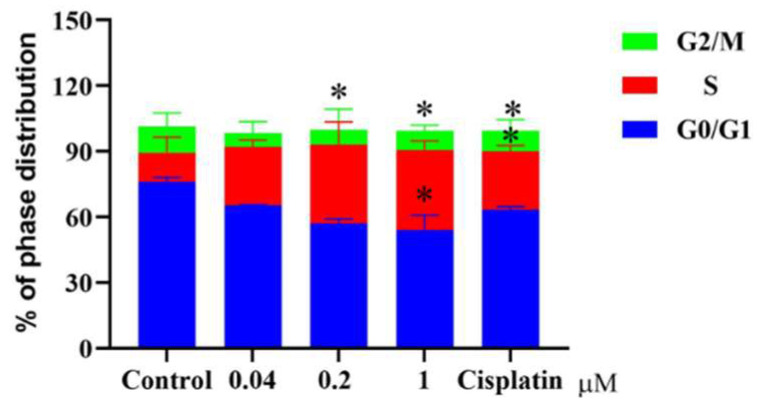
The cell cycle phase of A375 cells affected by **CHJ04022R** was analyzed by flow cytometry. All data were expressed as the mean ± SEM. * *p* < 0.05 compared to the control group.

The entire cell cycle of cell division is divided into two phases: interphase and mitosis. The interphase is divided into the G1 phase, S phase and G2 phase. Cells synthesize large amounts of RNA and proteins in the G1 phase, and prepare materials and energy for DNA replication in the S phase. Cells in the G2 phase prepare for division. After the end of the interphase, cells enter the M phase for orderly cell division. As seen in Figure 3, we found that the percentage of cells in the G0/G1 phase was obviously decreased after treatment with **CHJ04022R**, while cells in the G2/M phase was significantly increased dose-dependently, suggesting that **CHJ04022R** induced G2/M phase arrest in A375 cells.

### 2.8. CHJ04022R Induced Cell Apoptosis in A375 Cells

**Figure 4:**
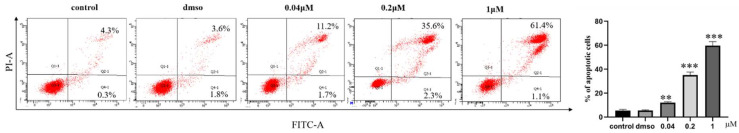
The percentage of cell apoptosis in A375 cells affected by different concentrations of **CHJ04022R** through flow cytometry using an Annexin V-FITC Apoptosis Detection Kit. All data were expressed as the mean ± SEM. ** *p* < 0.01, *** *p* < 0.001 compared to the control group.

Annexin V-FITC/PI apoptosis detection kit by flow cytometry was used to detect the apoptosis of A375 cells after **CHJ04022R** treatment. As seen in Figure 4, the percentage of the apoptotic population in A375 cells increased significantly, with a range of 4.3% to 61.4% at 48 h. Compared to the control, elevated cell apoptosis was observed in A375 cells after treatment with **CHJ04022R**.

### 2.9. Effects of CHJ04022R on the Expression of MMPs and the Activity of the PI3K/NF-κB Pathway

**Figure 5:**
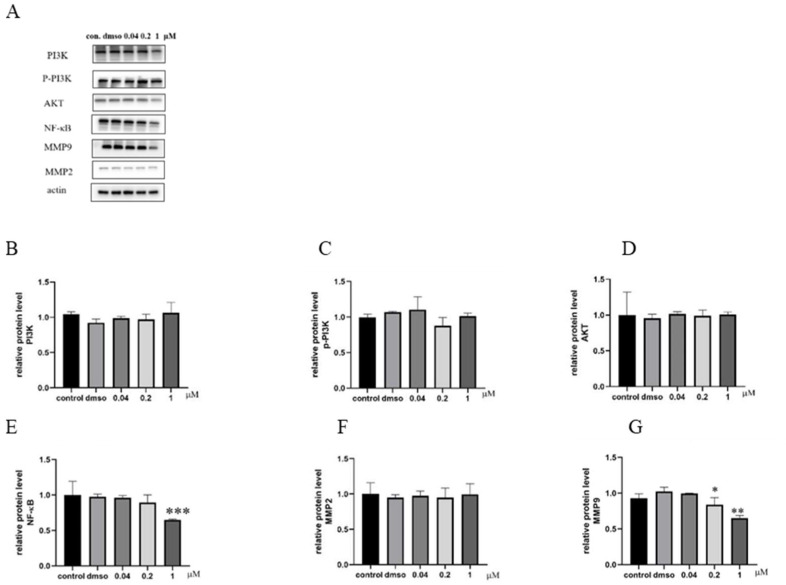
(**A**) The representative Western blot of PI3K, P-PI3K, AKT, NF-κB, MMP-2 and MMP-9 in A375 cells treated with indicated concentrations of **CHJ04022R** for 48 h. (**B**–**G**) The quantitative analysis of these proteins in A375 cells affected by **CHJ04022R**. Three independent experiments were performed. All data were expressed as the mean ± SEM. * *p* < 0.05, ** *p* < 0.01, *** *p* < 0.001 compared to the control group.

As indicated in Figure 5, **CHJ04022R** (0.2–1 μM) significantly decreased NF-κB and MMP-9 in A375 cells after 48 h of treatment, but did not significantly affect PI3K, P-PI3K, AKT and MMP2. Based on these findings, **CHJ04022R** may have suppressed the cell metastasis of A375 cells by suppressing MMP-9 through PI3K/NF-ĸB signaling pathways, but this needs further confirmation.

### 2.10. Safety Evaluation of CHJ04022R In Vivo

In order to test the safety of **CHJ04022R** in vivo, acute toxicity assay was performed. Rats were administered orally fixed doses of **CHJ04022R** (2 g/kg) and were then fed normally for 14 days. No signs of toxicity were found at the end (data not shown).

### 2.11. Effect of CHJ04022R on Tumor Growth in Nude Mice

**Figure 6:**
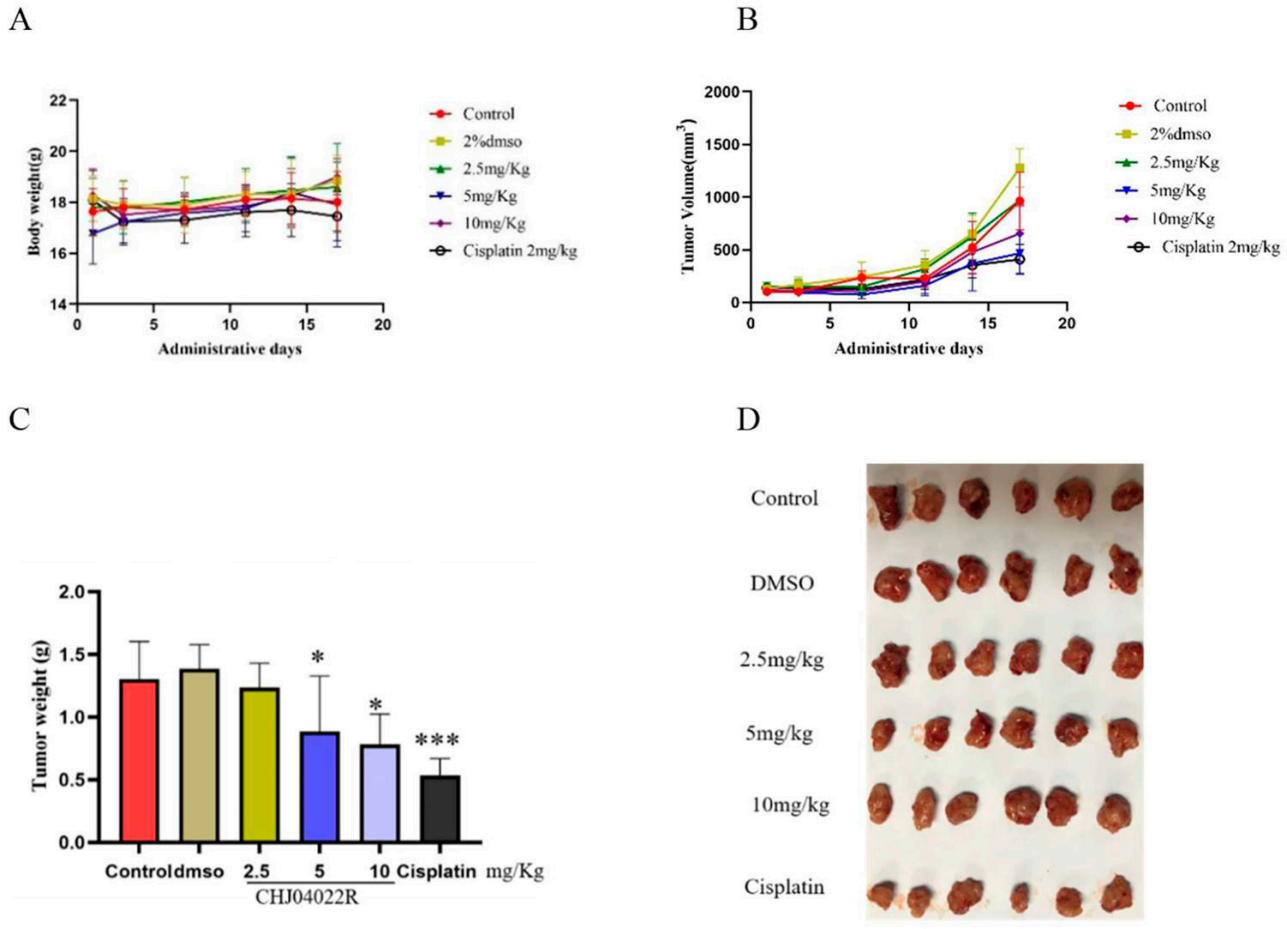
(**A**) The antitumor effect of **CHJ04022R** on the tumor growth of A375 cells in vivo. (**B**) Changes in the body weight of mice. (**C**) Changes in the tumor weight of mice. (**D**) Representative photographs of tumors, tumor weights and tumor volumes. * *p* < 0.05, *** *p* < 0.001 compared to the control group.

The in vivo antitumor effect of **CHJ04022R** was evaluated using the implanted A375 xenograft mouse model. Before the end of the experiment, the tumor size was measured with a caliper every three days to monitor the treatment effect in vivo. As shown in Figure 6 and Table 5, the mean tumor size of the control mice maintained a higher growth rate compared to the **CHJ04022R** treated mice. There was no significant difference in tumor volume between the different groups during the early stages of the experiment; however, the difference became significant after the 13th day. During treatment, appetite was normal and there was no significant difference in body weight. Data are expressed as mean ± SD (*n* = 10); * *p* < 0.05, *** *p* < 0.001 compared with the untreated control group.

## 3. Conclusions

In this work, we used a new SphK1 antagonist named **SAMS10** as the lead compound, and 50 new compounds were synthesized. The kinase activity of the 50 compounds was not as good as that of the lead compound **SASM10**. The reason for this phenomenon may be that **SAMS10** does not use the 4-methyl-piperidine group as its polar head in the protein cavity. Although the kinase activity of the compounds is not perfect, most of the compounds have good anticancer activity. Among them, the most potent compound **CHJ04022R** effectively inhibited A375 (IC_50_ = 2.95 μM). A number of Sphk1 inhibitor studies have demonstrated that Sphk1 inhibitor induces cell death, cell-cycle arrest and apoptosis in human cancer cells; however, no available information exists showing the ability of inhibiting migration and invasion of human melanoma cells.

We investigated the effect of **CHJ04022R** on the migration and invasion of A375 human melanoma cells and the results indicated that **CHJ04022R** significantly inhibited cell migration and promoted cell apoptosis and inducing G2/M phase arrest in A375 cells.

It has been reported that the expression and activity of MMPs against matrix macromolecules is linked to the promotion of cell invasiveness and metastasis. MMP-2/9 plays an important role in cancer invasion and metastasis, leading to transcription regulated by upstream regulatory factors NF-κ B, C-Jun and AP-1. Thus, we used Western blotting to investigate the expression of upstream regulatory factors of MMP-2/9 in A375 cells after exposure to **CHJ04022R** for 48 h. The results showed that **CHJ04022R** (0.2–1 μM) significantly reduced the protein expression of NF-ĸB levels when compared to the control groups. **CHJ04022R** (0.04–1 μM) significantly reduced the protein expression of MMP-9 levels when compared to the control groups. More interesting is that the inhibition of the PI3k/Akt pathway led to the decrease in the invasion of melanoma cells. The PI3K/Akt pathway plays a role in the MMPs for uPA gene regulation, cell survival and cell invasion. The AKT activation induced the invasion and metastasis of cancer cells by stimulating secretions of MMPs. NF-κB was linked with tumor cell proliferation, survival, invasion and metastasis. Based on these observations, we found that **CHJ04022R** may have affected the PI3K/NF-κB signaling pathway due to the reduction of protein expression, such as NF-κB and MMP9. Thus, **CHJ04022R** inhibited the migration and invasion of A375 cells via the suppression of the PI3K/NF-κB signaling pathway. While further research is required to determine the underlying details, these results provide new clues for the molecular mechanism of cell death induced by **CHJ04022R**.

Acute toxicity experiment indicated **CHJ04022R** exhibit good safety in vivo. Compared with the model control group, **CHJ04022R** showed dose-dependent inhibitory effect on the growth of transplanted tumor in nude mice and showed good antitumor activity.

These results suggest that **CHJ04022R** may act as new candidate drug for melanoma tumor and it could be an effective chemical tool for investigating the NF-κB signaling pathway related physiology and pathology and providing a promising chemical scaffold for further development.

## 4. Experimental Section

### 4.1. Materials and Methods

All chemicals were purchased from commercial sources and used as received. All final compounds are >95% pure by HPLC analysis. For detailed information on the materials and methods, please see Supporting information. Transwell cell culture chambers (Corning, NY, USA); MTT and dimethyl sulfoxide (DMSO) (Solarbio, Beijing, China); 8 mm pore size (Falcon, Corning, NY, USA); PI3 Kinase, p-PI3 Kinase, Akt, NF-κB p65, MMP-2, MMP-9, β-Actin, (CST, Danvers, MA, USA); antirabbit IgG (zsbio, Beijing, China); Annexin V-FITC apoptosis Detection Kit (C1062L, Beyotime, Shanghai, China); PI/RNase Staining Buffer (#550825, BD, Franklin Lakes, NJ, USA)

The A375 cell line was obtained from the National Collection of Authenticated Cell Cultures.

### 4.2. Synthesis of Intermediates (***5***)

The intermediate compounds (**5**) were synthesized from the raw material vanillin **1** through five steps, and Scheme 1 showed their synthetic route.

**4-(2-hydroxyethoxy)-3-methoxybenzaldehyde (2):**

To a stirred solution of vanillin (**1**) (5 g, 32.86 mmol) and 2-bromoethanol (5.8 g, 82.16 mmol) in acetonitrile (262.88 mL), potassium carbonate (6.8 g, 49.29 mmol) was added. The mixture was heated and stirred at 100 °C for 24 h, and detected by TLC (dichloromethane:methanol = 22:1). The whole was extracted with ethyl acetate. The extract was washed with H_2_O and brine, then dried over Na_2_SO_4_. The filtrate was concentrated under reduced pressure to give an oily residue, which was purified using silica gel chromatography (dichloromethane: methanol = 50:1) to afford compound **2** (6.44 g, 100%) as a white solid. ^1^H-NMR (CDCl_3_, 600 MHz) δ (ppm): 9.86 (s, 1H), 7.45 (dd, *J* = 4.4, 1.2 Hz, 1H), 7.43 (d, *J* = 1.8 Hz, 1H), 7.01 (d, *J* = 8.1 Hz, 1H), 4.21 (m, 2H), 4.04 (m, 2H), 3.93 (s, 3H). ^13^C-NMR (CDCl_3_, 150 MHz) δ (ppm): 190.97, 153.62, 150.40, 130.49, 126.77, 112.12, 109.25, 70.64, 61.07, 55.96. HR-MS (ESI): *m*/*z* calcd for C_10_H_13_O_4_ [M + H]^+^: 196.0814. found: 196.0736.

**2-(4-formyl-2-methoxyphenoxy)ethyl 4-methylbenzenesulfonate (3)**

4-(2-hydroxyethoxy)-3-methoxybenzaldehyde (**2**) (6.4 g, 32.62 mmol), *p*-toluenesulfonyl chloride (18.66 g, 97.86 mmol), triethylamine (22.61 mL, 163.1 mmol) and DMAP (0.8 g, 6.524 mmol) were dissolved in dichloromethane (280 mL). The mixture was stirred at room temperature for 24 h, and detected by TLC (dichloromethane:methanol = 20:1). The whole was extracted with dichloromethane. The extract was washed with H_2_O and brine, then dried over Na_2_SO_4_. The filtrate was concentrated under reduced pressure to give an oily residue, which was purified using silica gel chromatography (dichloromethane: methanol = 20:1) to afford a white solid compound **3** (10.27 g, 90%). ^1^H-NMR (CDCl_3_, 600 MHz) δ (ppm): 9.86 (s, 1H), 7.81 (d, *J* = 8.3 Hz, 2H), 7.41 (m, 2H), 7.33 (d, *J* = 8.0Hz, 2H), 4.42 (m, 2H), 4.32 (m, 2H), 3.90 (s, 3H), 2.44 (s, 3H). ^13^C-NMR (CDCl_3_, 150 MHz) δ (ppm): 190.84, 152.90, 150.04, 145.05, 132.70, 130.91, 129.87, 129.87, 128.01, 128.01, 126.38, 112.52, 109.71, 67.58, 66.56, 56.01, 21.68. HR-MS (ESI): *m*/*z* calcd for C_17_H_19_O_6_S [M + H]^+^: 351.0902. found: 351.0824.

The intermediate compounds (**5**) are obtained by substituting the *p*-toluenesulfonyl group of compound (**3**) with different amine groups. To a stirred solution of compound **3** (1.56 g, 4.45 mmol), amines (6.68 mmol) and potassium carbonate (0.68 g, 4.9 mmol) in CH_3_CN (134 mL) under argon was added tetrabutylammonium iodide (328.74 mg, 0.89 mmol) at 50 °C. The mixture was heated and stirred for 24 h, and detected by TLC (dichloromethane: methanol = 10:1). The organic phase was extracted from the mixture, and the crude product was separated and purified by silica gel column chromatography. The pure product was dissolved in methylamine solution (200 mL), and the whole was stirred at room temperature for 24 h under oxygen-free conditions. After the reaction solution was evaporated to dryness, it was dissolved in methanol (200 mL). Sodium borohydride (0.9 g, 15.81 mmol) was slowly added at 0 °C. The whole was moved to room temperature and stirred for 20 min. After the reaction was quenched with saturated NaHCO_3_, the whole was extracted with ethyl acetate. The extract was washed with H_2_O and brine, then dried over Na_2_SO_4_. The filtrate was concentrated under reduced pressure to give an oily residue. The crude product was separated by silica gel column chromatography to obtain intermediate compound (**5**) with a yield of 60–70%.

**1-(3-methoxy-4-(2-(4-methylpiperidin-1-yl)ethoxy)phenyl)-*N*-methylmethanamine (5-1):**

A white solid, yield 70%, ^1^H-NMR (CDCl_3_, 600 MHz) δ (ppm): 6.88 (d, *J* = 1.7 Hz, 1H), 6.84 (m, 2H), 4.14 (t, *J* = 6.0 Hz, 2H), 3.87 (s, 3H), 3.68 (s, 2H), 2.96 (d, *J* = 12.0 Hz, 2H), 2.82 (t, *J* = 12.0 Hz, 2H), 2.46 (s, 3H), 2.08 (m, 3H), 1.63 (d, *J* = 12.0 Hz, 2H), 1.37 (m, 1H), 1.28 (m, 2H), 0.92 (d, *J* = 6.0Hz, 3H). ^13^C-NMR (CDCl_3_, 150 MHz) δ (ppm): 149.68, 148.00, 129.46, 121.25, 113.21, 112.45, 66.95, 57.33, 56.08, 54.48 (3C), 34.18 (3C), 30.54, 21.85. HR-MS (ESI): *m*/*z* calcd for C_17_H_29_N_2_O_2_ [M + H]^+^: 293.2229. found: 293.2151.

**1-(3-methoxy-4-(2-(pyrrolidin-1-yl)ethoxy)phenyl)-*N*-methylmethanamine (5-2):**

A colorless oil, yield 60%, ^1^H-NMR (CDCl_3_, 400 MHz) δ: 6.92 (s, 1H), 6.84 (m, 2H), 4.14 (t, *J* = 8.0 Hz, 2H), 3.87 (s, 3H), 3.70 (s, 2H), 2.94 (t, *J* = 8.0 Hz, 2H), 2.81 (s, 1H), 2.63 (s, 4H), 2.45 (s, 3H), 1.80 (s, 4H). ^13^C-NMR (CDCl_3_, 100 MHz) δ: 149.51, 147.41, 132.58, 120.48, 113.20, 111.96, 67.98, 55.92, 55.66, 54.90, 54.61 (2C), 35.71, 23.48 (2C). HRMS (ESI): *m*/*z* calcd for C_15_H_25_N_2_O_2_ [M + H]^+^: 265.1916. found: 265.1867.

***N*** **,*N*-diethyl-2-(2-methoxy-4-((methylamino)methyl)phenoxy)ethan-1-amine (5-3):**

A colorless oil, yield 65%, ^1^H-NMR (CDCl_3_, 400 MHz) δ (ppm): 6.90 (s, 1H), 6.85 (m, 2H), 4.08 (t, *J* = 8.0 Hz, 2H), 3.87 (s, 3H), 3.69 (s, 2H), 2.92 (t, *J* = 4.0 Hz, 2H), 2.64 (q, *J* = 8.0 Hz, 4H), 2.44 (s, 4H), 1.06 (t, *J* = 8.0 Hz, 6H). ^13^C-NMR (CDCl_3_, 100 MHz) δ (ppm): 149.37, 147.43, 132.62, 120.43, 112.81, 111.89, 67.34, 55.93, 55.76, 51.70, 47.79 (2C), 35.81, 11.82 (2C), HRMS (ESI): *m*/*z* calcd for C_15_H_27_N_2_O_2_ [M + H]^+^: 267.2073. found: 267.2047.

**1-(4-(2-(1H-imidazol-1-yl)ethoxy)-3-methoxyphenyl)-*N*-methylmethanamine (5-4):**

A white solid, yield 70%, ^1^H-NMR (CDCl_3_, 400 MHz) δ (ppm): 7.63 (s, 1H), 7.07 (m, 2H), 6.91 (s, 1H), 6.76 (m, 2H), 4.33 (t, *J* = 4.0 Hz, 2H), 4.23 (t, *J* = 4.0 Hz, 2H), 3.86 (s, 3H), 3.69 (s, 2H), 2.45 (s, 3H), 2.15 (s, 1H). ^13^C-NMR (CDCl_3_, 100 MHz) δ (ppm): 149.93, 146.59, 137.66, 134.33, 129.35, 120.38, 119.56, 114.45, 112.23, 69.05, 55.94, 55.73, 46.61, 35.90. HRMS (ESI): *m*/*z* calcd for C_14_H_20_N_3_O_2_ [M + H]^+^: 262.1556. found: 262.3330.

### 4.3. General Synthesis Method of 1-Phenoxy-2,3-Epoxypropane Compounds (***6***)

Different substituted phenols, potassium carbonate (9.0 mmol) and tetrabutylammonium iodide (0.6 mmol), were dissolved in acetonitrile (16 mL). Under the condition of nitrogen, epibromopropane (12 mmol) was added dropwise. The whole was heated and stirred at 50 °C for 36 h, detected by TLC (petroleum ether: ethyl acetate = 20:1). The whole was extracted with ethyl acetate. The extract was washed with H_2_O and brine, then dried over Na_2_SO_4_. The filtrate was concentrated under reduced pressure to give an oily residue. The crude product was separated by silica gel column chromatography (Petroleum ether: ethyl acetate = 20:1) to obtain intermediate compound (**7**) with a yield of 75–85%. Table S1 shows the structures of phenoxy-2,3-epoxypropane compounds (**6**).

### 4.4. Synthesis and Structure Confirmation of Target Compounds (CHJ)

Compounds **CHJ** were synthesized by nucleophilic reaction between 1-phenoxy-2,3-epoxypropanes (**6**) (1.0 eq) and intermediate compounds (**5**) (1.2 eq). In their isopropanol solution, the catalytic amount of pyridine was added. The whole was heated to reflux for 6 h and detected by TLC (dichloromethane:methanol = 10:1). The resulting mixture was extracted with ethyl acetate. The extract was washed with H_2_O and brine, then dried over Na_2_SO_4_. The filtrate was concentrated under reduced pressure to afford crude product that was purified by silica gel column chromatography (dichloromethane:methanol = 20:1) to give the title compounds (**CHJ**) with yields of 80–90%.

**1-(2,6-dichlorophenoxy)-3-((3-methoxy-4-(2-(4-methylpiperidin-1-yl)ethoxy)benzyl)(methyl)amino)propan-2-ol (CHJ02029):**

A colorless oil, yield 88%, ^1^H-NMR (CD_3_OD, 400 MHz) δ (ppm): 7.36 (d, *J* = 8.0 Hz, 2H), 7.08 (t, *J* = 8.0 Hz, 1H), 7.01 (s, 1H), 6.87 (m, 2H), 4.15 (m, 3H), 3.99 (m, 2H), 3.80 (s, 3H), 3.59 (m, 2H), 3.14 (d, *J* = 11.20 Hz, 2H), 2.92 (t, *J* = 5.60 Hz, 2H), 2.74 (m, 1H), 2.59 (dd, *J* = 12.8, 7.6 Hz, 1H), 2.32 (m, 5H), 1.70 (d, *J* = 12.80 Hz, 2H), 1.45 (s, 1H), 1.30 (m, 2H), 0.95 (d, *J* = 6.4 Hz, 3H). ^13^C-NMR (CD_3_OD, 100 MHz) δ (ppm): 151.27, 149.68, 147.30, 131.70, 129.04, 128.89 (3C), 125.31, 121.62, 113.84, 113.09, 75.76, 67.86, 66.25, 62.02, 59.27, 56.86, 55.00, 53.81 (2C), 41.86, 33.04 (2C), 29.92, 20.60. IR (KBr, cm^−1^): 2947, 2926, 2872, 2841, 2792, 2360, 2340, 1651, 1592, 1511, 1475, 1455, 1367, 1286, 1262, 1230, 1127, 1036, 979, 937, 863, 807, 670. HR-MS (ESI): *m*/*z* calcd for C_26_H_37_Cl_2_N_2_O_4_ [M + H]^+^: 511.2130. found: 511.2047. UPLC: t_R_ = 6.75 min; purity ≥ 98% (UV: 210 nm).

**1-(4-bromo-3-(trifluoromethyl)phenoxy)-3-((3-methoxy-4-(2-(4-methylpiperidin-1-yl)ethoxy)benzyl)(methyl)amino)propan-2-ol (CHJ02049):**

A colorless oil, yield 87%, ^1^H-NMR (CD_3_OD, 400 MHz) δ (ppm): 7.64 (d, *J* = 8.80 Hz, 1H), 7.26 (s, 1H), 7.02 (d, *J* = 8.80 Hz, 1H), 6.95 (s, 1H), 6.83 (q, *J* = 8.0 Hz, 2H), 4.08 (m, 4H), 3.91 (m, 1H), 3.75 (s, 3H), 3.50 (q, *J* = 12.80 Hz, 2H), 3.03 (d, *J* = 11.60 Hz, 2H), 2.80 (t, *J* = 5.60 Hz, 2H), 2.62 (dd, *J* = 12.40, 5.60 Hz, 1H), 2.48 (dd, *J* = 12.40, 6.40 Hz, 1H), 2.32 (s, 3H), 2.17 (t, *J* = 11.60 Hz, 2H), 1.66 (d, *J* = 12.40 Hz, 2H), 1.39 (s, 1H), 1.29 (m, 2H), 0.94 (d, *J* = 6.40 Hz, 3H). ^13^C-NMR (CD_3_OD, 100 MHz) δ (ppm): 158.33, 149.62, 147.45, 135.7 (2C), 131.91, 121.46 (2C), 118.91, 114.35, 114.29, 113.48, 113.06, 70.99, 67.29, 66.69, 62.29, 58.70, 57.12, 54.94, 54.00 (2C), 42.25, 33.48 (2C), 33.25, 20.75. IR (KBr, cm^−1^): 2926, 2872, 2849, 2793, 2370, 2323, 1684, 1651, 1556, 1512, 1474, 1455, 1419, 1367, 1330, 1313, 1260, 1235, 1139, 1035, 980, 936, 879, 809, 753. HR-MS (ESI): *m*/*z* calcd for C_27_H_37_BrF_3_N_2_O_4_ [M + H]^+^: 589.1889. found: 589.2404. UPLC: t_R_ = 6.77 min; purity ≥ 99% (UV: 210 nm).

**1-(2,5-bis(trifluoromethyl)phenoxy)-3-((3-methoxy-4-(2-(4-methylpiperidin-1-yl)ethoxy)benzyl)(methyl)amino)propan-2-ol (CHJ02050):**

A white solid, yield 85%, m. p: 70–72 °C, ^1^H-NMR (CD_3_OD, 400 MHz) δ (ppm): 7.77 (d, *J* = 8.0 Hz, 1H), 7.46 (s, 1H), 7.38 (d, *J* = 8.0 Hz, 1H), 6.96 (s, 1H), 6.83 (q, *J* = 8.0 Hz, 2H), 4.13 (m, 5H), 3.76 (s, 3H), 3.51 (m, 2H), 3.03 (d, *J* = 11.20 Hz, 2H), 2.80 (t, *J* = 5.60 Hz, 2H), 2.63 (m, 2H), 2.30 (s, 3H), 2.17 (t, *J* = 11.60 Hz, 2H), 1.66 (d, *J* = 12.40 Hz, 2H), 1.40 (s, 1H), 1.29 (m, 2H), 0.94 (d, *J* = 6.40 Hz, 3H). ^13^C-NMR (CD_3_OD, 100 MHz) δ (ppm): 157.29, 149.66, 147.41, 131.89, 127.72, 127.67, 121.67, 121.40, 116.72, 116.68, 113.59, 112.97, 109.94, 109.90, 71.45, 67.17, 66.71, 62.28, 59.06, 57.11, 54.90, 53.98 (2C), 41.93, 33.46 (2C), 30.24, 20.73. IR (KBr, cm^−1^): 3562, 3354, 2945, 2877, 2831, 2800, 1624, 1595, 1517, 1463, 1435, 1330, 1259, 1232, 1174, 1132, 1087, 1043, 1022, 962, 910, 866, 833, 804, 750, 673. HR-MS (ESI): *m*/*z* calcd for C_28_H_37_F_6_N_2_O_4_ [M + H]^+^: 579.2658. found: 579.2549. UPLC: t_R_ = 6.58 min; purity ≥ 99% (UV: 210 nm).

**1-(3-bromophenoxy)-3-((3-methoxy-4-(2-(4-methylpiperidin-1-yl)ethoxy)benzyl)(methyl)amino)propan-2-ol (CHJ03001):**

A colorless oil, yield 86%, ^1^H-NMR (CDCl_3_, 400 MHz) δ (ppm): 7.10 (m, 3H), 6.82 (m, 4H), 4.14 (m, 3H), 3.94 (d, *J* = 4.80 Hz, 2H), 3.85 (s, 3H), 3.62 (d, *J* = 13.60 Hz, 1H), 3.45 (d, *J* = 13.20 Hz, 1H), 2.98 (d, *J* = 11.20 Hz, 2H), 2.84 (t, *J* = 6.0 Hz, 2H), 2.62 (m, 1H), 2.49 (dd, *J* = 12.40, 3.6 Hz, 1H), 2.29 (s, 3H), 2.11 (t, *J* = 11.60 Hz, 2H), 1.64 (d, *J* = 12.40 Hz, 2H), 1.30 (m, 3H), 0.93 (d, *J* = 4.0 Hz, 3H). ^13^C-NMR (CDCl_3_, 100 MHz) δ (ppm): 159.54, 149.44, 147.60, 131.18, 130.53, 124.07, 122.76, 121.30, 117.86, 113.58, 112.97, 112.49, 70.63, 66.81, 66.04, 62.37, 59.25, 57.40, 55.97, 54.49 (2C), 42.26, 34.19 (2C), 30.57, 21.87. IR (KBr, cm^−1^): 2947, 2924, 2871, 2846, 2792, 2360, 2340, 1651, 1591, 1572, 1512, 1476, 1463, 1459, 1368, 1324, 1283, 1261, 1229, 1157, 1138, 1090, 1035, 991, 936, 861, 804, 800, 674. HR-MS (ESI): *m*/*z* calcd for C_26_H_38_BrN_2_O_4_ [M + H]^+^: 521.2015. found: 521.1945. UPLC: t_R_ = 6.72 min; purity ≥ 97% (UV: 210 nm).

**1-(2-bromophenoxy)-3-((3-methoxy-4-(2-(4-methylpiperidin-1-yl)ethoxy)benzyl)(methyl)amino)propan-2-ol (CHJ03003):**

A colorless oil, yield 90%, ^1^H-NMR (CDCl_3_, 400 MHz) δ (ppm): 7.52 (d, *J* = 7.60 Hz, 1H), 7.23 (d, *J* = 8.0 Hz, 1H), 6.85 (m, 5H), 4.14 (t, *J* = 6.0 Hz, 3H), 4.03 (d, *J* = 4.40 Hz, 2H), 3.84 (s, 3H), 3.72 (q, *J* = 7.20 Hz, 1H), 3.63 (d, *J* = 12.80 Hz, 1H), 3.47 (d, *J* = 12.80 Hz, 1H), 2.98 (d, *J* = 11.20 Hz, 2H), 2.84 (t, *J* = 6.40 Hz, 2H), 2.72 (m, 1H), 2.59 (dd, *J* = 12.0, 3.60 Hz, 1H), 2.31 (s, 3H), 2.11 (t, *J* = 11.20 Hz, 2H), 1.64 (d, *J* = 12.40 Hz, 2H), 1.27 (m, 3H), 0.93 (d, *J* = 6.0 Hz, 3H). ^13^C-NMR (CDCl_3_, 100 MHz) δ (ppm): 155.10, 149.41, 147.54, 133.30, 131.32, 128.46, 122.19, 121.33, 113.54, 112.94, 112.52, 112.41, 71.39, 66.74, 66.25, 62.47, 59.35, 58.43, 57.39, 55.96, 54.47, 42.43, 34.17, 30.57, 21.86, 18.45. IR (KBr, cm^−1^): 2947, 2924, 2871, 2844, 2792, 2361, 2340, 1589, 1513, 1480, 1462, 1417, 1368, 1323, 1276, 1261, 1232, 1158, 1138, 1084, 1053, 1030, 979, 939, 872, 806, 749. HR-MS (ESI): *m*/*z* calcd for C_26_H_38_BrN_2_O_4_ [M + H]^+^: 521.2015. found: 521.1943. UPLC: t_R_ = 6.80 min; purity ≥ 99% (UV: 210 nm).

**1-(2-isopropylphenoxy)-3-((3-methoxy-4-(2-(4-methylpiperidin-1-yl)ethoxy)benzyl)(methyl)amino)propan-2-ol (CHJ03004):**

A colorless oil, yield 87%, ^1^H-NMR (CDCl_3_, 400 MHz) δ (ppm): 7.2 (d, *J* = 7.60 Hz, 1H), 7.13 (t, *J* = 7.60 Hz, 1H), 6.92 (t, *J* = 7.20 Hz, 1H), 6.82 (m, 4H), 4.14 (m, 3H), 3.99 (m, 2H), 3.84 (s, 3H), 3.65 (d, *J* = 13.20 Hz, 1H), 3.45 (d, *J* = 12.80 Hz, 1H), 3.26 (m, 1H), 2.98 (d, *J* = 11.20 Hz, 2H), 2.84 (t, *J* = 6.40 Hz, 2H), 2.69 (m, 1H), 2.53 (dd, *J* = 12.0, 3.20 Hz, 1H), 2.31 (s, 3H), 2.11 (t, *J* = 11.2 Hz, 2H), 1.64 (d, *J* = 12.0 Hz, 2H), 1.30 (m, 3H), 1.19 (d, *J* = 6.80 Hz, 6H), 0.96 (d, *J* = 6.0 Hz, 3H). ^13^C-NMR (CDCl_3_, 100 MHz) δ (ppm): 155.82, 149.43, 147.56, 137.05, 131.31, 126.55, 126.07, 121.26, 120.89, 112.93, 112.45, 111.30, 70.34, 66.78, 66.35, 62.47, 59.67, 57.40, 55.94, 54.48 (2C), 42.36, 34.19 (2C), 30.57, 26.90, 22.64 (2C), 21.88. IR (KBr, cm^−1^): 2950, 2925, 2870, 2792, 2360, 2340, 1597, 1513, 1491, 1452, 1418, 1365, 1323, 1261, 1238, 1193, 1138, 1088, 1033, 1030, 980, 937, 878, 822, 805, 751. HR-MS (ESI): *m*/*z* calcd for C_29_H_45_N_2_O_4_ [M + H]^+^: 485.3379. found: 485.3330. UPLC: t_R_ = 6.75 min; purity ≥ 99% (UV: 210 nm).

**1-(4-bromo-2-methoxyphenoxy)-3-((3-methoxy-4-(2-(4-methylpiperidin-1-yl)ethoxy)benzyl)(methyl)amino)propan-2-ol (CHJ03005):**

A colorless oil, yield 90%, ^1^H-NMR (CD_3_OD, 400 MHz) δ (ppm): 7.06 (s, 1H), 7.00 (d, *J* = 8.40 Hz, 1H), 6.95 (s, 1H), 6.83 (m, 3H), 4.10 (m, 3H), 3.96 (dd, *J* = 9.60, 5.60 Hz, 1H), 3.85 (dd, *J* = 9.60, 5.60 Hz, 1H), 3.77 (d, *J* = 12.0 Hz, 6H), 3.53 (m, 2H), 3.06 (d, *J* = 11.60 Hz, 2H), 2.83 (t, *J* = 5.88 Hz, 2H), 2.63 (dd, *J* = 13.44, 5.60 Hz, 1H), 2.49 (dd, *J* = 13.44, 6.80 Hz, 1H), 2.31 (s, 3H), 2.20 (t, *J* = 11.60 Hz, 2H), 1.67 (d, *J* = 12.80 Hz, 2H), 1.40 (s, 1H), 1.28 (m, 2H), 0.94 (d, *J* = 6.40 Hz, 3H). ^13^C-NMR (CD_3_OD, 100 MHz) δ (ppm): 150.42, 149.62, 147.91, 147.36, 131.88, 123.29, 121.47, 115.25, 114.88, 113.54, 113.05, 112.79, 71.91, 67.50, 66.61, 62.22, 58.89, 57.07, 55.34, 54.94, 53.96 (2C), 42.13, 33.39 (2C), 30.18, 20.71. IR (KBr, cm^−1^): 2946, 2924, 2843, 2792, 2360, 2340, 1589, 1556, 1539, 1506, 1459, 1418, 1398, 1364, 1324, 1255, 1225, 1183, 1136, 1084, 1029, 936, 857, 797, 670. HR-MS (ESI): *m*/*z* calcd for C_27_H_40_BrN_2_O_5_ [M + H]^+^: 551.2101. found: 551.2094. UPLC: t_R_ = 6.43 min; purity ≥ 98% (UV: 210 nm).

**1-((3-methoxy-4-(2-(4-methylpiperidin-1-yl)ethoxy)benzyl)(methyl)amino)-3-(2-methoxy-4-propylphenoxy)propan-2-ol (CHJ03011):**

A white solid, yield 83%, m. p: 45–47 °C, ^1^H-NMR (CD_3_OD, 400 MHz) δ (ppm): 6.97 (s, 1H), 6.87 (d, *J* = 8.0 Hz, 1H), 6.80 (m, 3H), 6.69 (d, *J* = 8.0 Hz, 1H), 4.10 (m, 3H), 3.96 (dd, *J* = 9.60, 3.60 Hz, 1H), 3.84 (dd, *J* = 9.60, 6.40 Hz, 1H), 3.78 (d, *J* = 13.60 Hz, 6H), 3.54 (m, 2H), 3.05 (d, *J* = 11.60 Hz, 2H), 2.82 (t, *J* = 5.60 Hz, 2H), 2.61 (dd, *J* = 12.80, 5.20 Hz, 1H), 2.51 (q, *J* = 7.20 Hz, 3H), 2.30 (s, 3H), 2.19 (t, *J* = 11.60 Hz, 2H), 1.63 (m, 4H), 1.40 (s, 1H), 1.28 (m, 2H), 0.94 (d, *J* = 7.20 Hz, 6H). ^13^C-NMR (CD_3_OD, 100 MHz) δ (ppm): 149.66, 149.33, 147.40, 146.45, 136.10, 131.85, 121.50, 120.41, 113.95, 113.60, 113.07, 112.54, 72.26, 67.62, 66.67, 62.16, 59.08, 57.08, 55.14, 54.96, 53.97 (2C), 42.01, 37.26, 33.41 (2C), 30.19, 24.48, 20.71, 12.68. IR (KBr, cm^−1^): 2922, 2868, 2791, 2360, 2340, 1597, 1516, 1458, 1419, 1371, 1330, 1261, 1230, 1138, 1091, 1031, 970, 850, 804, 750, 646, 553, 489. HR-MS (ESI): *m*/*z* calcd for C_30_H_47_N_2_O_5_ [M + H]^+^: 515.3485. found: 515.3423. UPLC: t_R_ = 6.79 min; purity ≥ 98% (UV: 210 nm).

**1-((3-methoxy-4-(2-(4-methylpiperidin-1-yl)ethoxy)benzyl)(methyl)amino)-3-(4-pentadecylphenoxy)propan-2-ol (CHJ03012):**

A white solid, yield 85%, m. p: 40–42 °C, ^1^H-NMR (CD_3_OD, 400 MHz) δ (ppm): 7.13 (t, *J* = 7.60 Hz,1H), 6.97 (s, 1H), 6.84 (m, 2H), 6.72 (m, 3H), 4.11 (m, 3H), 3.98 (m, 1H), 3.86 (m, 1H), 3.76 (s, 3H), 3.55 (m, 2H), 3.05 (d, *J* = 11.60 Hz, 2H), 2.81 (t, *J* = 5.60 Hz, 2H), 2.59 (m, 4H), 2.30 (m, 3H), 2.18 (t, *J* = 11.60 Hz, 2H), 1.64 (m, 4H), 1.28 (s, 27H), 0.94 (d, *J* = 6.40 Hz, 3H), 0.89 (t, *J* = 6.0 Hz, 3H). ^13^C-NMR (CD_3_OD, 100 MHz) δ (ppm): 159.02, 149.69, 147.42, 144.23, 131.93, 128.79, 121.47, 120.62, 114.35, 113.65, 113.08, 111.38, 70.34, 67.53, 66.71, 62.22, 59.19, 57.11, 54.98, 53.98 (2C), 53.38, 42.07, 35.56, 33.43 (2C), 31.67, 31.21, 30.21, 29.35 (6C), 29.20, 29.07, 28.91, 22.33, 20.72, 13.04. IR (KBr, cm^−1^): 2924, 2852, 2794, 2360, 2340, 1591, 1514, 1458, 1367, 1325, 1263, 1151, 1085, 1035, 937, 869, 806, 775, 694. HR-MS (ESI): *m*/*z* calcd for C_41_H_69_N_2_O_5_ [M + H]^+^: 653.5257. found: 653.5163. UPLC: t_R_ = 6.81 min; purity ≥ 97% (UV: 210 nm).

**1-(4-bromophenoxy)-3-((3-methoxy-4-(2-(4-methylpiperidin-1-yl)ethoxy)benzyl)(methyl)amino)propan-2-ol (CHJ03014):**

A colorless oil, yield 81%, ^1^H-NMR (CD_3_OD, 400 MHz) δ (ppm): 7.35 (d, *J* = 8.0 Hz, 2H), 6.95 (s, 1H), 6.82 (m, 4H), 4.10 (t, *J* = 5.60 Hz, 2H), 4.05 (m, 1H), 3.95 (dd, *J* = 9.60, 3.20 Hz, 1H), 3.84 (dd, *J* = 9.60, 6.0 Hz, 1H), 3.77 (s, 3H), 3.50 (q, *J* = 12.80 Hz, 2H), 3.03 (d, *J* = 11.20 Hz, 2H), 2.80 (t, *J* = 5.60 Hz, 2H), 2.61 (dd, *J* = 12.80, 6.0 Hz,1H), 2.48 (dd, *J* = 12.80, 7.20 Hz, 1H), 2.30 (s, 3H), 2.17 (t, *J* = 11.60 Hz, 2H), 1.65 (d, *J* = 12.80 Hz, 2H), 1.40 (s, 1H), 1.28 (m, 2H), 0.93 (d, *J* = 6.40 Hz, 3H). ^13^C-NMR (CD_3_OD, 100 MHz) δ (ppm): 158.28, 149.64, 147.44, 131.89 (2C), 131.89, 121.49, 116.19 (2C), 113.56, 113.10, 112.35, 70.66, 67.42, 66.70, 62.27, 58.96, 57.12, 54.88, 53.99 (2C), 42.21, 33.47 (2C), 30.23, 20.76. IR (KBr, cm^−1^): 2947, 2925, 2871, 2844, 2792, 2360, 2331, 1591, 1556, 1512, 1489, 1458, 1418, 1368, 1322, 1285, 1245, 1157, 1074, 1034, 980, 937, 879, 863, 821, 756, 647. HR-MS (ESI): *m*/*z* calcd for C_26_H_38_BrN_2_O_4_ [M + H]^+^: 521.2015. found: 521.1975. UPLC: t_R_ = 6.75 min; purity ≥ 99% (UV: 210 nm).

**1-(3-isopropylphenoxy)-3-((3-methoxy-4-(2-(4-methylpiperidin-1-yl)ethoxy)benzyl)(methyl)amino)propan-2-ol (CHJ03015):**

A colorless oil, yield 89%, ^1^H-NMR (CD_3_OD, 400 MHz) δ (ppm): 7.15 (t, *J* = 7.60 Hz, 1H), 6.97 (s, 1H), 6.82 (m, 4H), 6.69 (d, *J* = 8.0 Hz, 1H), 4.10 (m, 3H), 3.97 (m, 1H), 3.86 (m, 1H), 3.76 (s, 3H), 3.54 (m, 2H), 3.03 (d, *J* = 11.20 Hz, 2H), 2.82 (m, 3H), 2.63 (dd, *J* = 12.80, 5.60 Hz, 1H), 2.51 (dd, *J* = 12.40, 6.8 Hz, 1H), 2.30 (s, 3H), 2.17 (t, *J* = 11.60 Hz, 2H), 1.65 (d, *J* = 12.40 Hz, 2H), 1.40 (s, 1H), 1.24 (m, 8H), 0.94 (d, *J* = 6.0 Hz, 3H). ^13^C-NMR (CD_3_OD, 100 MHz) δ (ppm): 159.09, 150.37, 149.68, 147.44, 131.90, 128.90, 121.47, 118.55, 113.62, 113.09, 112.54, 111.36, 70.39, 67.54, 66.74, 62.21, 59.25, 57.12, 54.99 (3C), 42.05, 34.05, 33.46 (2C), 30.23, 23.03 (2C), 20.75. IR (KBr, cm^−1^): 2952, 2925, 2871, 2844, 2792, 2360, 2340, 1606, 1588, 1513, 1486, 1460, 1418, 1366, 1320, 1262, 1233, 1286, 1138, 1088, 1037, 1003, 980, 940, 870, 804, 789, 754, 700. HR-MS (ESI): *m*/*z* calcd for C_29_H_45_N_2_O_4_ [M + H]^+^: 485.3379. found: 485.3296. UPLC: t_R_ = 6.72 min; purity ≥ 98% (UV: 210 nm).

**1-((3-methoxy-4-(2-(4-methylpiperidin-1-yl)ethoxy)benzyl)(methyl)amino)-3-(3-methoxyphenoxy)propan-2-ol (CHJ03017):**

A colorless oil, yield 83%, ^1^H-NMR (CD_3_OD, 400 MHz) δ (ppm): 7.13 (t, *J* = 8.0 Hz, 1H), 6.96 (s, 1H), 6.83 (m, 2H), 6.48 (m, 3H), 4.09 (m, 3H), 3.96 (dd, *J* = 9.60, 6.80 Hz, 1H), 3.85 (dd, *J* = 9.20, 6.0 Hz, 1H), 3.75 (d, *J* = 7.60 Hz, 6H), 3.52 (m, 2H), 3.03 (d, *J* = 11.60 Hz, 2H), 2.80 (t, *J* = 5.60 Hz, 2H), 2.61 (dd, *J* = 12.80, 5.60 Hz, 1H), 2.49 (dd, *J* = 12.80, 7.20 Hz, 2H), 2.30 (s, 3H), 2.16 (t, *J* = 11.60 Hz, 2H), 1.65 (dd, *J* = 12.40 Hz, 2H), 1.40 (s, 1H), 1.27 (m, 2H), 0.93 (d, *J* = 6.40 Hz, 3H). ^13^C-NMR (CD_3_OD, 100 MHz) δ (ppm): 160.97, 160.23, 149.65, 147.42, 131.90, 129.48, 121.46, 113.58, 113.05, 106.35, 106.02, 100.65, 70.42, 67.48, 66.69, 62.23, 59.13, 57.12, 54.98, 54.28, 53.99 (2C), 42.10, 33.46 (2C), 30.23, 20.76. IR (KBr, cm^−1^): 2947, 2925, 2872, 2837, 2792, 2360, 2340, 1593, 1559, 1513, 1492, 1455, 1418, 1368, 1334, 1287, 1264, 1231, 1201, 1154, 1083, 1036, 980, 940, 834, 807, 762, 687. HR-MS (ESI): *m*/*z* calcd for C_27_H_41_N_2_O_5_ [M + H]^+^: 473.3015. found: 473.2947. UPLC: t_R_ = 6.78 min; purity ≥ 98% (UV: 210 nm).

**1-(5-bromo-2-fluorophenoxy)-3-((3-methoxy-4-(2-(4-methylpiperidin-1-yl)ethoxy)benzyl)(methyl)amino)propan-2-ol (CHJ03018):**

A colorless oil, yield 85%, ^1^H-NMR (CD_3_OD, 400 MHz) δ (ppm): 7.23 (d, *J* = 7.20 Hz, 1H), 7.02 (m, 3H), 6.85 (dd, *J* = 22.0, 8.0 Hz, 2H), 4.09 (m, 4H), 3.94 (dd, *J* = 10.0, 5.60 Hz, 1H), 3.78 (s, 3H), 3.50 (m, 2H), 3.05 (d, *J* = 11. 60 Hz, 2H), 2.81 (t, *J* = 5.60 Hz, 2H), 2.63 (dd, *J* = 12.80, 5.60 Hz, 1H), 2.50 (dd, *J* = 12.85, 6.51 Hz, 1H), 2.31 (s, 3H), 2.18 (t, *J* = 12.0 Hz, 2H), 1.66 (d, *J* = 13.20 Hz, 2H), 1.41 (s, 1H), 1.28 (m, 2H), 0.94 (d, *J* = 6.0 Hz, 3H). ^13^C-NMR (CD_3_OD, 100 MHz) δ (ppm): 150.62, 149.66, 147.40, 131.94, 123.66, 121.44, 118.06, 117.16, 116.96, 115.94, 113.59, 112.97, 72.04, 67.36, 66.68, 62.24, 58.85, 57.10, 54.98, 53.98 (2C), 42.10, 33.44 (2C), 30.22, 20.72. IR (KBr, cm^−1^): 2947, 2925, 2872, 2845, 2794, 2360, 2340, 1607, 1511, 1459, 1417, 1404, 1369, 1323, 1303, 1262, 1231, 1138, 1117, 1090, 1020, 962, 935, 877, 837, 803, 755, 627. HR-MS (ESI): *m*/*z* calcd for C_26_H_37_BrFN_2_O_4_ [M + H]^+^: 539.1921. found: 539.1911. UPLC: t_R_ = 6.81 min; purity ≥ 98% (UV: 210 nm).

**1-(3-bromo-4-chlorophenoxy)-3-((3-methoxy-4-(2-(4-methylpiperidin-1-yl)ethoxy)benzyl)(methyl)amino)propan-2-ol (CHJ03043):**

A colorless oil, yield 89%, ^1^H-NMR (CDCl_3_, 400 MHz) δ (ppm): 7.30 (m, 1H), 7.17 (s, 1H), 6.81 (m, 4H), 4.11 (m, 3H), 3.91 (m, 2H), 3.84 (s, 3H), 3.61 (d, *J* = 12.0 Hz, 1H), 3.45 (d, *J* = 12.80 Hz, 1H), 2.98 (d, *J* = 11.20 Hz, 2H), 2.84 (t, *J* = 6.40 Hz, 2H), 2.61 (m, 1H), 2.47 (dd, *J* = 12.40, 3.60 Hz, 1H), 2.29 (s, 3H), 2.11 (t, *J* = 11.20 Hz, 2H), 1.63 (d, *J* = 12.40 Hz, 2H), 1.29 (m, 3H), 0.93 (d, *J* = 6.0 Hz, 3H). ^13^C-NMR (CDCl_3_, 100 MHz) δ (ppm): 157.74, 149.48, 147.66, 131.13, 130.47, 126.18, 122.53, 121.31, 119.57, 115.31, 113.07, 112.55, 71.00, 66.93, 65.99, 62.37, 59.10, 57.39, 55.99, 54.50 (2C), 42.29, 34.21 (2C), 30.56, 21.87. IR (KBr, cm^−1^): 2923, 2846, 2360, 2340, 1700, 1651, 1613, 1590, 1559, 1539, 1511, 1470, 1460, 1418, 1373, 1337, 1288, 1262, 1229, 1157, 1138, 1083, 1035, 931, 859, 805, 669. HR-MS (ESI): *m*/*z* calcd for C_26_H_37_BrClN_2_O_4_ [M + H]^+^: 555.1625. found: 555.1610. UPLC: t_R_ = 6.72 min; purity ≥ 98% (UV: 210 nm).

**1-(3-bromo-4-methylphenoxy)-3-((3-methoxy-4-(2-(4-methylpiperidin-1-yl)ethoxy)benzyl)(methyl)amino)propan-2-ol (CHJ04010):**

A colorless oil, yield 82%, ^1^H-NMR (CD_3_OD, 400 MHz) δ (ppm): 7.15 (d, *J* = 8.40 Hz, 1H), 7.09 (s, 1H), 6.96 (s, 1H), 6.82 (m, 3H), 4.11 (t, *J* = 5.60 Hz, 2H), 4.04 (m, 1H), 3.95 (m, 1H), 3.83 (m, 1H), 3.77 (s, 3H), 3.51 (q, *J* = 12.80 Hz, 2H), 3.03 (d, *J* = 11.60 Hz, 2H), 2.80 (t, *J* = 5.60 Hz, 2H), 2.60 (dd, *J* = 12.40, 5.60 Hz, 1H), 2.48 (dd, *J* = 13.20, 6.80 Hz, 1H), 2.30 (d, *J* = 2.40 Hz, 6H), 2.17 (t, *J* = 11.60 Hz, 2H), 1.65 (d, *J* = 12.40 Hz, 2H), 1.40 (s, 1H), 1.27 (m, 2H), 0.94 (d, *J* = 6.40 Hz, 3H). ^13^C-NMR (CD_3_OD, 100 MHz) δ (ppm): 157.77, 149.67, 147.44, 131.94, 130.79, 129.42, 124.18, 121.46, 117.97, 113.65, 113.61, 113.06, 70.79, 67.42, 66.75, 62.25, 59.98, 57.13, 55.00, 53.99 (2C), 42.14, 33.47 (2C), 30.24, 20.74, 20.50. IR (KBr, cm^−1^): 2946, 2923, 2871, 2792, 2360, 2340, 1651, 1604, 1579, 1539, 1511, 1492, 1458, 1418, 1368, 1323, 1289, 1262, 1236, 1158, 1138, 1086, 1030, 1003, 932, 866, 838, 806, 757, 671. HR-MS (ESI): *m*/*z* calcd for C_27_H_40_BrN_2_O_4_ [M + H]^+^: 535.2171. found: 535.2149. UPLC: t_R_ = 6.62 min; purity ≥ 98% (UV: 210 nm).

**1-(2-bromo-5-(trifluoromethyl)phenoxy)-3-((3-methoxy-4-(2-(4-methylpiperidin-1-yl)ethoxy)benzyl)(methyl)amino)propan-2-ol (CHJ04011):**

A colorless oil, yield 85%, ^1^H-NMR (CD_3_OD, 400 MHz) δ (ppm): 7.71 (d, *J* = 8.0 Hz, 1H), 7.27 (s, 1H), 7.16 (d, *J* = 8.40 Hz, 1H), 6.95 (s, 1H), 6.82 (q, *J* = 8.0 Hz, 3H), 4.09 (m, 5H), 3.74 (s, 3H), 3.53 (s, 2H), 3.04 (d, *J* = 11.20 Hz, 2H), 2.81 (t, *J* = 5.60 Hz, 2H), 2.72 (dd, *J* = 12.40, 4.80 Hz, 1H), 2.58 (dd, *J* = 12.80, 6.40 Hz, 1H), 2.33 (s, 3H), 2.18 (t, *J* = 12.0 Hz, 2H), 1.66 (d, *J* = 12.80 Hz, 2H), 1.40 (s, 1H), 1.29 (m, 2H), 0.94 (d, *J* = 6.0 Hz, 3H). ^13^C-NMR (CD_3_OD, 100 MHz) δ (ppm): 155.81, 149.65, 147.39, 133.69, 131.95, 121.42, 118.16, 118.12, 116.09, 113.57, 112.96, 109.59, 109.55, 71.58, 67.30, 66.67, 62.39, 58.82, 57.10, 54.91, 53.97 (2C), 42.13, 33.45 (2C), 30.22, 20.73. IR (KBr, cm^−1^): 3560, 3354, 2927, 2868, 2818, 1591, 1516, 1462, 1421, 1371, 1332, 1255, 1226, 1165, 1130, 1080, 1041, 1020, 935, 904, 862, 802, 752. HR-MS (ESI): *m*/*z* calcd for C_27_H_37_BrF_3_N_2_O_4_ [M + H]^+^: 589.1889. found: 589.1827. UPLC: t_R_ = 6.74 min; purity ≥ 98% (UV: 210 nm).

**1-(3,5-dichlorophenoxy)-3-((3-methoxy-4-(2-(4-methylpiperidin-1-yl)ethoxy)benzyl)(methyl)amino)propan-2-ol (CHJ04012):**

A colorless oil, yield 88%, ^1^H-NMR (CD_3_OD, 400 MHz) δ (ppm): 6.96 (d, *J* = 9.60 Hz, 2H), 6.84 (m, 4H), 4.10 (t, *J* = 5.60 Hz, 2H), 4.02 (m, 2H), 3.87 (m, 1H), 3.78 (s, 3H), 3.48 (m, 2H), 3.03 (d, *J* = 11.20 Hz, 2H), 2.80 (t, *J* = 6.40 Hz, 2H), 2.60 (dd, *J* = 12.80, 6.0 Hz, 1H), 2.46 (dd, *J* = 12.80, 6.40 Hz, 1H), 2.31 (s, 3H), 2.17 (t, *J* = 12.0 Hz, 2H), 1.65 (d, *J* = 12.75 Hz, 2H), 1.40 (s, 1H), 1.27 (m, 2H), 0.94 (d, *J* = 6.40 Hz, 3H). ^13^C-NMR (CD_3_OD, 100 MHz) δ (ppm): 160.34, 149.66, 147.46, 135.12, 131.94, 121.46, 120.38, 113.52, 113.42 (3C), 113.04, 70.04, 67.26, 66.70, 62.29, 58.68, 57.13, 54.99, 54.00 (2C), 42.25, 33.47 (2C), 30.24, 20.75. IR (KBr, cm^−1^): 2948, 2925, 2872, 2843, 2793, 2360, 2340, 1590, 1571, 1513, 1442, 1424, 1368, 1323, 1303, 1262, 1192, 1157, 1138, 1039, 980, 938, 853, 831, 800, 756, 670. HR-MS (ESI): *m*/*z* calcd for C_26_H_37_Cl_2_N_2_O_4_ [M + H]^+^: 511.2130. found: 511.2075. UPLC: t_R_ = 6.69 min; purity ≥ 98% (UV: 210 nm).

**1-(3-bromo-4-fluorophenoxy)-3-((3-methoxy-4-(2-(4-methylpiperidin-1-yl)ethoxy)benzyl)(methyl)amino)propan-2-ol (CHJ04020):**

A colorless oil, yield 90%, ^1^H-NMR (CD_3_OD, 400 MHz) δ (ppm): 7.10 (m, 2H), 6.95 (s, 1H), 6.84 (m, 3H), 4.10 (m, 3H), 3.96 (m, 1H), 3.84 (m, 1H), 3.77 (s, 3H), 3.50 (q, *J* = 12.0 Hz, 2H), 3.03 (d, *J* = 11.20 Hz, 2H), 2.80 (t, *J* = 5.20 Hz, 2H), 2.60 (dd, *J* = 12.80, 6.0 Hz, 1H), 2.47 (dd, *J* = 11.20, 6.80 Hz, 1H), 2.31 (s, 3H), 2.17 (t, *J* = 11.60 Hz, 2H), 1.65 (d, *J* = 12.40 Hz, 2H), 1.41 (s, 1H), 1.28 (m, 2H), 0.93 (d, *J* = 6.0 Hz, 3H). ^13^C-NMR (CD_3_OD, 100 MHz) δ (ppm): 155.71, 149.65, 147.44, 131.92, 121.47, 118.73, 116.32, 116.08, 114.95, 114.88, 113.55, 113.07, 71.29, 67.38, 66.71, 62.27, 58.86, 57.12, 55.01, 54.00 (2C), 42.21, 33.47 (2C), 30.23, 20.76. IR (KBr, cm^−1^): 2947, 2925, 2872, 2843, 2793, 2360, 2340, 1591, 1513, 1493, 1458, 1418, 1368, 1322, 1262, 1220, 1203, 1157, 1138, 1088, 1035, 979, 938, 862, 840, 806, 774. HR-MS (ESI): *m*/*z* calcd for C_26_H_37_BrFN_2_O_4_ [M + H]^+^: 539.1921. found: 539.1888. UPLC: t_R_ = 6.74 min; purity ≥ 98% (UV: 210 nm).

**1-((3-methoxy-4-(2-(4-methylpiperidin-1-yl)ethoxy)benzyl)(methyl)amino)-3-(2,4,6-tribromophenoxy)propan-2-ol (CHJ04022):**

A colorless oil, yield 90%, ^1^H-NMR (CD_3_OD, 400 MHz) δ (ppm): 7.75 (s, 2H), 6.98 (s, 1H), 6.85 (m, 2H), 4.10 (t, *J* = 5.69 Hz, 2H), 4.22 (m, 1H), 4.12 (t, *J* = 5.60 Hz, 2H), 3.79 (s, 3H), 3.54 (q, *J* = 12.80 Hz, 2H), 3.03 (d, *J* = 11.20 Hz, 2H), 2.80 (t, *J* = 5.60 Hz, 2H), 2.72 (dd, *J* = 13.20, 5.20 Hz, 1H), 2.54 (dd, *J* = 12.80, 7.20 Hz, 1H), 2.32 (s, 3H), 2.16 (t, *J* = 11.60 Hz, 2H), 1.65 (d, *J* = 12.40 Hz, 2H), 1.39 (s, 1H), 1.30 (m, 2H), 0.93 (d, *J* = 6.40 Hz, 3H). ^13^C-NMR (CD_3_OD, 100 MHz) δ (ppm): 152.75, 149.67, 147.45, 134.96 (3C), 131.75, 121.55, 118.54, 117.13, 113.66, 113.14, 75.83, 67.96, 66.75, 62.14, 59.34, 57.11, 55.06, 53.99 (2C), 42.08, 33.48 (2C), 30.24, 20.77. IR (KBr, cm^−1^): 2923, 2846, 2360, 2340, 1700, 1651, 1613, 1590, 1559, 1539, 1511, 1470, 1460, 1418, 1373, 1337, 1288, 1262, 1229, 1157, 1138, 1083, 1035, 931, 859, 805. HR-MS (ESI): *m*/*z* calcd for C_26_H_36_Br_3_N_2_O_4_ [M + H]^+^: 677.0225. found: 677.0256. UPLC: t_R_ = 6.76 min; purity ≥ 97% (UV: 210 nm).

**1-(3-bromo-5-fluorophenoxy)-3-((3-methoxy-4-(2-(4-methylpiperidin-1-yl)ethoxy)benzyl)(methyl)amino)propan-2-ol (CHJ04023):**

A colorless oil, yield 85%, ^1^H-NMR (CD_3_OD, 400 MHz) δ (ppm): 6.95 (s, 1H), 6.85 (m, 4H), 6.65 (d, *J* = 10.80 Hz, 1H), 4.11 (t, *J* = 5.60 Hz, 2H), 4.02 (m, 2H), 3.87 (m, 1H), 3.78 (s, 3H), 3.50 (q, *J* = 12.80 Hz, 2H), 3.03 (d, *J* = 11.20 Hz, 2H), 2.81 (t, *J* = 5.60 Hz, 2H), 2.60 (dd, *J* = 12.40, 5.60 Hz, 1H), 2.46 (dd, *J* = 12.80, 6.40 Hz, 1H), 2.31 (s, 3H), 2.17 (t, *J* = 12.0 Hz, 2H), 1.65 (d, *J* = 12.80 Hz, 2H), 1.40 (s, 1H), 1.29 (m, 2H), 0.93 (d, *J* = 6.0 Hz, 3H). ^13^C-NMR (CD_3_OD, 100 MHz) δ (ppm): 164.64, 162.18, 161.02, 149.65, 147.45, 131.93, 122.34, 121.46, 113.83, 113.29, 110.85, 101.17, 71.09, 67.25, 66.68, 62.28, 58.72, 57.12, 55.00, 53.99 (2C), 42.23, 33.46 (2C), 30.23, 20.76. IR (KBr, cm^−1^): 2947, 2925, 2872, 2841, 2792, 2360, 2340, 1605, 1583, 1512, 1454, 1418, 1367, 1318, 1280, 1263, 1231, 1280, 1263, 1231, 1146, 1084, 1039, 980, 941, 833. HR-MS (ESI): *m*/*z* calcd for C_26_H_37_BrFN_2_O_4_ [M + H]^+^: 539.1921. found: 539.1879. UPLC: t_R_ = 6.70 min; purity ≥ 97% (UV: 210 nm). UPLC: t_R_ = 6.70 min; purity ≥ 97% (UV: 210 nm).

**1-(3-chlorophenoxy)-3-((3-methoxy-4-(2-(4-methylpiperidin-1-yl)ethoxy)benzyl)(methyl)amino)propan-2-ol (CHJ04024):**

A colorless oil, yield 83%, ^1^H-NMR (CD_3_OD, 400 MHz) δ (ppm): 7.21 (t, *J* = 8.40 Hz, 1H), 6.88 (m, 6H), 4.09 (m, 3H), 3.98 (m, 1H), 3.86 (m, 1H), 3.77 (s, 3H), 3.51 (q, *J* = 12.80 Hz, 1H), 3.03 (d, *J* = 11.20 Hz, 1H), 2.80 (t, *J* = 5.60 Hz, 2H), 2.61 (dd, *J* = 12.80, 6.80 Hz, 2H), 2.49 (dd, *J* = 12.80, 6.80 Hz, 2H), 2.30 (s, 3H), 2.16 (t, *J* = 11.60 Hz, 2H), 1.65 (d, *J* = 12.80 Hz, 2H), 1.39 (s, 1H), 1.28 (m, 2H), 0.93 (d, *J* = 6.0 Hz, 3H). ^13^C-NMR (CD_3_OD, 100 MHz) δ (ppm): 159.91, 149.66, 147.45, 134.44, 131.93, 130.12, 121.47, 120.46, 114.57, 113.58, 113.06, 112.81, 70.71, 67.38, 66.72, 62.26, 58.98, 57.13, 55.00, 53.99 (2C), 42.16, 33.48 (2C), 30.24, 20.76. IR (KBr, cm^−1^): 2947, 2925, 2872, 2843, 2792, 2360, 2340, 1651, 1595, 1580, 1539, 1511, 1470, 1459, 1419, 1367, 1326, 1283, 1260, 1231, 1192, 1157, 1138, 1091, 1036, 979, 935, 870, 807, 770, 681. HR-MS (ESI): *m*/*z* calcd for C_26_H_38_ClN_2_O_4_ [M + H]^+^: 477.2520. found: 477.2476. UPLC: t_R_ = 6.81 min; purity ≥ 98% (UV: 210 nm).

**1-((3-methoxy-4-(2-(4-methylpiperidin-1-yl)ethoxy)benzyl)(methyl)amino)-3-(3-(trifluoromethyl)phenoxy)propan-2-ol (CHJ04025):**

A colorless oil, yield 83%, ^1^H-NMR (CD_3_OD, 400 MHz) δ (ppm): 7.44 (t, *J* = 8.0 Hz, 1H), 7.17 (m, 3H), 6.97 (s, 1H), 6.85 (m, 2H), 4.09 (m, 4H), 3.93 (m, 1H), 3.76 (s, 3H), 3.53 (m, 2H), 3.03 (d, *J* = 11.20 Hz, 2H), 2.80 (t, *J* = 5.60 Hz, 2H), 2.63 (dd, *J* = 12.40, 5.20 Hz, 1H), 2.51 (dd, *J* = 12.80, 6.80 Hz, 1H), 2.31 (s, 3H), 2.17 (t, *J* = 11.60 Hz, 2H), 1.65 (d, *J* = 12.80 Hz, 2H), 1.40 (s, 1H), 1.28 (m, 2H), 0.93 (d, *J* = 6.40 Hz, 3H). ^13^C-NMR (CD_3_OD, 100 MHz) δ (ppm): 159.32, 149.66, 147.45, 131.92, 131.39, 131.55, 130.01, 121.46, 117.96, 116.91, 113.57, 113.06, 110.96, 70.71, 67.38, 66.72, 62.26, 58.98, 57.13, 55.00, 53.99 (2C), 42.16, 33.48 (2C), 30.24, 20.76. IR (KBr, cm^−1^): 2947, 2926, 2873, 2845, 2794, 2360, 2340, 1651, 1593, 1557, 1539, 1513, 1493, 1453, 1419, 1367, 1330, 1289, 1262, 1234, 1165, 1096, 1065, 1037, 979, 934, 880, 794, 753, 698. HR-MS (ESI): *m*/*z* calcd for C_27_H_38_F_3_N_2_O_4_ [M + H]^+^: 511.2784. found: 511.2765. UPLC: t_R_ = 6.73 min; purity ≥ 96% (UV: 210 nm).

**1-(3,4-dichlorophenoxy)-3-((3-methoxy-4-(2-(4-methylpiperidin-1-yl)ethoxy)benzyl)(methyl)amino)propan-2-ol (CHJ04026):**

A colorless oil, yield 81%, ^1^H-NMR (CD_3_OD, 400 MHz) δ (ppm): 7.36 (d, *J* = 9.20 Hz, 1H), 7.05 (s, 1H), 6.95 (s, 1H), 6.84 (m, 3H), 4.10 (t, *J* = 5.60 Hz, 2H), 4.04 (m, 1H), 3.97 (m, 1H), 3.86 (m, 1H), 3.77 (s, 3H), 3.50 (q, *J* = 12.80 Hz, 1H), 3.03 (d, *J* = 11.60 Hz, 2H), 2.80 (t, *J* = 5.60 Hz, 2H), 2.61 (dd, *J* = 12.80, 6.0 Hz, 1H), 2.47 (dd, *J* = 12.80, 6.80 Hz, 1H), 2.31 (s, 3H), 2.16 (t, *J* = 11.60 Hz, 2H), 1.65 (d, *J* = 12.80 Hz, 2H), 1.40 (s, 1H), 1.27 (m, 2H), 0.94 (d, *J* = 6.40 Hz, 3H). ^13^C-NMR (CD_3_OD, 100 MHz) δ (ppm): 158.36, 149.64, 147.45, 132.25, 131.93, 130.48, 123.33, 121.47, 116.16, 114.57, 113.52, 113.07, 70.99, 67.33, 66.70, 62.29, 58.76, 57.13, 54.98, 54.00 (2C), 42.26, 33.48 (2C), 30.24, 20.76. IR (KBr, cm^−1^): 2947, 2926, 2872, 2841, 2792, 2360, 2340, 1651, 1592, 1570, 1539, 1511, 1475, 1455, 1419, 1367, 1286, 1262, 1230, 1191, 1156, 1127, 1092, 1036, 979, 937, 861, 807, 757, 670. HR-MS (ESI): *m*/*z* calcd for C_26_H_37_Cl_2_N_2_O_4_ [M + H]^+^: 511.2134. found: 511.2115. UPLC: t_R_ = 6.76 min; purity ≥ 99% (UV: 210 nm).

**1-(2-iodophenoxy)-3-((3-methoxy-4-(2-(4-methylpiperidin-1-yl)ethoxy)benzyl)(methyl)amino)propan-2-ol (CHJ04027):**

A colorless oil, yield 90%, ^1^H-NMR (CD_3_OD, 400 MHz) δ (ppm): 7.72 (d, *J* = 7.60 Hz, 1H), 7.30 (t, *J* = 8.0 Hz, 1H), 6.96 (s, 1H), 6.90 (d, *J* = 8.40 Hz, 1H), 6.83 (q, *J* = 8.0 Hz, 2H), 6.70 (t, *J* = 7.60 Hz, 1H), 4.09 (m, 3H), 3.98 (m, 2H), 3.74 (s, 3H), 3.55 (q, *J* = 12.80 Hz, 2H), 3.03 (d, *J* = 11.20 Hz, 2H), 2.80 (t, *J* = 5.60 Hz, 2H), 2.74 (d, *J* = 5.20 Hz, 1H), 2.64 (dd, *J* = 12.80, 7.60 Hz, 1H), 2.32 (s, 3H), 2.17 (t, *J* = 11.60 Hz, 2H), 1.65 (d, *J* = 12.80 Hz, 2H), 1.40 (s, 1H), 1.29 (m, 2H), 0.94 (d, *J* = 6.40 Hz, 3H). ^13^C-NMR (CD_3_OD, 100 MHz) δ (ppm): 157.53, 149.66, 147.40, 139.11, 131.94, 129.30, 122.32, 121.47, 113.60, 113.07, 112.12, 85.66, 71.22, 67.40, 62.42, 59.28, 57.11, 55.00, 53.97 (3C), 42.07, 33.47 (2C), 30.23, 20.75. IR (KBr, cm^−1^): 2946, 2923, 2871, 2844, 2792, 2360, 2340, 1584, 1513, 1471, 1441, 1418, 1368, 1323, 1261, 1231, 1192, 1158, 1138, 1084, 1050, 1030, 1019, 979, 962, 938, 873, 822, 806, 749. HR-MS (ESI): *m*/*z* calcd for C_26_H_38_IN_2_O_4_ [M + H]^+^: 569.1876. found: 569.1842. UPLC: t_R_ = 7.37 min; purity ≥ 98% (UV: 210 nm).

**1-(4-bromo-2,6-dichlorophenoxy)-3-((3-methoxy-4-(2-(4-methylpiperidin-1-yl)ethoxy)benzyl)(methyl)amino)propan-2-ol (CHJ04033):**

A colorless oil, yield 84%, ^1^H-NMR (CD_3_OD, 400 MHz) δ (ppm): 7.56 (s, 2H), 6.97 (s, 1H), 6.85 (m, 2H), 4.16 (m, 1H), 4.12 (t, *J* = 5.60 Hz, 2H), 3.99 (m, 2H), 3.79 (s, 3H), 3.52 (m, 2H), 3.04 (d, *J* = 11.20 Hz, 2H), 2.81 (t, *J* = 5.60 Hz, 2H), 2.67 (dd, *J* = 13.20, 4.80 Hz, 1H), 2.55 (dd, *J* = 13.20, 7.60 Hz, 1H), 2.30 (s, 3H), 2.17 (t, *J* = 12.0 Hz, 2H), 1.65 (d, *J* = 12.40 Hz, 2H), 1.40 (s, 1H), 1.29 (m, 2H), 0.94 (d, *J* = 6.0 Hz, 3H). ^13^C-NMR (CD_3_OD, 100 MHz) δ (ppm): 150.93, 149.67, 147.43, 131.79, 131.46 (3C), 129.99, 121.50, 116.13, 113.67, 113.08, 76.07, 67.97, 66.71, 62.14, 59.26, 57.09, 55.02, 53.97 (2C), 42.01, 33.44 (2C), 30.21, 20.76. IR (KBr, cm^−1^): 2947, 2924, 2872, 2840, 2794, 2360, 2340, 1544, 1511, 1459, 1419, 1375, 1320, 1259, 1231, 1193, 1158, 1138, 1084, 1031, 994, 933, 856, 803. HR-MS (ESI): *m*/*z* calcd for C_26_H_36_BrCl_2_N_2_O_4_ [M + H]^+^: 589.1236. found: 589.1220.

**1-(3-bromo-5-chlorophenoxy)-3-((3-methoxy-4-(2-(4-methylpiperidin-1-yl)ethoxy)benzyl)(methyl)amino)propan-2-ol (CHJ04034):**

A colorless oil, yield 85%, ^1^H-NMR (CD_3_OD, 400 MHz) δ (ppm): 7.12 (s, 1H), 7.02 (s, 1H), 6.95 (s, 1H), 6.91 (s, 1H), 6.83 (m, 2H), 4.10 (t, *J* = 5.60 Hz, 2H), 4.02 (m, 2H), 3.87 (m, 1H), 3.78 (s, 3H), 3.48 (m, 2H), 3.04 (d, *J* = 11.60 Hz, 2H), 2.81 (t, *J* = 5.60 Hz, 2H), 2.60 (dd, *J* = 12.40, 6.0 Hz, 1H), 2.46 (dd, *J* = 12.40, 6.0 Hz, 1H), 2.31 (s, 3H), 2.18 (t, *J* = 12.0 Hz, 2H), 1.66 (d, *J* = 12.80 Hz, 2H), 1.40 (s, 1H), 1.27 (m, 2H), 0.94 (d, *J* = 6.0 Hz, 3H). ^13^C-NMR (CD_3_OD, 100 MHz) δ (ppm): 160.39, 149.66, 147.44, 135.27, 131.85, 123.18, 122.49, 121.46, 116.33, 113.87, 113.54, 113.04, 71.04, 67.26, 66.67, 62.29, 58.67, 57.11, 55.01, 53.99 (2C), 42.26, 33.45 (2C), 30.22, 20.74. IR (KBr, cm^−1^): 2947, 2926, 2870, 2840, 2793, 2360, 2331, 1588, 1563, 1539, 1512, 1459, 1437, 1420, 1367, 1335, 1319, 1301, 1230, 1259, 1190, 1156, 1138, 1091, 1038, 978, 930, 912, 864, 831, 770, 670. HR-MS (ESI): *m*/*z* calcd for C_26_H_37_BrClN_2_O_4_ [M + H]^+^: 555.1625. found: 555.1600. UPLC: t_R_ = 6.63 min; purity ≥ 99% (UV: 210 nm).

**1-(2-bromo-5-fluorophenoxy)-3-((3-methoxy-4-(2-(4-methylpiperidin-1-yl)ethoxy)benzyl)(methyl)amino)propan-2-ol (CHJ04036):**

A colorless oil, yield 82%, ^1^H-NMR (CD_3_OD, 400 MHz) δ (ppm): 7.48 (m, 1H), 6.95 (s, 1H), 6.82 (m, 3H), 6.63 (t, *J* = 8.40 Hz, 1H), 4.11 (t, *J* = 5.6 Hz, 3H), 4.01 (m, 2H), 3.76 (s, 3H), 3.53 (s, 2H), 3.04 (d, *J* = 11.20 Hz, 2H), 2.81 (t, *J* = 5.60 Hz, 2H), 2.71 (dd, *J* = 12.80, 5.20 Hz, 1H), 2.58 (dd, *J* = 12.80, 7.20 Hz, 1H), 2.32 (s, 3H), 2.18 (t, *J* = 11.60 Hz, 2H), 1.66 (d, *J* = 12.80 Hz, 2H), 1.41 (s, 1H), 1.29 (m, 2H), 0.94 (d, *J* = 6.40 Hz, 3H). ^13^C-NMR (CD_3_OD, 100 MHz) δ (ppm): 164.01, 161.58, 156.35, 149.65, 147.40, 133.34, 131.93, 121.47, 113.59, 107.89, 106.05, 101.44, 71.52, 67.28, 66.65, 62.36, 58.95, 57.09, 54.96, 53.96 (2C), 42.01, 33.43 (2C), 30.21, 20.73. IR (KBr, cm^−1^): 3529, 3277, 3088, 2929, 2852, 2796, 2769, 2428, 1681, 1604, 1514, 1477, 1452, 1417, 1371, 1286, 1259, 1224, 1151, 1101, 1037, 960, 871, 833, 790, 748, 609, 451. HR-MS (ESI): *m*/*z* calcd for C_26_H_37_BrFN_2_O_4_ [M + H]^+^: 539.1921. found: 539.1914. UPLC: t_R_ = 6.70 min; purity ≥ 98% (UV: 210 nm).

***N*-(3-(2-hydroxy-3-((3-methoxy-4-(2-(4-methylpiperidin-1-yl)ethoxy)benzyl)(methyl)amino)propoxy)phenyl)acetamide (CHJ04058):**

A white solid, yield 90%, m. p: 60–62 °C, ^1^H-NMR (CD_3_OD, 400 MHz) δ (ppm): 7.27 (s, 1H), 7.17 (t, *J* = 8.0 Hz, 1H), 7.05 (m, 2H), 6.86 (m, 2H), 6.63 (d, *J* = 8.0 Hz, 1H), 4.11 (m, 3H), 3.97 (m, 1H), 3.87 (m, 1H), 3.78 (s, 3H), 3.55 (s, 2H), 3.13 (d, *J* = 11.20 Hz, 2H), 2.91 (t, *J* = 5.20 Hz, 1H), 2.65 (dd, *J* = 12.40, 6.80 Hz, 1H), 2.53 (dd, *J* = 12.40, 6.80 Hz, 1H), 2.32 (m, 5H), 2.11 (s, 3H), 1.70 (d, *J* = 13.20 Hz, 2H), 1.46 (s, 1H), 1.32 (m, 3H), 0.94 (d, *J* = 6.0 Hz, 3H). ^13^C-NMR (CD_3_OD, 100 MHz) δ (ppm): 170.23, 159.35, 149.61, 147.25, 139.66, 131.87, 129.11, 121.58, 113.73, 113.06, 112.11, 109.83, 106.29, 70.42, 67.37, 66.13, 62.12, 59.09, 56.86, 55.00, 53.79 (2C), 42.01, 33.02 (2C), 29.92, 22.53, 20.59. IR (KBr, cm^−1^): 2924, 2852, 2360, 2340, 1699, 1670, 1651, 1616, 1556, 1540, 1510, 1491, 1458, 1419, 1373, 1286, 1265, 1230, 1198, 1156, 1083, 1034, 980, 871, 768, 686, 669. HR-MS (ESI): *m*/*z* calcd for C_28_H_42_N_3_O_5_ [M + H]^+^: 500.3124. found: 500.3071. UPLC: t_R_ = 6.54 min; purity ≥ 99% (UV: 210 nm).

**1-(2,4-dichlorophenoxy)-3-((3-methoxy-4-(2-(4-methylpiperidin-1-yl)ethoxy)benzyl)(methyl)amino)propan-2-ol (CHJ04059):**

A colorless oil, yield 81%, ^1^H-NMR (CD_3_OD, 400 MHz) δ (ppm): 7.37 (s, 1H), 7.23 (d, *J* = 8.40 Hz, 1H), 7.00 (d, *J* = 8.80 Hz, 1H), 6.94 (s, 1H), 6.82 (q, *J* = 8.0 Hz, 2H), 4.11 (t, *J* = 5.60 Hz, 3H), 4.00 (m, 2H), 3.75 (s, 3H), 3.52 (s, 2H), 3.06 (d, *J* = 11.60 Hz, 2H), 2.82 (t, *J* = 5.60 Hz, 2H), 2.69 (dd, *J* = 12.80, 5.60 Hz, 1H), 2.54 (dd, *J* = 12.80, 6.80 Hz, 1H), 2.32 (s, 3H), 2.19 (t, *J* = 12.0 Hz, 2H), 1.67 (d, *J* = 12.80 Hz, 2H), 1.41 (s, 1H), 1.29 (m, 2H), 0.94 (d, *J* = 6.40 Hz, 3H). ^13^C-NMR (CD_3_OD, 100 MHz) δ (ppm): 153.47, 149.61, 147.37, 131.88, 129.29, 127.46, 125.32, 123.37, 121.45, 114.33, 113.50, 112.98, 71.53, 67.36, 66.58, 62.33, 58.86, 57.07, 54.92, 53.96 (2C), 42.19, 33.41 (2C), 30.20, 20.73. IR (KBr, cm^−1^): 2947, 2924, 2872, 2845, 2360, 2339, 1590, 1513, 1484, 1458, 1419, 1389, 1368, 1323, 1290, 1263, 1232, 1156, 1060, 1028, 1007, 938, 867, 846, 804, 745, 653. HR-MS (ESI): *m*/*z* calcd for C_26_H_37_Cl_2_N_2_O_4_ [M + H]^+^: 511.2130. found: 511.2120. UPLC: t_R_ = 6.67 min; purity ≥ 98% (UV: 210 nm).

***N*-(4-(2-hydroxy-3-((3-methoxy-4-(2-(4-methylpiperidin-1-yl)ethoxy)benzyl)(methyl)amino)propoxy)phenyl)acetamide (CHJ04061):**

A white solid, yield 90%, m. p: 60–62 °C, ^1^H-NMR (CD_3_OD, 400 MHz) δ (ppm): 7.41 (d, *J* = 8.0 Hz, 2H), 6.98 (s, 1H), 6.86 (m, 4H), 4.14 (m, 3H), 3.96 (m, 1H), 3.85 (m, 1H), 3.78 (s, 3H), 3.54 (d, *J* = 5.60 Hz, 2H), 3.14 (d, *J* = 11.20 Hz, 2H), 2.92 (t, *J* = 5.20 Hz, 1H), 2.64 (dd, *J* = 12.80, 7.20 Hz, 1H), 2.52 (dd, *J* = 12.80, 7.20 Hz, 1H), 2.32 (m, 5H), 2.09 (s, 3H), 1.70 (d, *J* = 13.20 Hz, 2H), 1.46 (s, 1H), 1.32 (m, 3H), 0.95 (d, *J* = 6.4 Hz, 3H). ^13^C-NMR (CD_3_OD, 100 MHz) δ (ppm): 170.23, 159.35, 149.61, 147.25, 139.66, 131.87, 129.11, 121.58, 113.73, 113.06, 112.11, 109.83, 106.29, 70.42, 67.37, 66.13, 62.12, 59.09, 56.86, 55.00, 53.79 (2C), 42.01, 33.02 (2C), 29.92, 22.53, 20.59. IR (KBr, cm^−1^): 2922, 2848, 2362, 2340, 2044, 1681, 1602, 1548, 1512, 1460, 1417, 1369, 1325, 1259, 1240, 1136, 1031, 931, 819, 750, 686, 669. HR-MS (ESI): *m*/*z* calcd for C_28_H_42_N_3_O_5_ [M + H]^+^: 500.3124. found: 500.3074. UPLC: t_R_ = 6.68 min; purity ≥ 99% (UV: 210 nm).

**1-((3-methoxy-4-(2-(4-methylpiperidin-1-yl)ethoxy)benzyl)(methyl)amino)-3-(2-(trifluoromethyl)phenoxy)propan-2-ol (CHJ04082):**

A colorless oil, yield 83%, ^1^H-NMR (CDCl_3_, 400 MHz) δ (ppm): 7.55 (d, *J* = 7.60 Hz, 1H), 7.47 (t, *J* = 8.0 Hz, 1H), 7.00 (t, *J* = 8.40 Hz, 2H), 6.81 (m, 3H), 4.10 (m, 5H), 3.84 (s, 3H), 3.62 (d, *J* = 12.80 Hz, 1H), 3.47 (d, *J* = 12.80 Hz, 1H), 3.00 (d, *J* = 10.80 Hz, 2H), 2.86 (t, *J* = 6.0 Hz, 2H), 2.68 (m, 1H), 2.59 (m, 1H), 2.30 (s, 3H), 2.15 (t, *J* = 10.80 Hz, 2H), 1.64 (d, *J* = 12.0 Hz, 2H), 1.31 (m, 3H), 0.93 (d, *J* = 5.60 Hz, 3H). ^13^C-NMR (CDCl_3_, 100 MHz) δ (ppm): 156.61, 149.49, 147.54, 133.28, 131.37, 127.08, 121.34 (2C), 120.33 (2C), 113.18, 112.96, 112.61, 70.86, 66.82, 66.17, 62.45, 59.40, 57.34, 55.95, 54.43 (2C), 42.43, 34.08 (2C), 30.50, 21.81. IR (KBr, cm^−1^): 2939, 2873, 2841, 2794, 2362, 1602, 1510, 1460, 1363, 1323, 1269, 1132, 1033, 974, 948, 879, 808, 758, 650. HR-MS (ESI): *m*/*z* calcd for C_27_H_38_F_3_N_2_O_4_ [M + H]^+^: 511.2784. found: 511.2741. UPLC: t_R_ = 6.66 min; purity ≥ 99% (UV: 210 nm).

**1-(4-chlorophenoxy)-3-((3-methoxy-4-(2-(4-methylpiperidin-1-yl)ethoxy)benzyl)(methyl)amino)propan-2-ol (CHJ04083):**

A colorless oil, yield 90%, ^1^H-NMR (CDCl_3_, 400 MHz) δ (ppm): 7.22 (d, *J* = 15.2 Hz, 2H), 6.81 (m, 5H), 4.16 (t, *J* = 6.0 Hz, 2H), 4.09 (m, 1H), 3.92 (d, *J* = 4.40 Hz, 2H), 3.84 (s, 3H), 3.62 (d, *J* = 13.20 Hz, 1H), 3.45 (d, *J* = 13.20 Hz, 1H), 2.99 (d, *J* = 10.8 Hz, 2H), 2.85 (t, *J* = 6.0 Hz, 2H), 2.63 (t, *J* = 11.60 Hz, 1H), 2.50 (m, 1H), 2.29 (s, 3H), 2.14 (t, *J* = 10.80 Hz, 2H), 1.64 (d, *J* = 12.40 Hz, 2H), 1.32 (m, 3H), 0.93 (d, *J* = 5.60 Hz, 3H). ^13^C-NMR (CDCl_3_, 100 MHz) δ (ppm): 157.41, 149.51, 147.62, 131.25, 129.30 (2C), 125.84, 121.32, 115.88 (2C), 113.16, 112.59, 70.74, 66.91, 66.11, 62.37, 59.29, 57.36, 55.99, 54.47 (2C), 42.28, 34.12 (2C), 30.52, 21.83. IR (KBr, cm^−1^): 2947, 2925, 2872, 2843, 2792, 2360, 2325, 1651, 1595, 1539, 1511, 1492, 1458, 1418, 1367, 1322, 1283, 1246, 1157, 1138, 1092, 1035, 1008, 824, 672. HR-MS (ESI): *m*/*z* calcd for C_26_H_38_ClN_2_O_4_ [M + H]^+^: 477.2520. found: 477.2471. UPLC: t_R_ = 6.74 min; purity ≥ 96% (UV: 210 nm).

**1-((3-methoxy-4-(2-(4-methylpiperidin-1-yl)ethoxy)benzyl)(methyl)amino)-3-(4-methoxyphenoxy)propan-2-ol (CHJ04084):**

A colorless oil, yield 90%, ^1^H-NMR (CDCl_3_, 400 MHz) δ (ppm): 6.82 (m, 7H), 4.17 (t, *J* = 6.0 Hz, 2H), 4.09 (m, 1H), 3.91 (d, *J* = 4.40 Hz, 2H), 3.84 (s, 3H), 3.76 (s, 3H), 3.62 (d, *J* = 12.80 Hz, 1H), 3.46 (d, *J* = 12.80 Hz, 1H), 3.01 (d, *J* = 12.4 Hz, 2H), 2.86 (t, *J* = 6.0 Hz, 2H), 2.63 (t, *J* = 11.20 Hz, 1H), 2.51 (m, 1H), 2.28 (s, 3H), 2.16 (t, *J* = 11.2 Hz, 2H), 1.65 (d, *J* = 12.0 Hz, 2H), 1.31 (m, 3H), 0.93 (d, *J* = 5.60 Hz, 3H). ^13^C-NMR (CDCl_3_, 100 MHz) δ (ppm): 154.01, 152.97, 149.49, 147.52, 131.40, 121.31, 115.55 (2C), 114.64 (2C), 113.17, 112.57, 71.16, 66.78, 66.29, 62.37, 59.53, 57.34, 55.98, 55.73, 54.43 (2C), 42.25, 34.04 (2C), 30.48, 21.80. IR (KBr, cm^−1^): 2947, 2925, 2871, 2834, 2792, 2360, 2325, 1595, 1510, 1459, 1418, 1368, 1322, 1262, 1231, 1156, 1138, 1036, 980, 937, 878, 824, 748. HR-MS (ESI): *m*/*z* calcd for C_27_H_41_N_2_O_5_ [M + H]^+^: 473.3015. found: 473.2975. UPLC: t_R_ = 6.74 min; purity ≥ 99% (UV: 210 nm).

**1-(2-chlorophenoxy)-3-((3-methoxy-4-(2-(4-methylpiperidin-1-yl)ethoxy)benzyl)(methyl)amino)propan-2-ol (CHJ04085):**

A colorless oil, yield 83%, ^1^H-NMR (CDCl_3_, 400 MHz) δ (ppm): 7.34 (d, *J* = 8.0 Hz, 1H), 7.19 (t, *J* = 7.60 Hz, 1H), 6.86 (m, 5H), 4.15 (t, *J* = 6.0 Hz, 3H), 4.03 (d, *J* = 4.40 Hz, 2H), 3.84 (s, 3H), 3.63 (d, *J* = 13.2 Hz, 1H), 3.47 (d, *J* = 13.2 Hz, 1H), 3.00 (d, *J* = 13.14 Hz, 2H), 2.85 (t, *J* = 6.40 Hz, 2H), 2.70 (m, 1H), 2.58 (m, 1H), 2.30 (s, 3H), 2.13 (t, *J* = 11.20 Hz, 2H), 1.64 (d, *J* = 12.0 Hz, 2H), 1.30 (m, 3H), 0.93 (d, *J* = 6.0 Hz, 3H). ^13^C-NMR (CDCl_3_, 100 MHz) δ (ppm): 154.31, 149.41, 147.52, 131.30, 130.25, 127.69, 123.15, 121.72, 121.34, 113.80, 112.97, 112.53, 71.46, 66.64, 66.24, 62.42, 59.34, 57.36, 55.95, 54.41 (2C), 42.40, 34.08 (2C), 30.58, 21.83. IR (KBr, cm^−1^): 2925, 2872, 2845, 2792, 2360, 2325, 1591, 1512, 1486, 1455, 1418, 1368, 1322, 1276, 1258, 1232, 1158, 1137, 1084, 1061, 980, 937, 877, 807, 749, 693. HR-MS (ESI): *m*/*z* calcd for C_26_H_38_ClN_2_O_4_ [M + H]^+^: 477.2520. found: 477.2506. UPLC: t_R_ = 6.74 min; purity ≥ 99% (UV: 210 nm).

**1-(3-bromo-4-methylphenoxy)-3-((3-methoxy-4-(2-(pyrrolidin-1-yl)ethoxy)benzyl)(methyl)amino)propan-2-ol (CHJ04064):**

A colorless oil, yield 80%, ^1^H-NMR (CD_3_OD, 400 MHz) δ (ppm): 7.15 (d, *J* = 8.40 Hz, 1H), 7.09 (s, 1H), 6.98 (s, 1H), 6.83 (m, 3H), 4.13 (t, *J* = 5.20 Hz, 2H), 4.05 (m, 1H), 3.96 (m, 1H), 3.82 (m, 4H), 3.54 (m, 2H), 3.04 (t, *J* = 5.20 Hz, 2H), 2.83 (s, 4H), 2.61 (dd, *J* = 12.40, 5.6 Hz, 1H), 2.49 (dd, *J* = 12.80, 6.80 Hz, 1H), 2.30 (d, *J* = 6.80 Hz, 6H), 1.87 (s, 4H). ^13^C-NMR (CD_3_OD, 100 MHz) δ (ppm): 157.76, 149.69, 147.25, 132.08, 130.80, 129.43, 124.18, 121.47, 117.94, 113.75, 113.64, 112.98, 70.76, 67.36, 67.27, 62.21, 58.95, 54.94, 54.50, 54.23 (2C), 42.08, 22.77 (2C), 20.50. IR (KBr, cm^−1^): 2924, 2873, 2850, 2793, 2360, 2339, 1604, 1513, 1492, 1459, 1418, 1369, 1325, 1263, 1235, 1139, 1031, 976, 928, 863, 805, 750, 669. HR-MS (ESI): *m*/*z* calcd for C_25_H_36_BrN_2_O_4_ [M + H]^+^: 507.1858. found: 507.1858. UPLC: t_R_ = 6.73min; purity ≥ 97% (UV: 210 nm).

**1-(2-bromo-5-(trifluoromethyl)phenoxy)-3-((3-methoxy-4-(2-(pyrrolidin-1-yl)ethoxy)benzyl)(methyl)amino)propan-2-ol (CHJ04065):**

A colorless oil, yield 82%, ^1^H-NMR (CD_3_OD, 400 MHz) δ (ppm): 7.71 (d, *J* = 8.0 Hz, 1H), 7.27 (s, 1H), 7.16 (d, *J* = 8.0 Hz, 1H), 6.97 (s, 1H), 6.84 (m, 2H), 4.10 (m, 5H), 3.75 (s, 3H), 3.55 (s, 2H), 3.07 (t, *J* = 5.20 Hz, 2H), 2.87 (s, 4H), 2.73 (dd, *J* = 12.80, 4.80 Hz, 1H), 2.60 (dd, *J* = 12.40, 6.40 Hz, 1H), 2.34 (s, 3H), 1.89 (s, 4H). ^13^C-NMR (CD_3_OD, 100 MHz) δ (ppm): 155.80, 149.68, 147.16, 133.70, 132.16, 130.42, 121.44, 118.14, 116.09, 113.78, 112.88, 109.57, 71.55, 67.09, 62.34, 58.80, 54.86, 54.45, 54.22 (2C), 42.08, 23.39, 22.76 (2C), 12.54. IR (KBr, cm^−1^): 2968, 2938, 2879, 2793, 2361, 2323, 1734, 1700, 1518, 1492, 1459, 1419, 1398, 1328, 1268, 1252, 1167, 1137, 1080, 1035, 977, 935, 906, 861, 748, 670. HR-MS (ESI): *m*/*z* calcd for C_25_H_33_BrF_3_N_2_O_4_ [M + H]^+^: 561.1576. found: 561.1569. UPLC: t_R_ = 6.81 min; purity ≥ 99% (UV: 210 nm).

**1-(3,5-dichlorophenoxy)-3-((3-methoxy-4-(2-(pyrrolidin-1-yl)ethoxy)benzyl)(methyl)amino)propan-2-ol (CHJ04066):**

A colorless oil, yield 84%, ^1^H-NMR (CD_3_OD, 400 MHz) δ (ppm): 6.98 (d, *J* = 3.60 Hz, 2H), 6.87 (m, 3H), 6.82 (d, *J* = 8.40 Hz, 1H), 4.13 (t, *J* = 4.80 Hz, 2H), 4.03 (m, 2H), 3.88 (m, 1H), 3.79 (s, 3H), 3.52 (q, *J* = 12.80 Hz, 2H), 3.06 (t, *J* = 5.20 Hz, 2H), 2.85 (s, 4H), 2.62 (dd, *J*= 12.40, 6.0 Hz, 1H), 2.47 (dd, *J* = 12.40, 6.40 Hz, 1H), 2.32 (s, 3H), 1.88 (s, 4H). ^13^C-NMR (CD_3_OD, 100 MHz) δ (ppm): 160.34, 149.67, 147.23, 135.12, 132.16, 121.46, 120.37, 113.68, 113.40 (3C), 112.94, 71.00, 67.21, 67.17, 62.25, 58.63, 54.92, 54.48, 54.24 (2C), 42.20, 22.76 (2C). IR (KBr, cm^−1^): 2933, 2877, 2843, 2787, 2361, 2340, 1591, 1514, 1454, 1421, 1330, 1290, 1261, 1230, 1165, 1130, 1089, 1028, 964, 908, 875, 808, 752, 692, 661, 617. HR-MS (ESI): *m*/*z* calcd for C_24_H_33_Cl_2_N_2_O_4_ [M + H]^+^: 483.1817. found: 483.1801. UPLC: t_R_ = 6.74 min; purity ≥ 98% (UV: 210 nm).

**1-((3-methoxy-4-(2-(pyrrolidin-1-yl)ethoxy)benzyl)(methyl)amino)-3-(2,4,6-tribromophenoxy)propan-2-ol (CHJ04068):**

A white solid, yield 84%, m. p: 61–63 °C, ^1^H-NMR (CD_3_OD, 400 MHz) δ (ppm): 7.76 (s, 2H), 6.99 (s, 1H), 6.86 (m, 2H), 4.23 (m, 1H), 4.11 (t, *J* = 5.20 Hz, 2H), 3.97 (s, 2H), 3.79 (s, 3H), 3.54 (q, *J* = 12.80 Hz, 2H), 2.94 (t, *J* = 5.60 Hz, 2H), 2.72 (s, 5H), 2.55 (dd, *J* = 12.80, 7.60 Hz, 1H), 2.32 (s, 3H), 1.83 (s, 4H). ^13^C-NMR (CD_3_OD, 100 MHz) δ (ppm): 152.74, 149.66, 147.42, 134.96 (2C), 134.96, 131.75, 121.53, 118.54, 117.14, 113.56, 113.07, 75.82, 67.94, 67.68, 62.12, 59.35, 55.01, 54.58, 54.23 (2C), 42.05, 22.83 (2C). IR (KBr, cm^−1^): 3103, 2924, 2873, 2808, 1695, 1597, 1514, 1435, 1371, 1334, 1253, 1138, 1031, 989, 852, 798, 734, 684, 570. HR-MS (ESI): *m*/*z* calcd for C_24_H_32_Br_3_N_2_O_4_ [M + H]^+^: 648.9912. found: 648.9938. UPLC: t_R_ = 6.64 min; purity ≥ 99% (UV: 210 nm).

**1-(3,4-dichlorophenoxy)-3-((3-methoxy-4-(2-(pyrrolidin-1-yl)ethoxy)benzyl)(methyl)amino)propan-2-ol (CHJ04072):**

A colorless oil, yield 85%, ^1^H-NMR (CD_3_OD, 400 MHz) δ (ppm): 7.37 (d, *J* = 8.80 Hz, 1H), 7.06 (s, 1H), 6.96 (s, 1H), 6.85 (m, 3H), 4.11 (t, *J* = 5.20 Hz, 2H), 4.04 (m, 1H), 3.98 (m, 1H), 3.86 (m, 1H), 3.77 (s, 3H), 3.51 (q, *J* = 12.80 Hz, 2H), 2.97 (t, *J* = 5.20 Hz, 2H), 2.76 (s, 4H), 2.61 (dd, *J* = 12.80, 6.0 Hz, 1H), 2.47 (dd, *J* = 12.80, 6.80 Hz, 1H), 2.32 (s, 3H), 1.85 (s, 4H). ^13^C-NMR (CD_3_OD, 100 MHz) δ (ppm): 158.36, 149.64, 147.37, 132.25, 131.99, 130.48, 123.32, 121.45, 116.13, 114.57, 113.50, 112.99, 70.96, 67.30, 62.27, 58.73, 54.92, 54.56, 54.24 (2C), 53.40, 42.22, 22.81 (2C). IR (KBr, cm^−1^): 2926, 2875, 2851, 2802, 2361, 2340, 1736, 1651, 1593, 1563, 1512, 1475, 1462, 1418, 1368, 1328, 1284, 1262, 1230, 1127, 1035, 976, 932, 902, 860, 805, 752, 671. HR-MS (ESI): *m*/*z* calcd for C_24_H_33_Cl_2_N_2_O_4_ [M + H]^+^: 483.1817. found: 483.1790. UPLC: t_R_ = 6.63 min; purity ≥ 98% (UV: 210 nm).

**1-((4-(2-(diethylamino)ethoxy)-3-methoxybenzyl)(methyl)amino)-3-(2-isopropylphenoxy)propan-2-ol (CHJ04089):**

A colorless oil, yield 82%, ^1^H-NMR (CDCl_3_, 400 MHz) δ (ppm): 7.20 (d, *J* = 7.60 Hz, 1H), 7.13 (t, *J* = 8.0 Hz, 1H), 6.93 (t, *J* = 7.20 Hz, 1H), 6.82 (m, 4H), 4.11 (m, 3H), 3.98 (m, 2H), 3.84 (s, 3H), 3.65 (d, *J* = 12.80 Hz, 1H), 3.46 (d, *J* = 12.80 Hz, 1H), 3.26 (m, 1H), 2.94 (t, *J* = 6.8 Hz, 2H), 2.66 (m, 5H), 2.54 (m, 1H), 2.31 (s, 3H), 1.20 (d, *J* = 7.20 Hz, 6H), 1.08 (t, *J* = 7.20 Hz, 6H). ^13^C-NMR (CDCl_3_, 100 MHz) δ (ppm): 155.82, 149.38, 147.59, 137.05, 131.20, 126.55, 126.07, 121.26, 120.89, 112.69, 112.41, 111.30, 70.35, 67.24, 66.34, 62.47, 59.66, 55.94, 51.66, 47.85 (2C), 42.36, 26.89, 22.63 (2C), 11.78 (2C). IR (KBr, cm^−1^): 3035, 2965, 2933, 2871, 2360, 1598, 1513, 1491, 1451, 1417, 1368, 1286, 1261, 1239, 1197, 1139, 1088, 1034, 983, 938, 881, 823, 751. HR-MS (ESI): *m*/*z* calcd for C_27_H_43_N_2_O_4_ [M + H]^+^: 459.3223. found: 459.3166. UPLC: t_R_ = 6.58 min; purity ≥ 99% (UV: 210 nm).

**1-(2-bromo-5-(trifluoromethyl)phenoxy)-3-((4-(2-(diethylamino)ethoxy)-3-methoxybenzyl)(methyl)amino)propan-2-ol (CHJ04090):**

A colorless oil, yield 81%, ^1^H-NMR (CDCl_3_, 400 MHz) δ (ppm): 7.63 (d, *J* = 8.40 Hz, 1H), 7.10 (m, 2H), 6.81 (m, 3H), 4.11 (m, 5H), 3.84 (s, 3H), 3.65 (d, *J* = 12.80 Hz, 1H), 3.47 (d, *J* = 13.20 Hz, 1H), 2.94 (t, *J* = 6.80 Hz, 2H), 2.67 (m, 5H), 2.55 (m, 1H), 2.32 (s, 3H), 1.08 (t, *J* = 7.20 Hz, 6H). ^13^C-NMR (CDCl_3_, 100 MHz) δ (ppm): 155.44, 149.39, 147.63, 133.70 (2C), 131.09, 121.31 (2C), 118.73, 116.43, 112.73, 112.45, 109.89, 71.63, 67.28, 66.10, 62.48, 58.95, 55.94, 51.65, 47.85 (2C), 42.42, 11.77 (2C). IR (KBr, cm^−1^): 2969, 2936, 2876, 2837, 2360, 2340, 1651, 1597, 1539, 1512, 1488, 1461, 1420, 1329, 1296, 1263, 1231, 1169, 1128, 1081, 1036, 979, 934, 857, 814, 751, 656, 565. HR-MS (ESI): *m*/*z* calcd for C_25_H_35_BrF_3_N_2_O_4_ [M + H]^+^: 563.1732. found: 563.1676. UPLC: t_R_ = 6.71 min; purity ≥ 98% (UV: 210 nm).

**1-(3,4-dichlorophenoxy)-3-((4-(2-(diethylamino)ethoxy)-3-methoxybenzyl)(methyl)amino)propan-2-ol (CHJ04091):**

A colorless oil, yield 85%, ^1^H-NMR (CDCl_3_, 400 MHz) δ (ppm): 7.31 (d, *J* = 8.8 Hz, 1H), 7.00 (s, 1H), 6.80 (m, 4H), 4.09 (m, 3H), 3.91 (m, 2H), 3.85 (s, 3H), 3.62 (d, *J* = 12.80 Hz, 1H), 3.45 (d, *J* = 12.80 Hz, 1H), 2.95 (t, *J* = 6.40 Hz, 2H), 2.67 (m, 5H), 2.47 (m, 1H), 2.29 (s, 3H), 1.08 (t, *J* = 7.20 Hz, 6H). ^13^C-NMR (CDCl_3_, 100 MHz) δ (ppm): 157.83, 149.37, 147.63, 132.81, 131.03, 130.65, 124.20, 121.31, 116.45, 114.63, 112.73, 112.46, 70.97, 67.20, 65.97, 62.37, 59.08, 55.96, 51.65, 47.80 (2C), 42.28, 11.70 (2C). IR (KBr, cm^−1^): 2970, 2936, 2874, 2832, 2360, 2326, 1700, 1651, 1593, 1559, 1539, 1511, 1475, 1459, 1418, 1337, 1285, 1263, 1230, 1158, 1126, 1090, 1126, 1035, 930, 865, 806, 671. HR-MS (ESI): *m*/*z* calcd for C_24_H_35_Cl_2_N_2_O_4_ [M + H]^+^: 485.1974. found: 485.1904. UPLC: t_R_ = 6.76 min; purity ≥ 98% (UV: 210 nm).

**1-(3-bromo-4-fluorophenoxy)-3-((4-(2-(diethylamino)ethoxy)-3-methoxybenzyl)(methyl)amino)propan-2-ol (CHJ04092):**

A colorless oil, yield 82%, ^1^H-NMR (CDCl_3_, 400 MHz) δ (ppm): 7.09 (m, 1H), 7.02 (t, *J* = 8.80 Hz, 1H), 6.81 (m, 4H), 4.09 (m, 3H), 3.90 (m, 2H), 3.85 (s, 3H), 3.62 (d, *J* = 12.80 Hz, 1H), 3.46 (d, *J* = 12.80 Hz, 1H), 2.95 (t, *J* = 6.40 Hz, 2H), 2.67 (m, 5H), 2.47 (m, 1H), 2.29 (s, 3H), 1.09 (t, *J* = 7.20 Hz, 6H). ^13^C-NMR (CDCl_3_, 100 MHz) δ (ppm): 155.25, 149.38, 147.62, 131.06, 121.3 (2C), 119.02 (2C), 116.52, 114.98, 112.58, 109.01, 71.29, 67.19, 66.04, 62.37, 59.14, 55.96, 51.64, 47.80 (2C), 42.27, 11.69 (2C). IR (KBr, cm^−1^): 2969, 2936, 2875, 2832, 2360, 2340, 1651, 1592, 1556, 1539, 1511, 1493, 1459, 1418, 1373, 1333, 1262, 1220, 1203, 1158, 1139, 1092, 1036, 929, 864, 805, 752. HR-MS (ESI): *m*/*z* calcd for C_24_H_35_BrFN_2_O_4_ [M + H]^+^: 513.1764. found: 513.1710. UPLC: t_R_ = 6.82 min; purity ≥ 98% (UV: 210 nm).

**1-(4-bromo-3-(trifluoromethyl)phenoxy)-3-((4-(2-(diethylamino)ethoxy)-3-methoxybenzyl)(methyl)amino)propan-2-ol (CHJ04093):**

A colorless oil, yield 83%, ^1^H-NMR (CDCl_3_, 400 MHz) δ (ppm): 7.57 (d, *J* = 8.80 Hz, 1H), 7.24 (s, 1H), 6.92 (d, *J* = 8.80 Hz, 1H), 6.82 (m, 3H), 4.11 (t, *J* = 6.40 Hz, 3H), 3.96 (m, 2H), 3.84 (s, 3H), 3.63 (d, *J* = 12.80 Hz, 1H), 3.46 (d, *J* = 12.80 Hz, 1H), 2.97 (t, *J* = 6.4 Hz, 2H), 2.67 (m, 5H), 2.48 (m, 1H), 2.30 (s, 3H), 1.10 (t, *J* = 7.2 Hz, 6H). ^13^C-NMR (CDCl_3_, 100 MHz) δ (ppm): 157.82, 149.40, 147.62, 135.72 (2C), 131.02, 121.32 (2C), 118.88 (2C), 114.64, 112.77, 110.18, 71.29, 67.19, 66.04, 62.37, 59.14, 55.96, 51.64, 47.80 (2C), 42.27, 11.69 (2C). IR (KBr, cm^−1^): 2969, 2936, 2875, 2800, 2360, 2340, 1603, 1512, 1475, 1462, 1419, 1373, 1330, 1313, 1260, 1233, 1171, 1139, 1097, 1036, 1017, 980, 932, 880, 810, 752, 702. HR-MS (ESI): *m*/*z* calcd for C_25_H_35_BrF_3_N_2_O_4_ [M + H]^+^: 563.1732. found: 563.1695. UPLC: t_R_ = 6.82 min; purity ≥ 98% (UV: 210 nm).

**1-(4-bromo-2,6-dichlorophenoxy)-3-((4-(2-(diethylamino)ethoxy)-3-methoxybenzyl)(methyl)amino)propan-2-ol (CHJ04094):**

A colorless oil, yield 87%, ^1^H-NMR (CDCl_3_, 400 MHz) δ (ppm): 7.44 (s, 2H), 6.81 (m, 3H), 4.06 (m, 5H), 3.84 (s, 3H), 3.61 (d, *J* = 12.80 Hz, 1H), 3.49 (d, *J* = 12.80 Hz, 1H), 2.96 (t, *J* = 6.80 Hz, 2H), 2.69 (m, 5H), 2.59 (m, 1H), 2.28 (s, 3H), 1.09 (t, *J* = 7.20 Hz, 6H). ^13^C-NMR (CDCl_3_, 100 MHz) δ (ppm): 150.74, 149.36, 147.52, 131.62 (3C), 131.24, 130.16, 121.30, 116.51, 112.76, 112.45, 71.29, 67.19, 66.04, 62.37, 59.14, 55.96, 51.64, 47.80 (2C), 42.27, 11.69 (2C). IR (KBr, cm^−1^): 2966, 2935, 2875, 2800, 2362, 2340, 1597, 1548, 1514, 1456, 1375, 1328, 1261, 1134, 1031, 995, 929, 854, 802, 748, 702, 569. HR-MS (ESI): *m*/*z* calcd for C_24_H_34_BrCl_2_N_2_O_4_ [M + H]^+^: 563.1079. found: 563.1039. UPLC: t_R_ = 6.82 min; purity ≥ 97% (UV: 210 nm).

**1-((4-(2-(1H-imidazol-1-yl)ethoxy)-3-methoxybenzyl)(methyl)amino)-3-(4-bromo-3-(trifluoromethyl)phenoxy)propan-2-ol (CHJ04097):**

A white solid, yield 85%, m. p: 110–112 °C, ^1^H-NMR (CDCl_3_, 400 MHz) δ (ppm): 7.64 (s, 1H), 7.56 (d, *J* = 8.8 Hz, 1H), 7.23 (s, 1H), 7.10 (s, 1H), 7.05 (s, 1H), 6.92 (d, *J* = 8.8 Hz, 1H), 6.84 (s, 1H), 6.74 (m, 2H), 4.34 (m, 2H), 4.24 (m, 2H), 4.10 (m, 1H), 3.96 (m, 2H), 3.84 (s, 3H), 3.62 (d, *J* = 12.80 Hz, 1H), 3.46 (d, *J* = 12.80 Hz, 1H), 2.62 (t, *J* = 11.20 Hz, 1H), 2.49 (m, 1H), 2.30 (s, 3H). ^13^C-NMR (CDCl_3_, 100 MHz) δ (ppm): 157.80, 149.88, 146.88, 137.64, 135.73, 132.45, 130.69, 129.26, 121.25, 119.59, 118.87, 114.63, 114.58, 114.18, 112.82, 110.21, 70.88, 68.92, 66.00, 62.35, 59.07, 55.95, 46.69, 42.35. IR (KBr, cm^−1^): 3111, 2929, 2877, 2787, 2362, 2340, 1600, 1514, 1473, 1419, 1321, 1261, 1230, 1170, 1138, 1093, 1026, 962, 908, 871, 810, 759, 663. HR-MS (ESI): *m*/*z* calcd for C_24_H_28_BrF_3_N_3_O_4_ [M + H]^+^: 558.1215. found: 558.1172. UPLC: t_R_ = 6.57 min; purity ≥ 99% (UV: 210 nm).

**1-((4-(2-(1H-imidazol-1-yl)ethoxy)-3-methoxybenzyl)(methyl)amino)-3-(2-bromo-5-(trifluoromethyl)phenoxy)propan-2-ol (CHJ04099):**

A white solid, yield 90%, m. p: 110–112 °C, ^1^H-NMR (CDCl_3_, 400 MHz) δ (ppm): 7.62 (d, *J* = 12.40 Hz, 2H), 7.08 (m, 4H), 6.85 (s, 1H), 6.78 (d, *J* = 8.0 Hz, 1H), 6.71 (d, *J* = 8.0 Hz, 1H), 4.34 (m, 2H), 4.23 (m, 2H), 4.09 (m, 3H), 3.83 (s, 3H), 3.64 (d, *J* = 12.80 Hz, 1H), 3.47 (d, *J* = 13.20 Hz, 1H), 2.73 (t, *J* = 11.60 Hz, 1H), 2.55 (m, 1H), 2.32 (s, 3H). ^13^C-NMR (CDCl_3_, 100 MHz) δ (ppm): 155.41, 149.90, 146.85, 137.67, 133.71, 132.57, 130.71, 129.39, 122.28, 121.25, 119.58, 118.81, 116.41, 114.19, 112.80, 109.89, 71.55, 68.94, 66.15, 62.45, 58.93, 55.94, 46.65, 42.52. IR (KBr, cm^−1^): 3118, 2879, 2845, 2787, 2362, 2340, 1676, 1595, 1516, 1460, 1421, 1388, 1332, 1290, 1259, 1138, 1114, 1085, 1029, 964, 906, 819, 752. HR-MS (ESI): *m*/*z* calcd for C_24_H_28_BrF_3_N_3_O_4_ [M + H]^+^: 558.1215. found: 558.1160. UPLC: t_R_ = 6.71 min; purity ≥ 98% (UV: 210 nm). UPLC: t_R_ = 6.55 min; purity ≥ 99% (UV: 210 nm).

**1-((4-(2-(1H-imidazol-1-yl)ethoxy)-3-methoxybenzyl)(methyl)amino)-3-(4-bromophenoxy)propan-2-ol (CHJ05001):**

A white solid, yield 85%, m. p: 110–112 °C, ^1^H-NMR (CDCl_3_, 400 MHz) δ (ppm): 7.63 (s, 1H), 7.35 (d, *J* = 8.4 Hz, 2H), 7.08 (d, *J* = 14.80 Hz, 2H), 6.78 (m, 5H), 4.34 (m, 2H), 4.23 (m, 2H), 4.09 (m, 1H), 3.92 (d, *J* = 4.40 Hz, 2H), 3.83 (s, 3H), 3.61 (d, *J* = 13.20 Hz, 1H), 3.45 (d, *J* = 12.80 Hz, 1H), 2.62 (t, *J* = 11.60 Hz, 1H), 2.49 (m, 1H), 2.29 (s, 3H). ^13^C-NMR (CDCl_3_, 100 MHz) δ (ppm): 157.89, 149.88, 146.84, 137.67, 132.56, 132.24 (2C), 129.38, 121.23, 119.58, 116.36 (2C), 114.19, 113.12, 112.81, 70.60, 68.95, 66.12, 62.34, 59.31, 55.96, 46.65, 42.34. IR (KBr, cm^−1^): 3111, 2931, 2879, 2843, 2771, 2362, 2340, 1712, 1587, 1514, 1487, 1456, 1419, 1355, 1325, 1242, 1139, 1099, 1064, 1028, 999, 960, 910, 883, 858, 819, 754, 692, 663, 615. HR-MS (ESI): *m*/*z* calcd for C_23_H_29_BrN_3_O_4_ [M + H]^+^: 490.1341. found: 490.1341. UPLC: t_R_ = 6.72 min; purity ≥ 98% (UV: 210 nm).

**1-((4-(2-(1H-imidazol-1-yl)ethoxy)-3-methoxybenzyl)(methyl)amino)-3-(3-bromo-4-methylphenoxy)propan-2-ol (CHJ05002):**

A white solid, yield 90%, m. p: 110–112 °C, ^1^H-NMR (CDCl_3_, 400 MHz) δ (ppm): 7.63 (s, 1H), 7.30 (d, *J* = 8.80 Hz, 1H), 7.16 (s, 1H), 7.08 (d, *J* = 16.0 Hz, 2H), 6.78 (m, 4H), 4.34 (m, 2H), 4.23 (m, 2H), 4.09 (m, 1H), 3.92 (m, 2H), 3.84 (s, 3H), 3.61 (d, *J* = 13.2 Hz, 1H), 3.45 (d, *J* = 13.2 Hz, 1H), 2.61 (t, *J* = 11.6 Hz, 1H), 2.48 (m, 1H), 2.29 (s, 3H). ^13^C-NMR (CDCl_3_, 100 MHz) δ (ppm): 157.73, 149.89, 146.87, 137.67, 132.50, 130.48, 129.37, 126.16, 122.52, 121.24, 119.55 (2C), 115.30, 114.19, 112.81, 70.74, 68.95, 66.04, 62.35, 59.12, 55.97, 46.66, 42.37. IR (KBr, cm^−1^): 3113, 2927, 2877, 2845, 2785, 2362, 2340, 1589, 1564, 1512, 1467, 1419, 1384, 1323, 1261, 1239, 1141, 1089, 1029, 960, 906, 858, 808, 754, 666. HR-MS (ESI): *m*/*z* calcd for C_23_H_28_BrClN_3_O_4_ [M + H]^+^: 524.0952. found: 524.0916. UPLC: t_R_ = 6.72 min; purity ≥ 99% (UV: 210 nm).

**1-((4-(2-(1H-imidazol-1-yl)ethoxy)-3-methoxybenzyl)(methyl)amino)-3-(3-bromo-4-methylphenoxy)propan-2-ol (CHJ05003):**

A white solid, yield 90%, m.p: 110–112 °C, ^1^H-NMR (CDCl_3_, 400 MHz) δ (ppm): 7.64 (s, 1H), 7.10 (s, 3H), 7.06 (s, 1H), 6.86 (s, 1H), 6.73 (m, 3H), 4.34 (m, 2H), 4.24 (m, 2H), 4.09 (m, 1H), 3.92 (m, 2H), 3.84 (s, 3H), 3.61 (d, *J* = 12.80 Hz, 1H), 3.46 (d, *J* = 13.20 Hz, 1H), 2.62 (t, *J* = 12.0 Hz, 1H), 2.48 (m, 1H), 2.30 (d, *J* = 10.4 Hz, 6H). ^13^C-NMR (CDCl_3_, 100 MHz) δ (ppm): 157.34, 149.86, 146.83, 137.67, 132.49, 130.99, 130.04, 129.37, 124.80, 121.24, 119.60, 118.30, 114.15, 113.90, 112.77, 70.68, 68.93, 66.11, 62.33, 59.30, 55.96, 46.64, 42.30, 21.84. IR (KBr, cm^−1^): 3112, 2924, 2877, 2844, 2777, 2361, 2340, 1605, 1564, 1511, 1492, 1454, 1418, 1264, 1239, 1226, 1158, 1141, 1091, 1029, 962, 896, 866, 815, 800, 764, 739, 658, 614. HR-MS (ESI): *m*/*z* calcd for C_24_H_31_BrN_3_O_4_ [M + H]^+^: 504.1498. found: 504.1460. UPLC: t_R_ = 6.71 min; purity ≥ 98% (UV: 210 nm).

### 4.5. The In Vitro Kinase Test

The SphKs assays were used the Kinase-Glo Plus Luminescent Kinase Assay Kit purchased from Promega by quantifying the amount of ATP remaining in the solution after the kinase reaction. The reaction volume is 50 μM. PF-543, purchased from Selleck, was used as a positive control. The compound was dissolved in pure DMSO to prepare 10 mM stock solutions and diluted with kinase buffer (pH = 7.4, composition: 40 mM/L Tris, 10 mM/L MgCl_2_, 0.1 g/L BSA, 1 mM/L DTT, 10 µM/L ATP). SphK1 was added to 96-well plates and treated with the appropriate concentration of compound (10µM) at 30 °C for 40 min. ATP test solution was added and the mixture was incubated at room temperature for 5 min. The luminescence was immediately measured using a microplate spectrophotometer (AD 340, Beckman, CA, USA). Graphpad Prism 9 software was used for data analysis.

### 4.6. 2.23-(4,5-Dimethylthiazol-2-yl)-2,5-Diphenytetrazolium Bromide (MTT) Assay

The effect of bufotalin on the viability of A375 cells was determined by an MTT assay. Three groups of cells at a volume of 200 µL/well (approximate density, 3 × 10^4^ cells/mL) were seeded into 96-well plates. After 24 h of incubation, different concentrations of **CHJ04022R** were added, and the cells were further cultured for 48 h. Then, 20 µL of MTT solution (working concentration, 5 mg/mL; Sigma-Aldrich, St. Louis, MO, USA) was added into each well, and the cells were incubated for an additional 4 h. Removing supernatant with filter paper and retaining crystallization. The resulting formazan crystals were dissolved using 150 µL dimethyl sulfoxide (DMSO). Absorbance was measured at a wavelength of 490 nM using a microplate reader. The absorbance of the cells treated with DMEM medium containing 0.2% DMSO was regarded as the vehicle group (survival rate 100%). The absorbance of cells treated with DMEM medium containing cisplatin was the positive control group.

### 4.7. Wound Healing Assay

A375 cells (1 × 10^6^ cells/mL) were seeded in 6-well plates for 24 h. After reaching confluency, a sterile yellow micropipette tip was used to create wounds in each plate. The unscraped cells in the plate were washed with PBS three times, dead cells were removed and fresh MEM medium supplemented with FBS containing different concentrations of **CHJ04022R** (0, 0.04, 0.2 and 1 μM, respectively) were added for 24 h. The cells were treated with fresh MEM medium supplemented with FBS containing 0.02‰ DMSO was regarded as the vehicle group. At the end of the incubation time, the wound healing area in each well was photographed with an inverted microscope. The rate of wound closure was also compared at the indicated time.

### 4.8. Transwell ASSAY

The cell migration assay was used to measure cell migration of A375 cells in vitro. For cell migration, transwell cell culture chambers (8 mm pore size) without coated matrigel were used. A375 cells (1 × 10^6^ cells/mL) with serum-free MEM medium were added into the upper chamber, together with various concentrations of **CHJ04022R** (0, 0.04, 0.2 and 1 μM). The cells were treated with serum-free MEM medium containing 0.02‰ DMSO was regarded as the vehicle group. The 90% MEM medium containing 10% FBS was added to the lower chamber as chemoattractant. After 24 h or 48 h incubation at 37 °C, the chambers were rinsed with PBS. The cells that migrated to the lower surface of the filters were fixed with 4% formaldehyde in PBS for 20 min. Then, the chambers were rinsed with PBS and cells were stained with crystal violet for 15 min. The chambers were rinsed with PBS and non-migrating cells were removed from the top chamber with a cotton swab. Then, five fields of vision were selected and photographed under a 10× objective lens.

### 4.9. Cell Cycle Analysis

Cell cycle distribution was analyzed by flow cytometry. Cells (4 mL) were inoculated in a petri dish (6 × 10^5^ cells/plate). The cells were cultured for 24 h, and the medium was replaced with 4 mL complete medium containing **CHJ04022R** (0, 0.04, 0.2 and 1 μM) for A375 cells. The Cisplatin (6 μM) group was used as the positive control group. At 48 h after incubation, 1 × 10^6^ cells were harvested by typsinization, rinsed with phosphate-buffered saline (PBS) and fixed with cold 70% ethanol at 4 °C overnight. The cells were centrifuged at 1500 r/min for 5 min. The cells were incubation in 0.5 mL of staining solution (PI/RNase; 550825; BD Biosciences, Piscataway, NJ, USA), and incubated at 37 °C for 15 min. The percentage of cells at each stage of the cell cycle was analyzed by flow cytometry.

### 4.10. Apoptosis Detection by Annexin V-FITC/PI Staining

Cells (4 mL) were inoculated in a petri dish (6 × 10^5^cells/plate). The cells were cultured for 24 h, and the medium was replaced with 4 mL complete medium containing **CHJ04022R** (0, 0.04, 0.2 and 1 μM) for A375 cells. Add 0.02‰ DMSO group as solvent control. The cells were incubated for 48 h, trypsinized and centrifuged at 1000× *g* for 5 min. The cells were resuspended in pre-cooled PBS and centrifuged again at 1000× *g* for 5 min. Annexin V-FITC and PI were added, the solutions were mixed well and the samples were incubated for 15 min in the dark at room temperature. Then, the solutions were kept at 4 °C. Cell fluorescence was detected by flow cytometry.

### 4.11. Western Blot Assay

A375 cells were cultured in a 10 cm dish with 1 × 10^6^ cells/mL, and the cell density reached about 60% after incubation for 24 h. The cells were washed with PBS and incubated with **CHJ04022R** (0, 0.04, 0.2 and 1 μM) at different concentrations for 48 h. Next, 0.02‰ DMSO was added as solvent control. The cells were collected on ice and centrifuged at 5000× *g* at 4 °C for 5 min. We quantitated the total protein concentration of the supernatants by using a Bradford protein assay kit. Proteins (30 μg per channel) were isolated with 10% SDS-PAGE and transferred to PVDF membrane. The membrane of total protein was blocked with 5% non-fat milk which was in the TBST buffer and the phosphorylated protein was blocked with 5% FBS, which was in the TBST buffer for 1 h at room temperature. The membranes were washed with the TBST buffer and then incubated with primary antibodies at 4 °C overnight. The primary antibodies used in the study were as follows: beta-actin, PI3K, P-PI3K, AKT, NF-κB, MMP-2 and MMP-9 (1:1000). After washing three times with TBST buffer, the membrane was then incubated with antirabbit IgG (1:10,000) at room temperature for 1 h. The membrane was washed three times with TBST buffer. The blots were developed using the azure c400. The bands of target protein were subjected to densitometric analysis using Image-Pro Plus 6.0 software.

### 4.12. The Xenograft Tumor Animal Model

Typically, a diluted A375 cells suspension (0.2 mL, 1 × 10^7^ cells/mouse) was injected subcutaneously into the right arm pit of the nude BALB/c mice at day 0. Mice were randomly divided into six groups (*n* = 10) and given orally with **CHJ04022R** (0, 2.5, 5 and 10 mg·kg^−1^·day^−1^) when the tumor searched about 100 mm^3^. The formulation that was used to administer **CHJ04022R** as follows: V_Nacl_ = **CHJ04022R** stock concentration/**CHJ04022R** concentration. Cisplatin (2 mg·kg^−1^·day^−1^) was given orally to mice in single dose as a positive control group. The body weight and tumor size of each mouse was determined twice a week. The solid tumor volume (V) was determined by measuring the longest diameter (A) and shortest diameter (B) of the tumor using digital vernier caliper measurements and calculated as follows: V = (A × B^2^)/2. After sacrificing the mice on day 14, the tumors and normal tissues were harvested for molecular assessment.

### 4.13. Molecular Docking

The protein crystal structure of SphK1 was downloaded from the RCSB Protein Data Bank (PDB code: 3vzb, resolution: 2 Å). The binding site of the original ligand Sph is defined as the active site for docking. Docking of receptors and ligands was performed using LibDock program in Discovery Studio (BIOVIA, Discovery Studio 2020). The conformation generation method of ligand is set to “Best”. Other parameters were set to their default values. Finally, the docking results were sorted according to LibDock score.

## Figures and Tables

**Table 1:** The structures of intermediate compounds (**5**).

Code	Structure	Code	Structure
**5-1**	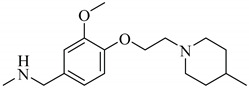	5-2	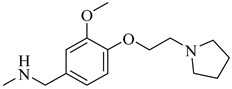
**5-3**	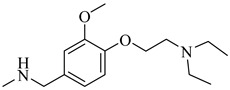	5-4	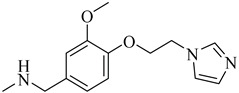

**Table 2:** Percent inhibition of CHJ series compounds (10 μM) on SphK1.

Code	Structure	SphK1 (%Inhibition)	Code	Structure	SphK1 (%Inhibition)
**SAMS10** **(lead comd)**	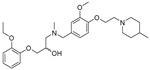	60	**CHJ** **04036**	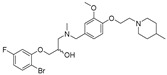	12
**CHJ** **02029**	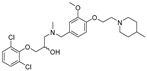	1	**CHJ** **04058**	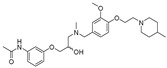	27
**CHJ** **02049**	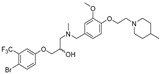	4	**CHJ** **04059**	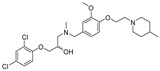	3
**CHJ** **02050**	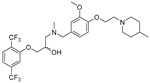	0	**CHJ** **04060**	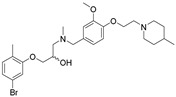	28
**CHJ** **03001**	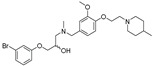	0	**CHJ** **04061**	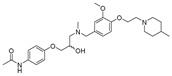	19
**CHJ** **03003**	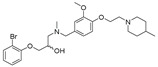	4	**CHJ** **04064**	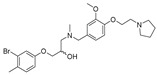	7
**CHJ** **03004**	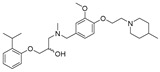	4	**CHJ** **04065**	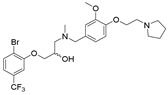	0
**CHJ** **03005**	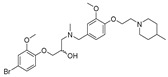	10	**CHJ** **04066**	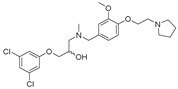	0
**CHJ** **03011**	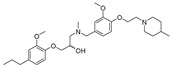	21	**CHJ** **04068**	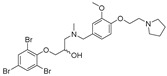	16
**CHJ** **03012**	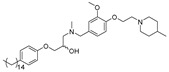	19	**CHJ** **04072**	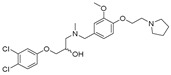	20
**CHJ** **03014**	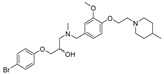	20	**CHJ** **04082**	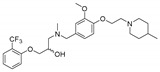	32
**CHJ** **03015**	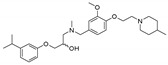	4	**CHJ** **04083**	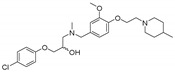	32
**CHJ** **03017**	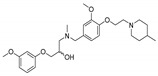	0	**CHJ** **04084**	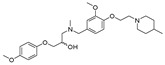	24
**CHJ** **03018**	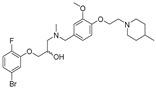	0	**CHJ** **04085**	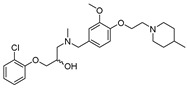	11
**CHJ** **03043**	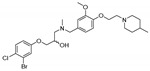	0	**CHJ** **04086**	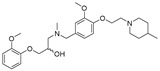	7
**CHJ** **04010**	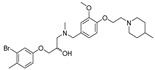	0	**CHJ** **04089**	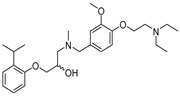	17
**CHJ** **04011**	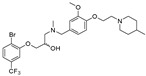	5	**CHJ** **04090**	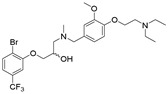	2
**CHJ** **04012**	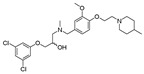	2	**CHJ** **04091**	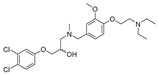	7
**CHJ** **04020**	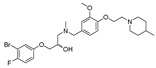	24	**CHJ** **04092**	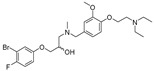	16
**CHJ** **04022**	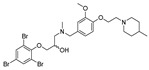	30	**CHJ** **04093**	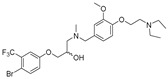	11
**CHJ** **04023**	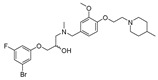	20	**CHJ** **04094**	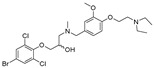	0
**CHJ** **04024**	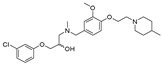	18	**CHJ** **04097**	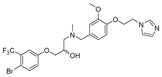	6
**CHJ** **04025**	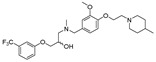	10	**CHJ** **04099**	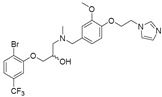	16
**CHJ** **04026**	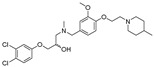	4	**CHJ** **05001**	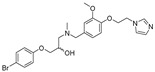	23
**CHJ** **04027**	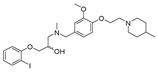	11	**CHJ** **05002**	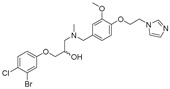	26
**CHJ** **04033**	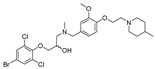	3	**CHJ** **05003**	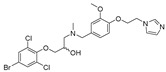	17
**CHJ** **04034**	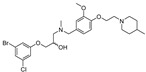	11	**PF-543** ^a^	-	49 ± 2.1 n M

^a^ PF-543 is a positive control. IC_50_ values were calculated from three independent experiments.

**Table 3:** In vitro inhibitory activities of CHJ series compounds against the selected cancer cells.

Code	IC_50_ (μM) ^a^			
	A549	SKOV3	A375	LOVO
**SAMS10**	14.64 ± 0.06	21.07 ± 0.18	27.11 ± 0.66	11.48 ± 0.07
**CHJ02029**	3.55 ± 0.06	6.30 ± 0.16	2.43 ± 0.12	1.87 ± 0.02
**CHJ02049**	0.52 ± 0.23	0.62 ± 0.08	0.62 ± 0.04	1.25 ± 0.03
**CHJ02050**	1.09 ± 0.55	4.04 ± 0.06	1.87 ± 0.07	3.88 ± 1.80
**CHJ03001**	1.30 ± 0.43	2.48 ± 0.63	1.95 ± 0.33	1.26 ± 0.53
**CHJ03003**	6.08 ± 0.90	3.00 ± 0.99	5.95 ± 0.42	0.70 ± 0.26
**CHJ03004**	2.10 ± 1.25	6.19 ± 0.25	0.72 ± 0.80	2.54 ± 0.10
**CHJ03005**	15.58 ± 0.16	3.99 ± 0.21	5.60 ± 0.81	6.66 ± 0.11
**CHJ03011**	10.56 ± 0.02	21.21 ± 1.08	1.82 ± 1.23	15.19 ± 1.78
**CHJ03012**	2.19 ± 1.04	3.54 ± 1.02	0.16 ± 0.33	1.95 ± 0.43
**CHJ03014**	1.09 ± 0.06	6.19 ± 0.62	1.63 ± 0.72	0.53 ± 0.15
**CHJ03015**	1.03 ± 0.13	4.47 ± 1.62	3.22 ± 0.42	0.55 ± 0.21
**CHJ03017**	17.25 ± 0.79	6.21 ± 1.04	8.68 ± 0.11	2.44 ± 0.03
**CHJ03018**	18.76 ± 1.06	1.79 ± 0.23	3.13 ± 0.06	1.72 ± 0.58
**CHJ03043**	2.50 ± 1.32	2.90 ± 0.19	0.54 ± 0.26	0.95 ± 0.41
**CHJ04010**	4.51 ± 1.29	4.96 ± 1.26	0.45 ± 0.46	2.78 ± 0.23
**CHJ04011**	4.05 ± 1.12	2.41 ± 1.03	0.48 ± 0.28	1.01 ± 0.08
**CHJ04012**	6.38 ± 0.07	4.34 ± 0.32	1.24 ± 1.01	2.12 ± 0.04
**CHJ04020**	1.38 ± 1.14	3.40 ± 1.17	1.60 ± 0.92	3.83 ± 1.80
**CHJ04022**	1.88 ± 0.19	1.03 ± 0.24	0.05 ± 0.01	0.69 ± 0.03
**CHJ04023**	5.44 ± 2.54	8.03 ± 2.77	0.81 ± 0.33	3.07 ± 1.62
**CHJ04024**	5.45 ± 1.03	1.37 ± 0.34	0.98 ± 0.76	18.61 ± 3.22
**CHJ04025**	4.21 ± 2.24	3.32 ± 1.02	2.34 ± 1.63	2.55 ± 1.65
**CHJ04026**	0.97 ± 0.21	7.45 ± 2.26	1.50 ± 0.06	0.82 ± 0.42
**CHJ04027**	6.22 ± 1.34	3.02 ± 1.76	0.73 ± 0.13	2.35 ± 1.04
**CHJ04033**	0.95 ± 0.37	6.22 ± 3.05	0.92 ± 0.07	2.23 ± 1.26
**CHJ04034**	0.86 ± 0.45	3.06 ± 1.10	0.65 ± 0.14	1.16 ± 0.94
**CHJ04036**	17.84 ± 3.62	15.5 ± 2.56	18.36 ± 1.10	3.67 ± 0.41
**CHJ04058**	245.31 ± 2.40	101.71 ± 1.48	56.21 ± 0.55	53.96 ± 2.14
**CHJ04059**	10.63 ± 3.37	3.26 ± 1.21	3.23 ± 0.49	3.92 ± 0.56
**CHJ04061**	333.10 ± 2.7	143.47 ± 3.1	62.13 ± 4.21	97.14 ± 4.21
**CHJ04064**	21.50 ± 3.65	17.05 ± 0.24	9.24 ± 2.65	3.55 ± 2.14
**CHJ04065**	25.48 ± 2.11	27.07 ± 1.62	10.82 ± 1.79	3.14 ± 1.06
**CHJ04066**	29.90 ± 2.45	77.07 ± 4.07	10.29 ± 1.40	2.50 ± 0.65
**CHJ04068**	5.46 ± 1.76	4.21 ± 1.23	1.54 ± 1.34	0.52 ± 0.40
**CHJ04072**	12.53 ± 2.44	9.62 ± 4.23	2.26 ± 0.37	5.21 ± 2.31
**CHJ04082**	22.26 ± 1.98	28.54 ± 5.22	7.51 ± 2.62	2.82 ± 1.05
**CHJ04083**	13.35 ± 2.02	10.36 ± 1.43	2.44 ± 1.03	1.23 ± 0.17
**CHJ04084**	42.69 ± 3.06	96.38 ± 2.72	16.70 ± 2.48	7.54 ± 1.02
**CHJ04085**	28.83 ± 5.12	49.22 ± 1.97	7.32 ± 1.80	1.94 ± 0.79
**CHJ04089**	27.78 ± 1.73	42.71 ± 2.61	15.03 ± 2.37	49.50 ± 3.54
**CHJ04090**	35.01 ± 2.21	36.4 ± 2.17	11.70 ± 2.12	34.05 ± 2.59
**CHJ04091**	11.05 ± 1.33	17.8 ± 3.11	4.01 ± 3.11	10.63 ± 3.20
**CHJ04092**	30.16 ± 2.76	34.5 ± 2.70	11.23 ± 1.05	47.91 ± 3.43
**CHJ04093**	20.12 ± 2.01	10.24 ± 1.69	2.60 ± 1.22	14.45 ± 2.14
**CHJ04094**	18.40 ± 3.17	15.86 ± 2.11	2.05 ± 1.01	28.51 ± 4.42
**CHJ04097**	24.38 ± 2.78	33.59 ± 2.47	14.85 ± 2.77	32.10 ± 3.39
**CHJ04099**	23.10 ± 2.58	37.39 ± 2.48	16.16 ± 2.11	38.24 ± 2.94
**CHJ05001**	30.69 ± 3.12	40.12 ± 3.02	15.78 ± 1.73	49.10 ± 2.71
**CHJ05002**	35.66 ± 2.71	42.96 ± 2.37	13.66 ± 2.06	33.10 ± 3.27
**CHJ05003**	33.97 ± 2.11	35.11 ± 1.35	24.92 ± 1.93	52.49 ± 1.22
**Cisplatin**	4.21 ± 1.31	1.91 ± 0.43	5.91 ± 1.64	2.64 ± 1.12

^a^ IC_50_ values were calculated from three independent experiments using the MTT assay. The values are reported as the mean ± SD.

**Table 4:** In vitro inhibitory activity of CHJ04022R and CHJ04022S against A375.

Compound	Structure	IC_50_ (μM)
**CHJ04022R**	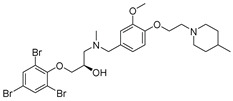	2.95 ± 0.23
**CHJ04022S**	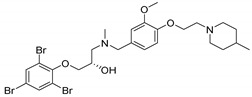	3.98 ± 0.33
**Cisplatin**	-	3.75 ± 0.62

**Table 5:** Tumor weight of mice and inhibition rate.

Group	Dose (mg/Kg)	Tumor Weight (g, Mean ± SD)	Inhibition Rate (%)	*p* Value
**Control**	-	1.30 ± 0.28		-
**DMSO**	-	1.38 ± 0.18		-
**CHJ04022R**	2.5	1.23 ± 0.18	10.84	-
	5	0.88 ± 0.41	36.14	<0.05
	10	0.78 ± 0.22	49.40	<0.05
**Cisplatin**	2	0.53 ± 0.12	61.45	<0.0001

## Data Availability

The data presented in this study are available in Supplementary Material here.

## Supplementary Materials

The following supporting information can be downloaded at: https://www.mdpi.com/article/10.3390/molecules27062020/s1, Table S1: The structures of phenoxy-2,3-epoxypropane compounds (**6**).
