# Supplementary material for: Design, Synthesis and Biological Activity Testing of Library of Sphk1 Inhibitors
Source: Molecules. 2022 Mar 21;27(6):2020. doi: 10.3390/molecules27062020 (PMC8951126; doi:10.3390/molecules27062020)
Supplement: Supplementary file 1 [file molecules-27-02020-s001.zip › molecules-1633392-supplementary.pdf]

Design, Synthesis and Biological Activity Testing of Library of Sphk1 Inhibitors

Shuangshuang Geng[a#], Haijiao Chen[a#], Yan Li[a], Ying Li[a], Jingxiang Pang[b], Feipeng Zhang[a], Zhiqiang Qu[a], Mengjun Li[a], Na Liu[a], Qingqiang Yao[a], Yanling Mu[a*], and Bo Liu[a*]

[a] Institute of Materia Medica, Shandong First Medical University & Shandong Academy of Medical Sciences, Jinan 250117, Shandong, P. R. China

[b] Biomedical Sciences College & Shandong Medicinal Biotechnology Centre, Shandong First Medical University & Shandong Academy of Medical Sciences, Jinan 250117, Shandong, P. R. China

[*]Email: muyanling@sdfmu.edu.cn;

[*]Email: mls_liub@ujn.edu.cn

#S.G. and H.C. contributed equally to this paper.

Table of Contents

1. The structures of phenoxy-2,3-epoxypropane compounds (**6**)

2. Copies of NMR (^{1}H and ^{13}C) spectra for Compounds **5** and **CHJ** series compounds.

1. The structures of phenoxy-2,3-epoxypropane compounds (6)

Table S1. The structures of phenoxy-2,3-epoxypropane compounds (6)

Code	Structure	Code	Structure	Code	Structure
**6-1**	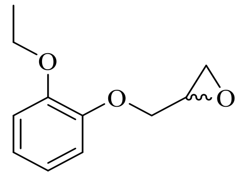	**6-14**	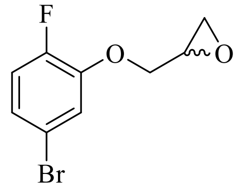	**6-27**	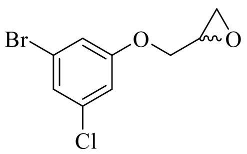
**6-2**	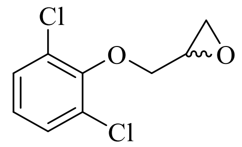	**6-15**	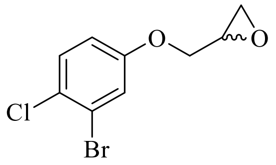	**6-28**	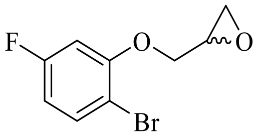
**6-3**	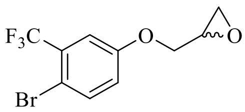	**6-16**	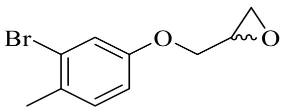	**6-29**	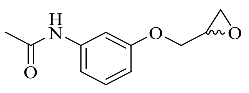
**6-4**	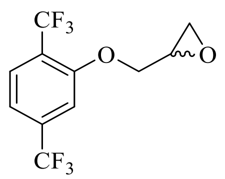	**6-17**	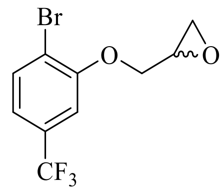	**6-30**	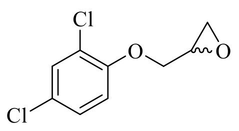

**6-5**	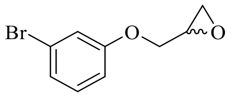	**6-18**	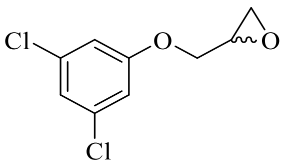	**6-31**	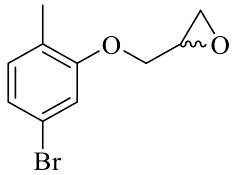
**6-6**	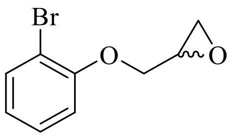	**6-19**	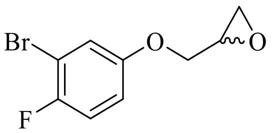	**6-32**	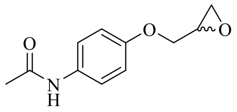
**6-7**	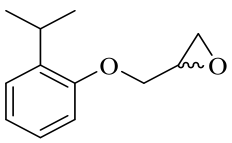	**6-20**	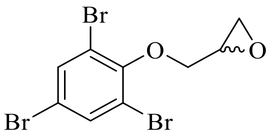	**6-33**	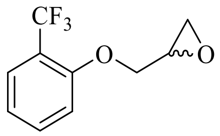
**6-8**	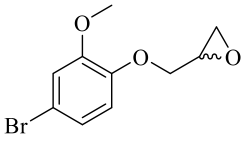	**6-21**	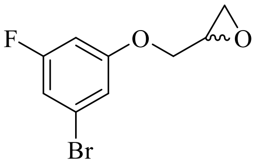	**6-34**	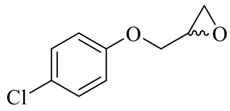
**6-9**	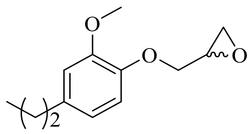	**6-22**	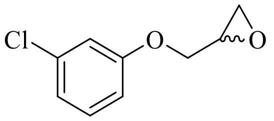	**6-35**	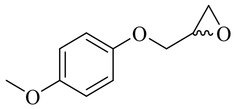
**6-10**	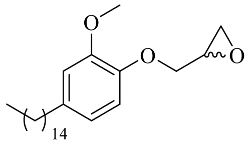	**6-23**	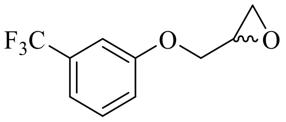	**6-36**	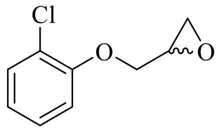
**6-11**	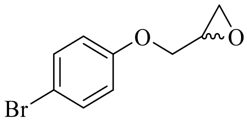	**6-24**	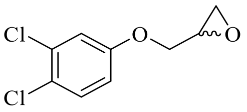	**6-37**	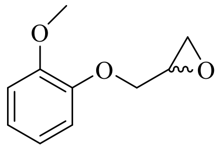
**6-12**	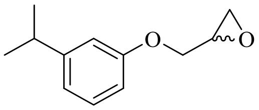	**6-25**	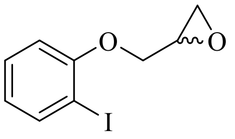		
**6-13**	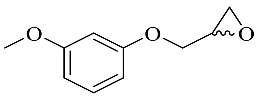	**6-26**	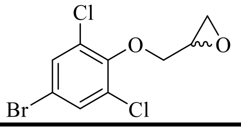		

2. Copies of NMR (^{1}H and ^{13}C) Compounds

Copies of NMR (^{1}H and ^{13}C) of Compound 5-1

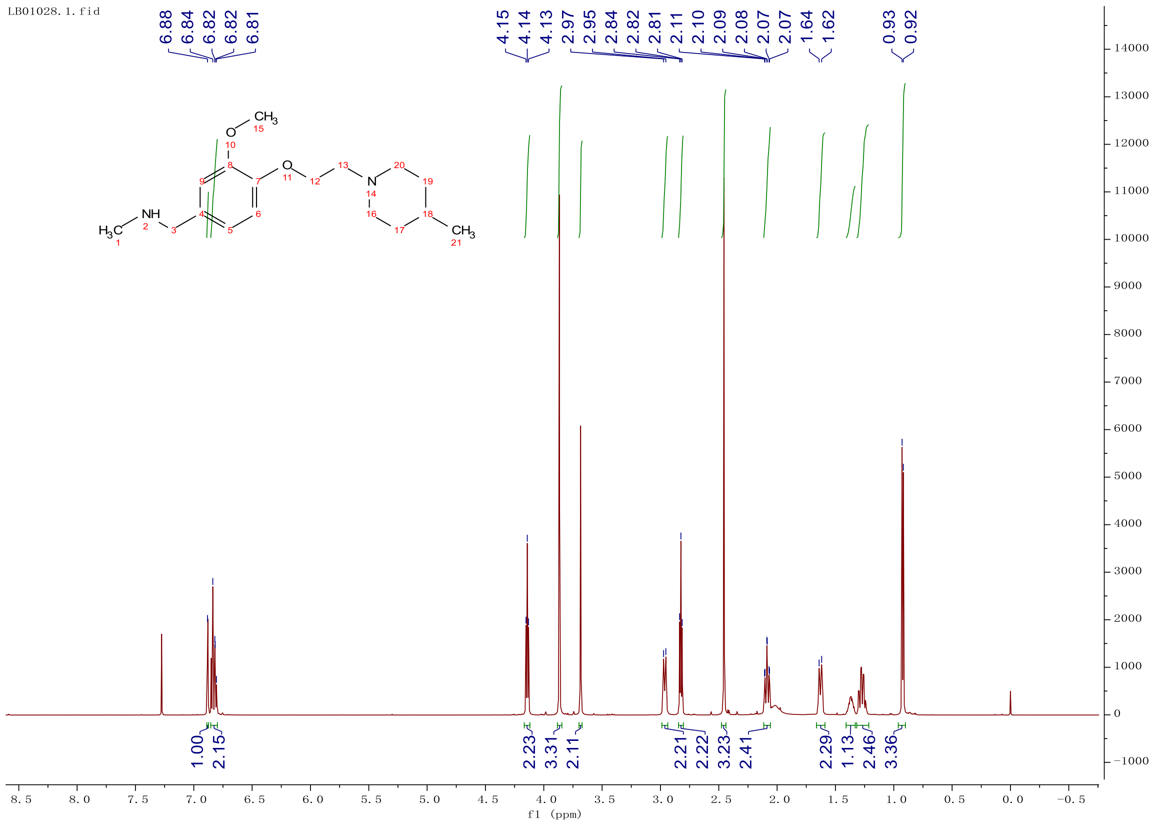

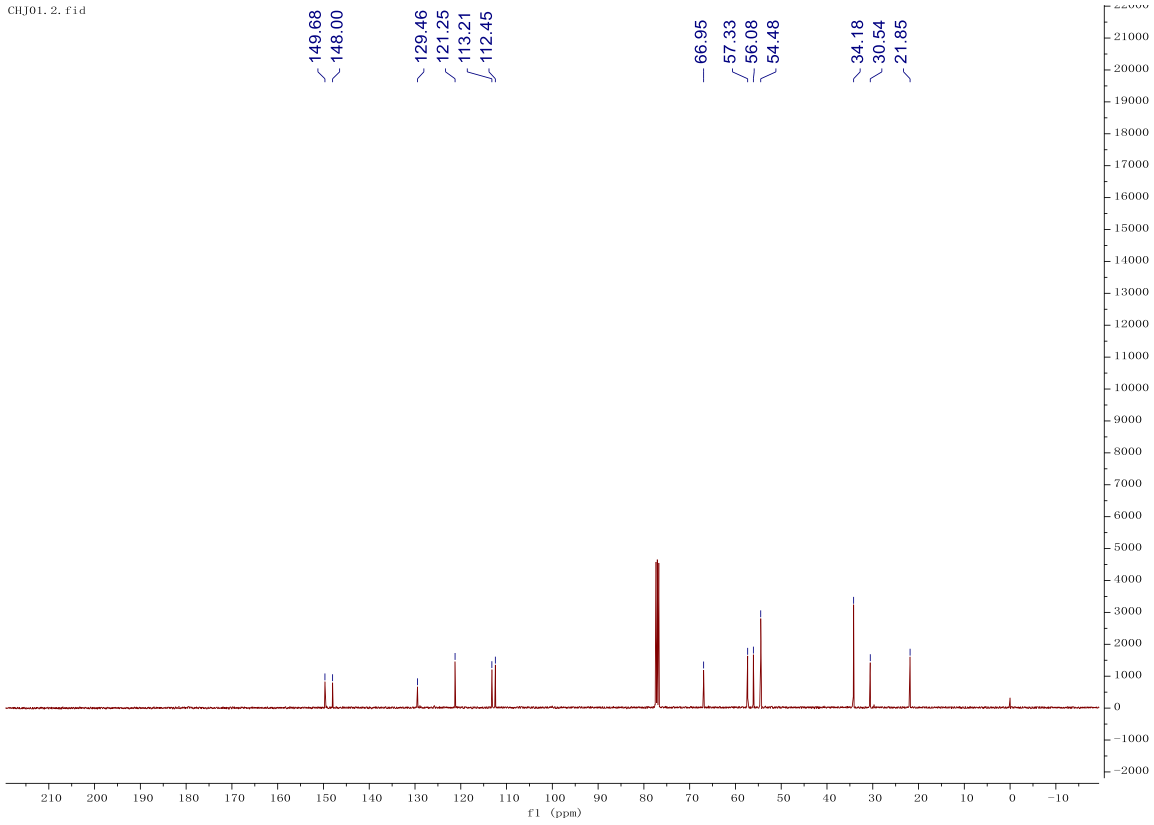

Copies of NMR (^1H and ^{13}C) of Compound 5-2

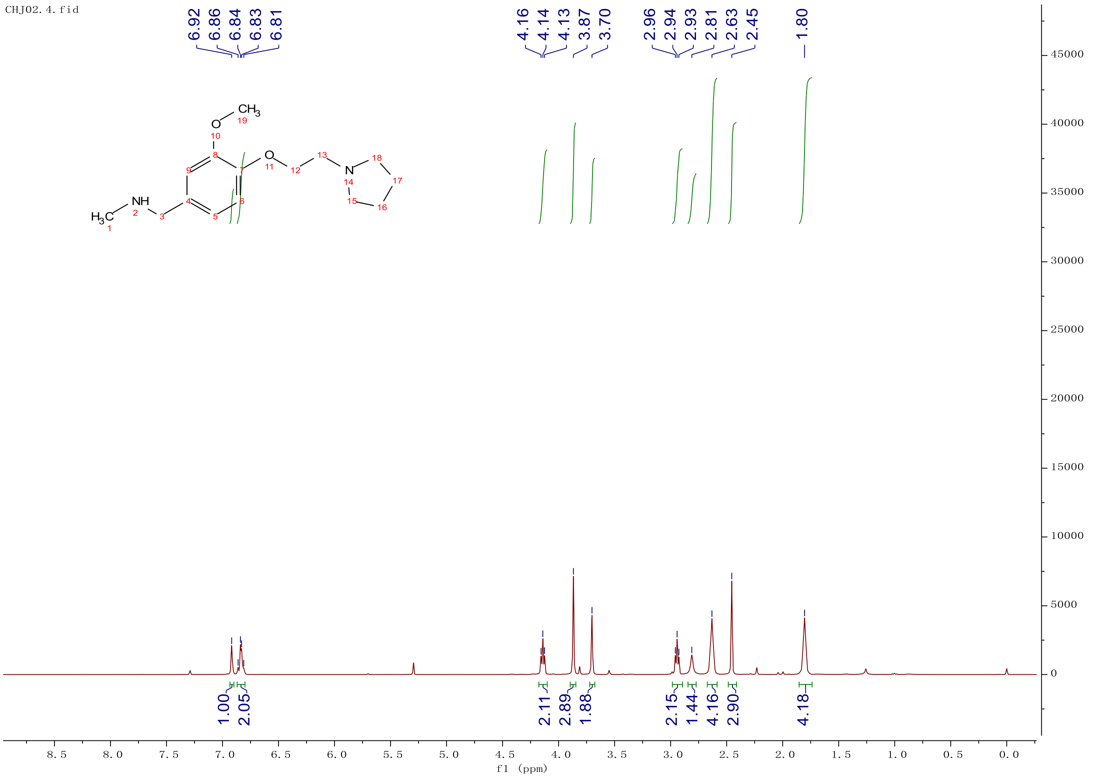

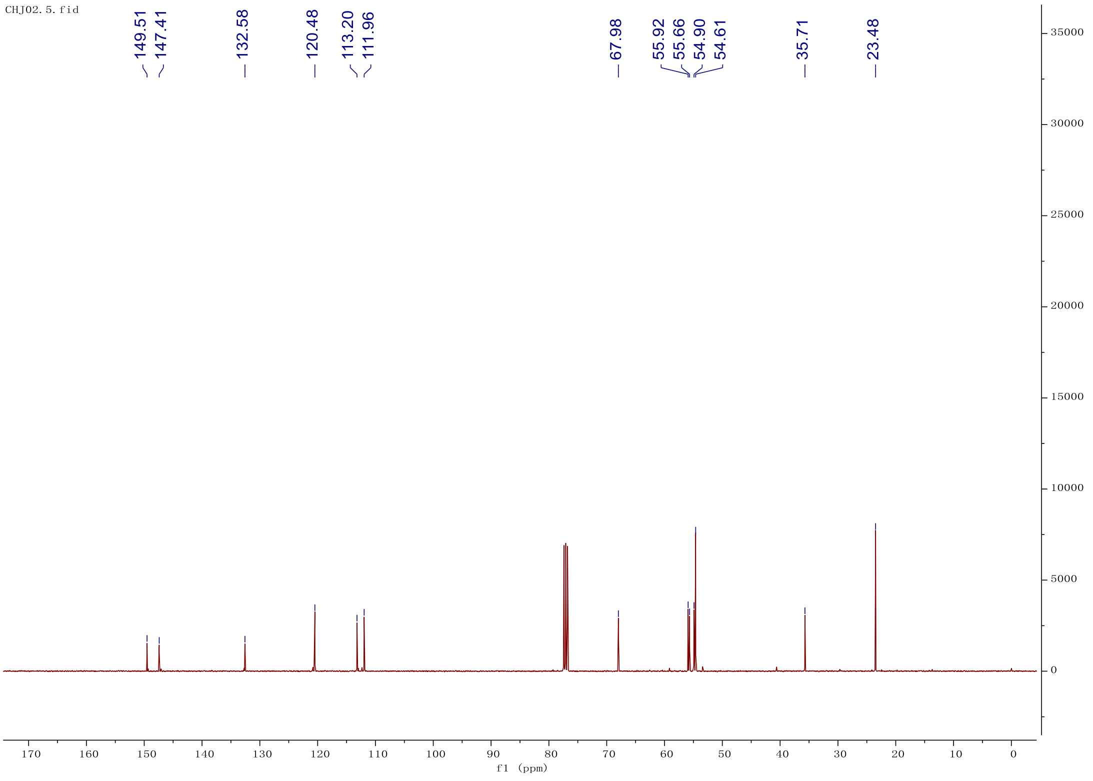

Copies of NMR (^{1}H and ^{13}C) of Compound 5-3

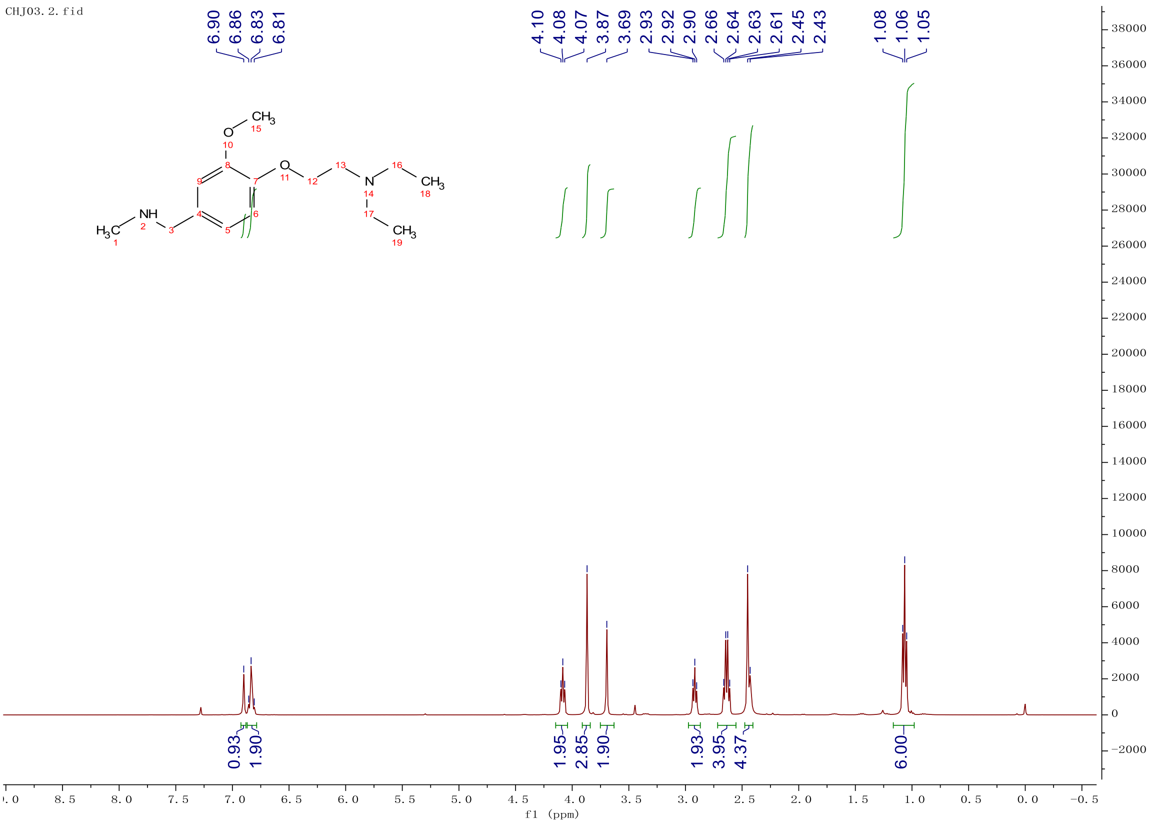

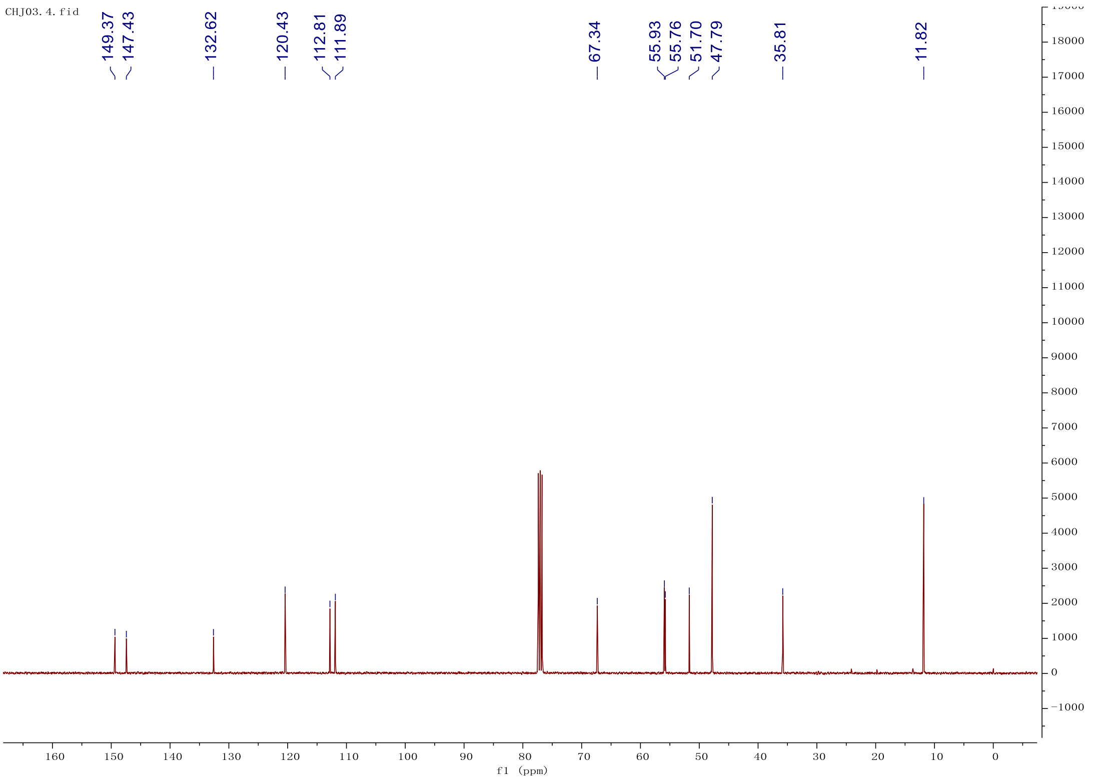

Copies of NMR (^{1}H and ^{13}C) of Compound 5-4

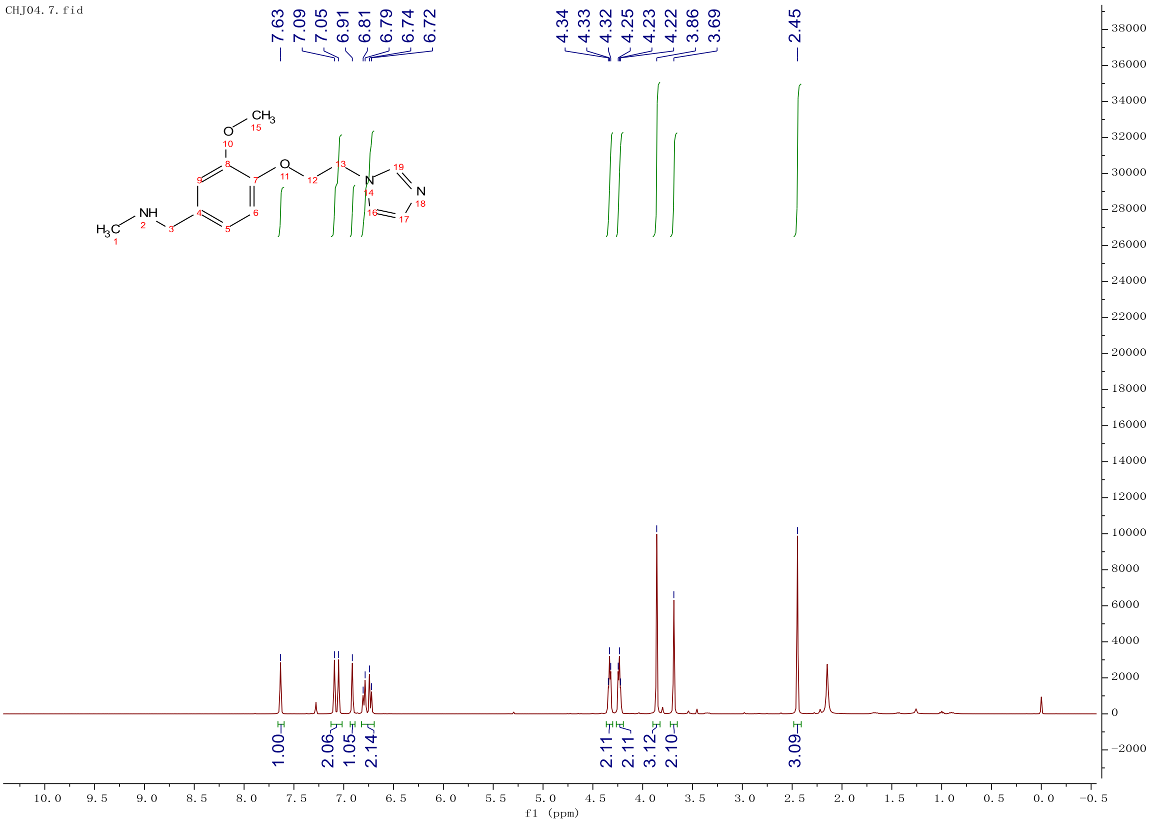

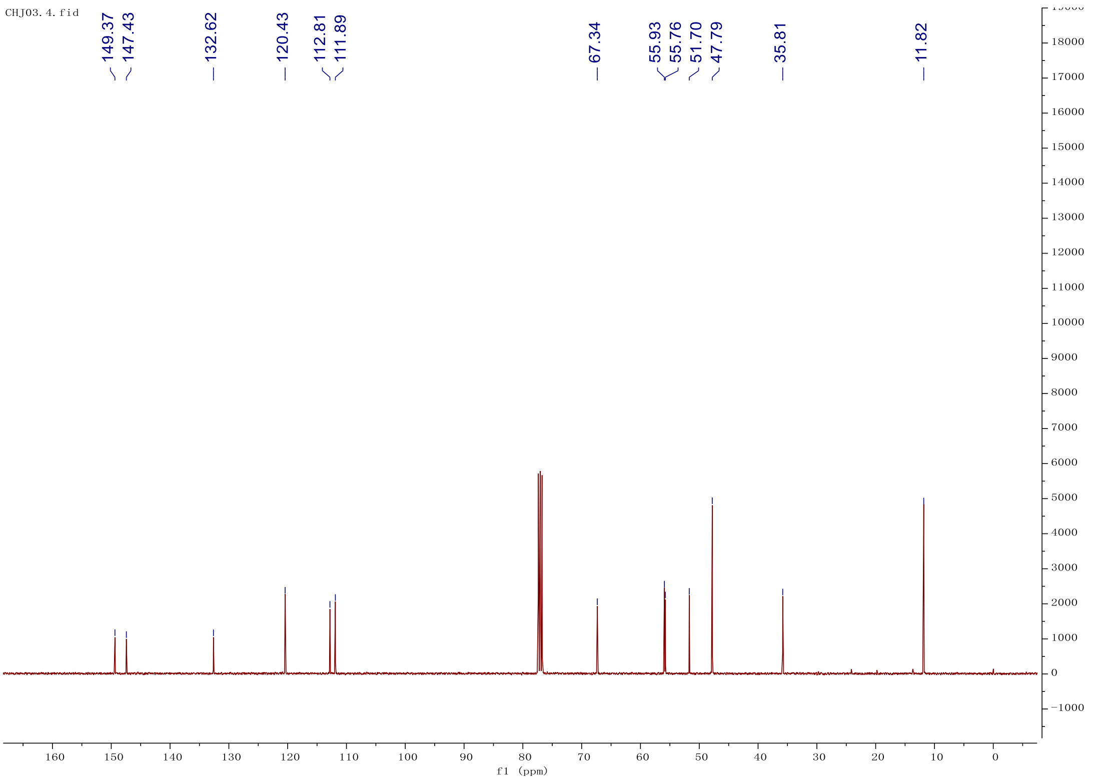

NMR (^{1}H and ^{13}C) and IR (KBr) of Compound CHJ02029:

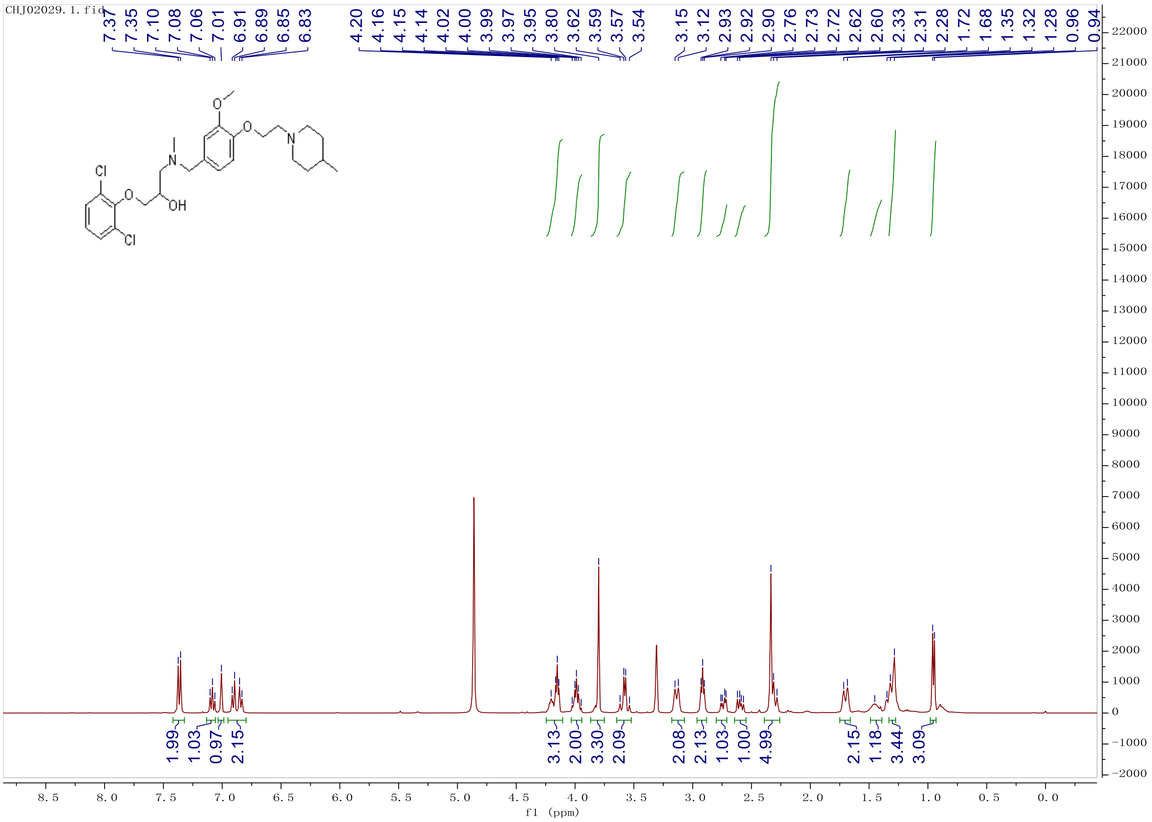

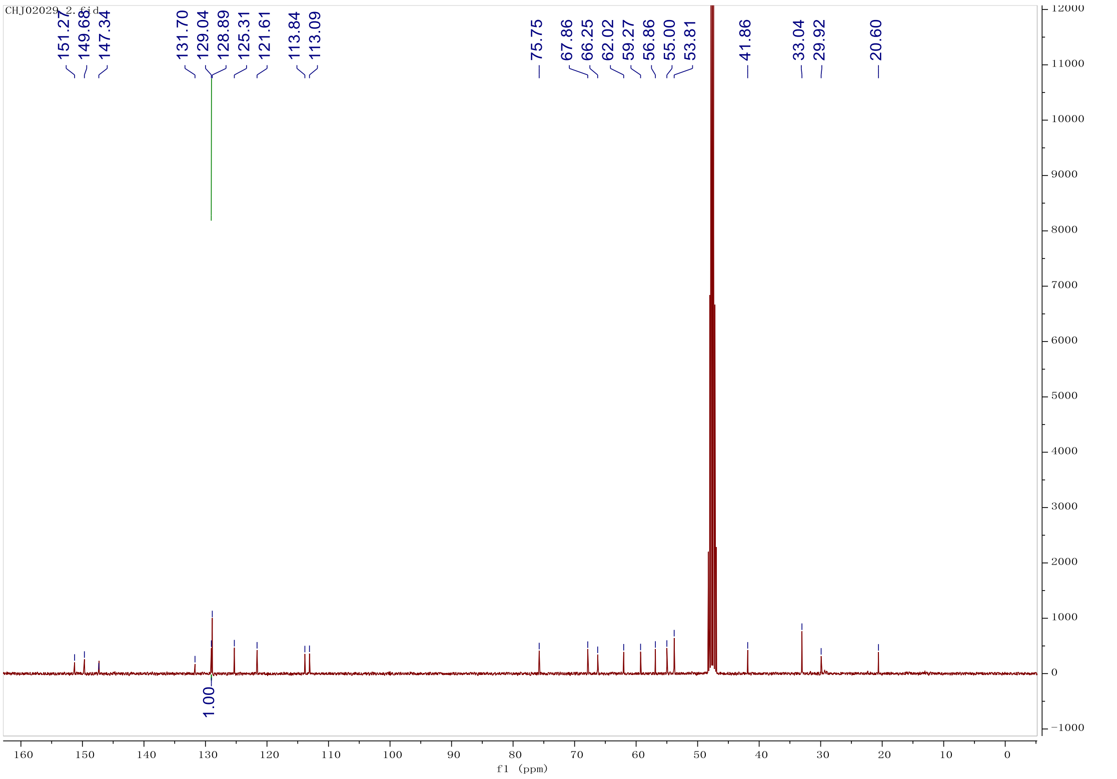

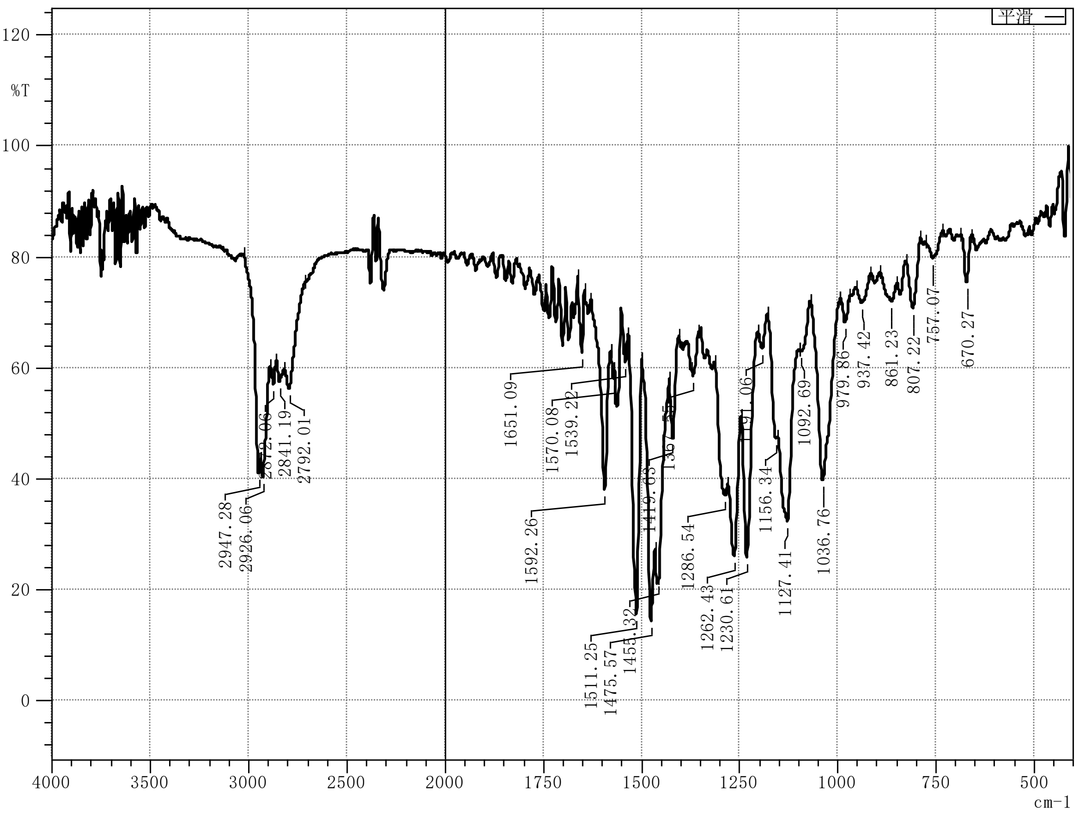

NMR (^{1}H and ^{13}C) and IR (KBr) of Compound CHJ02049:

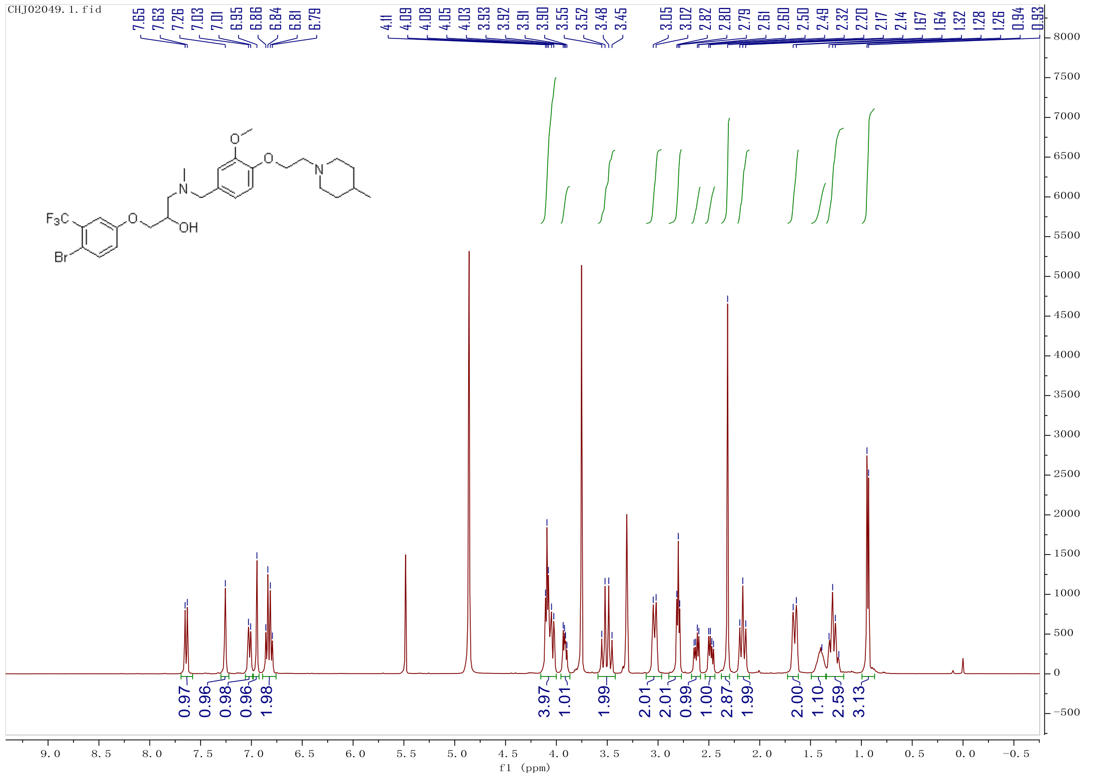

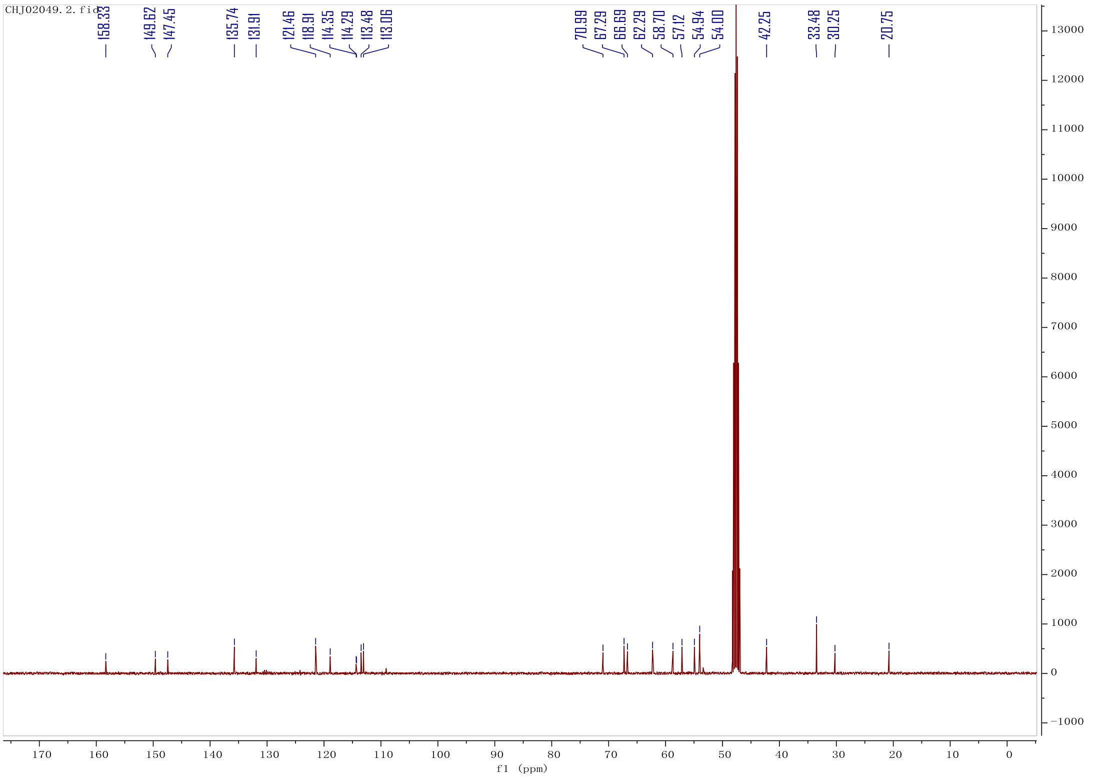
CHJ02049.2.fid
158.33
149.62
147.45
135.74
131.91
121.46
118.91
114.35
114.29
113.48
113.06
70.99
67.29
66.69
62.29
58.70
57.12
54.94
54.00
42.25
33.48
30.25
20.75
f1 (ppm)

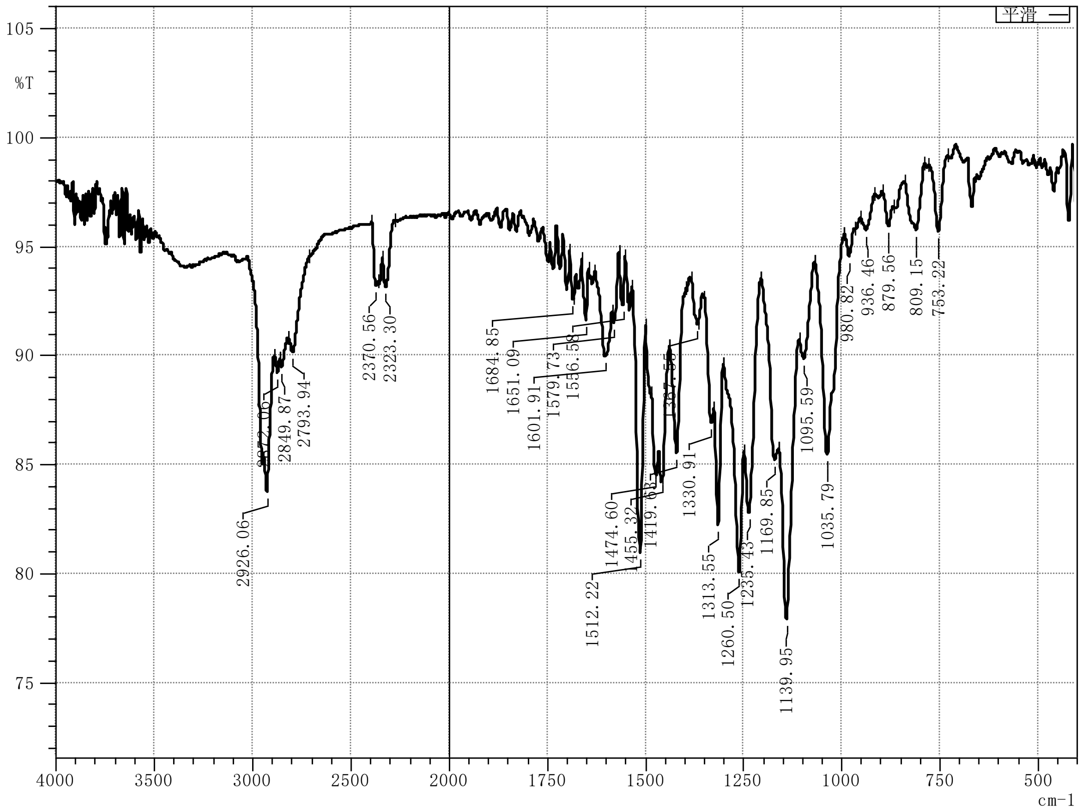
%T
2926.06
2849.87
2793.94
2370.56
2323.30
1684.85
1651.09
1601.91
1579.73
1512.22
1474.60
1419.68
1330.91
1313.55
1260.50
1235.43
1169.85
1139.95
1095.59
1035.79
980.82
936.46
879.56
809.15
753.22
cm-1

NMR (^{1}H and ^{13}C) and IR (KBr) of Compound CHJ02050:

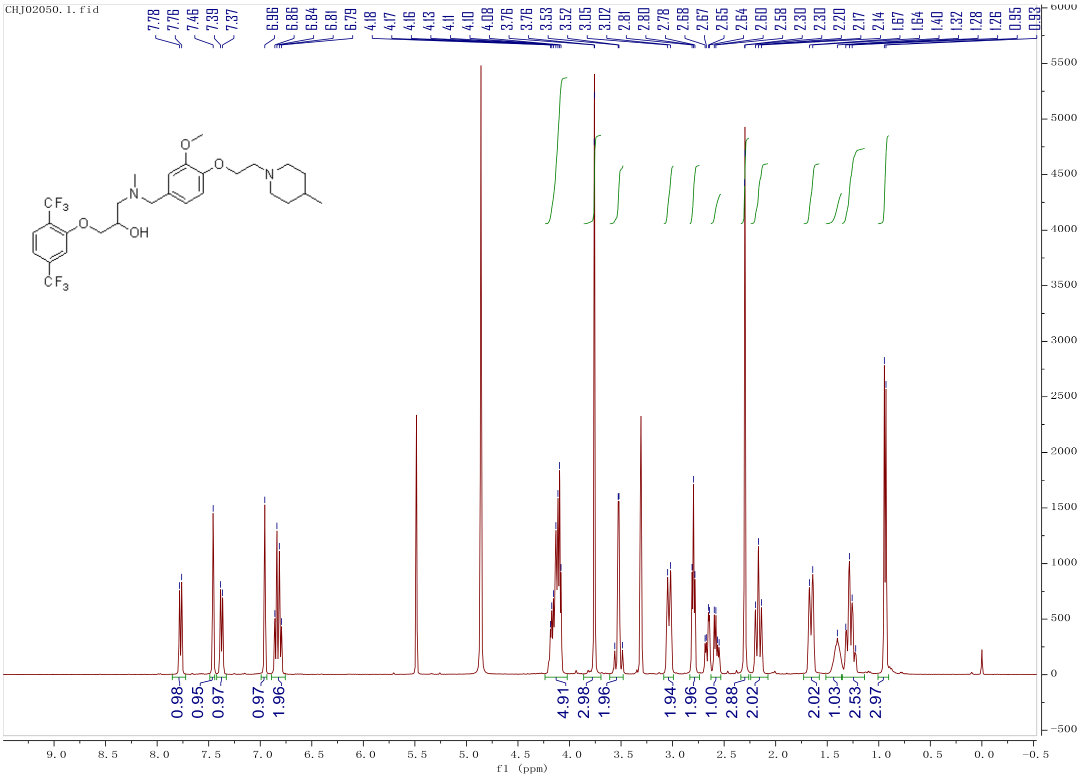

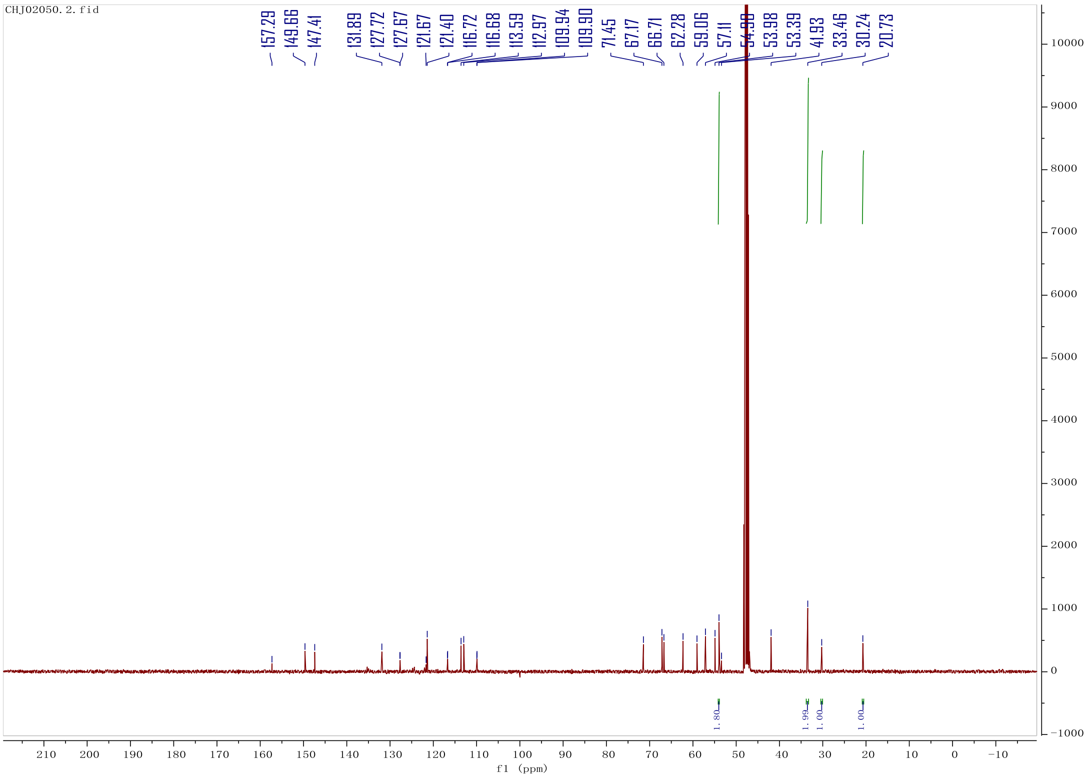

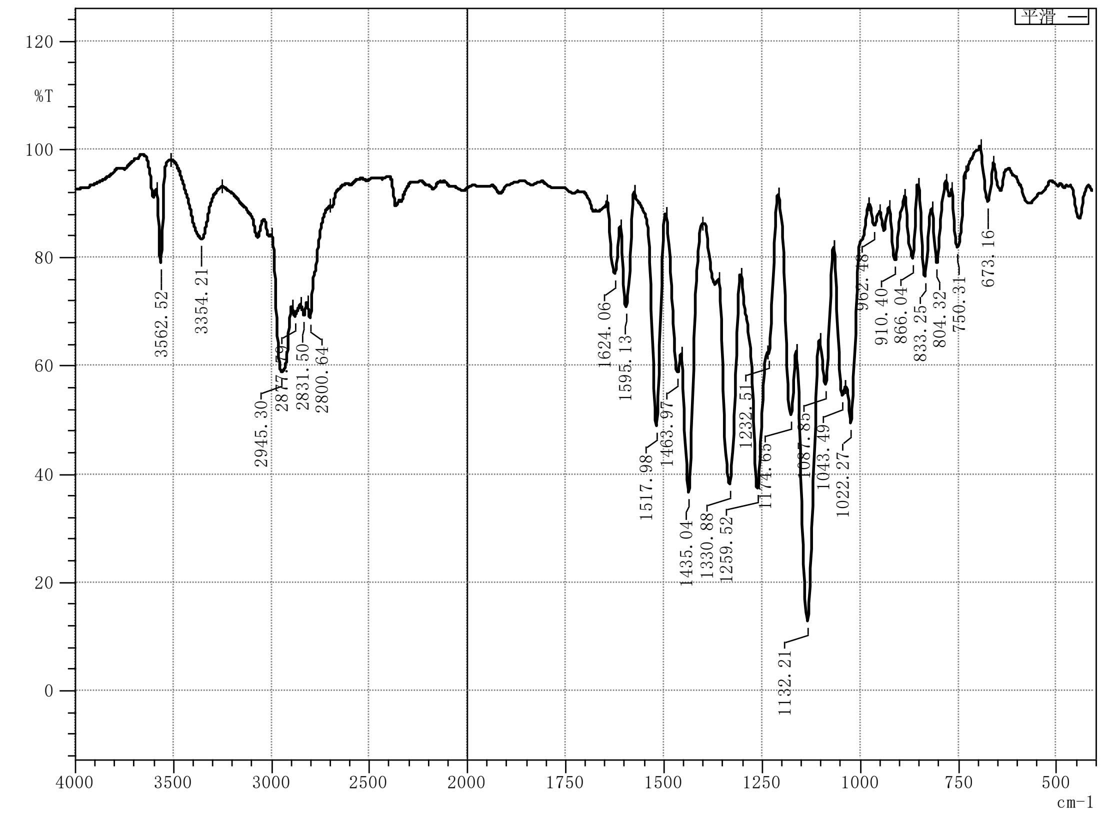

NMR (^{1}H and ^{13}C) and IR (KBr) of Compound CHJ03001:

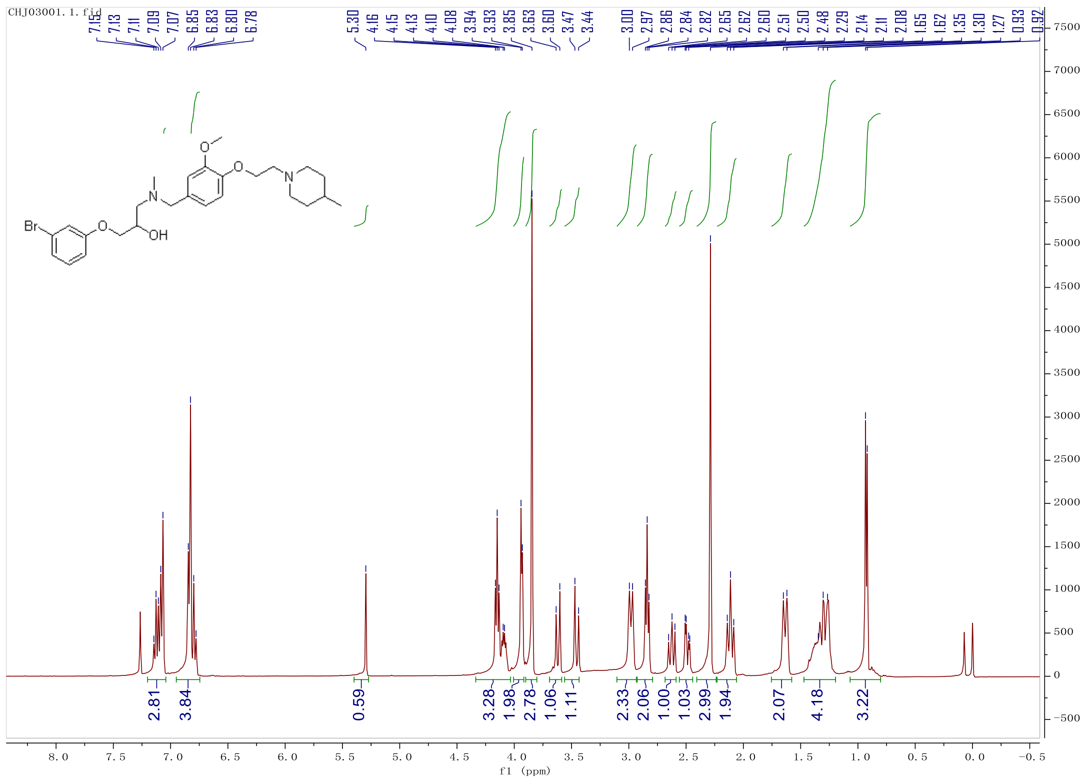

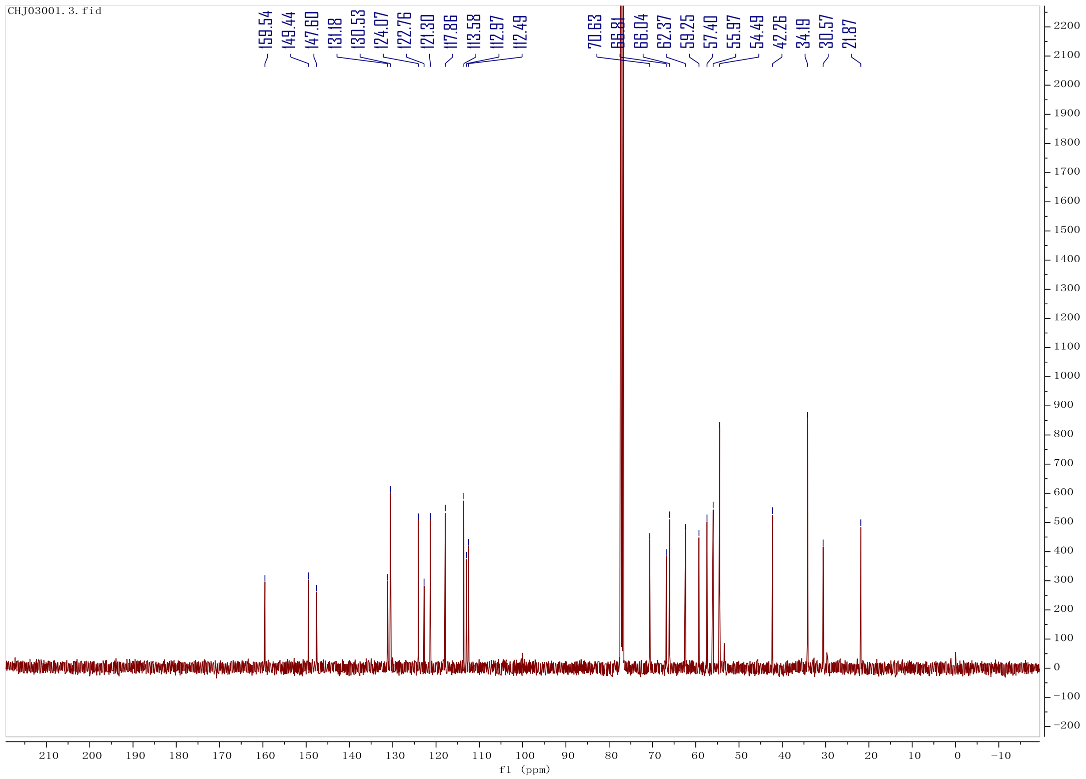
CHJ03001.3.fid
159.54
149.44
147.60
131.18
130.53
124.07
122.76
121.30
117.86
113.58
112.97
112.49
70.63
66.81
66.04
62.37
59.25
57.40
55.97
54.49
42.26
34.19
30.57
21.87
f1 (ppm)

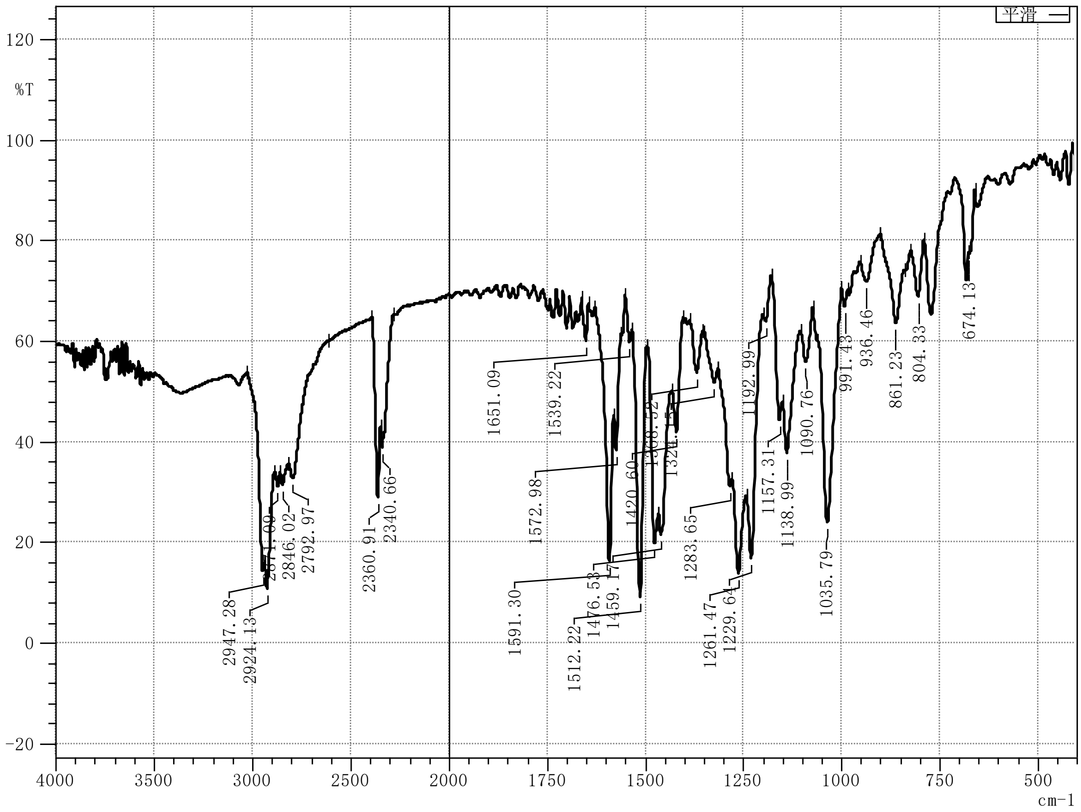
平滑
%T
cm-1
2947.28
2924.13
2846.02
2792.97
2360.91
2340.66
1651.09
1539.22
1572.98
1591.30
1512.22
1476.53
1459.17
1420.60
1283.65
1261.47
1229.64
1192.99
1157.31
1138.99
1090.76
1035.79
991.43
936.46
861.23
804.33
674.13

NMR (^{1}H and ^{13}C) and IR (KBr) of Compound CHJ03003:

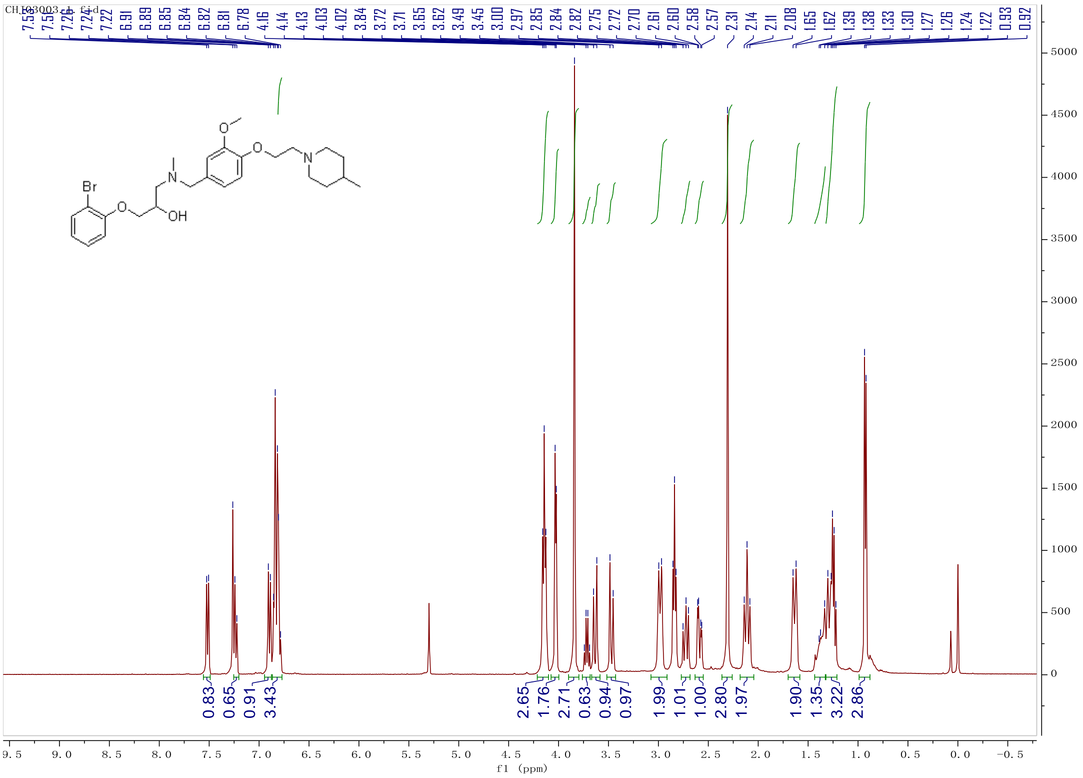

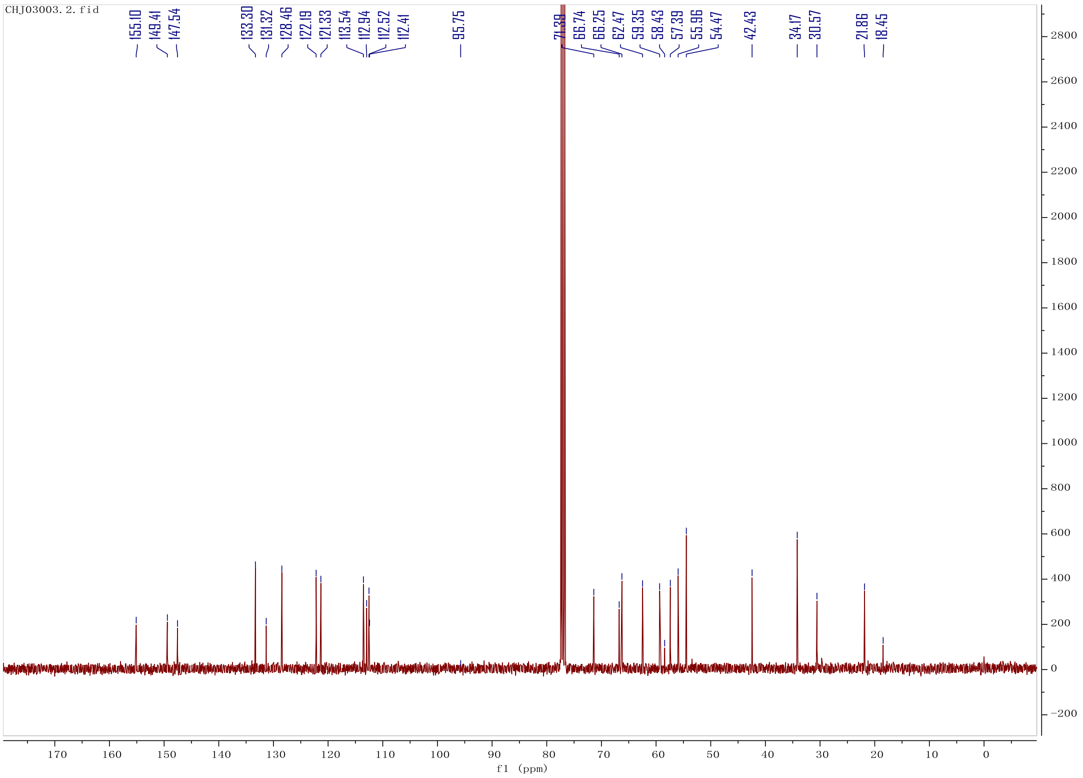

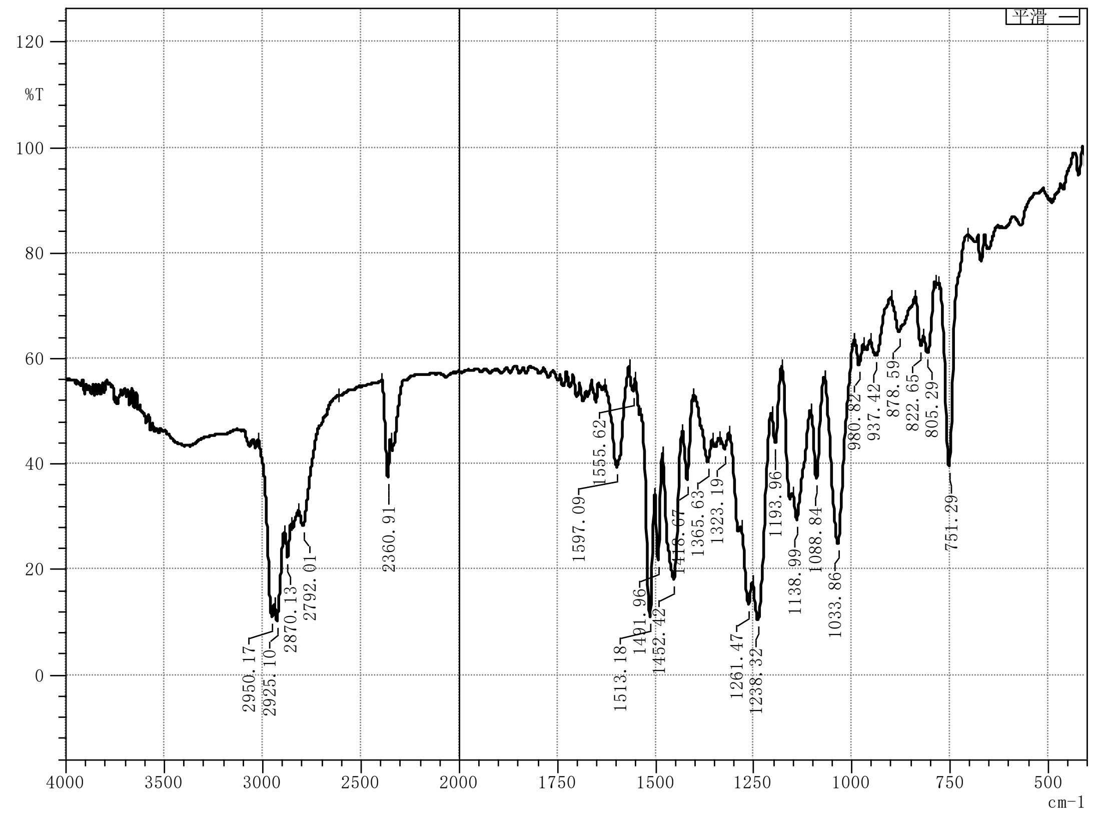

NMR (^{1}H and ^{13}C) and IR (KBr) of Compound CHJ03004:

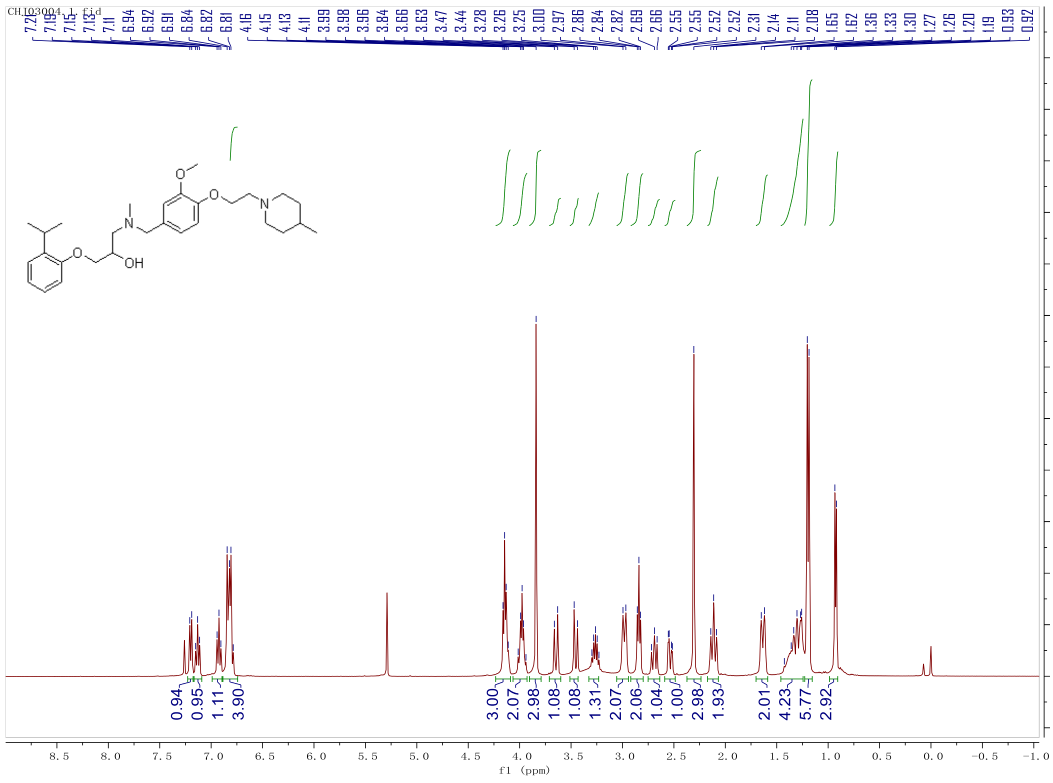

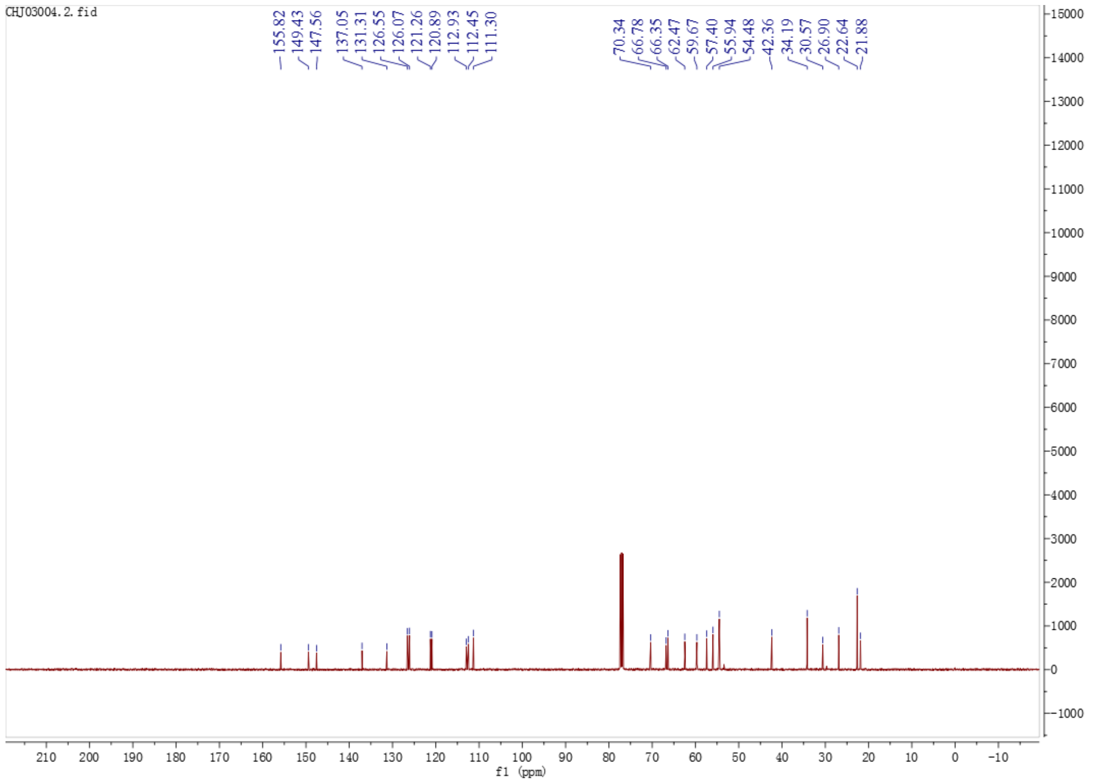
CHJ03004.2.fid
155.82
149.43
147.56
137.05
131.31
126.55
126.07
121.26
120.89
112.93
112.45
111.30
70.34
66.78
66.35
62.47
59.67
57.40
55.94
54.48
42.36
34.19
30.57
26.90
22.64
21.88
f1 (ppm)

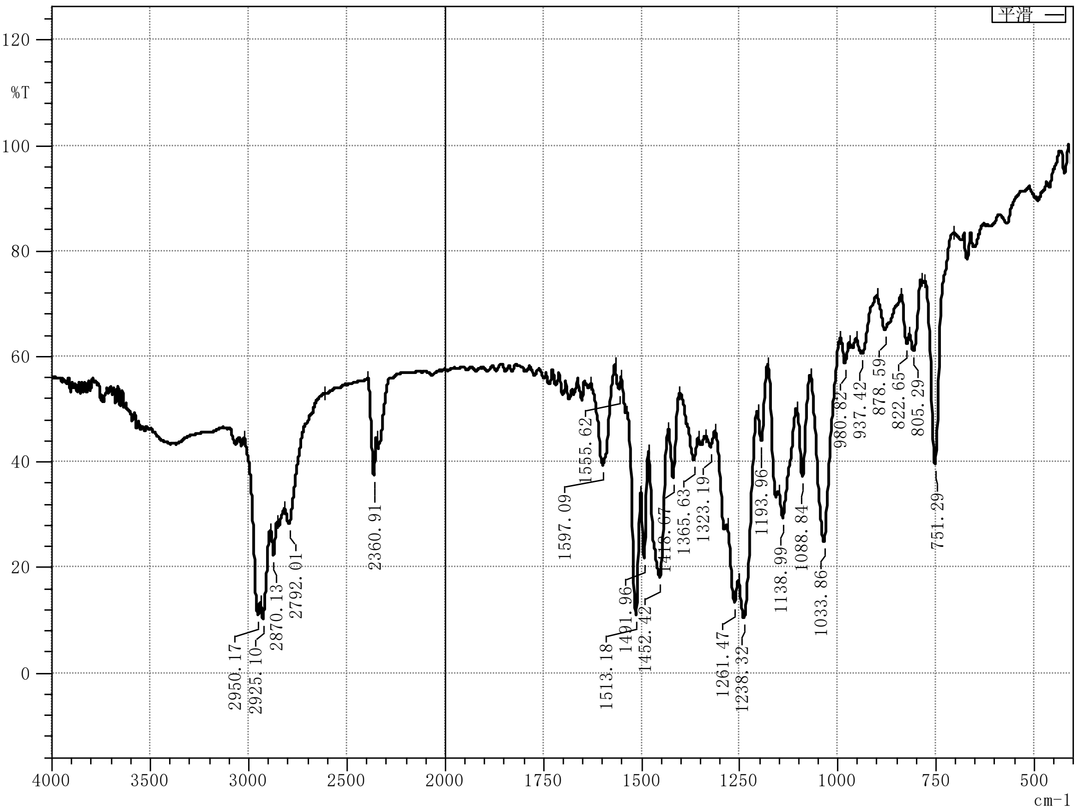
%T
2950.17
2925.10
2870.13
2792.01
2360.91
1597.09
1555.62
1513.18
1452.42
1365.63
1323.19
1261.47
1238.32
1193.96
1138.99
1088.84
1033.86
980.82
937.42
878.59
822.65
805.29
751.29
cm-1

NMR (^{1}H and ^{13}C) and IR (KBr) of Compound CHJ03005:

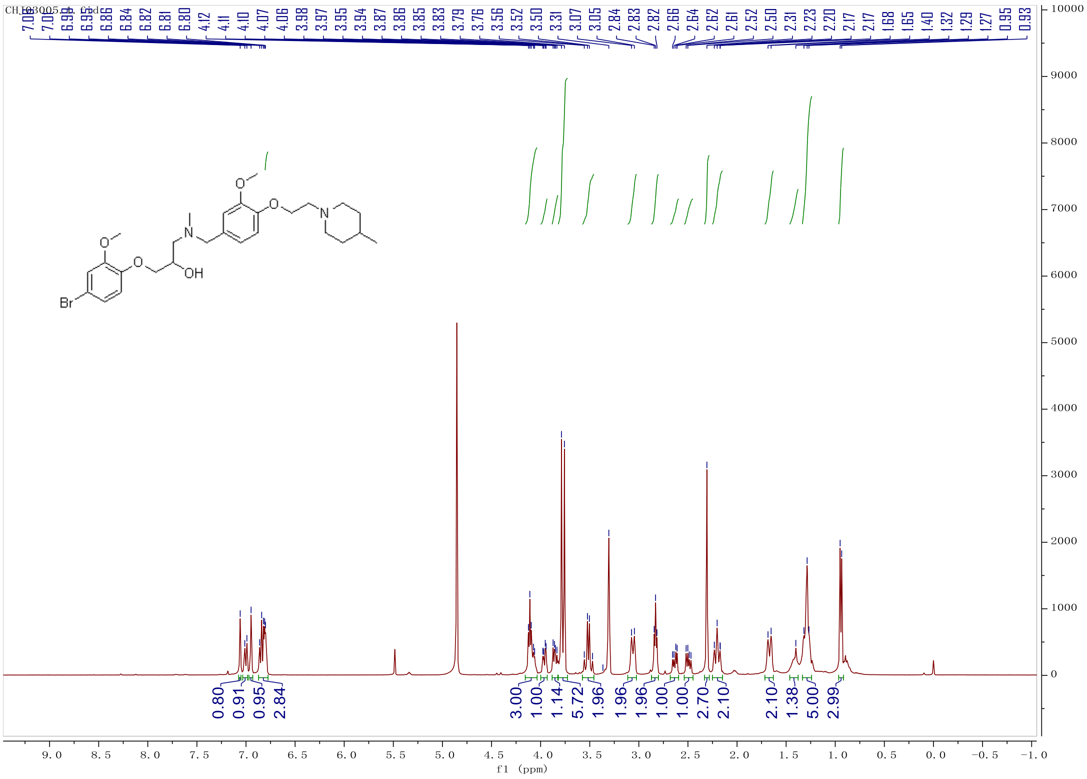

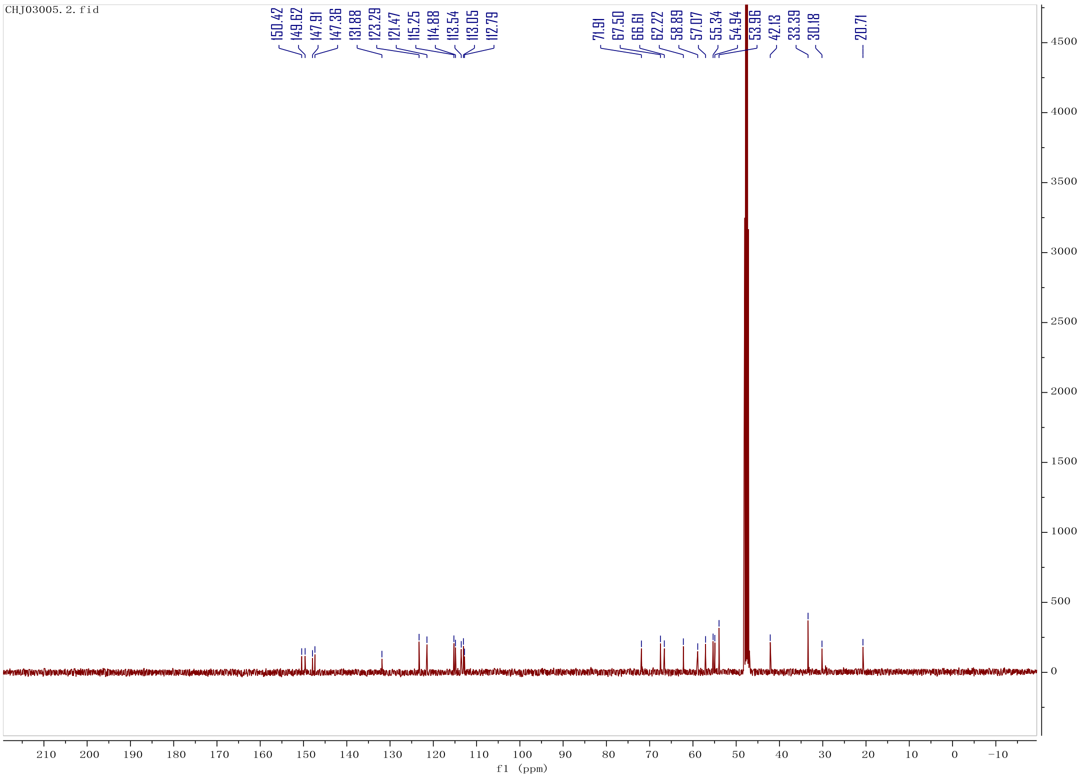

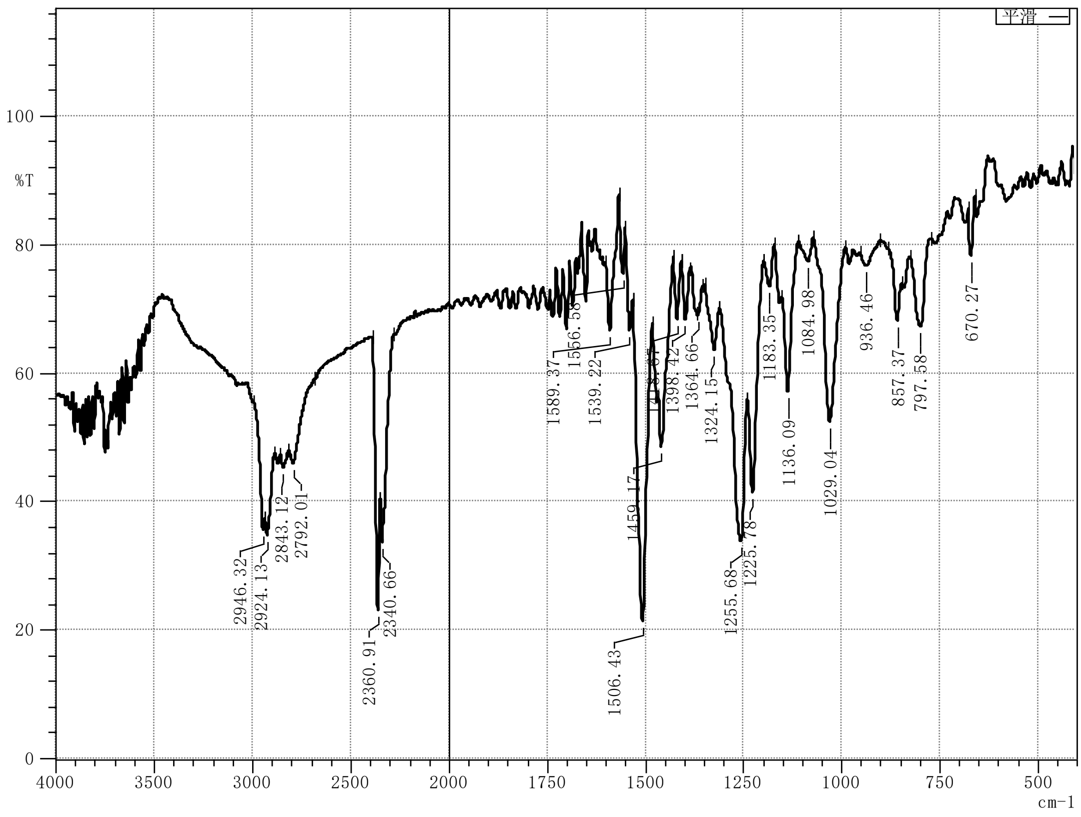

NMR (^{1}H and ^{13}C) and IR (KBr) of Compound CHJ03011:

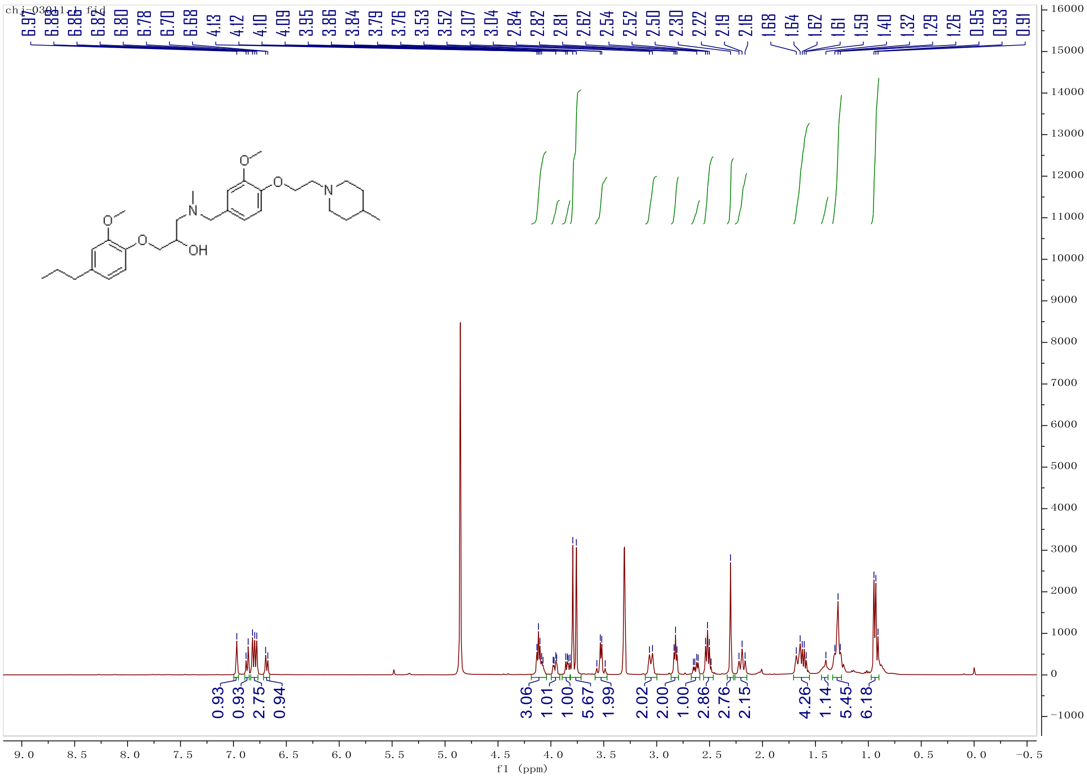

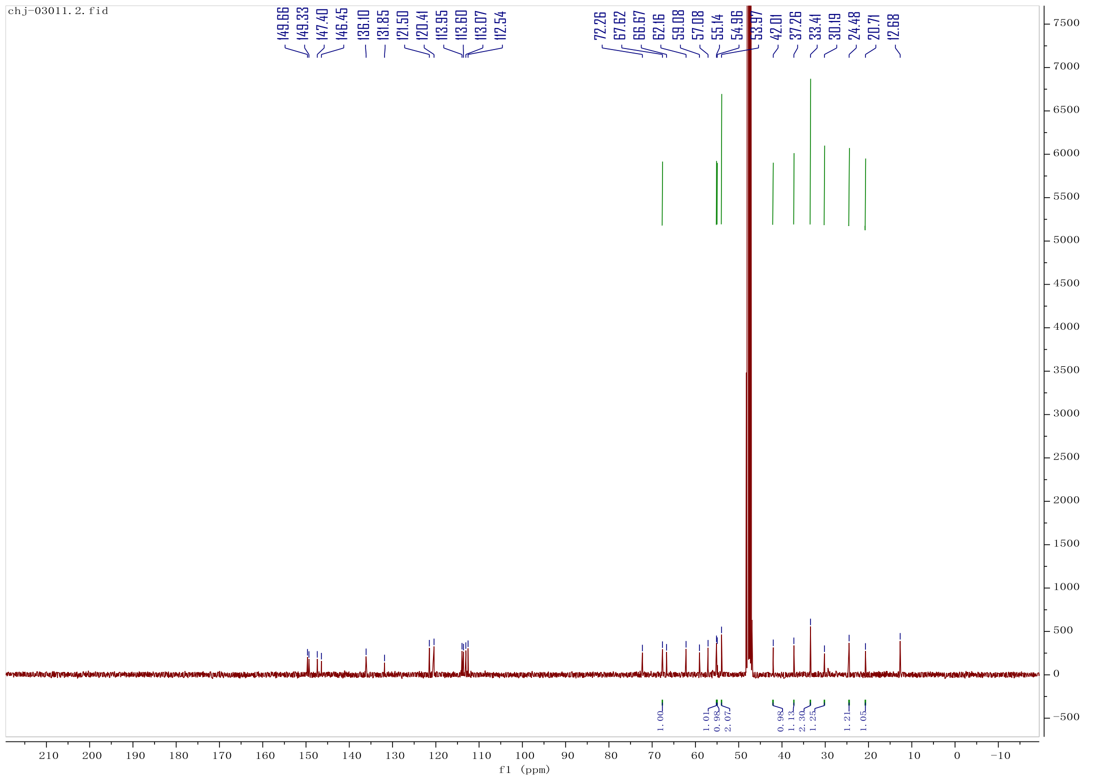

chj-03011.2.fid
149.66
149.33
147.40
146.45
136.10
131.85
121.50
120.41
113.95
113.60
113.07
112.54
72.26
67.62
66.67
62.16
59.08
57.08
55.14
54.96
53.97
42.01
37.26
33.41
30.19
24.48
20.71
12.68
f1 (ppm)

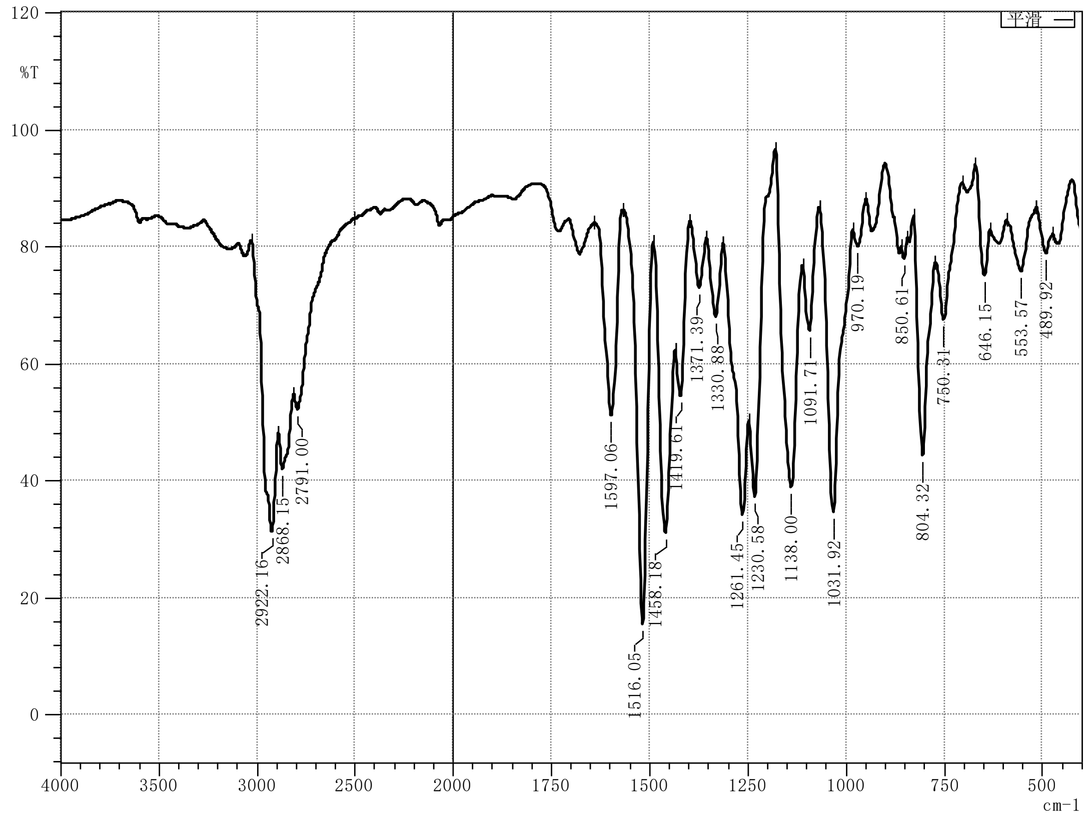

%T
2922.16
2868.15
2791.00
1597.06
1516.05
1458.18
1419.61
1371.39
1330.88
1261.45
1230.58
1138.00
1091.71
1031.92
970.19
850.61
804.32
750.31
646.15
553.57
489.92
cm-1

NMR (^{1}H and ^{13}C) and IR (KBr) of Compound CHJ03012:

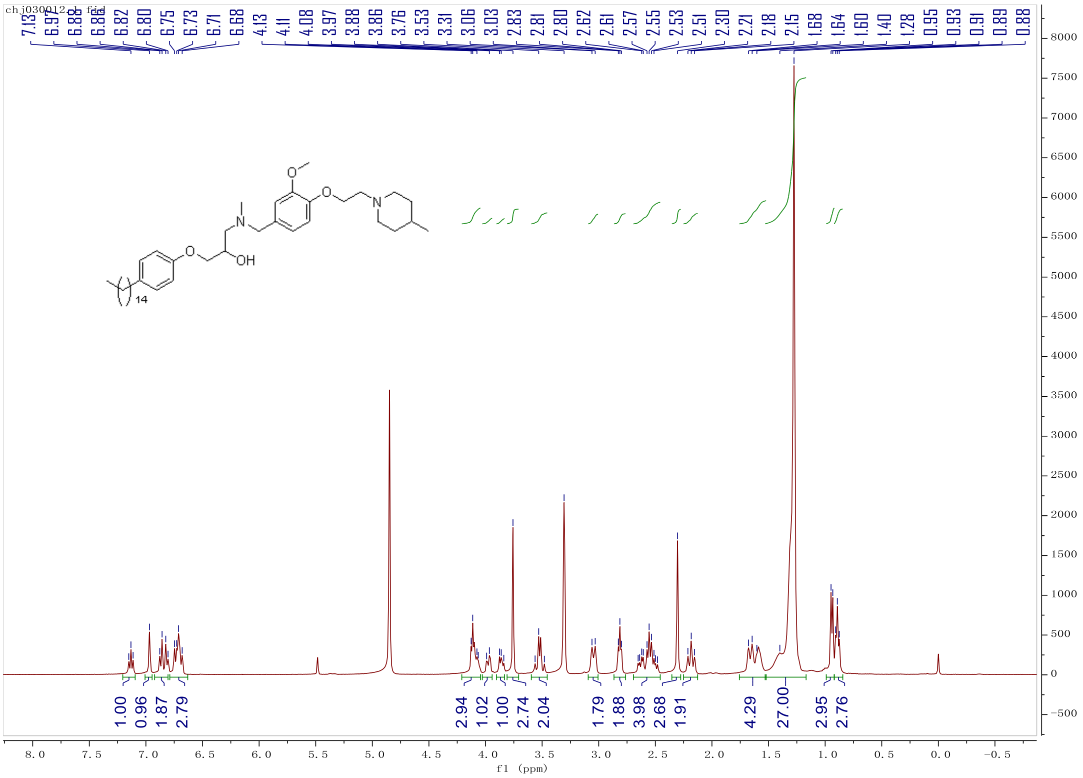

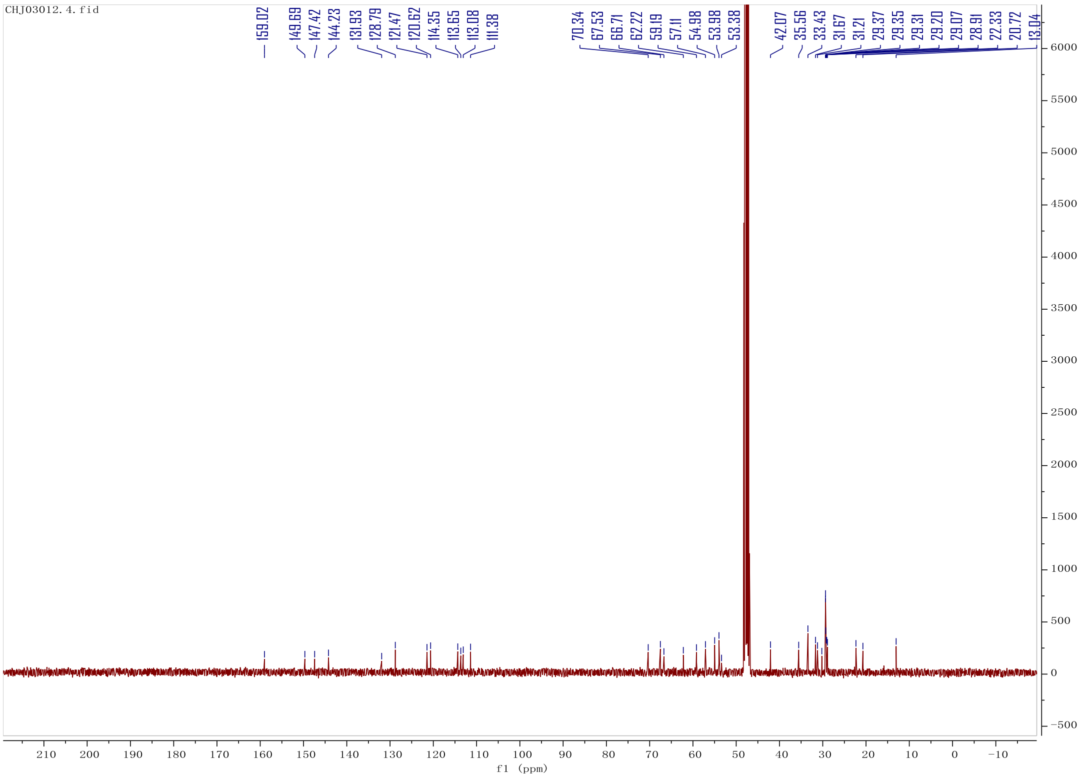

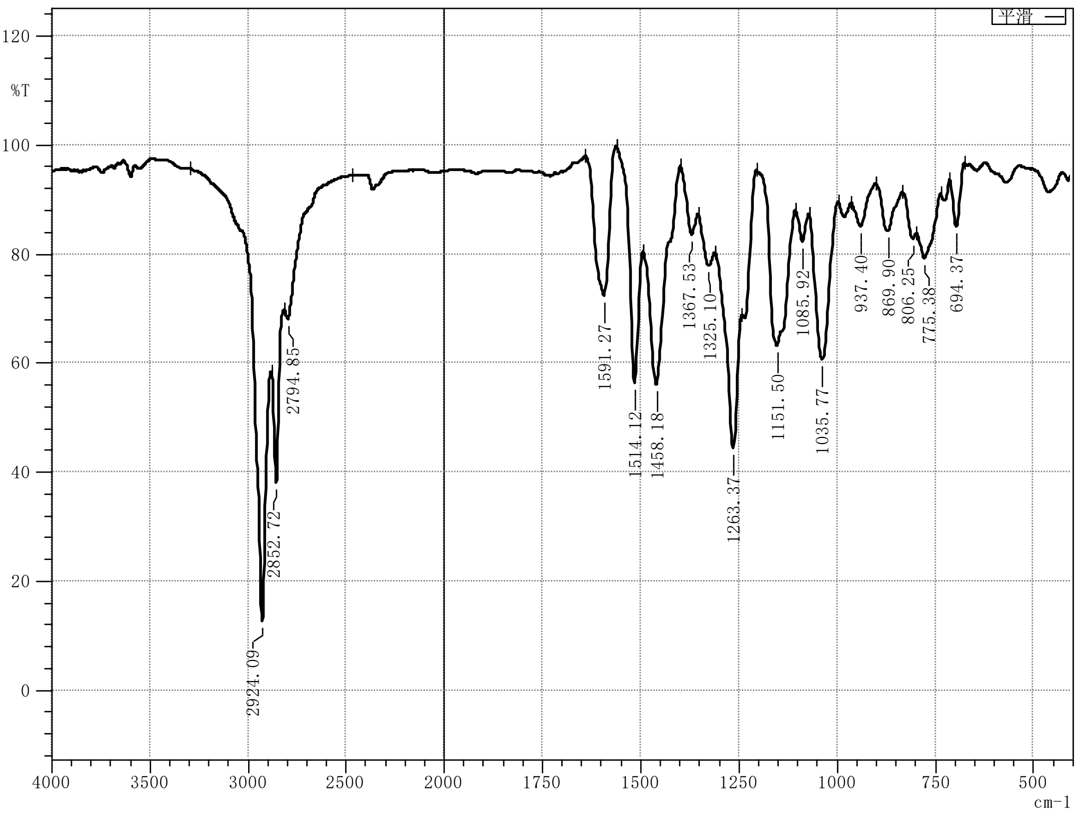

NMR (^{1}H and ^{13}C) and IR (KBr) of Compound CHJ03014:

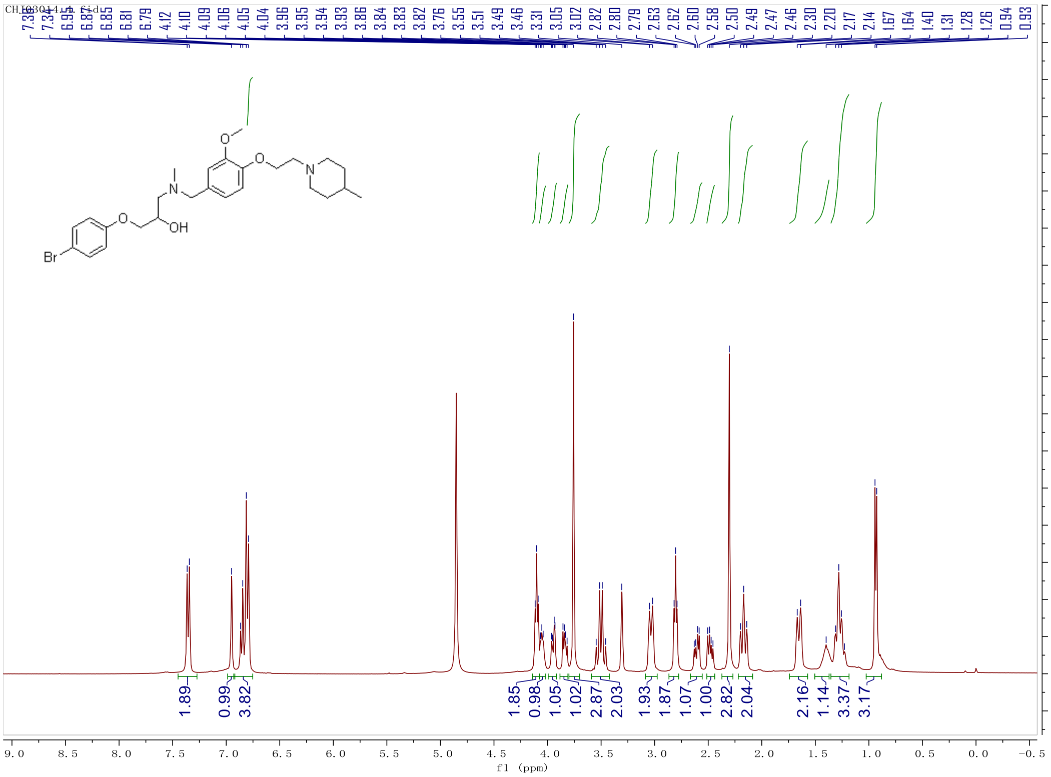

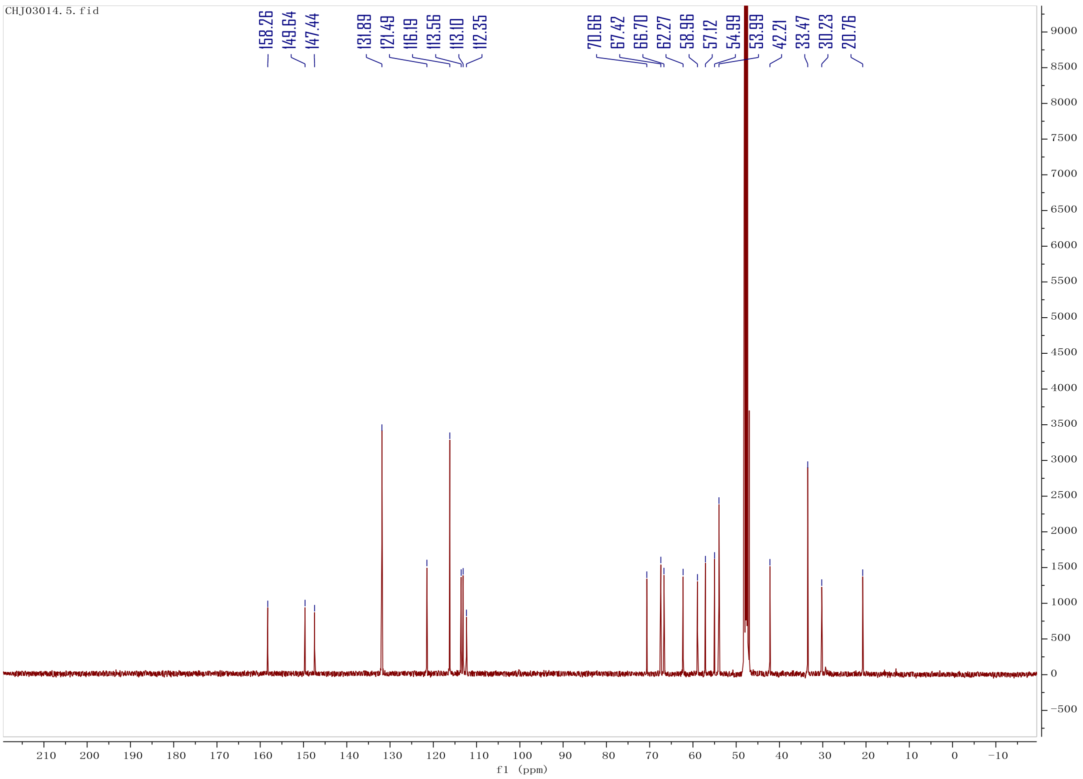

CHJ03014.5.fid
158.26
149.64
147.44
131.89
121.49
116.19
113.56
113.10
112.35
70.66
67.42
66.70
62.27
58.96
57.12
54.99
53.99
42.21
33.47
30.23
20.76
f1 (ppm)

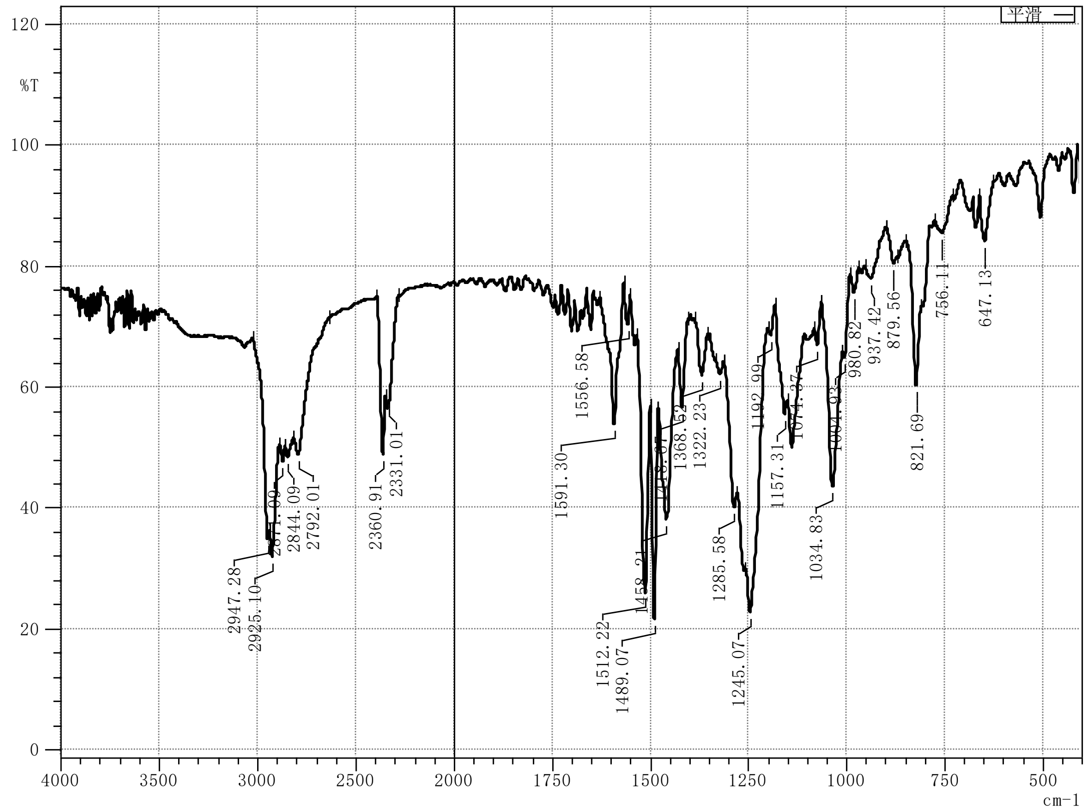

%T
cm-1
2947.28
2925.10
2844.09
2792.01
2360.91
2331.01
1591.30
1556.58
1512.22
1489.07
1368.52
1322.23
1285.58
1245.07
1192.99
1157.31
1034.83
980.82
937.42
879.56
821.69
756.11
647.13

NMR (^{1}H and ^{13}C) and IR (KBr) of Compound CHJ03015:

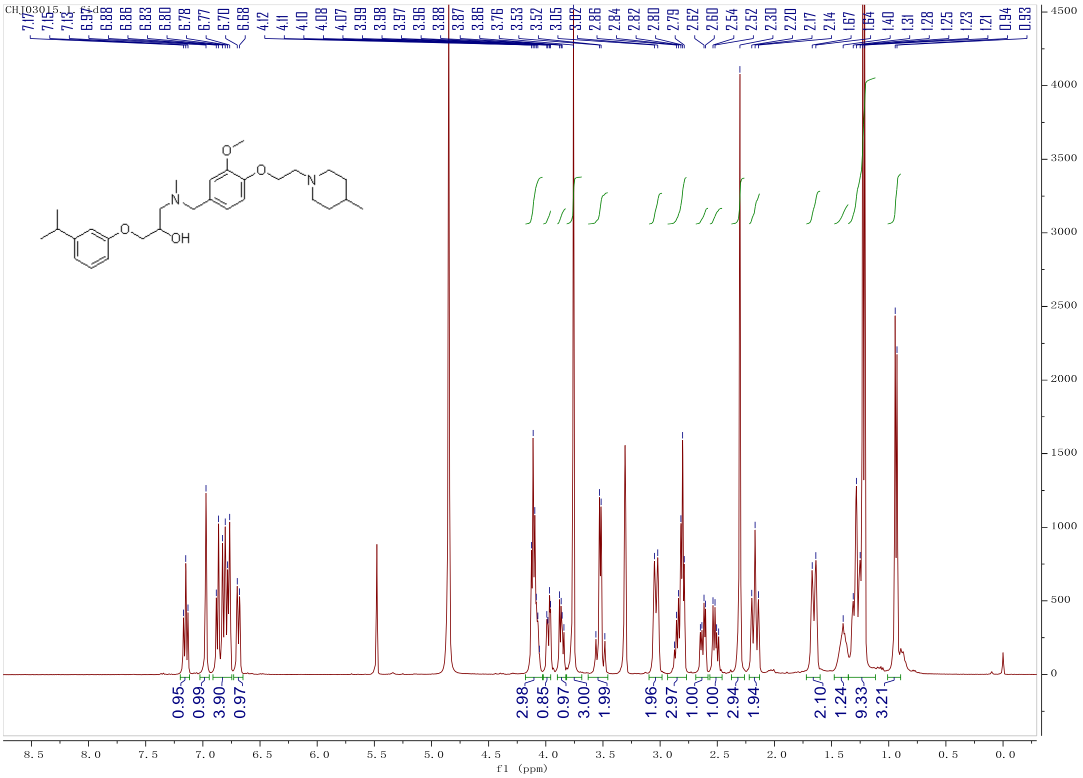

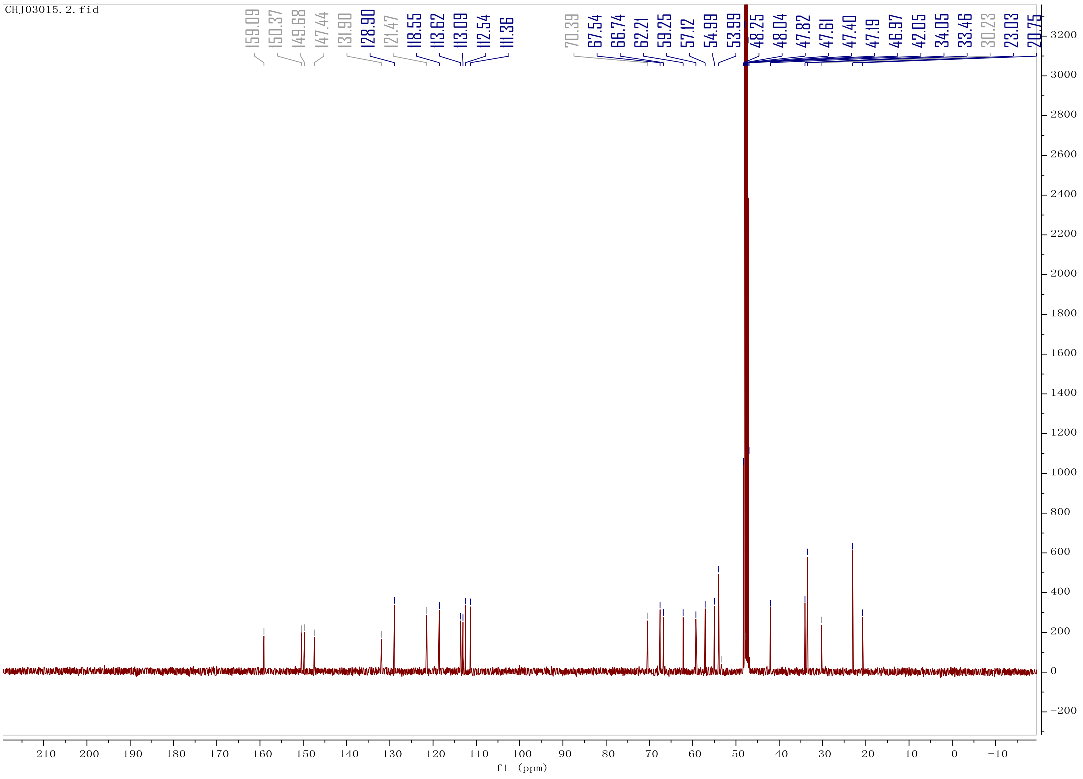

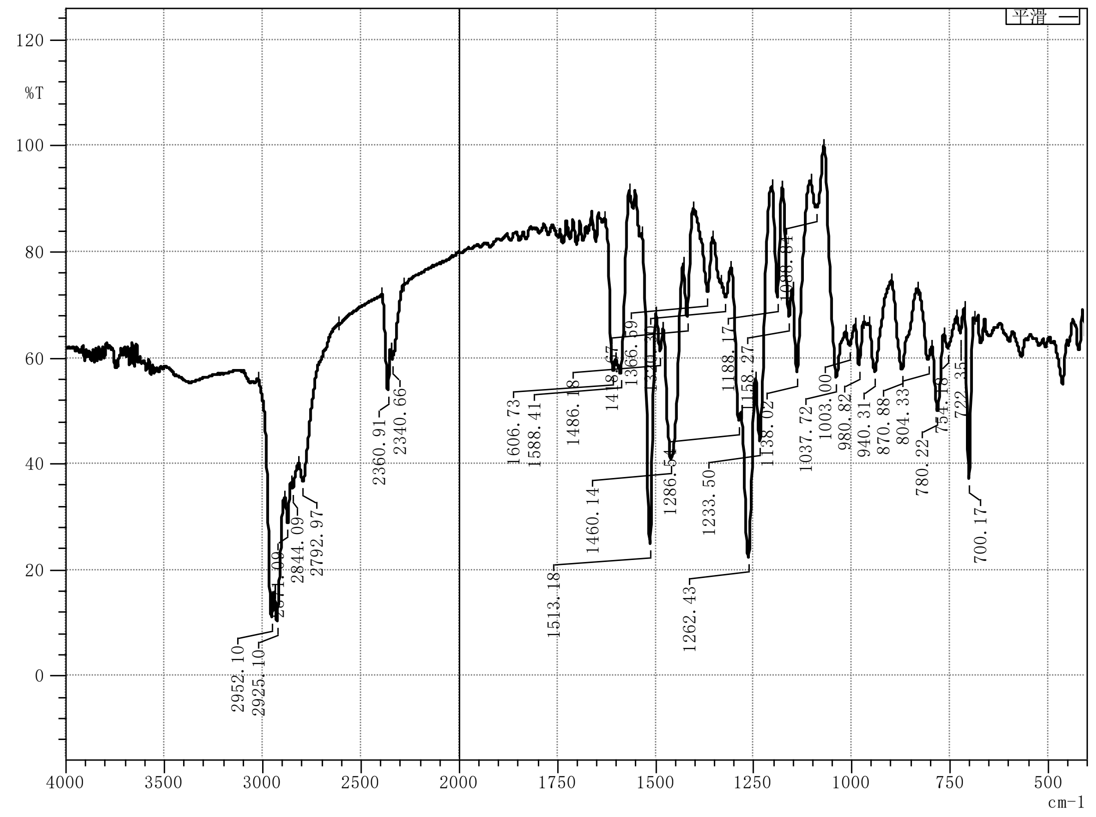

NMR (^{1}H and ^{13}C) and IR (KBr) of Compound CHJ03017:

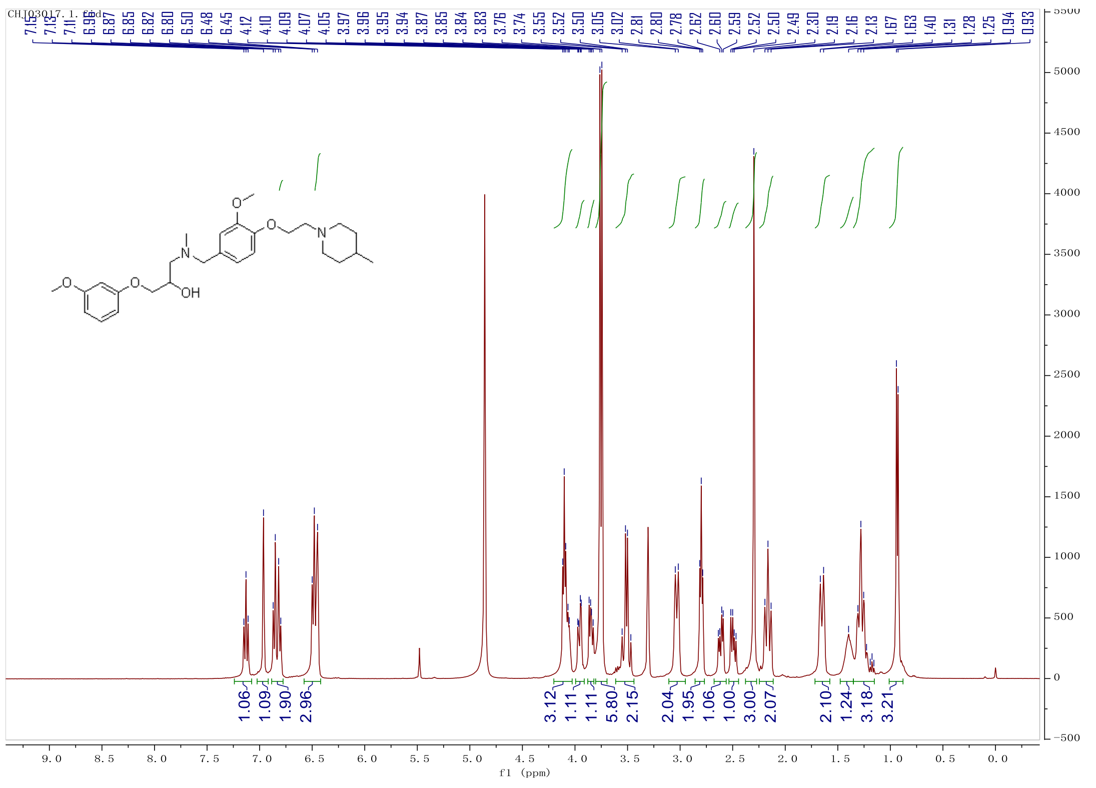

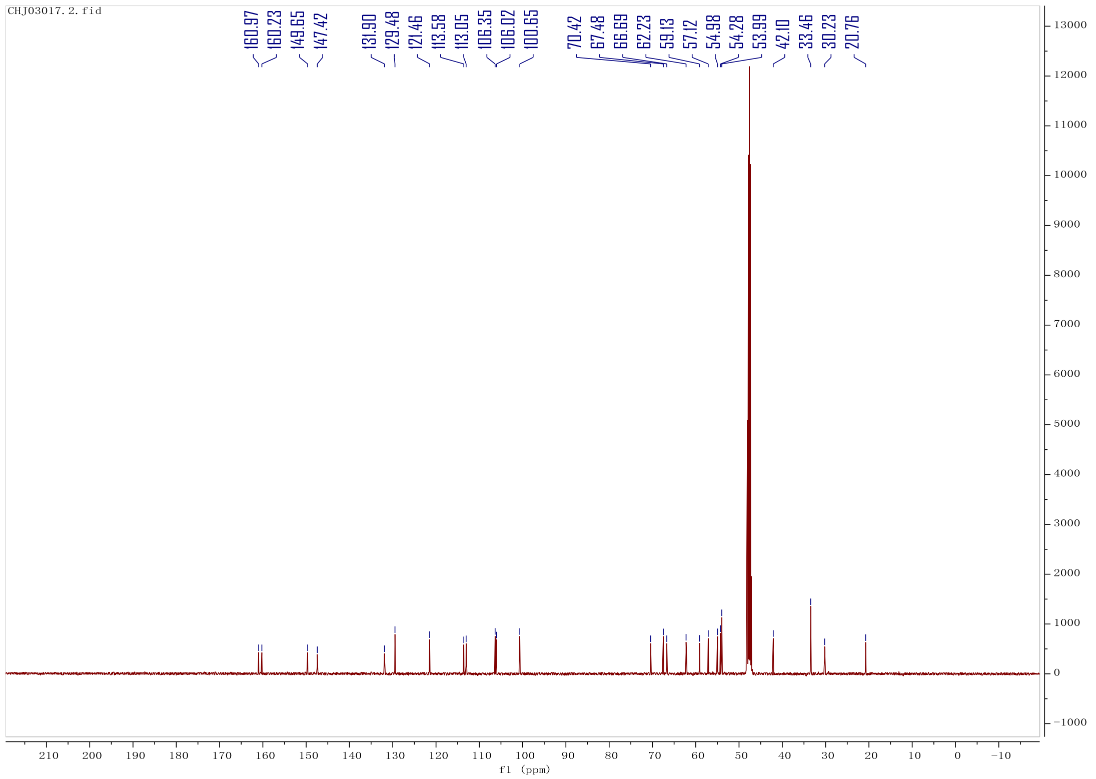
CHJ03017.2.fid
160.97
160.23
149.65
147.42
131.90
129.48
121.46
113.58
113.05
106.35
106.02
100.65
70.42
67.48
66.69
62.23
59.13
57.12
54.98
54.28
53.99
42.10
33.46
30.23
20.76
f1 (ppm)

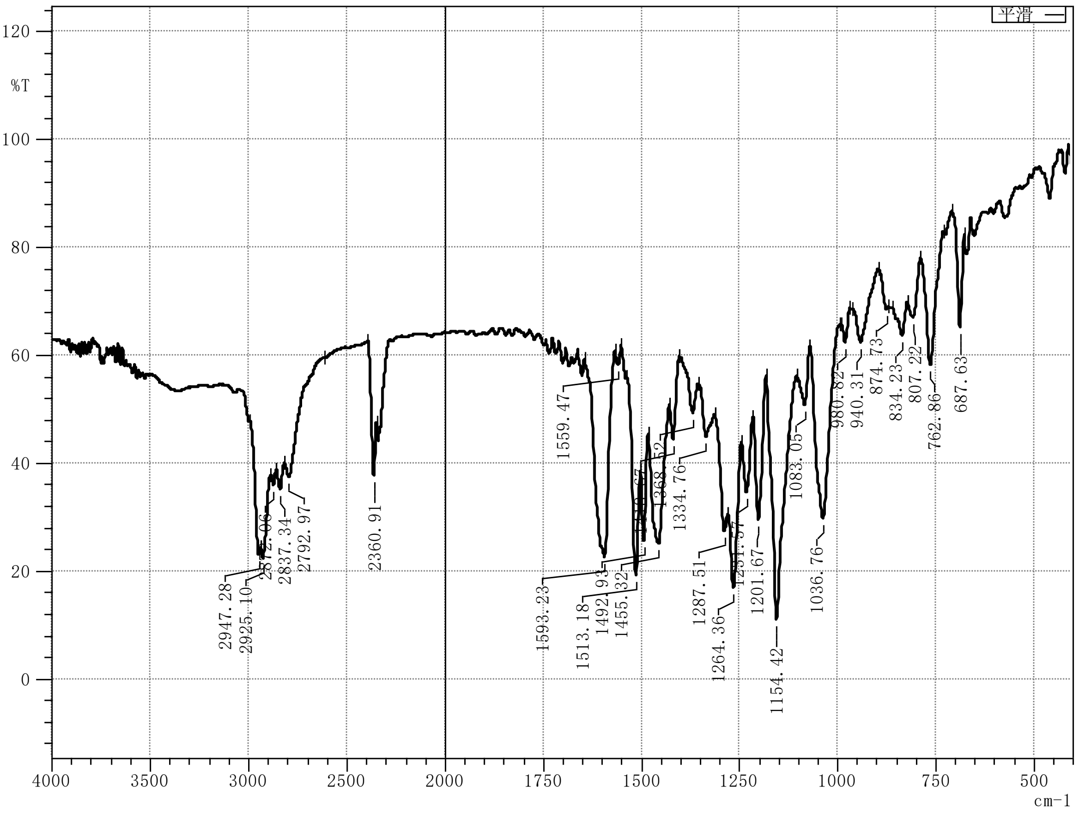
%T
cm-1
2947.28
2925.10
2837.34
2792.97
2360.91
1593.23
1559.47
1513.18
1492.93
1455.32
1334.76
1287.51
1264.36
1201.67
1154.42
1083.05
1036.76
980.82
940.31
874.73
834.23
807.22
762.86
687.63

NMR (^{1}H and ^{13}C) and IR (KBr) of Compound CHJ03018:

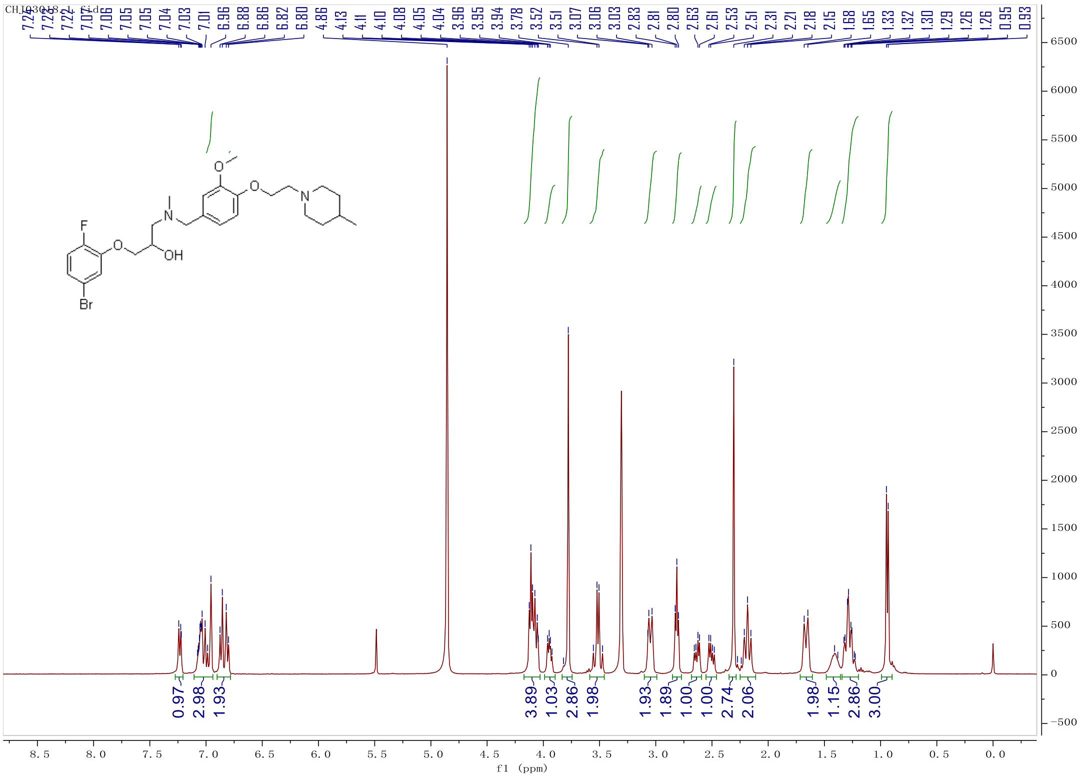

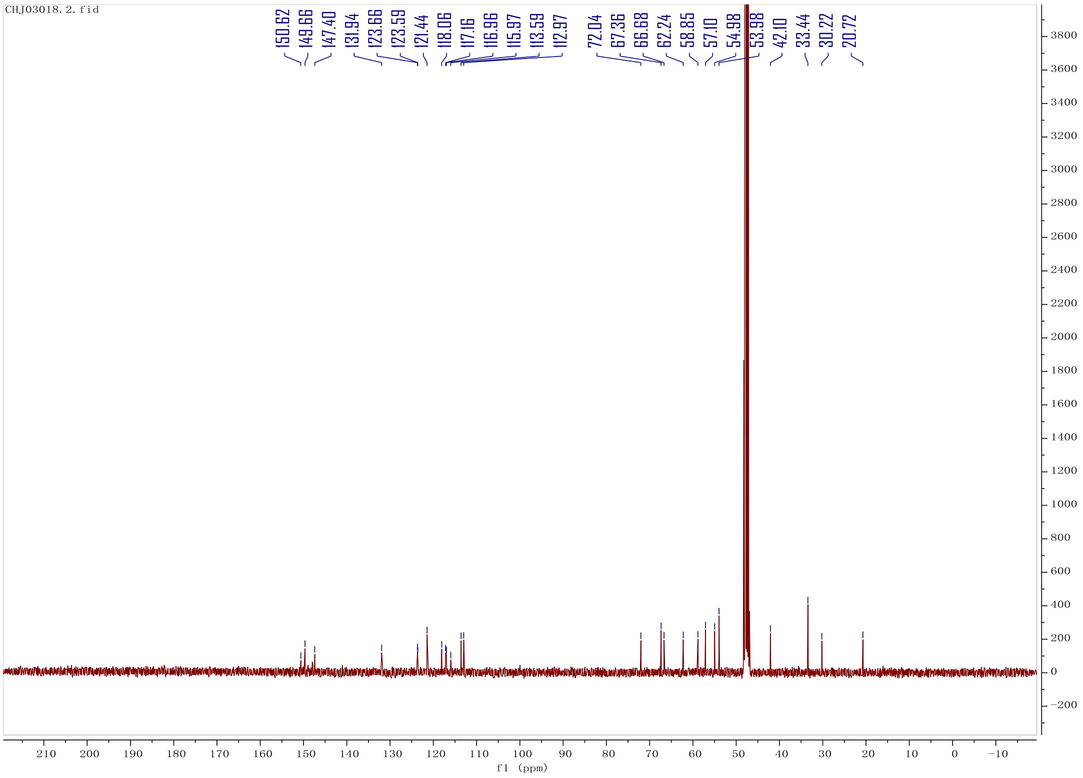

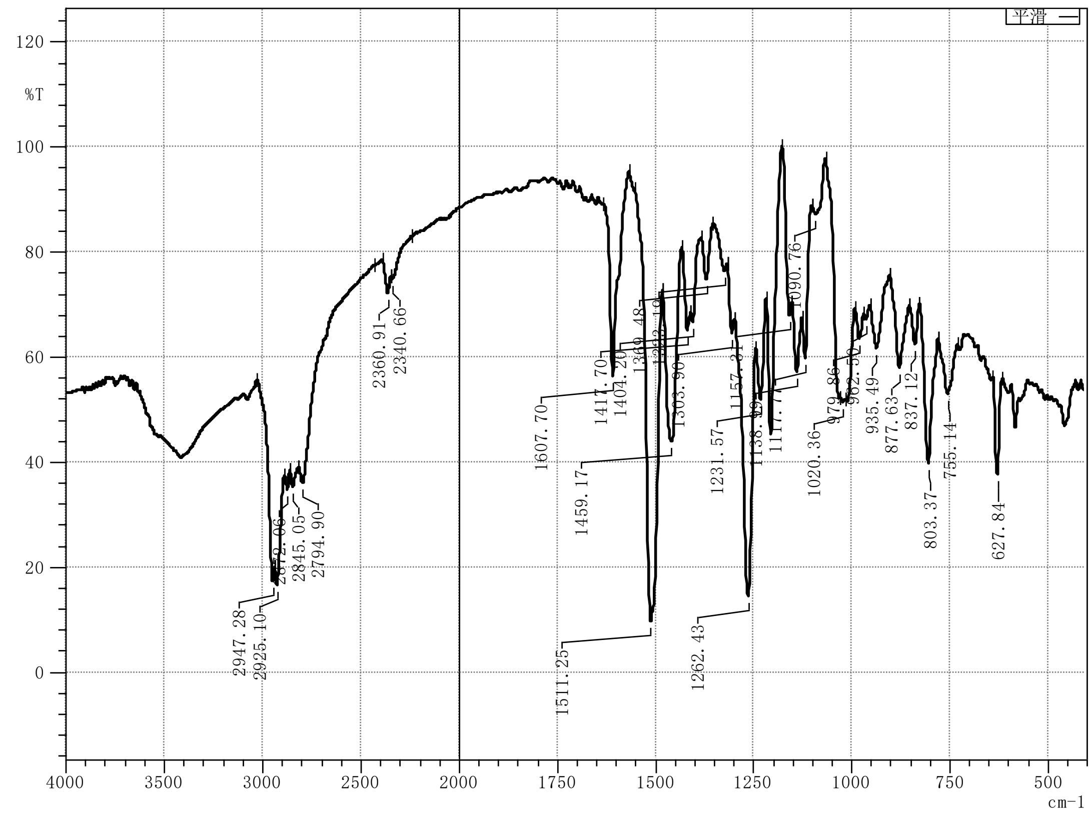

NMR (^{1}H and ^{13}C) and IR (KBr) of Compound CHJ03043:

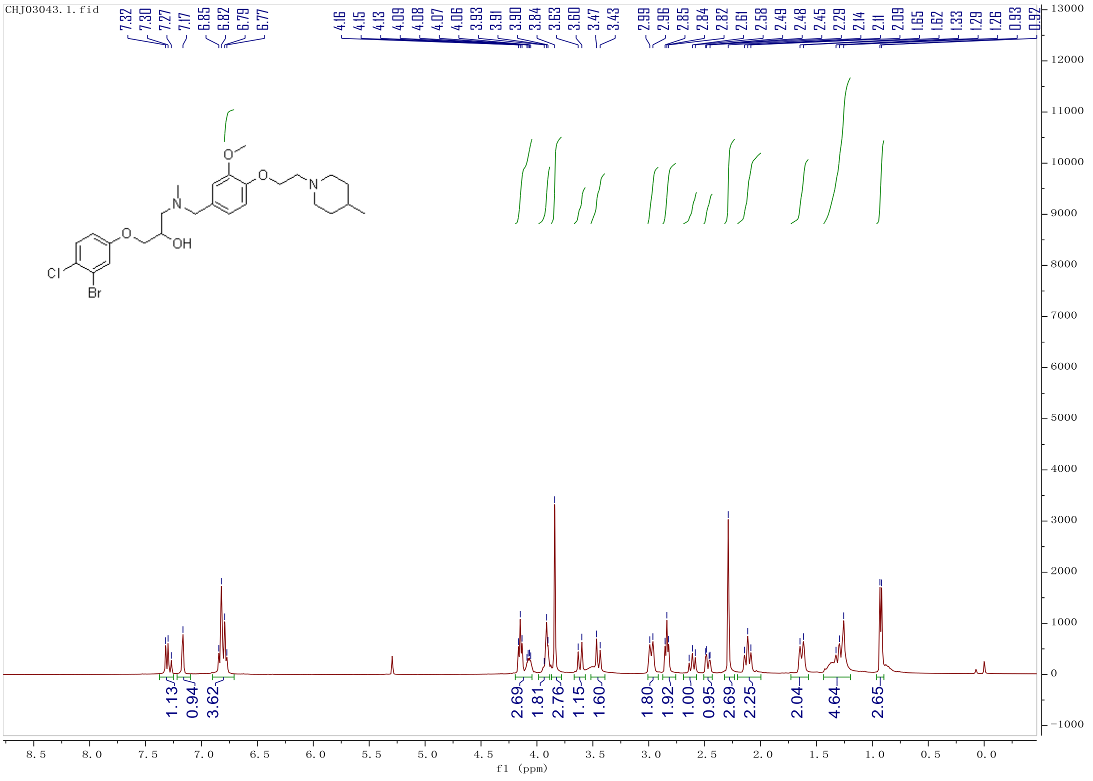

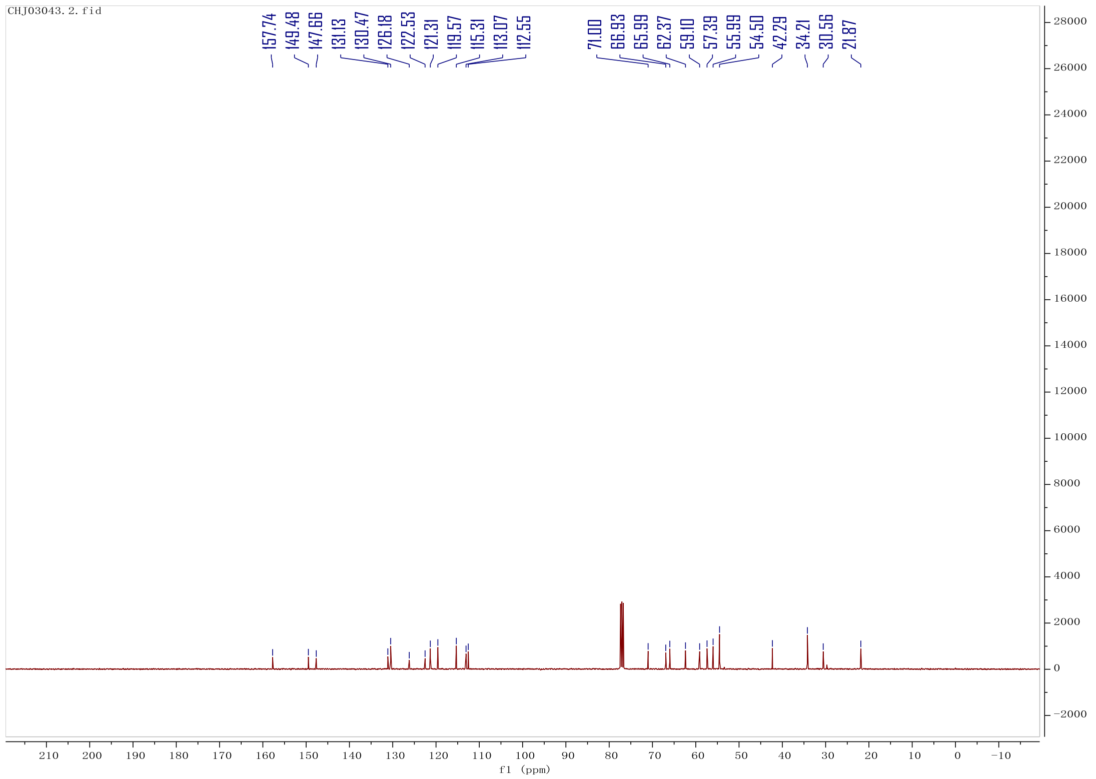

CHJ03043.2.fid
157.74
149.48
147.66
131.13
130.47
126.18
122.53
121.31
119.57
115.31
113.07
112.55
71.00
66.93
65.99
62.37
59.10
57.39
55.99
54.50
42.29
34.21
30.56
21.87
f1 (ppm)

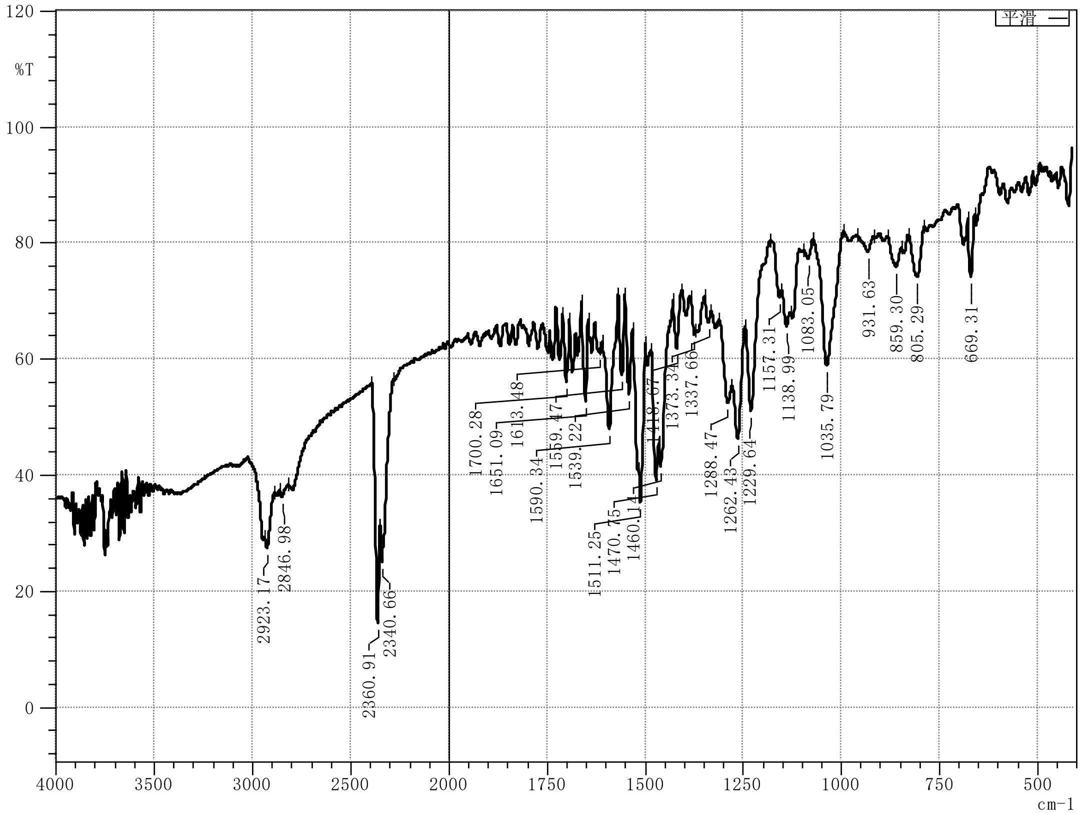

%T
平滑
2923.17
2846.98
2360.91
2340.66
1700.28
1651.09
1613.48
1590.34
1559.47
1539.22
1511.25
1470.75
1460.14
1418.67
1373.34
1337.66
1288.47
1262.43
1229.64
1157.31
1138.99
1083.05
1035.79
931.63
859.30
805.29
669.31
cm-1

NMR (^{1}H and ^{13}C) and IR (KBr) of Compound CHJ04010:

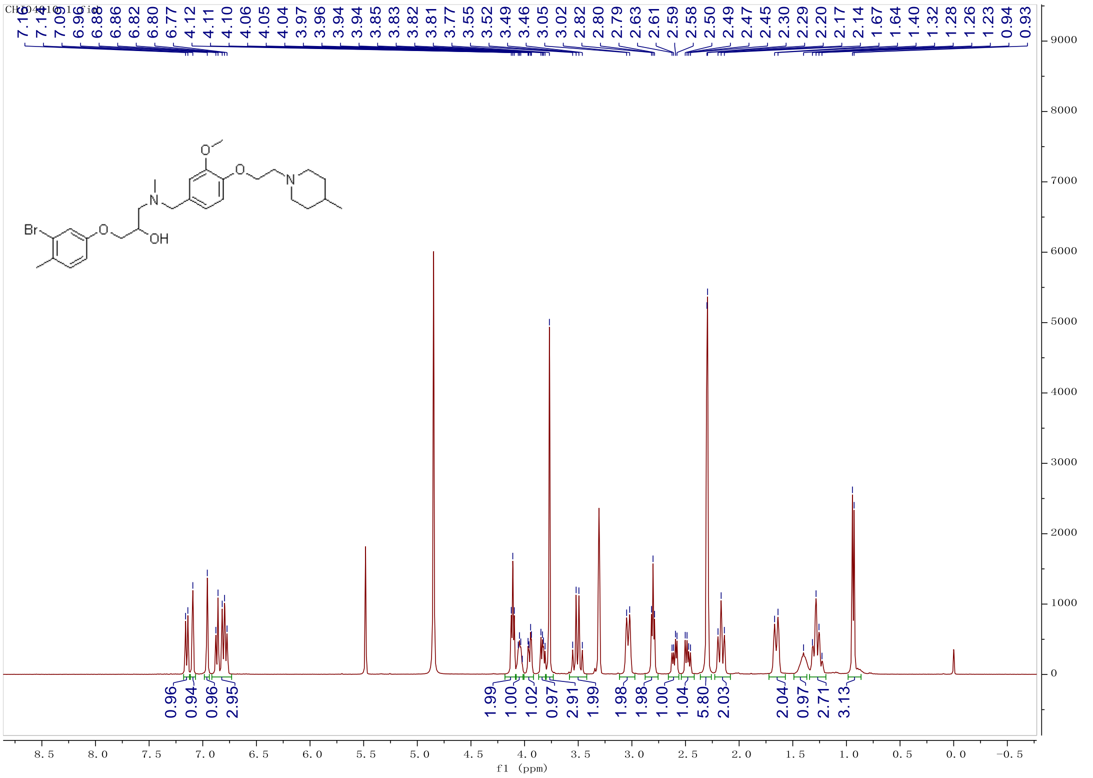

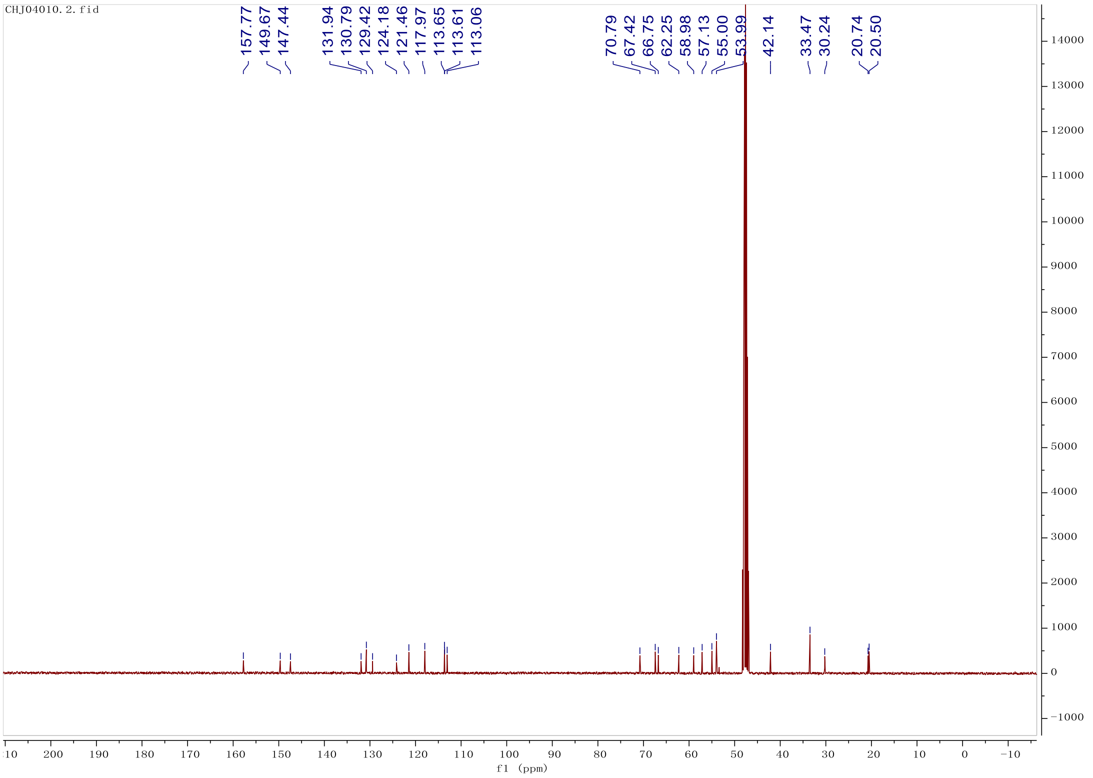

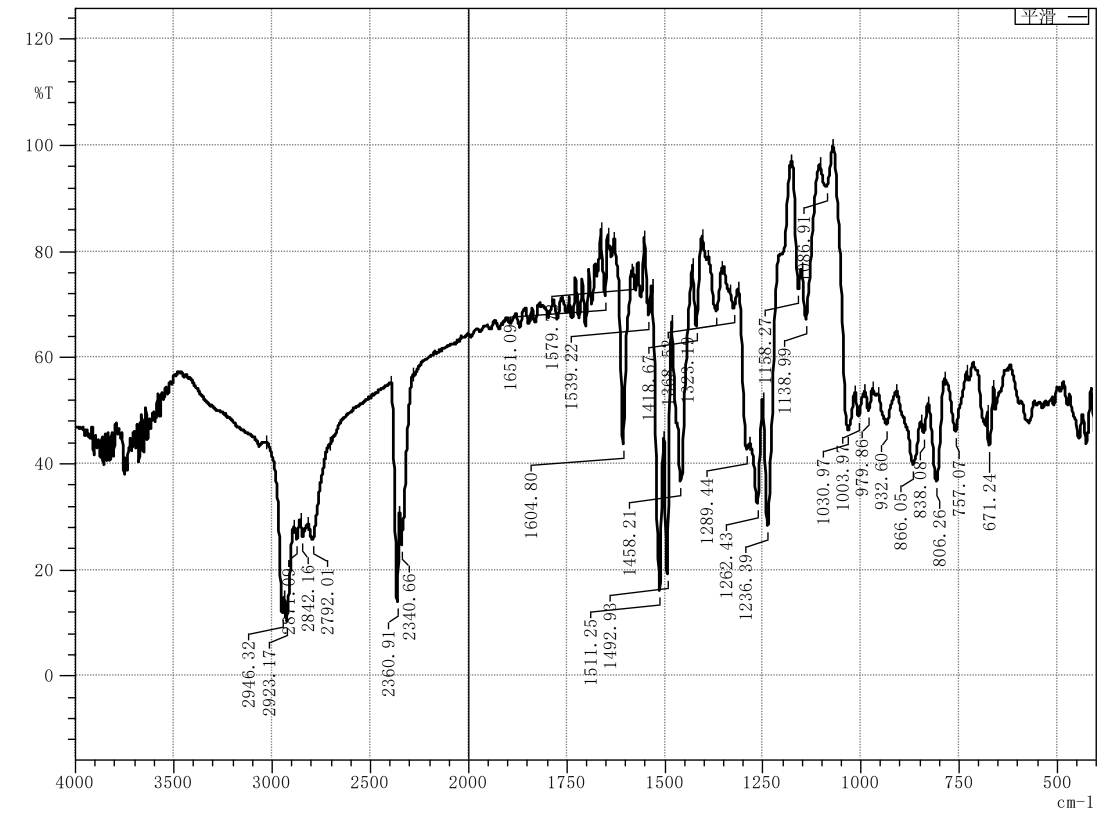

NMR (^{1}H and ^{13}C) and IR (KBr) of Compound CHJ04011:

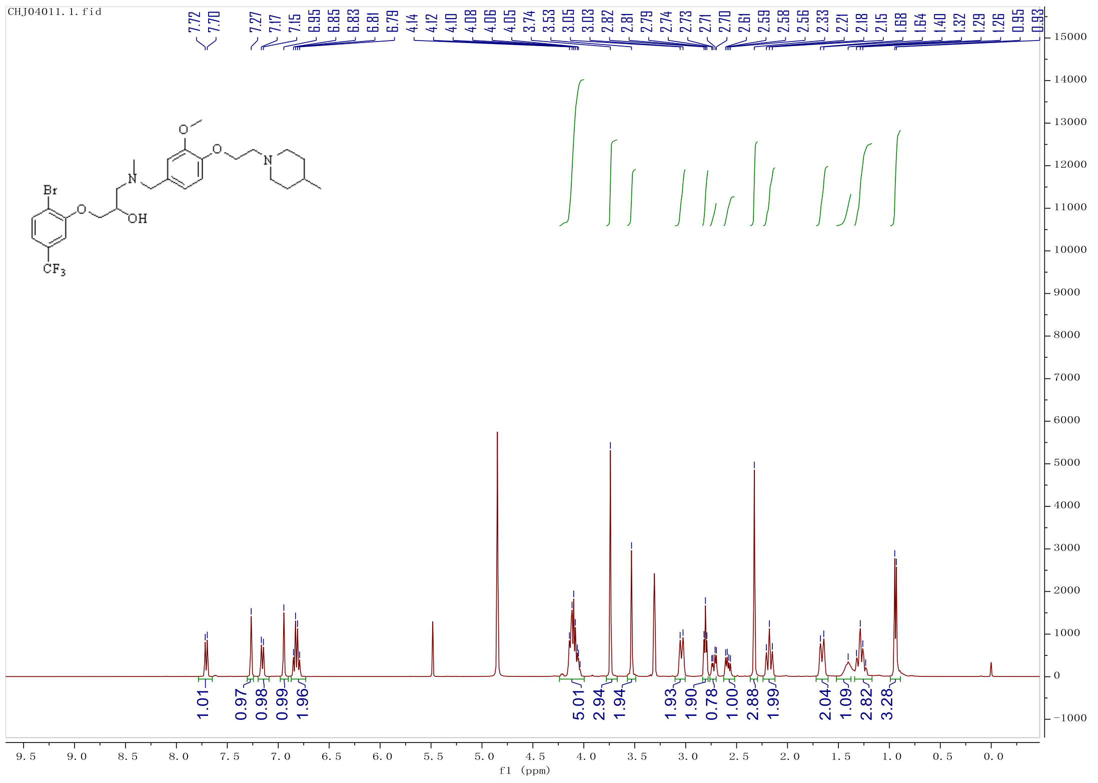

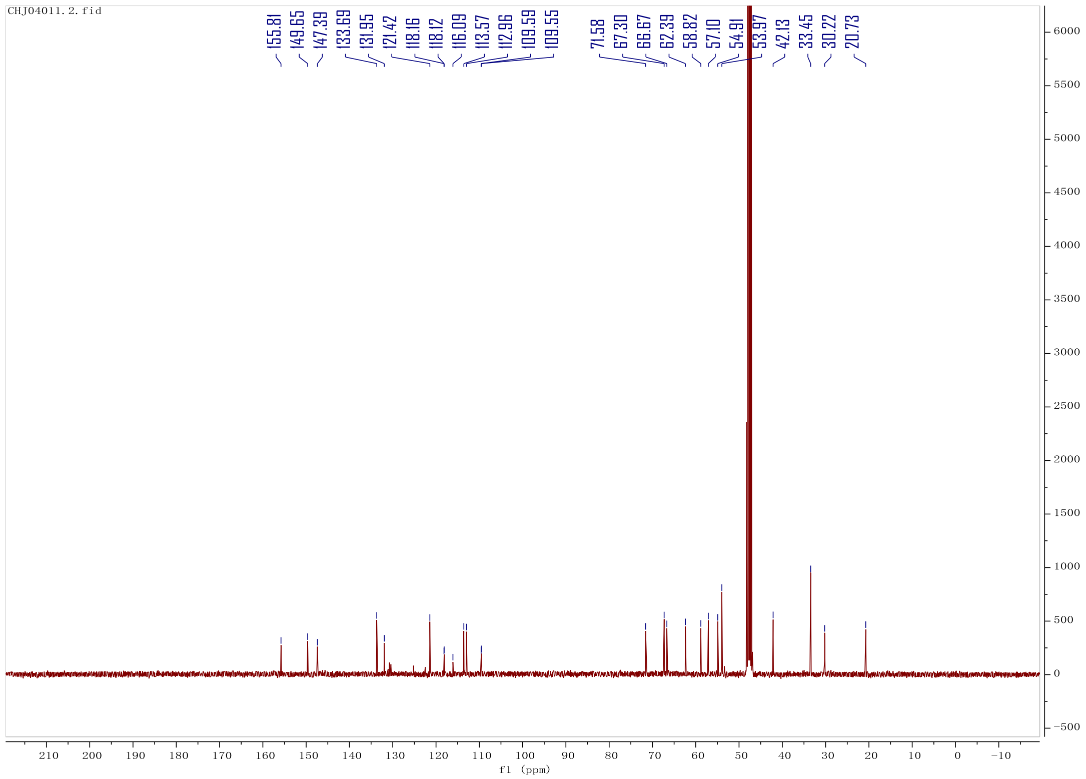
CHJ04011.2.fid
155.81
149.65
147.39
133.69
131.95
121.42
118.16
118.12
116.09
113.57
112.96
109.59
109.55
71.58
67.30
66.67
62.39
58.82
57.10
54.91
53.97
42.13
33.45
30.22
20.73
f1 (ppm)

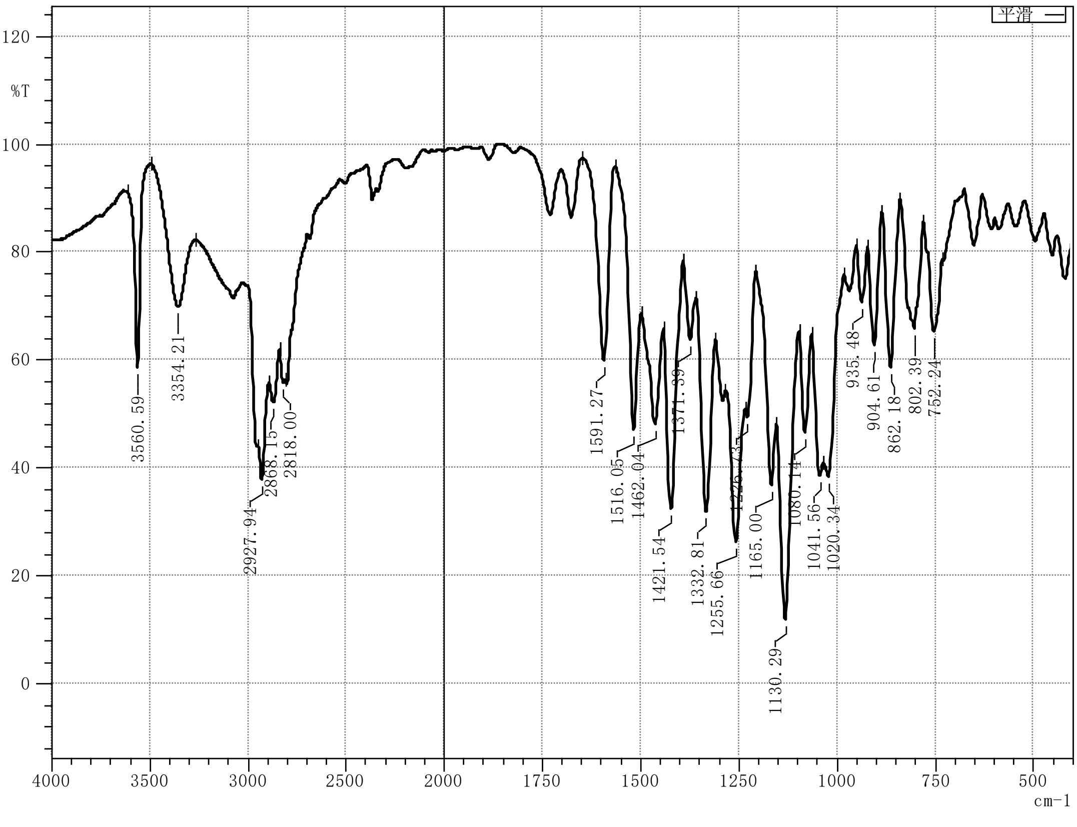
%T
cm-1
3560.59
3354.21
2927.94
2868.15
2818.00
1591.27
1516.05
1462.04
1421.54
1371.39
1332.81
1255.66
1165.00
1130.29
1080.14
1041.56
1020.34
935.48
904.61
862.18
802.39
752.24

NMR (^{1}H and ^{13}C) and IR (KBr) of Compound CHJ04012:

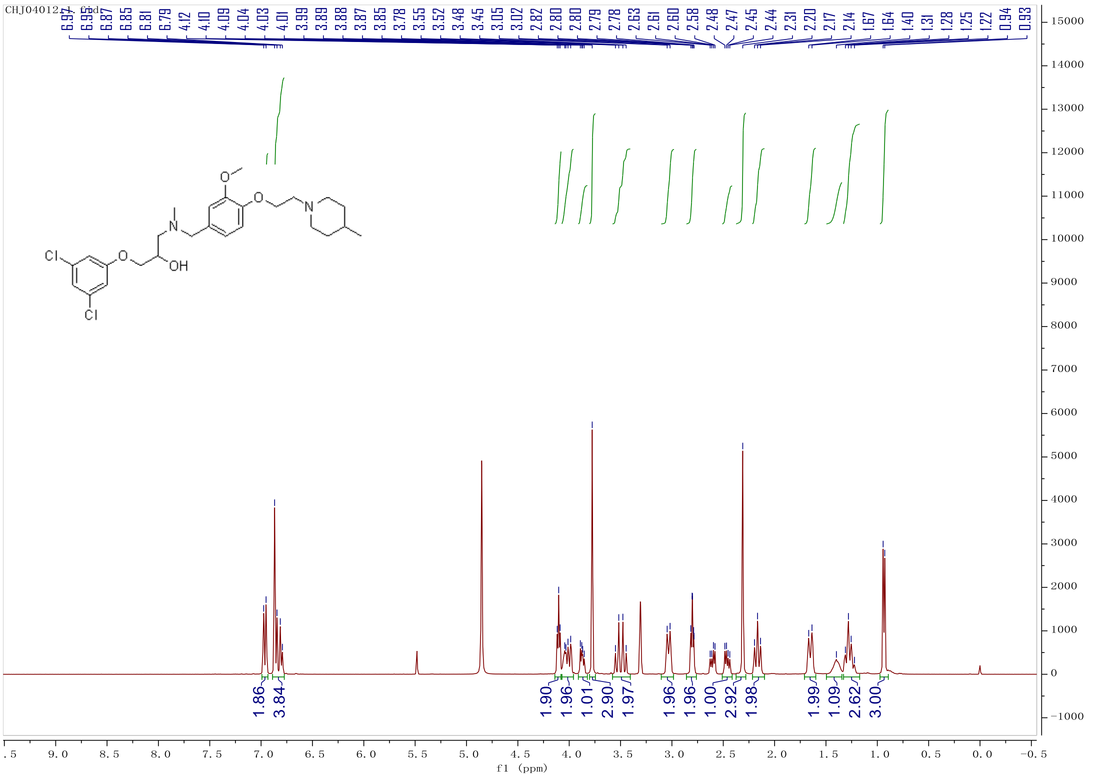

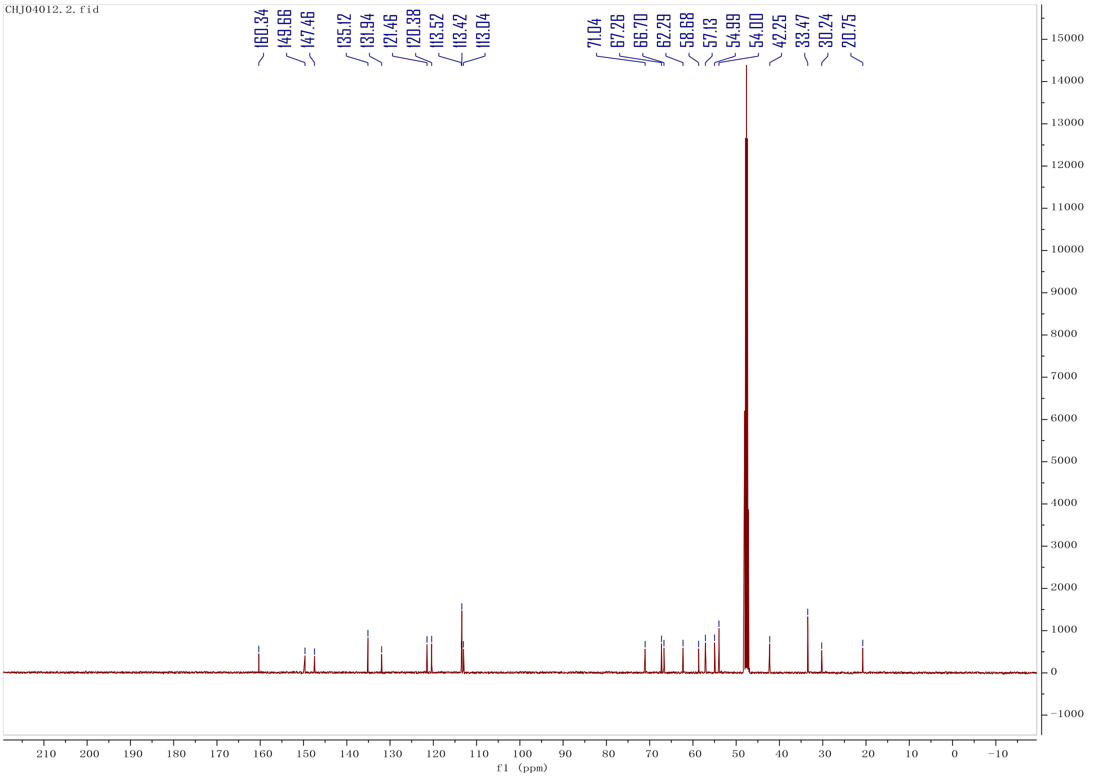

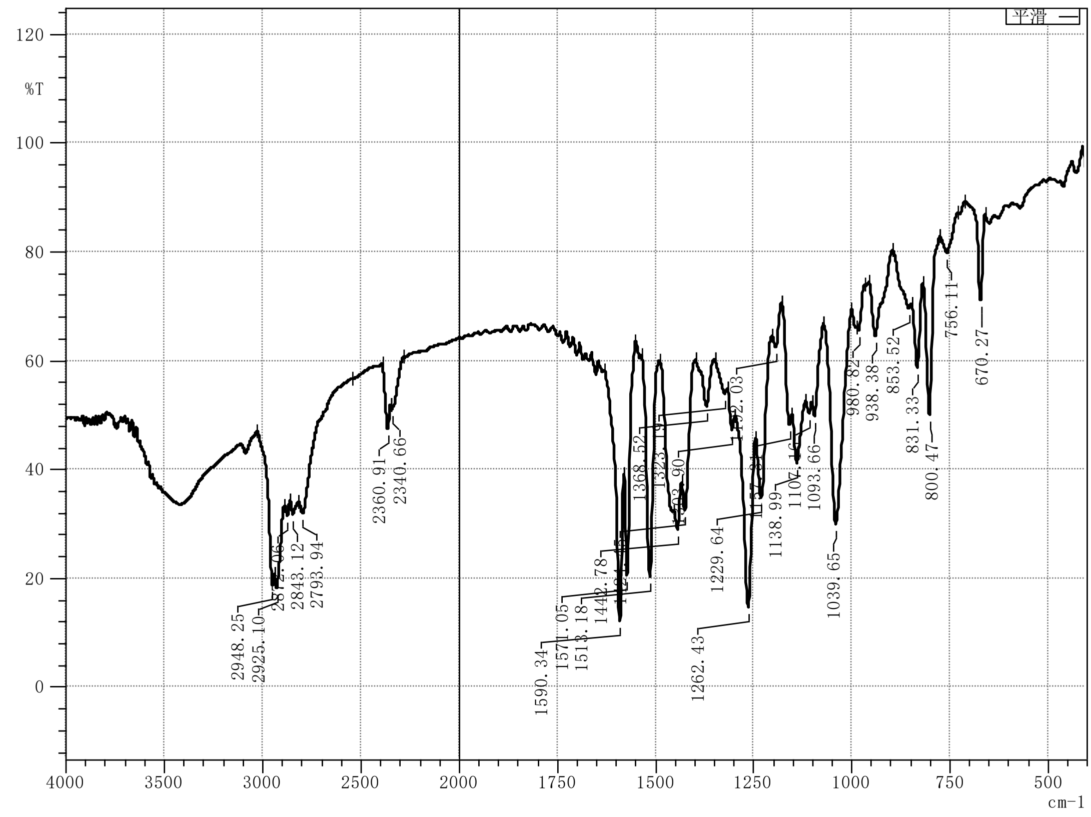

NMR (^{1}H and ^{13}C) and IR (KBr) of Compound CHJ04020:

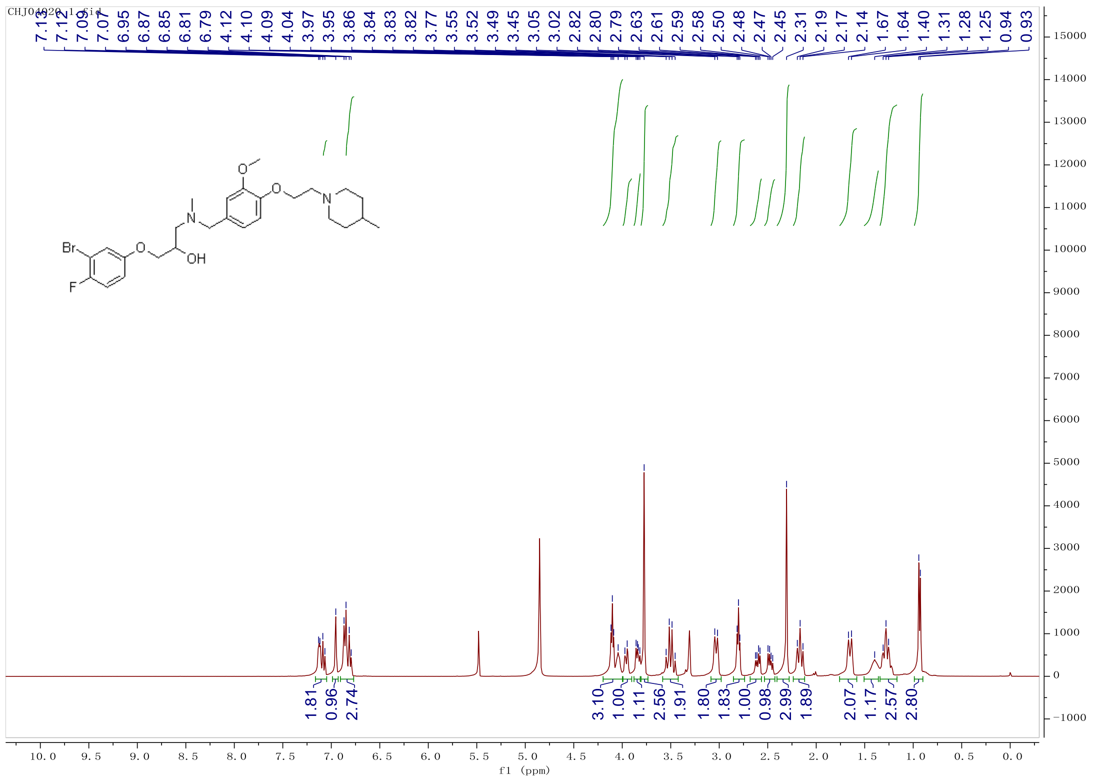

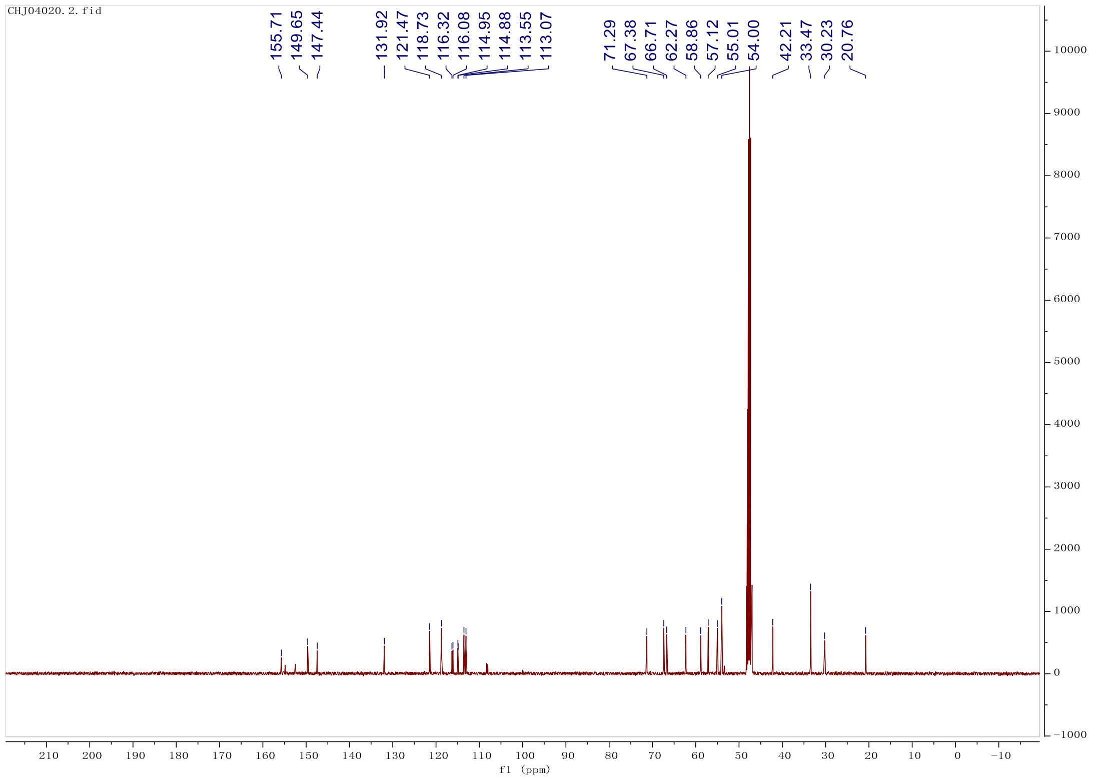
CHJ04020.2.fid
155.71
149.65
147.44
131.92
121.47
118.73
116.32
116.08
114.95
114.88
113.55
113.07
71.29
67.38
66.71
62.27
58.86
57.12
55.01
54.00
42.21
33.47
30.23
20.76
f1 (ppm)

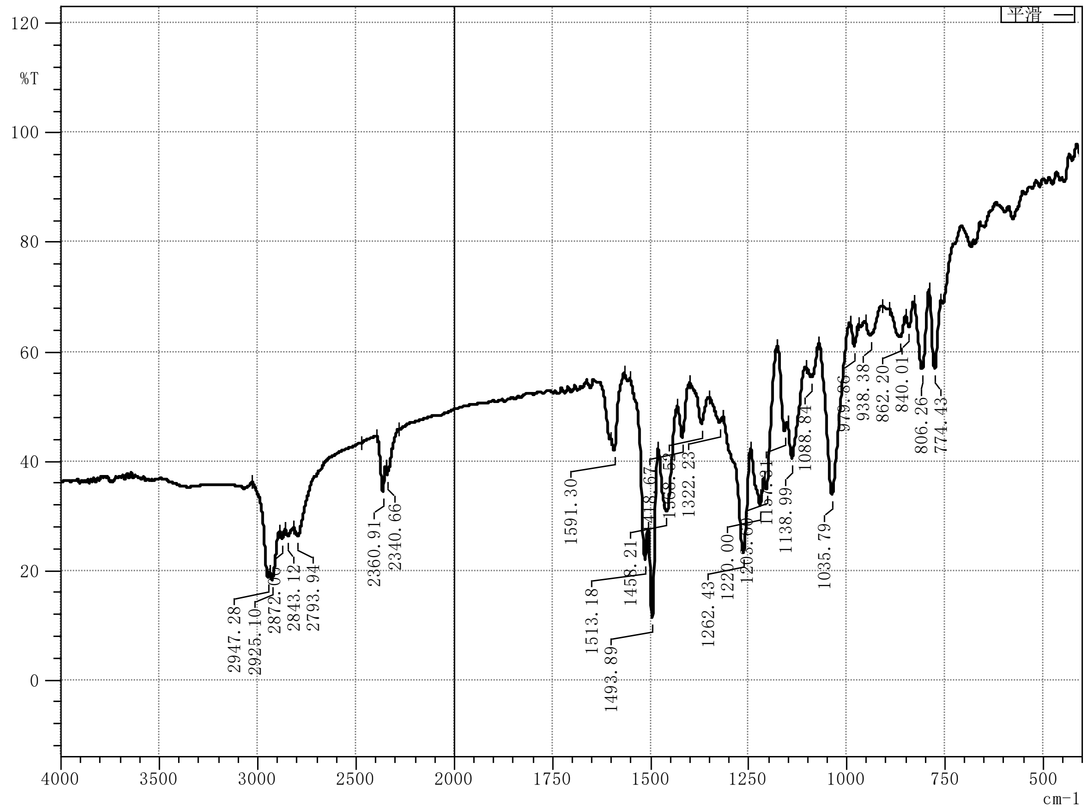
%T
cm-1
2947.28
2925.10
2843.12
2793.94
2360.91
2340.66
1591.30
1513.18
1493.89
1322.23
1262.43
1220.00
1138.99
1088.84
1035.79
938.38
862.20
840.01
806.26
774.43

NMR (^{1}H and ^{13}C) and IR (KBr) of Compound CHJ04022:

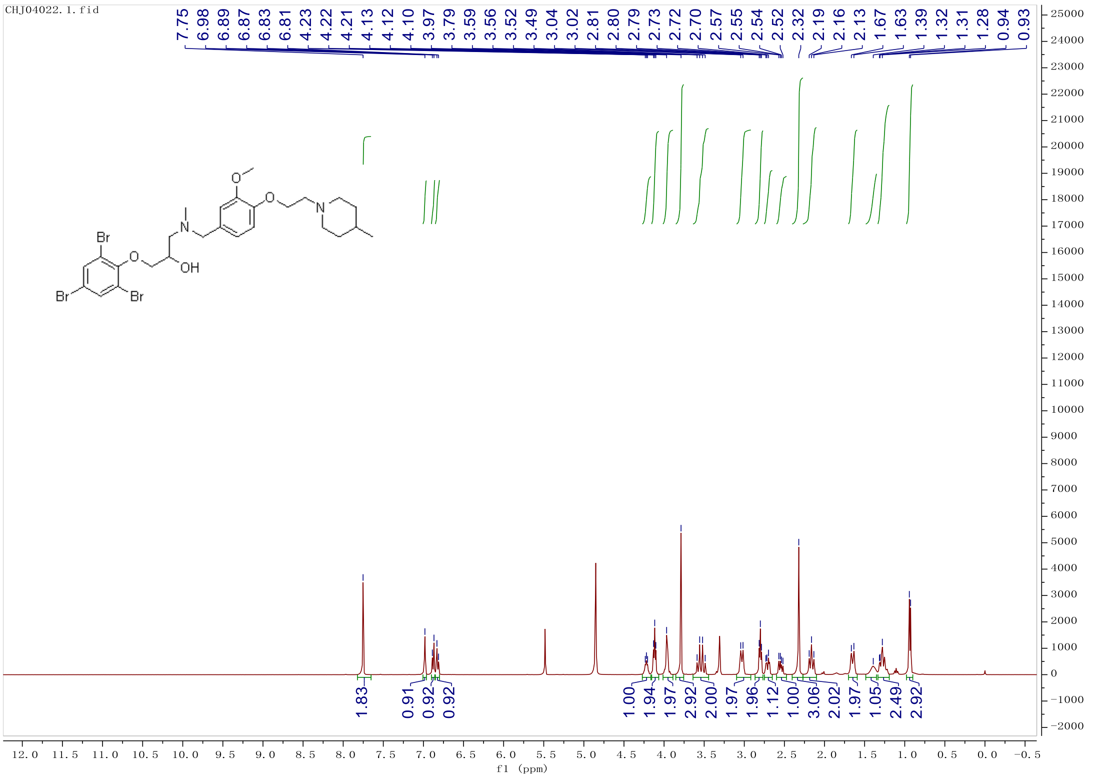

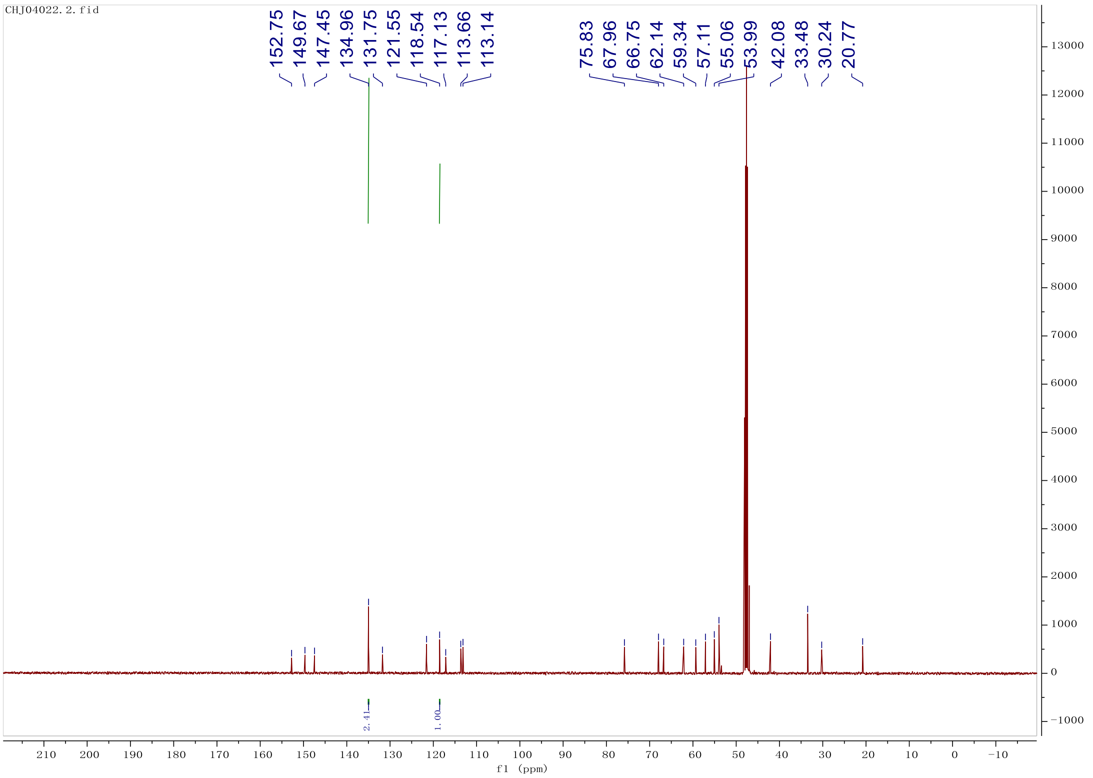

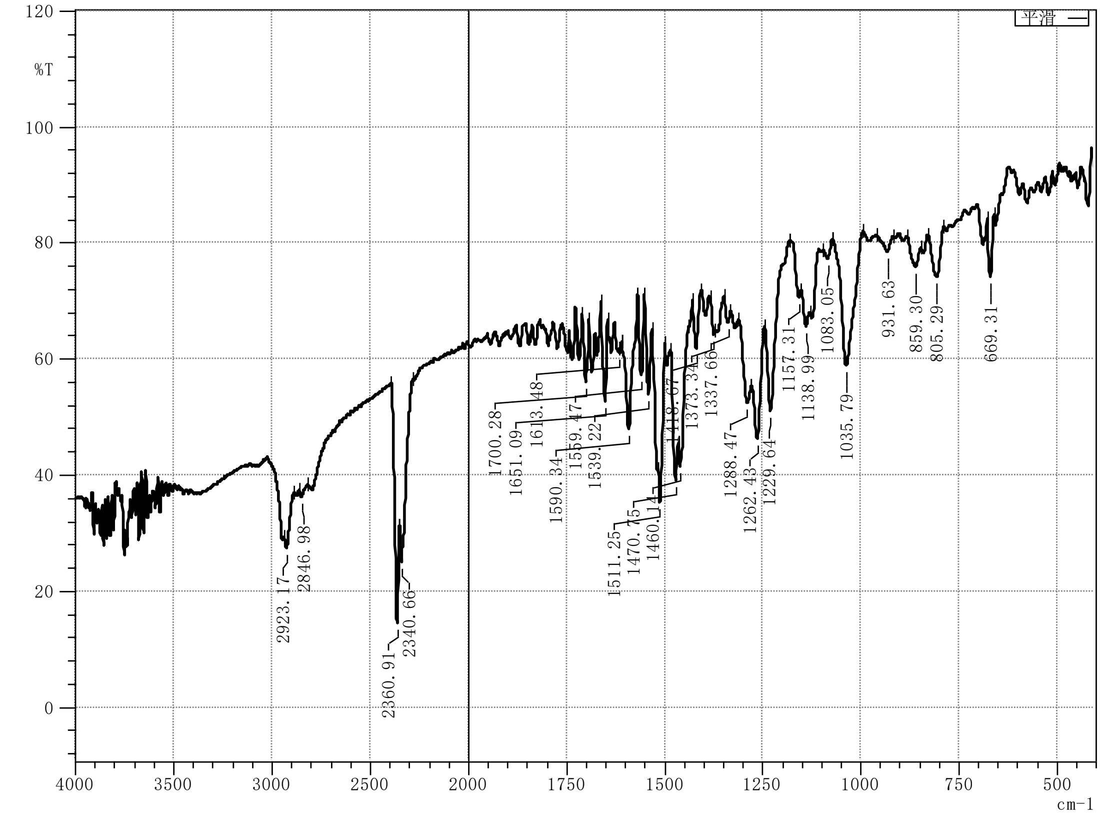

NMR (^{1}H and ^{13}C) and IR (KBr) of Compound CHJ04023:

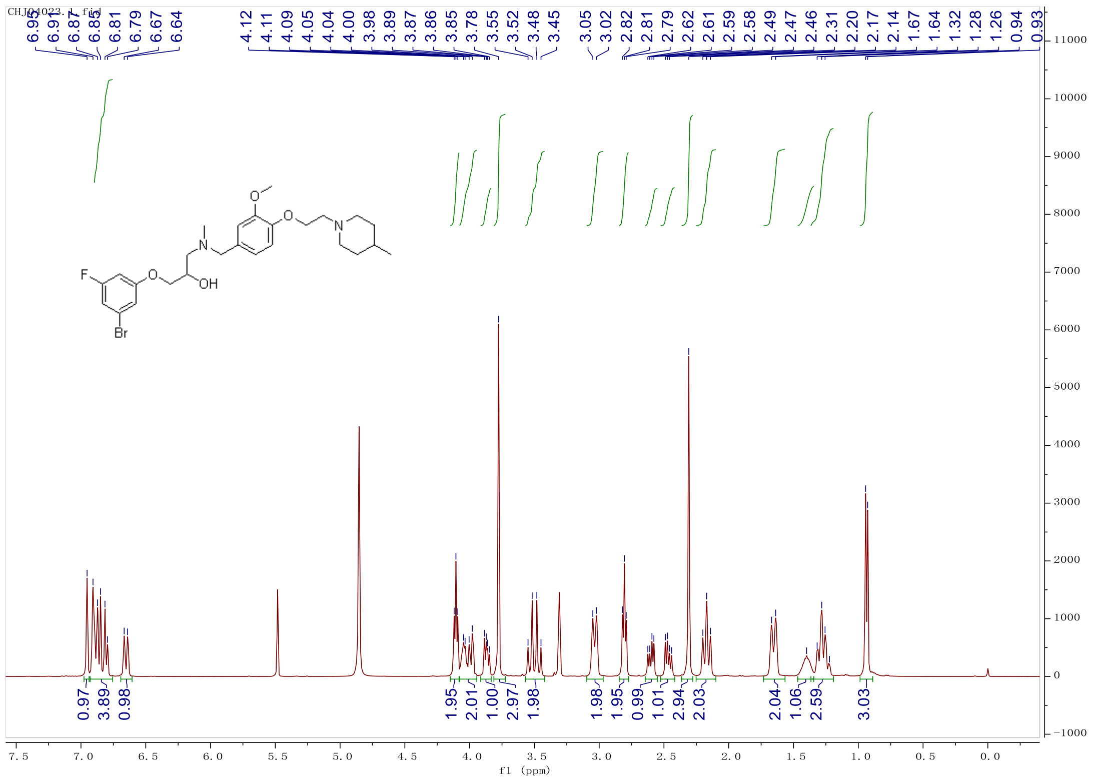

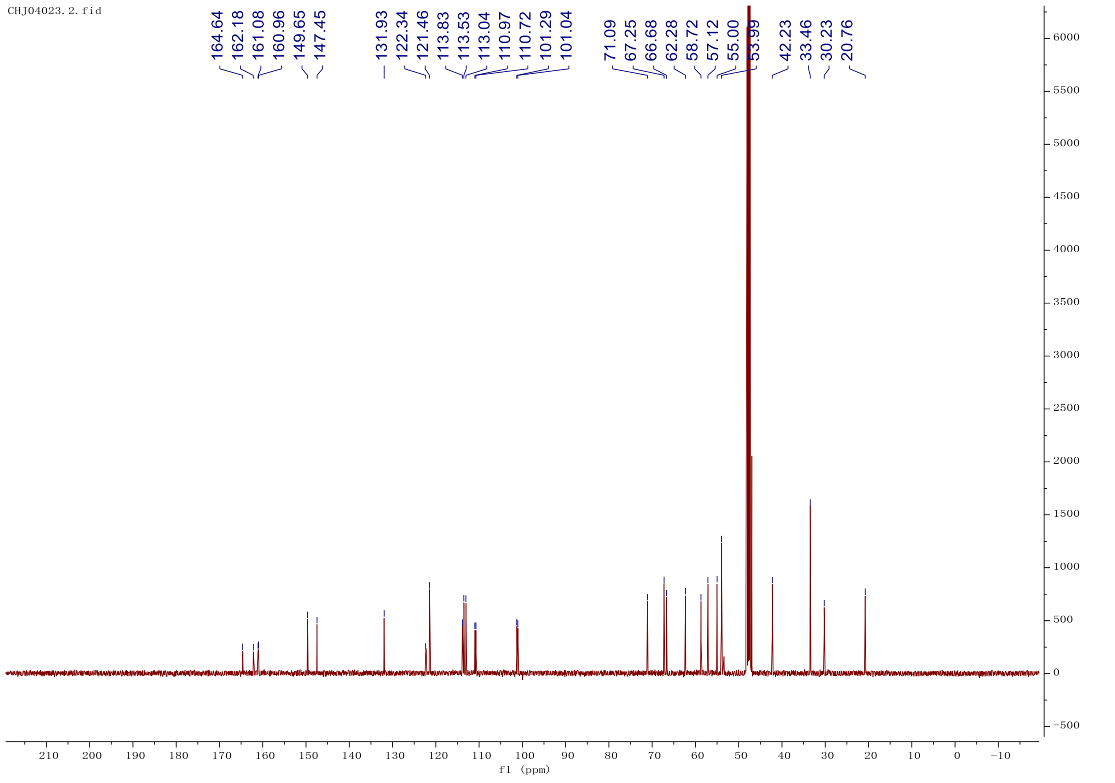
CHJ04023.2.fid
164.64
162.18
161.08
160.96
149.65
147.45
131.93
122.34
121.46
113.83
113.53
113.04
110.97
110.72
101.29
101.04
71.09
67.25
66.68
62.28
58.72
57.12
55.00
53.99
42.23
33.46
30.23
20.76
f1 (ppm)

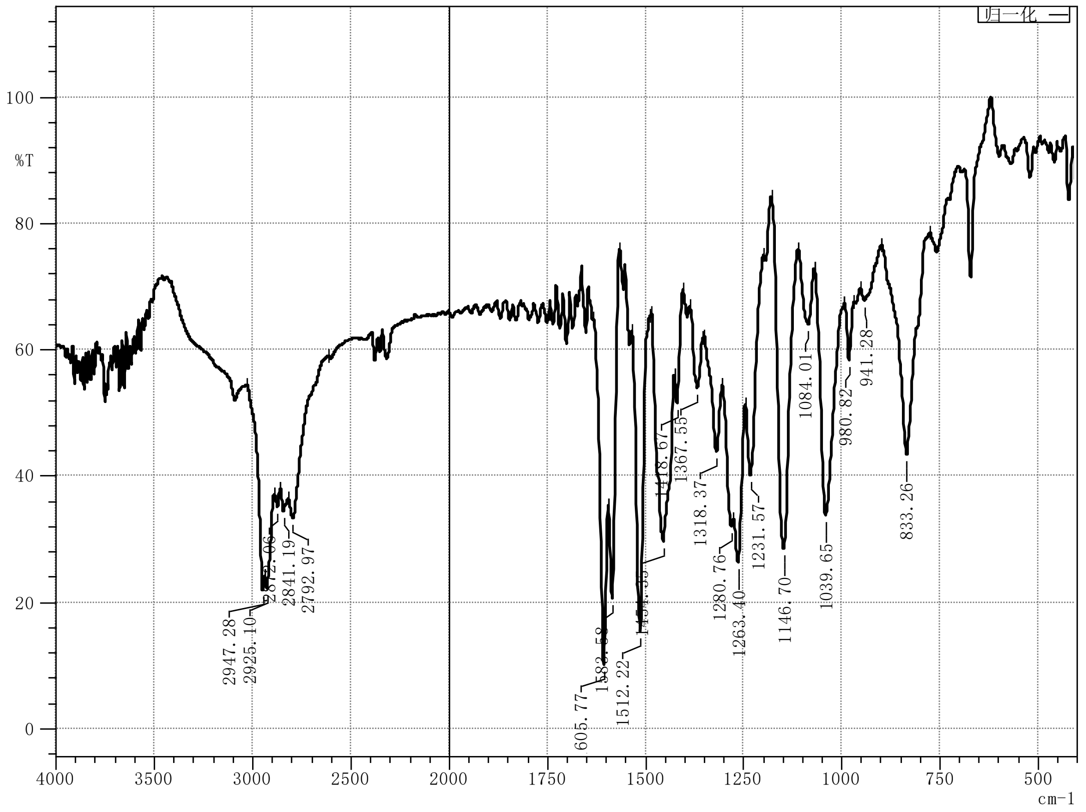
归一化
%T
cm-1
2947.28
2925.10
2841.19
2792.97
605.77
1512.22
1418.67
1367.55
1318.37
1280.76
1263.40
1231.57
1146.70
1084.01
1039.65
980.82
941.28
833.26

NMR (^{1}H and ^{13}C) and IR (KBr) of Compound CHJ04024:

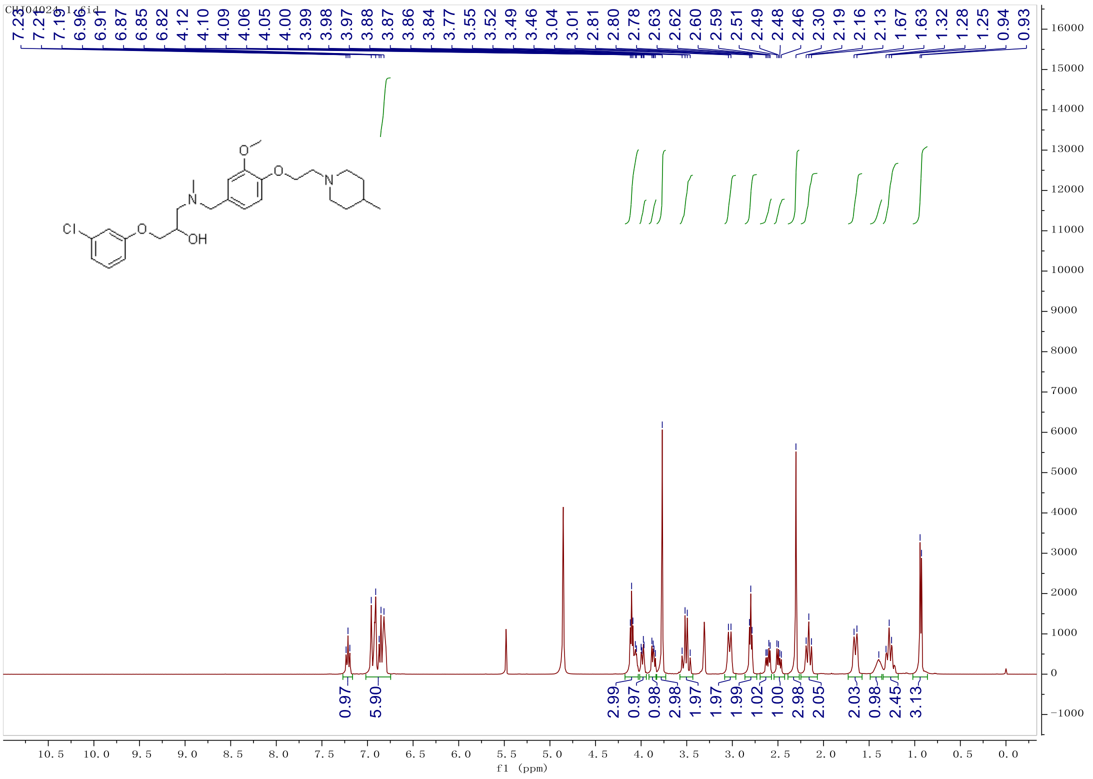

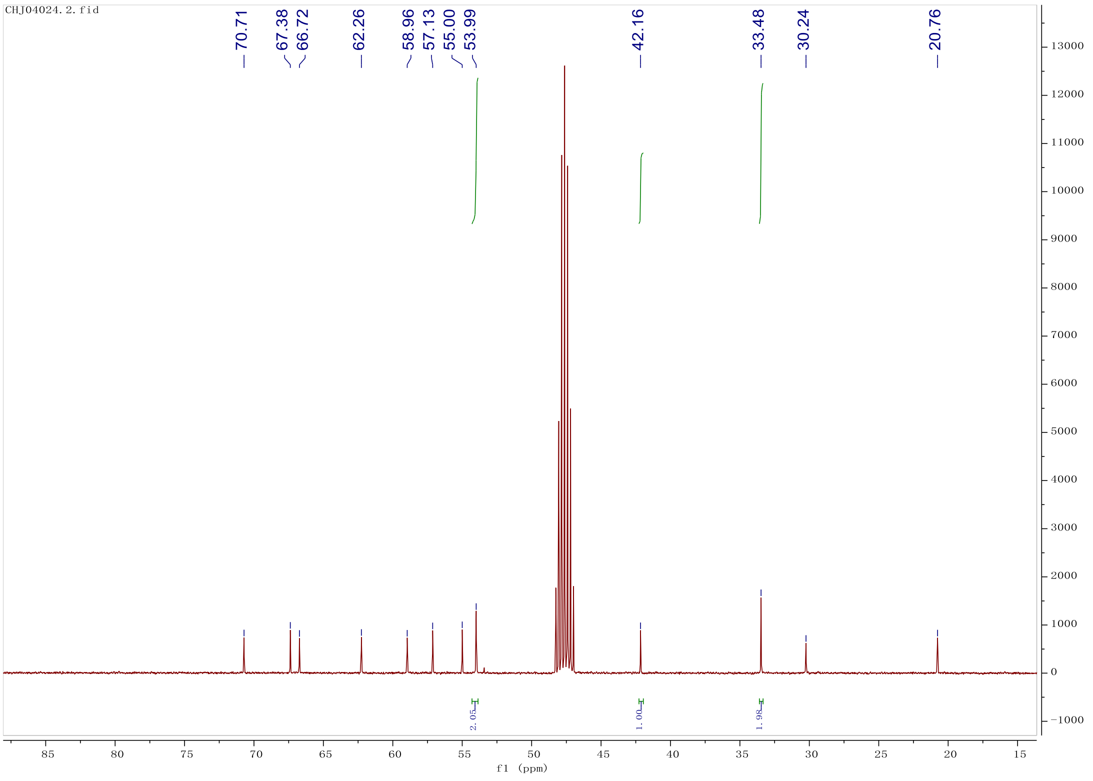

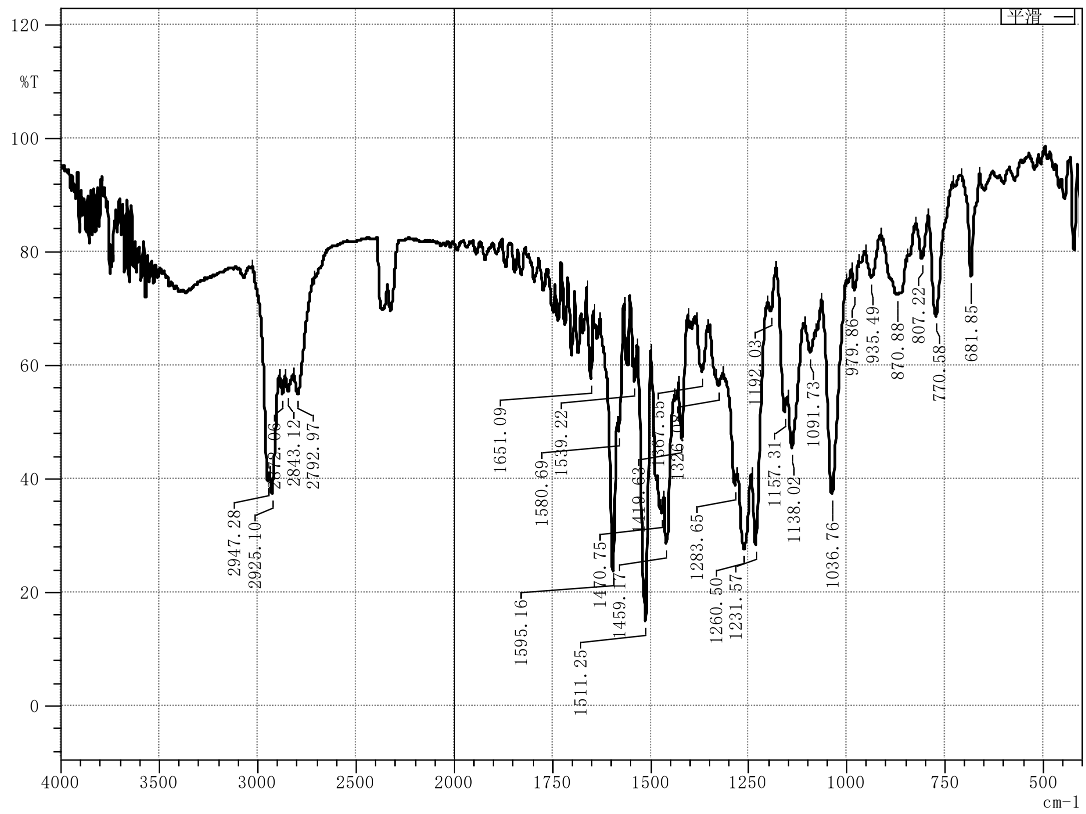

NMR (^{1}H and ^{13}C) and IR (KBr) of Compound CHJ04025:

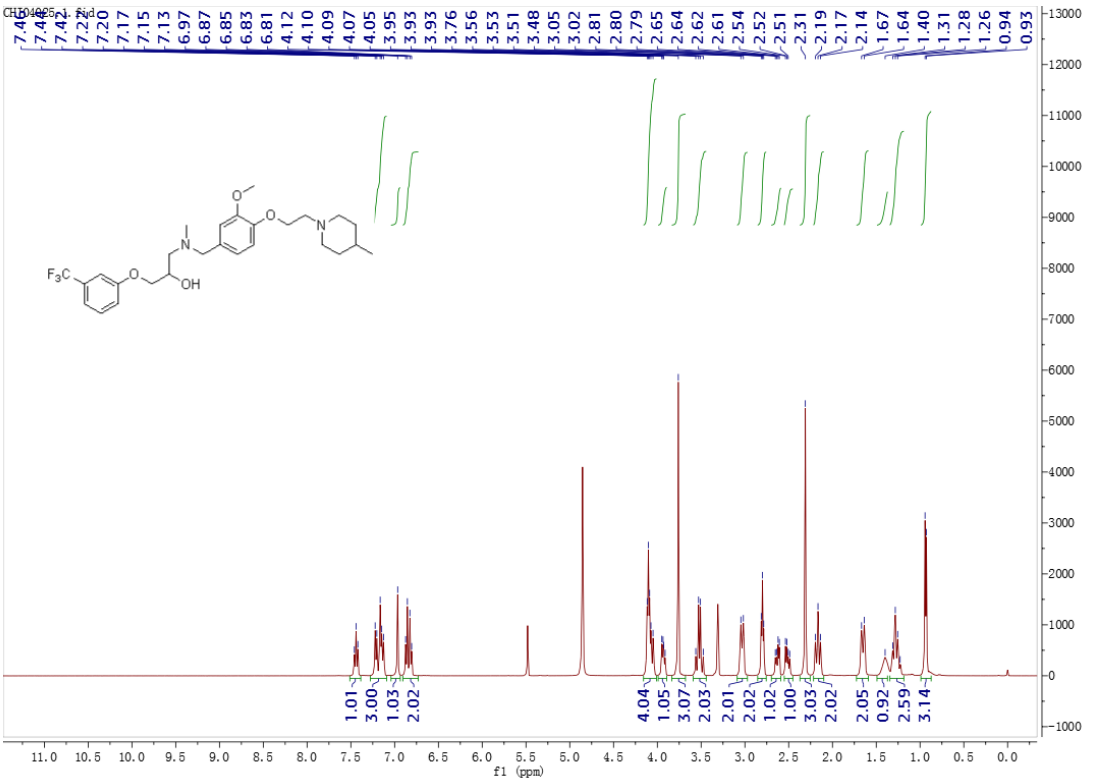

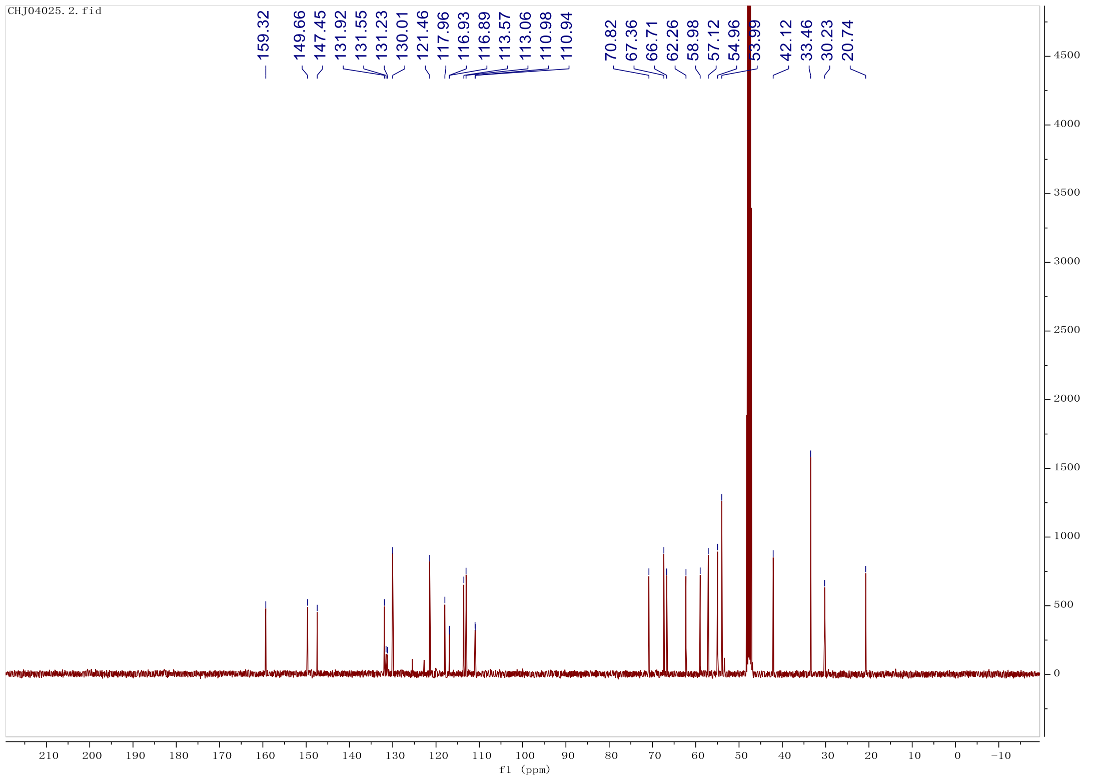
CHJ04025.2.fid
159.32
149.66
147.45
131.92
131.55
131.23
130.01
121.46
117.96
116.93
116.89
113.57
113.06
110.98
110.94
70.82
67.36
66.71
62.26
58.98
57.12
54.96
53.99
42.12
33.46
30.23
20.74
f1 (ppm)

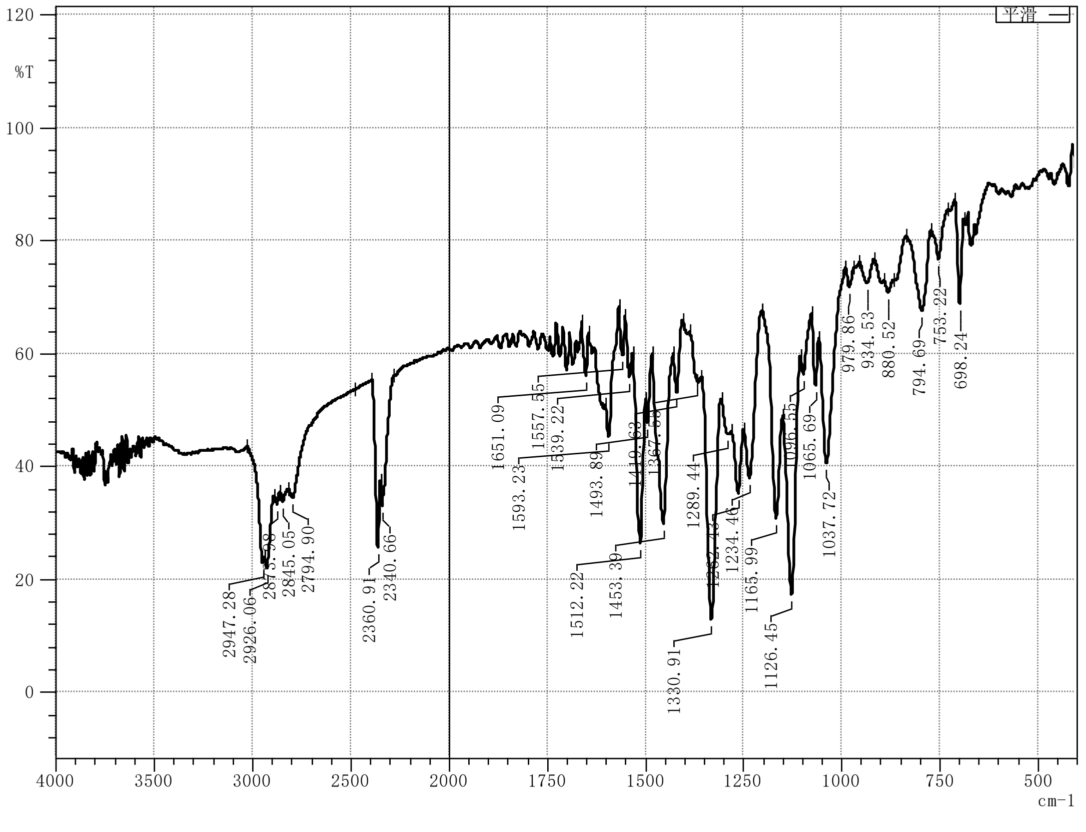
%T
cm-1
2947.28
2926.06
2845.05
2794.90
2360.91
2340.66
1651.09
1593.23
1557.55
1539.22
1512.22
1493.89
1453.39
1289.44
1330.91
1234.46
1165.99
1126.45
1065.69
1037.72
979.86
934.53
880.52
794.69
753.22
698.24

NMR (^{1}H and ^{13}C) and IR (KBr) of Compound CHJ04026:

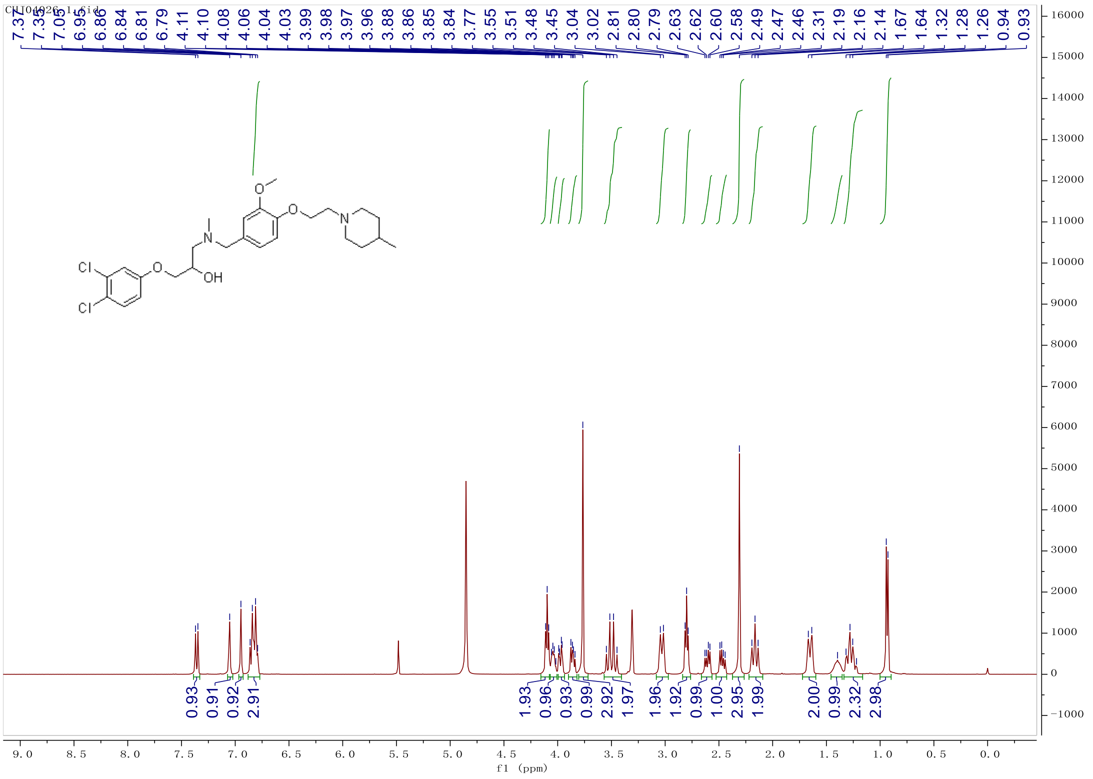

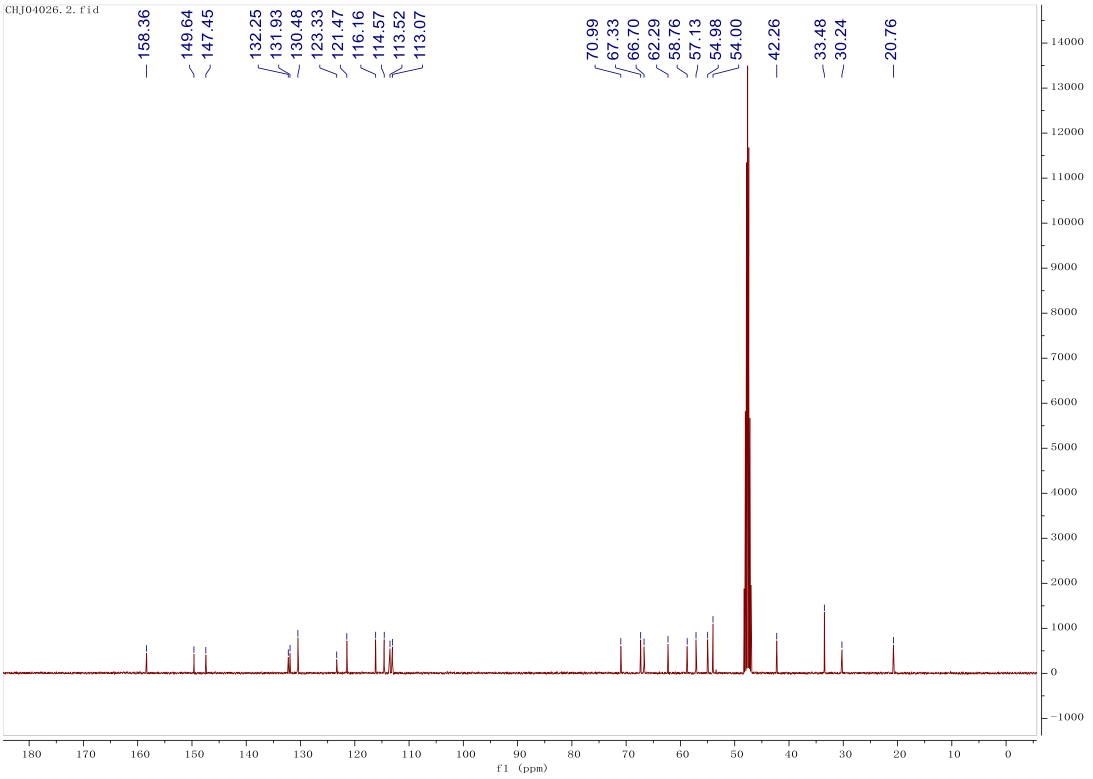

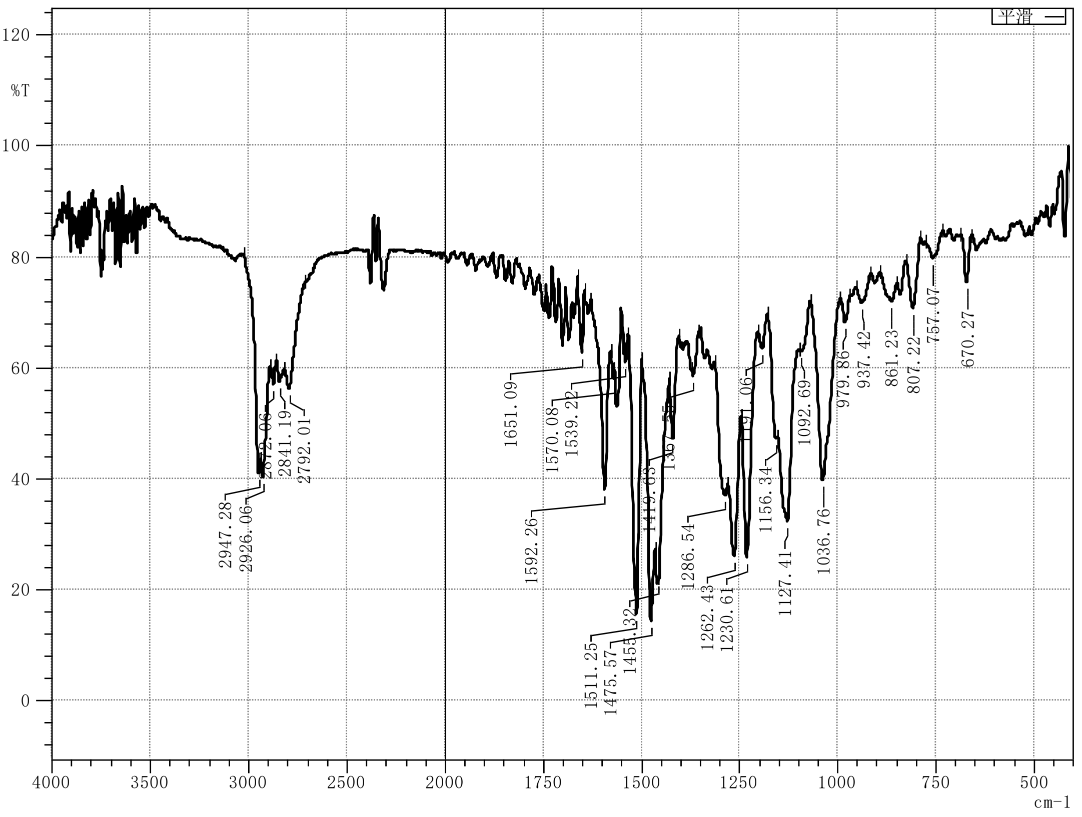

NMR (^{1}H and ^{13}C) and IR (KBr) of Compound CHJ04027:

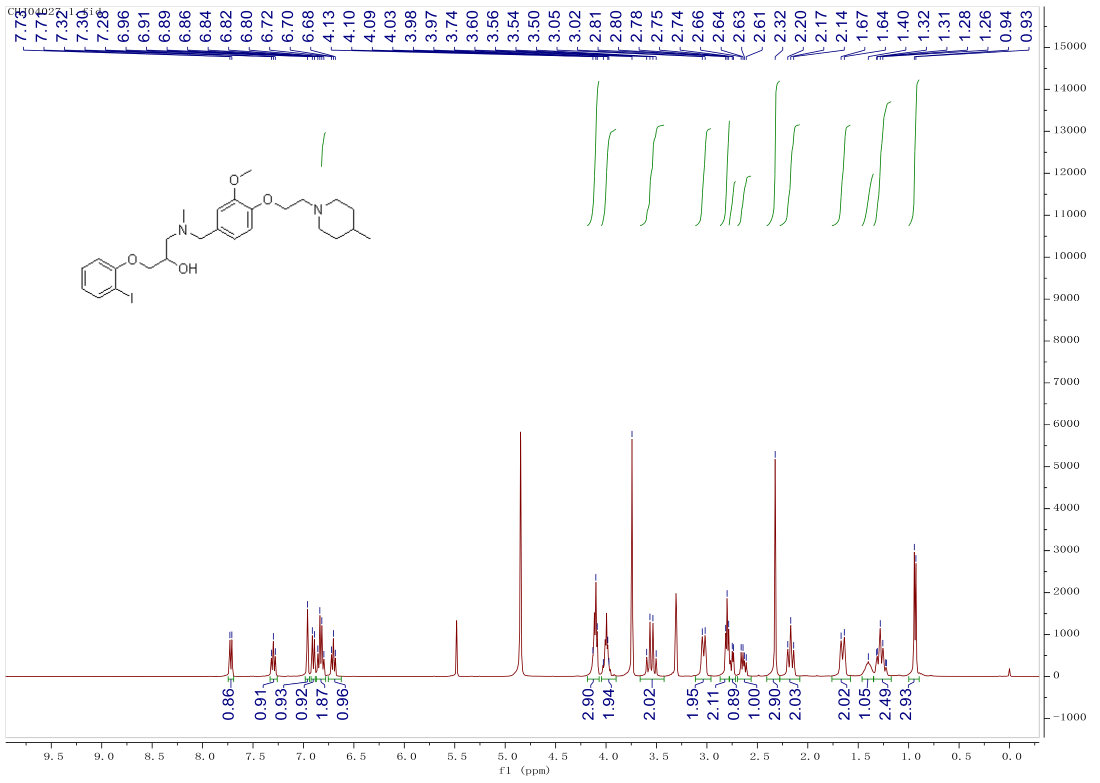

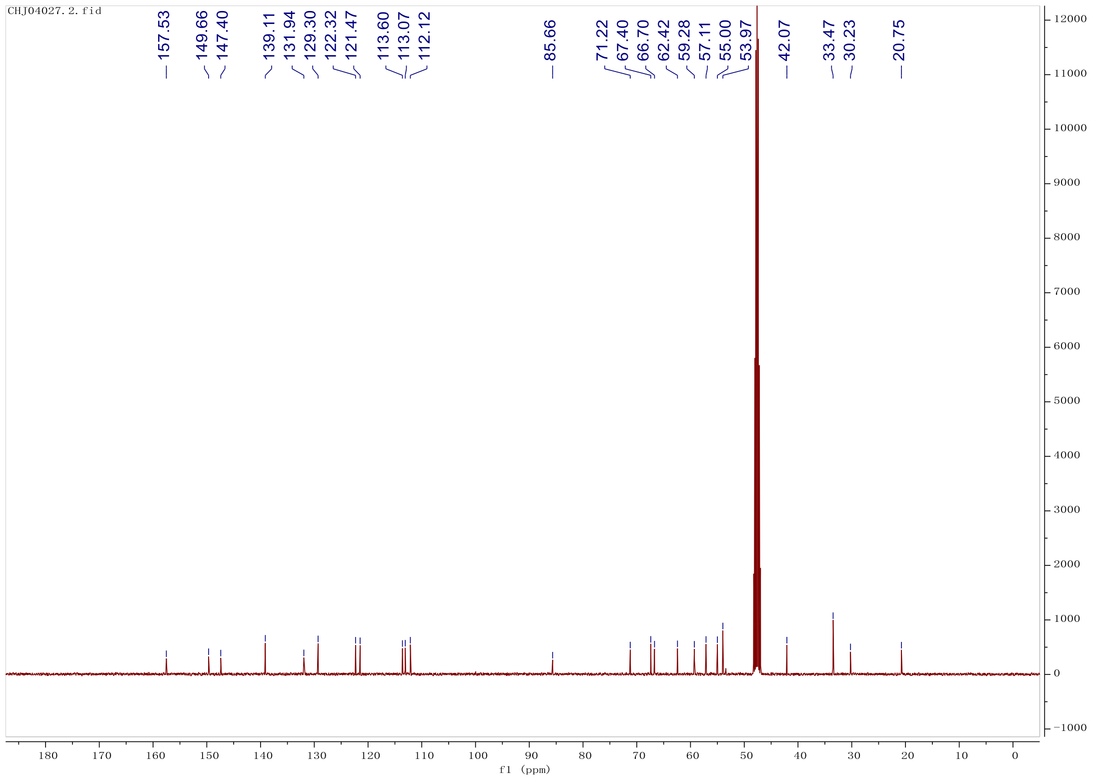
CHJ04027.2.fid
157.53
149.66
147.40
139.11
131.94
129.30
122.32
121.47
113.60
113.07
112.12
85.66
71.22
67.40
66.70
62.42
59.28
57.11
55.00
53.97
42.07
33.47
30.23
20.75
12000
11000
10000
9000
8000
7000
6000
5000
4000
3000
2000
1000
0
−1000
180
170
160
150
140
130
120
110
100
90
80
70
60
50
40
30
20
10
0
f1 (ppm)

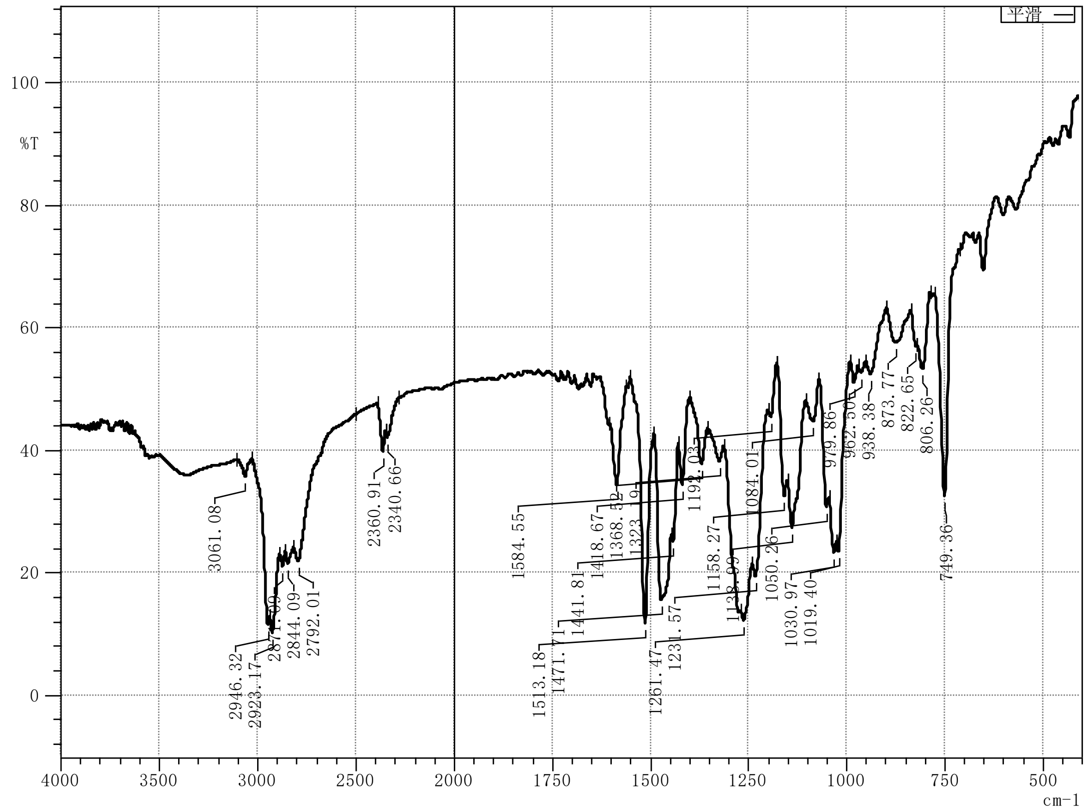
100
%T
80
60
40
20
0
3061.08
2946.32
2923.17
2844.09
2792.01
2360.91
2340.66
1584.55
1513.18
1471.71
1441.81
1418.67
1368.52
1261.47
1231.57
1192.03
1158.27
1084.01
1050.26
1030.97
1019.40
979.86
962.50
938.38
873.77
822.65
806.26
749.36
4000
3500
3000
2500
2000
1750
1500
1250
1000
750
500
cm-1

NMR (^{1}H and ^{13}C) and IR (KBr) of Compound CHJ04033:

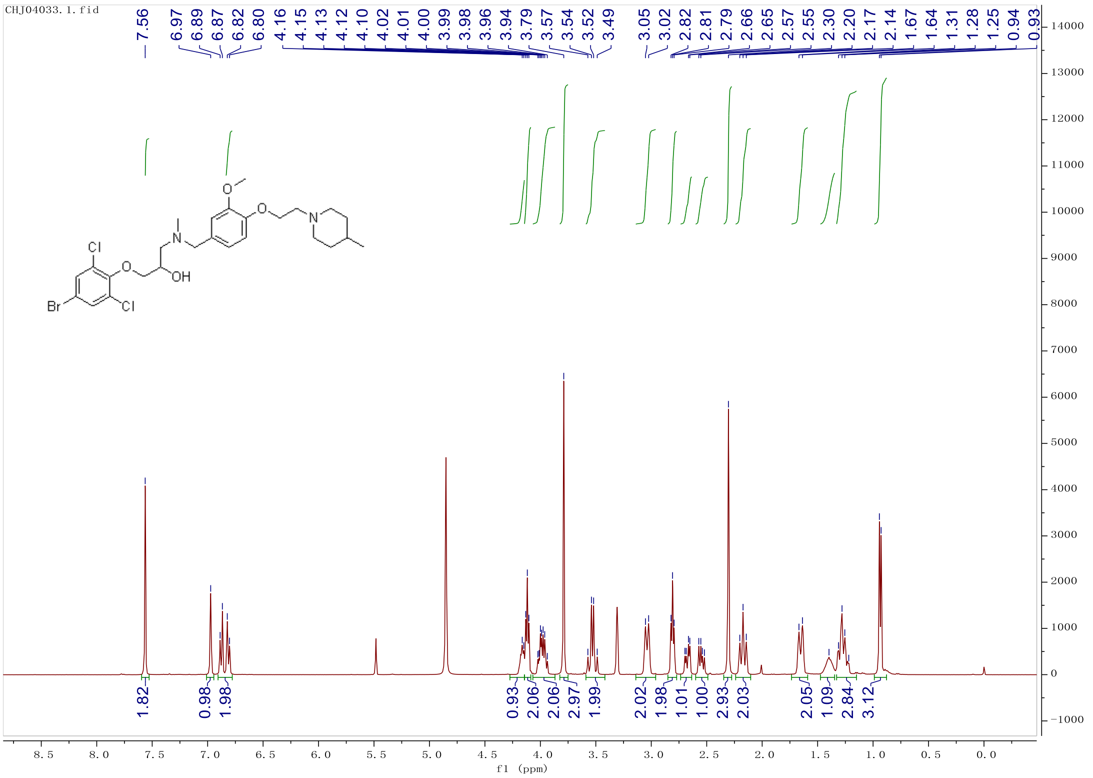

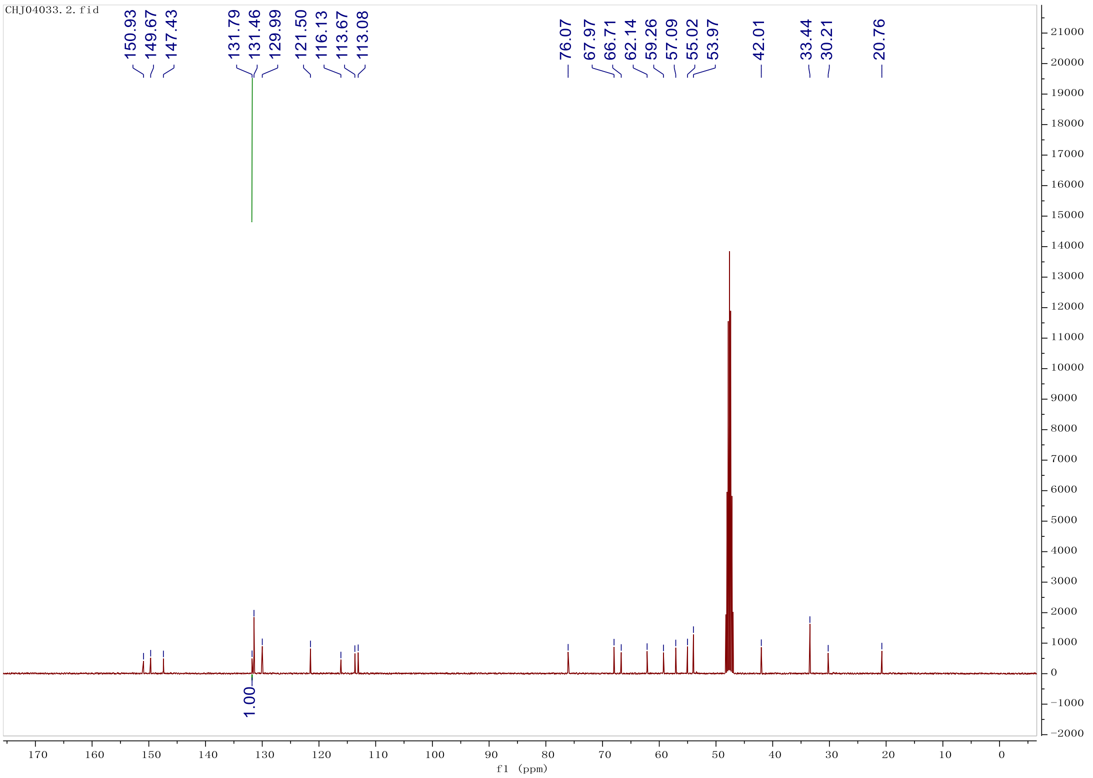

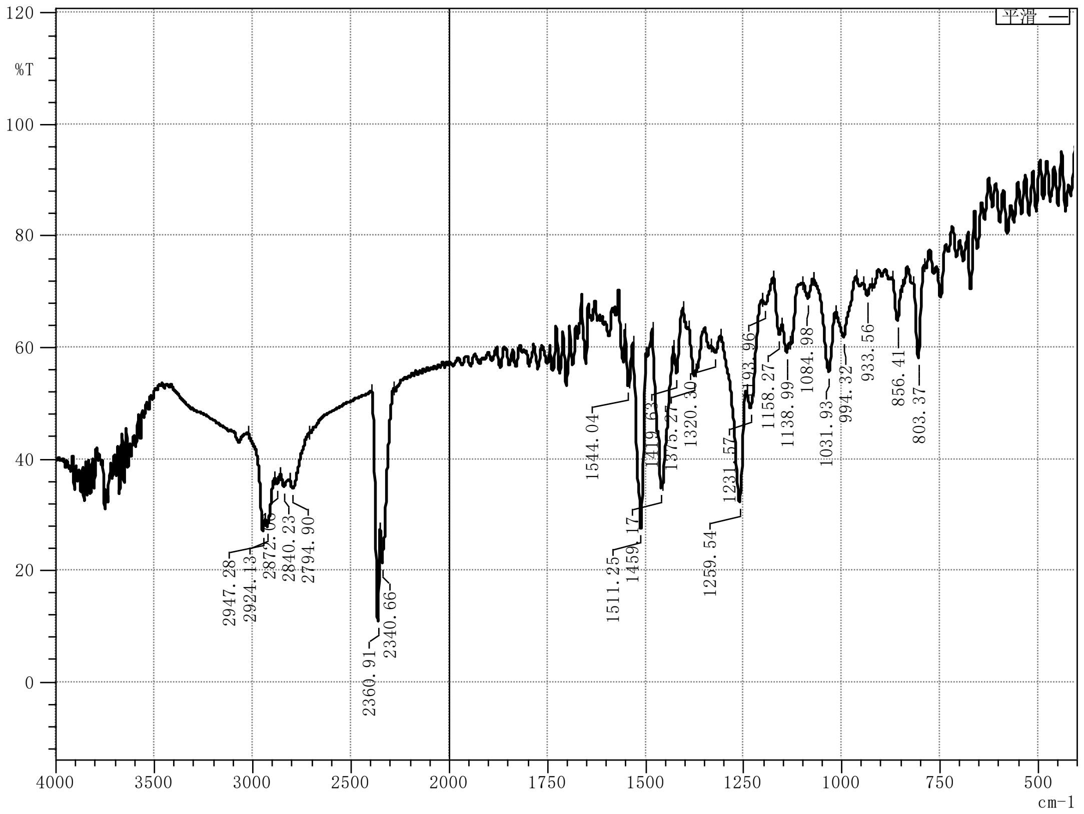

NMR (^{1}H and ^{13}C) and IR (KBr) of Compound CHJ04034:

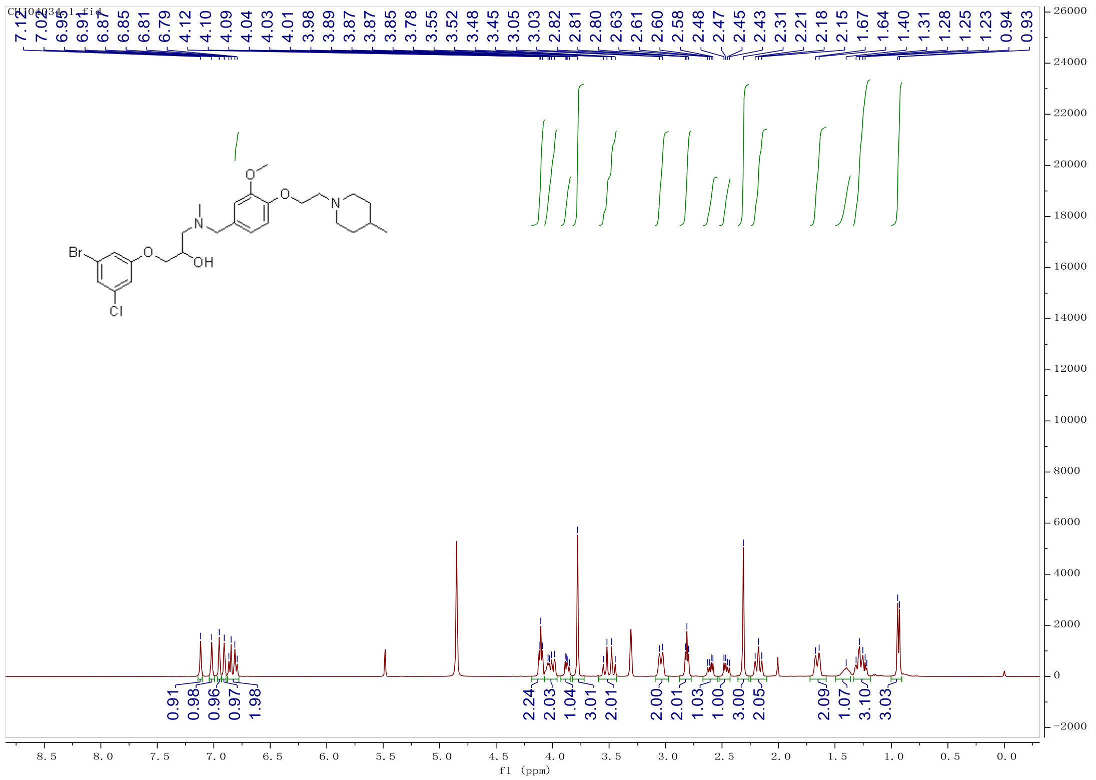

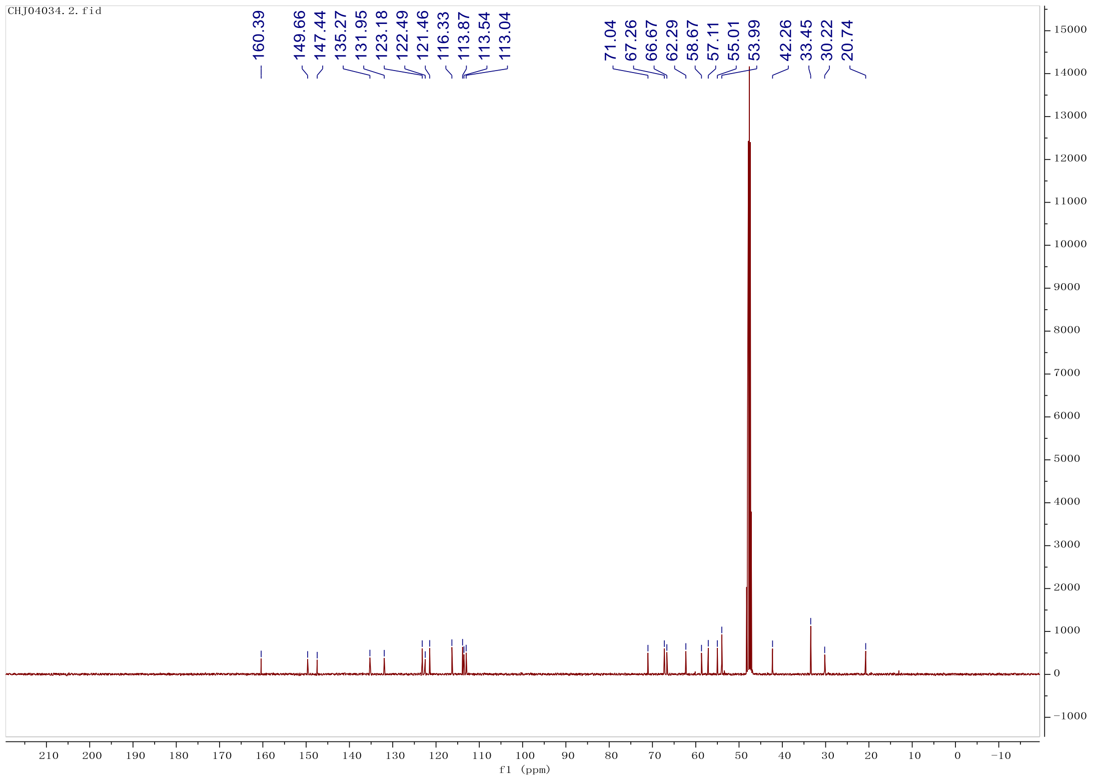

CHJ04034.2.fid
160.39
149.66
147.44
135.27
131.95
123.18
122.49
121.46
116.33
113.87
113.54
113.04
71.04
67.26
66.67
62.29
58.67
57.11
55.01
53.99
42.26
33.45
30.22
20.74
f1 (ppm)

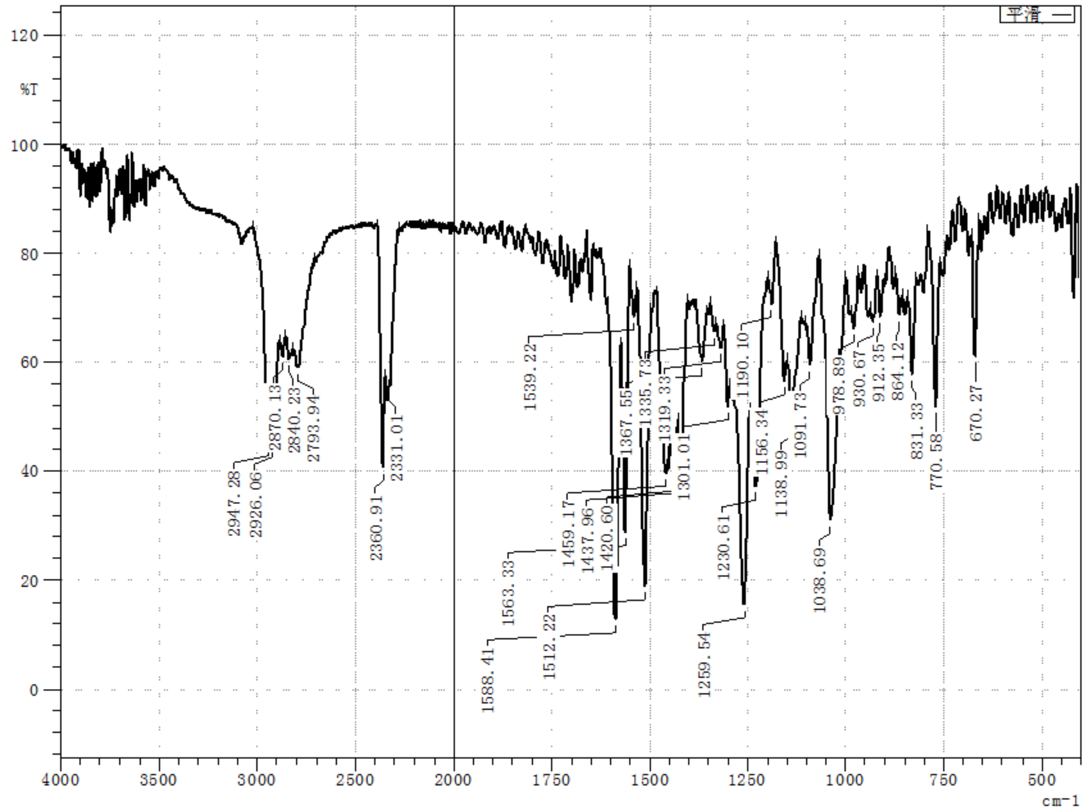

平滑
%T
2947.28
2926.06
2870.13
2840.23
2793.94
2360.91
2331.01
1588.41
1563.33
1539.22
1512.22
1459.17
1437.96
1420.60
1367.55
1335.73
1319.33
1301.01
1259.54
1230.61
1190.10
1156.34
1138.99
1091.73
1038.69
978.89
930.67
912.35
864.12
831.33
770.58
670.27
cm-1

NMR (^{1}H and ^{13}C) and IR (KBr) of Compound CHJ04036:

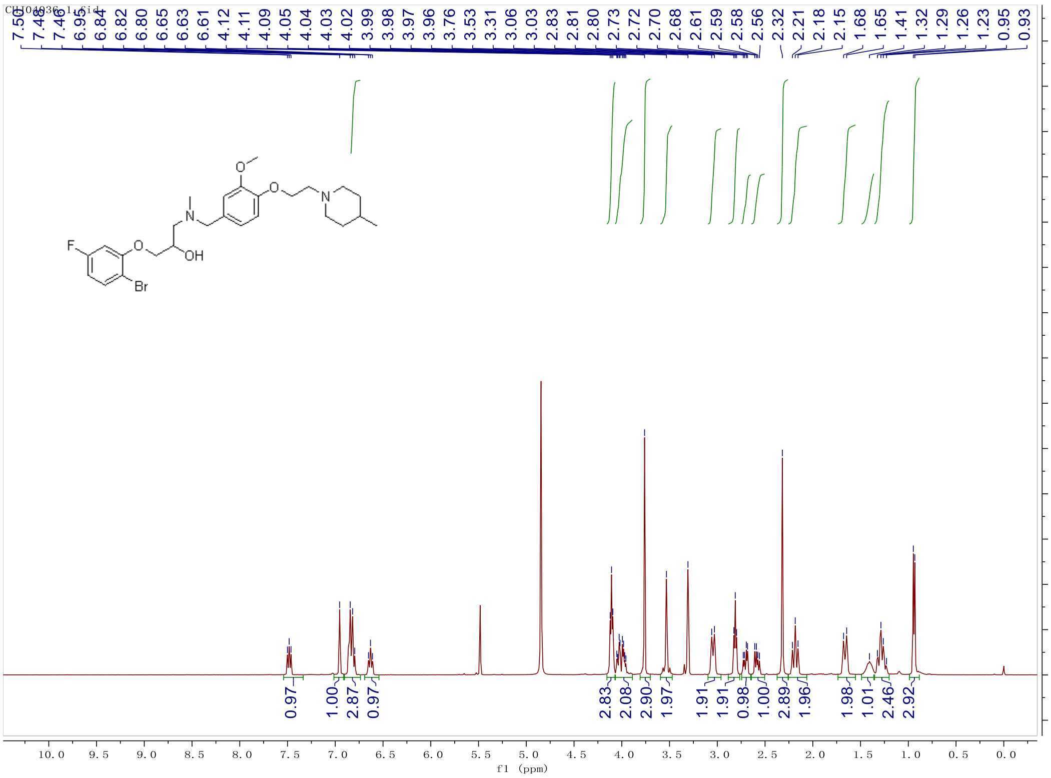

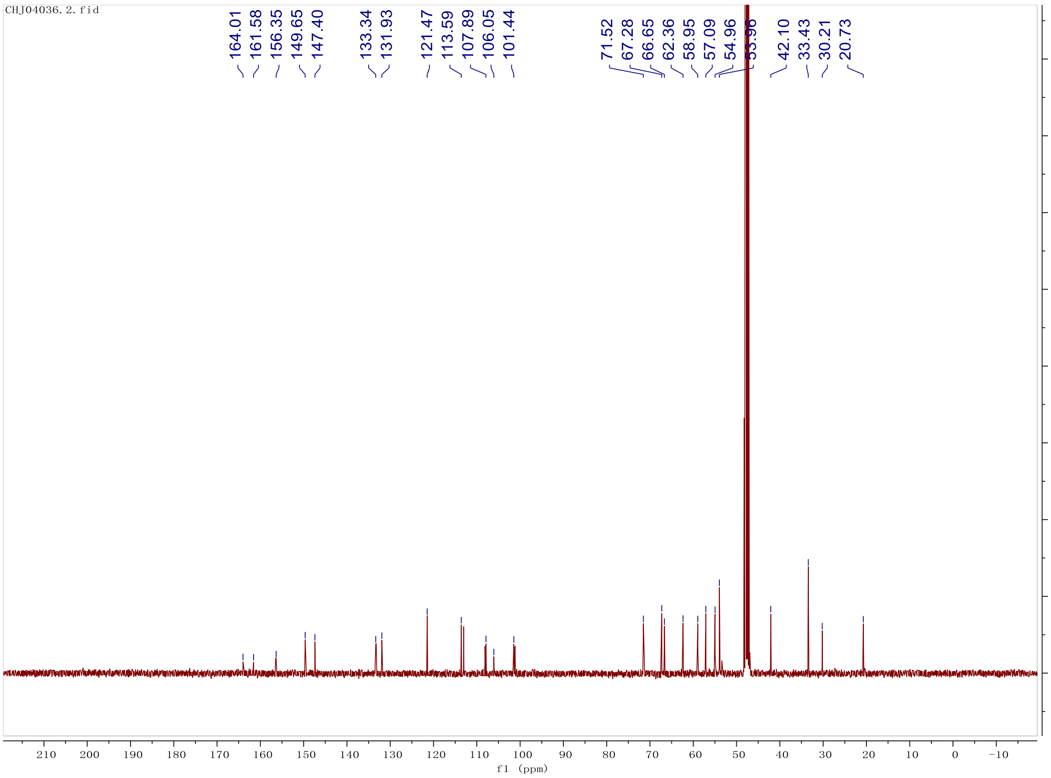

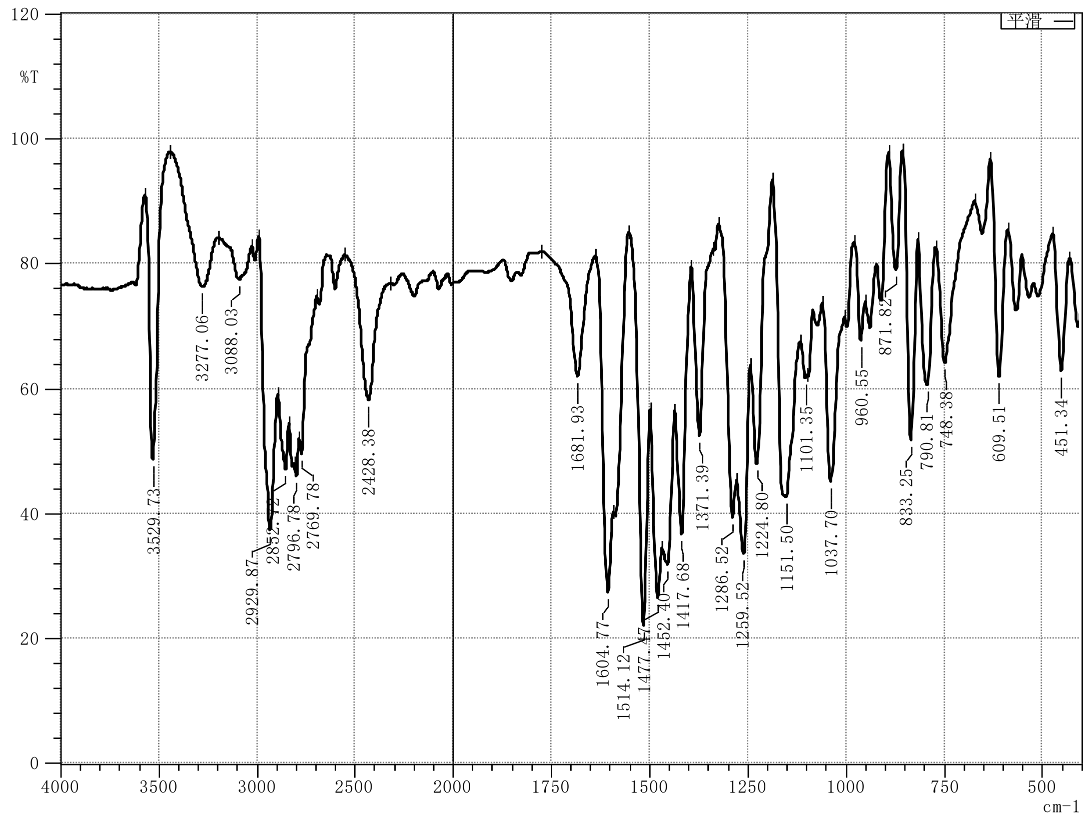

NMR (^{1}H and ^{13}C) and IR (KBr) of Compound CHJ04058:

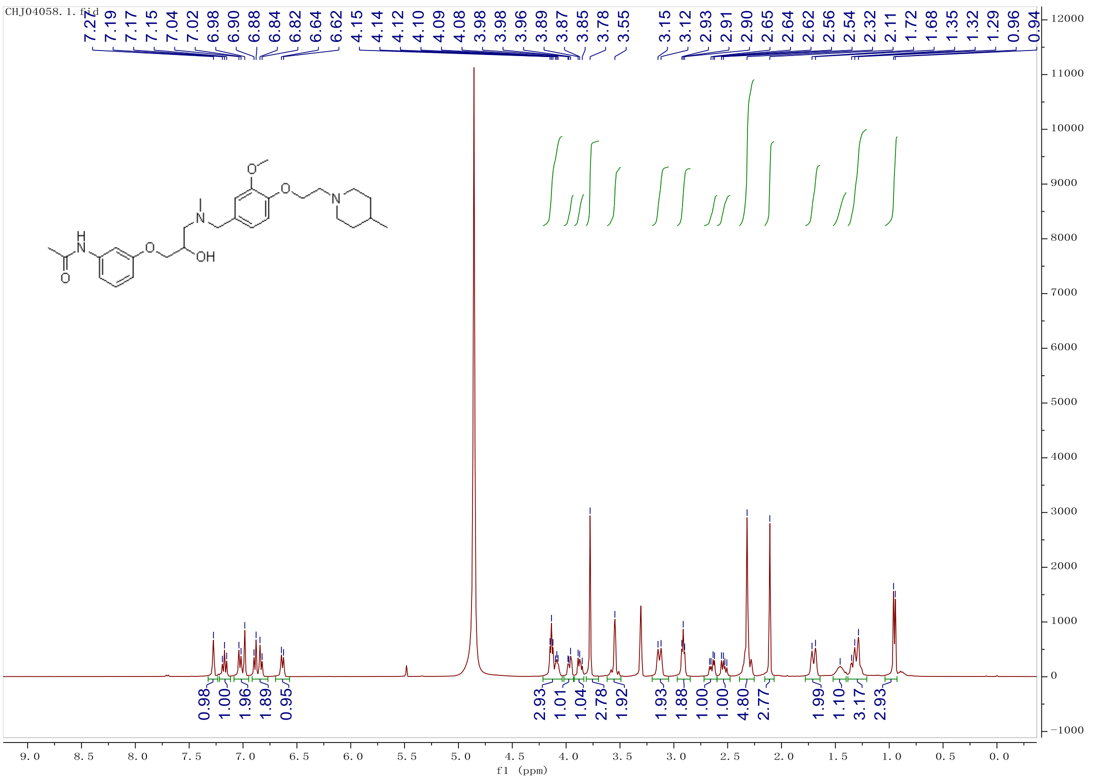

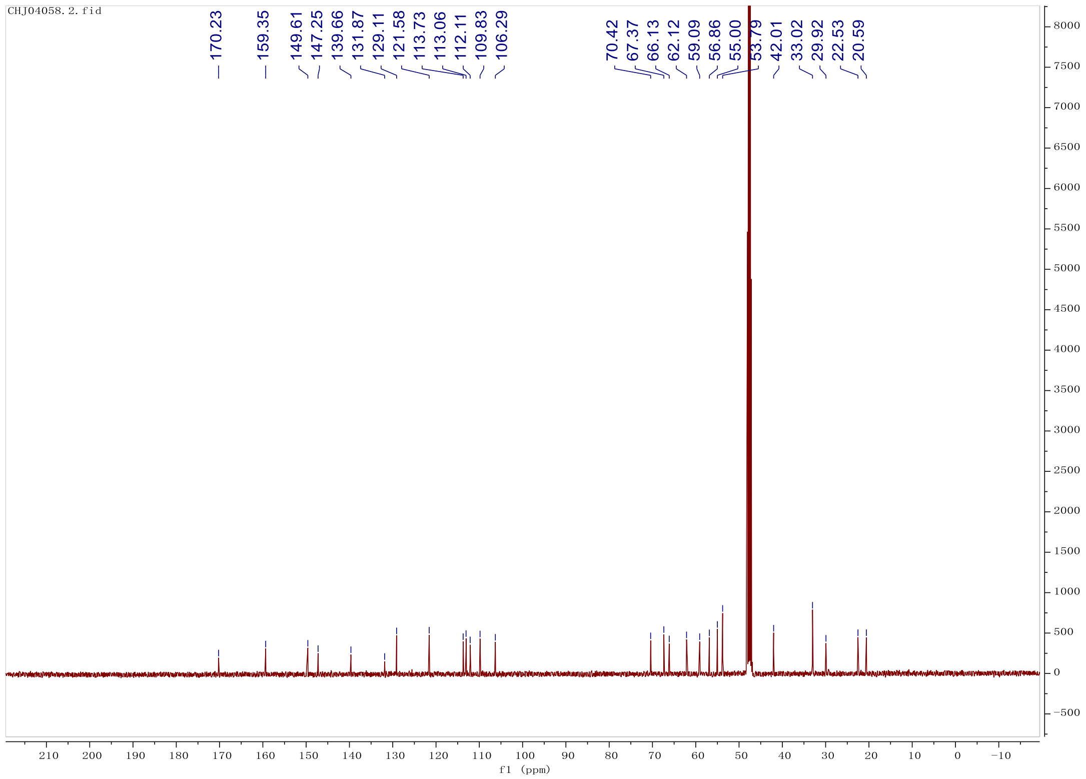
CHJ04058.2.fid
170.23
159.35
149.61
147.25
139.66
131.87
129.11
121.58
113.73
113.06
112.11
109.83
106.29
70.42
67.37
66.13
62.12
59.09
56.86
55.00
53.79
42.01
33.02
29.92
22.53
20.59
f1 (ppm)

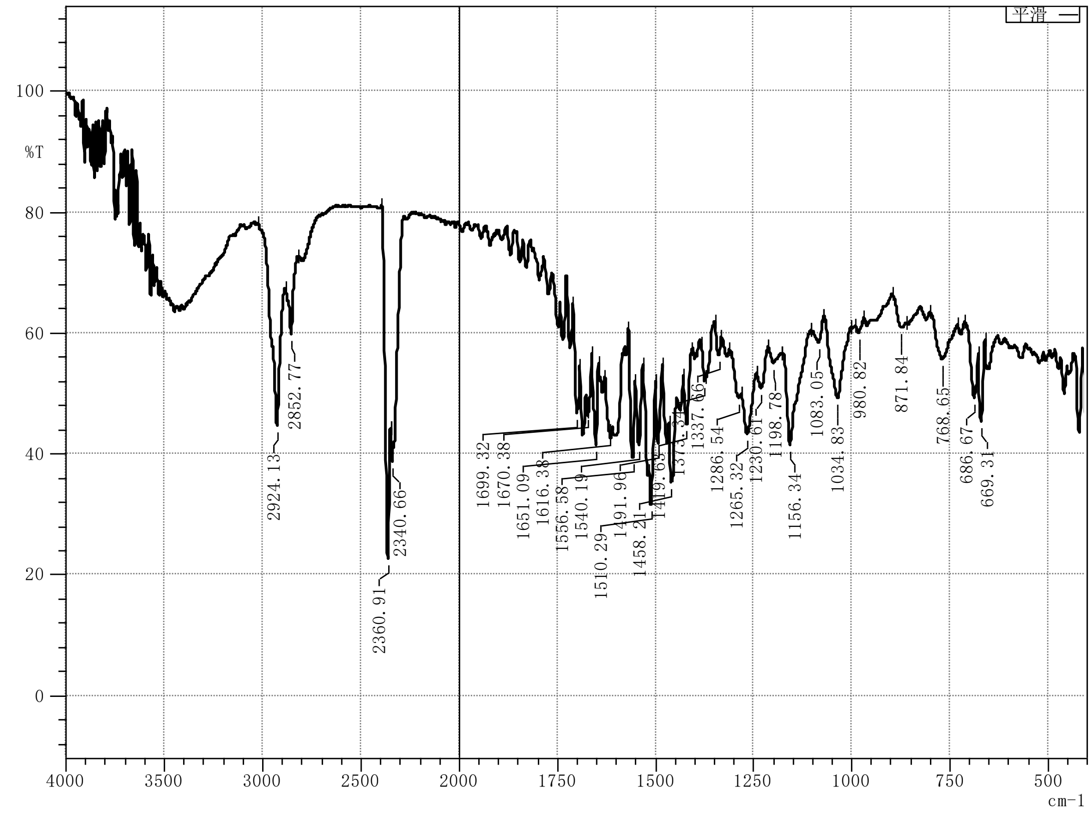
%T
cm-1
2924.13
2852.77
2360.91
2340.66
1699.32
1670.38
1651.09
1616.38
1556.58
1540.19
1510.29
1491.96
1458.21
1337.66
1286.54
1265.32
1230.61
1198.78
1156.34
1083.05
1034.83
980.82
871.84
768.65
686.67
669.31

NMR (^{1}H and ^{13}C) and IR (KBr) of Compound CHJ04059:

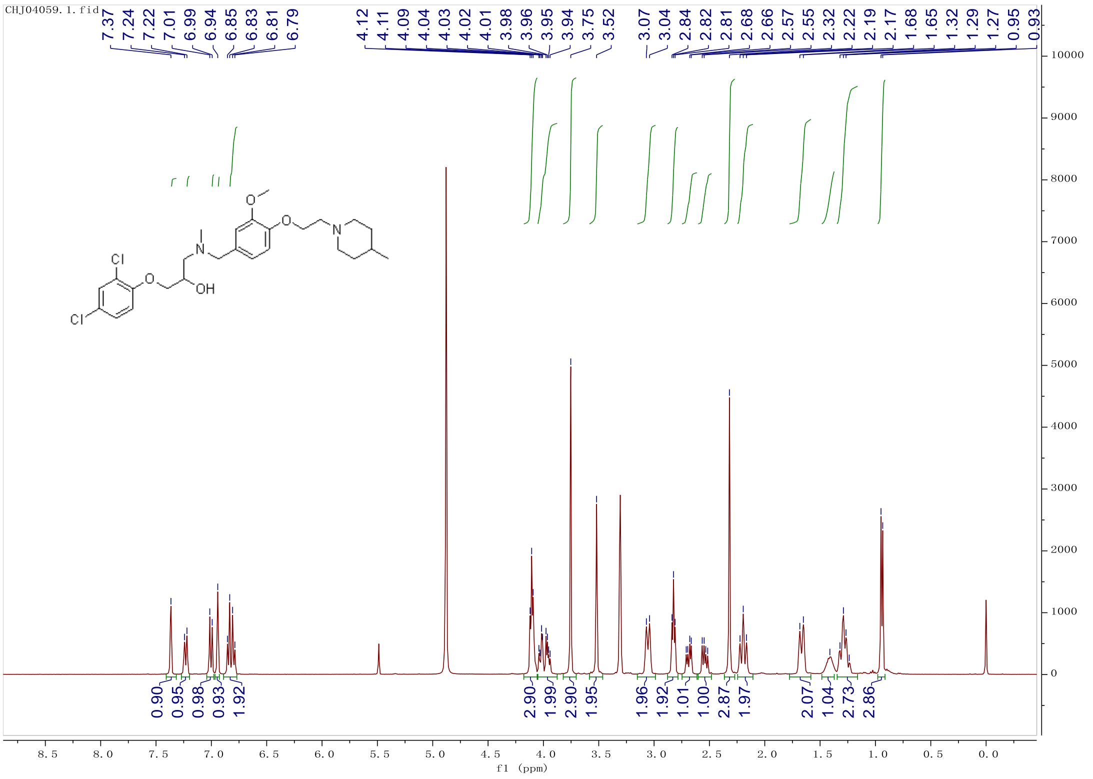

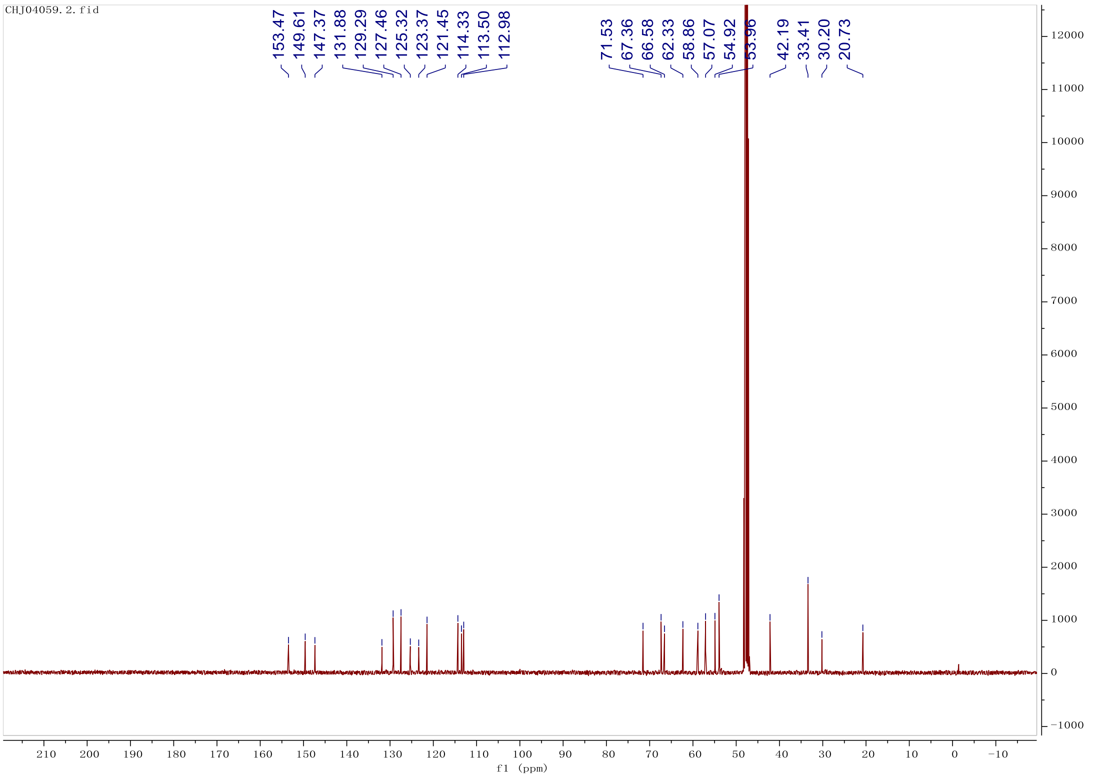

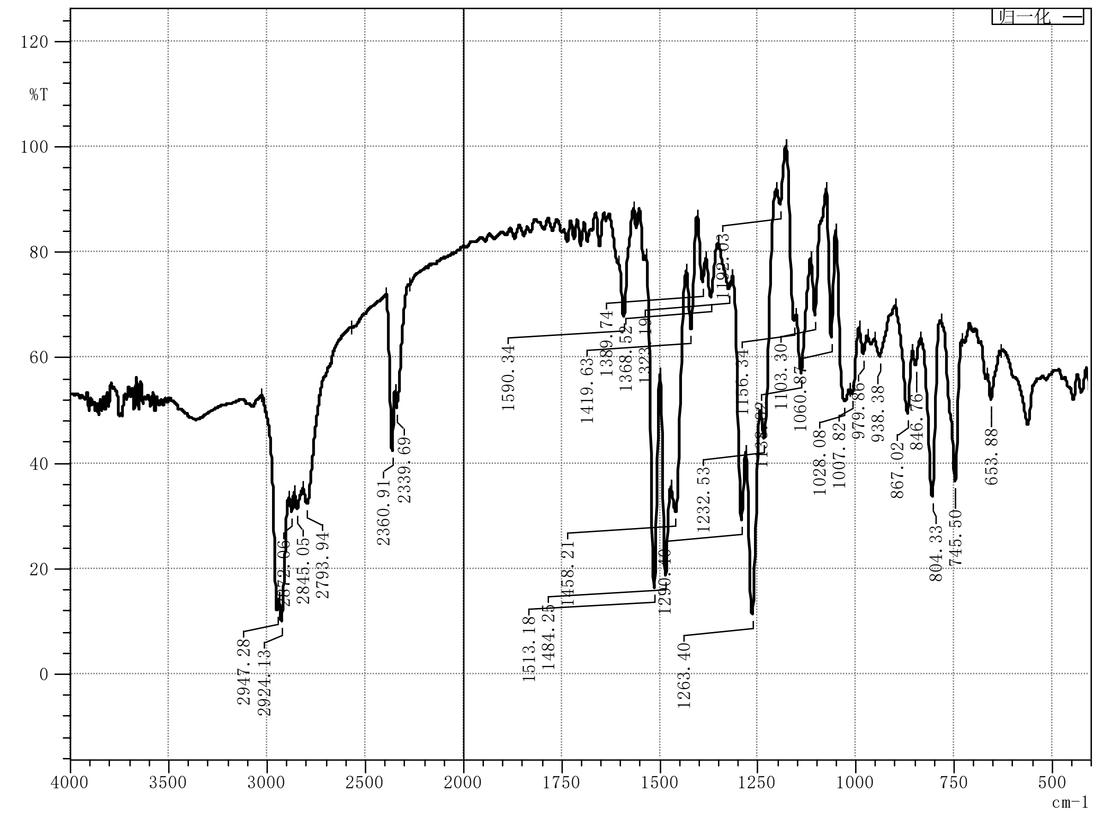

NMR (^{1}H and ^{13}C) and IR (KBr) of Compound CHJ04060:

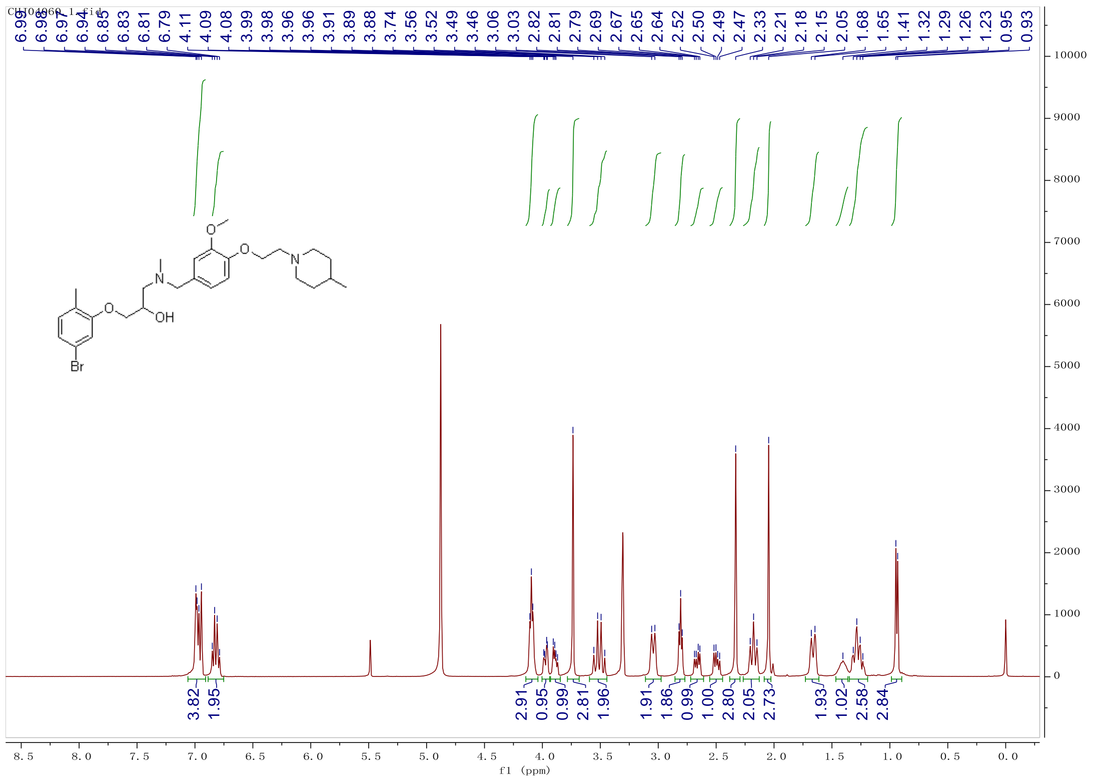

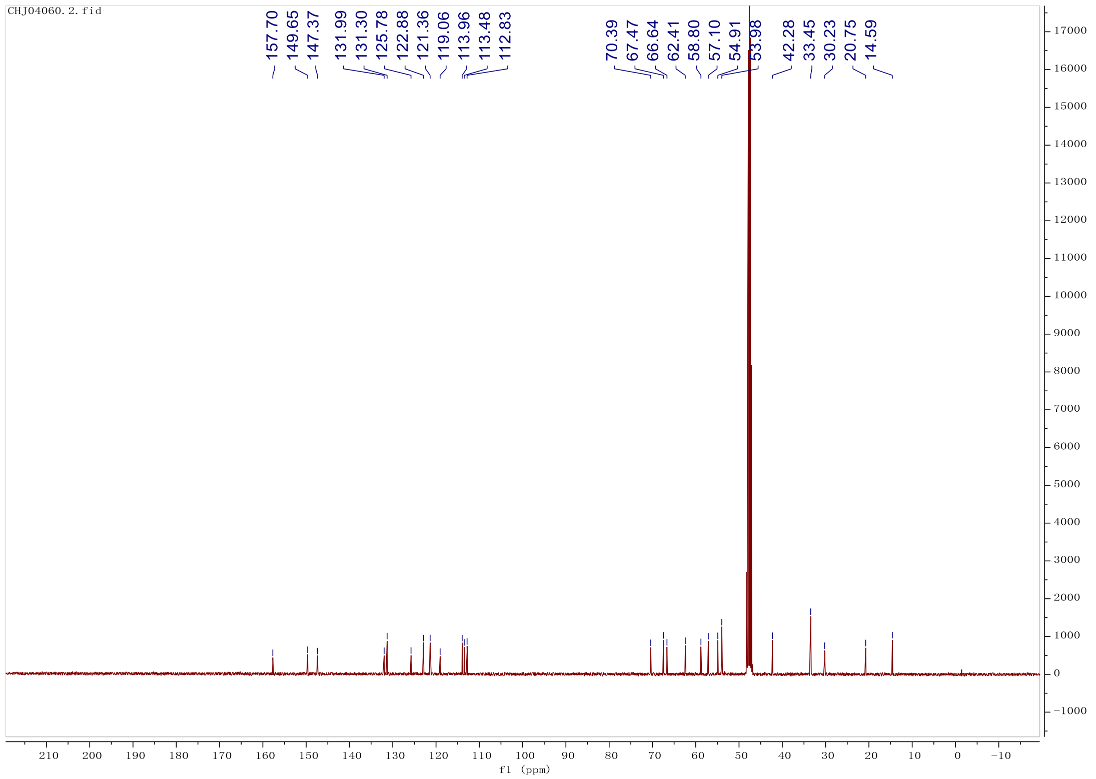
CHJ04060.2.fid
157.70
149.65
147.37
131.99
131.30
125.78
122.88
121.36
119.06
113.96
113.48
112.83
70.39
67.47
66.64
62.41
58.80
57.10
54.91
53.98
42.28
33.45
30.23
20.75
14.59
f1 (ppm)

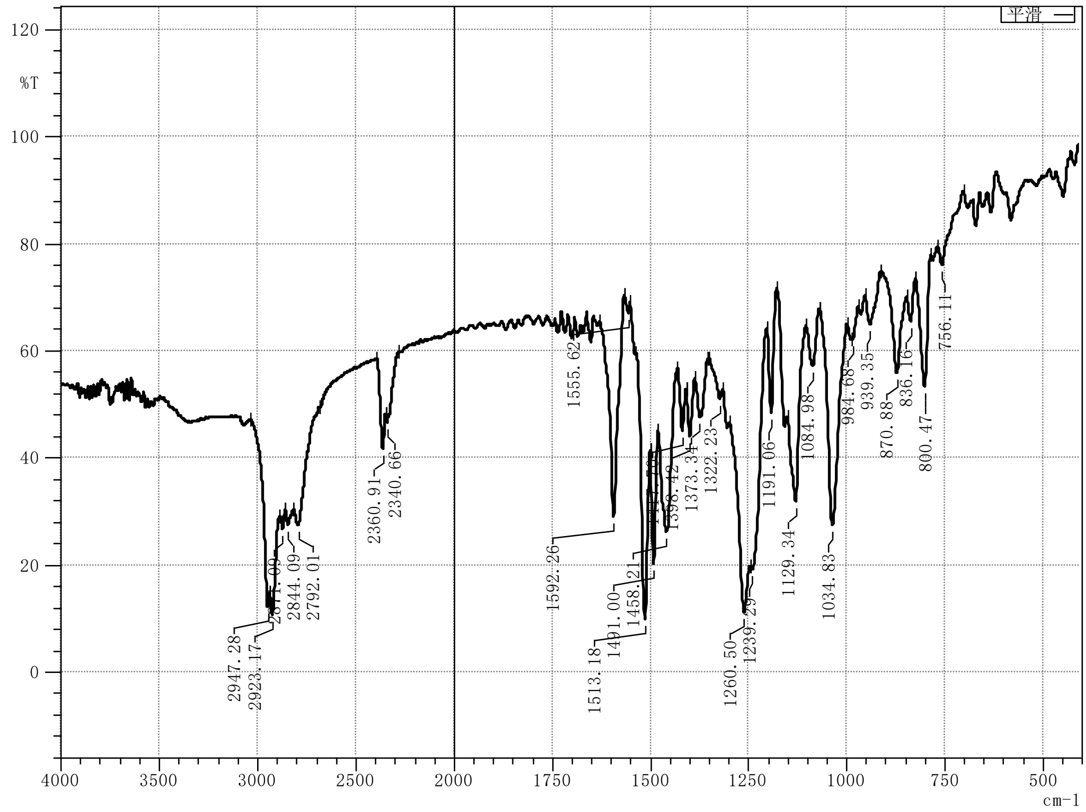
%T
cm-1
2947.28
2923.17
2844.09
2792.01
2360.91
2340.66
1592.26
1555.62
1513.18
1491.00
1458.21
1398.42
1373.34
1322.23
1260.50
1239.29
1191.06
1129.34
1084.98
1034.83
984.68
939.35
870.88
836.16
800.47
756.11

NMR (^{1}H and ^{13}C) and IR (KBr) of Compound CHJ04061:

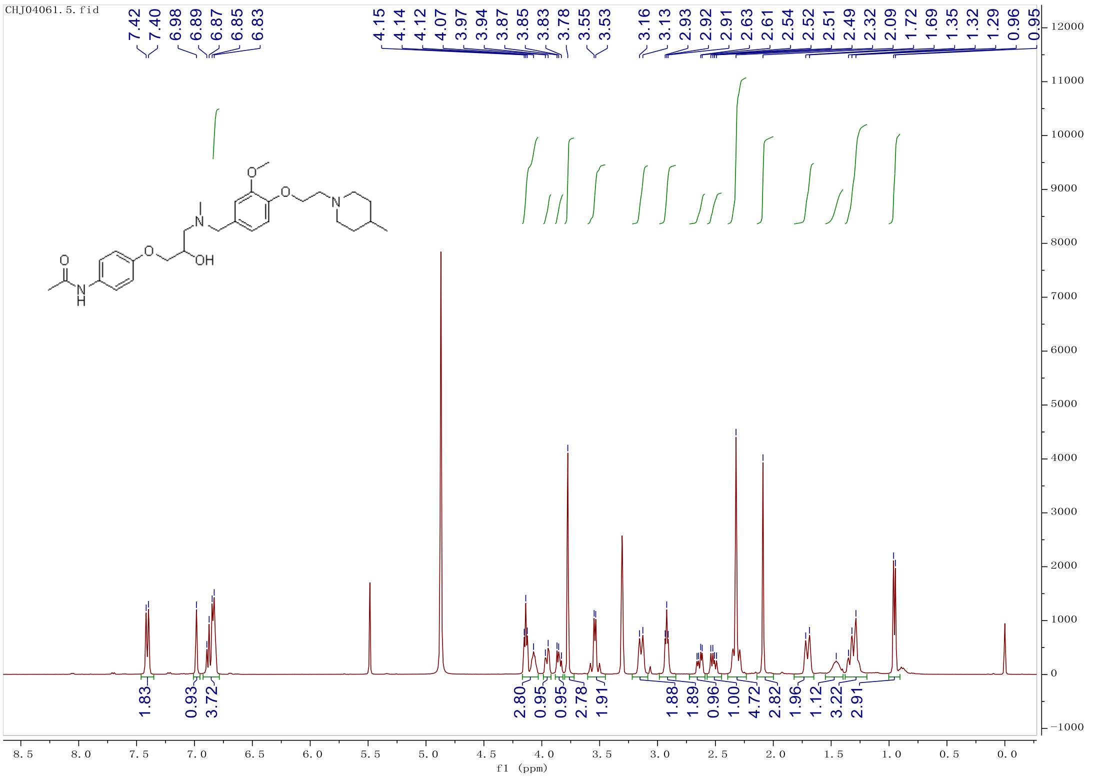

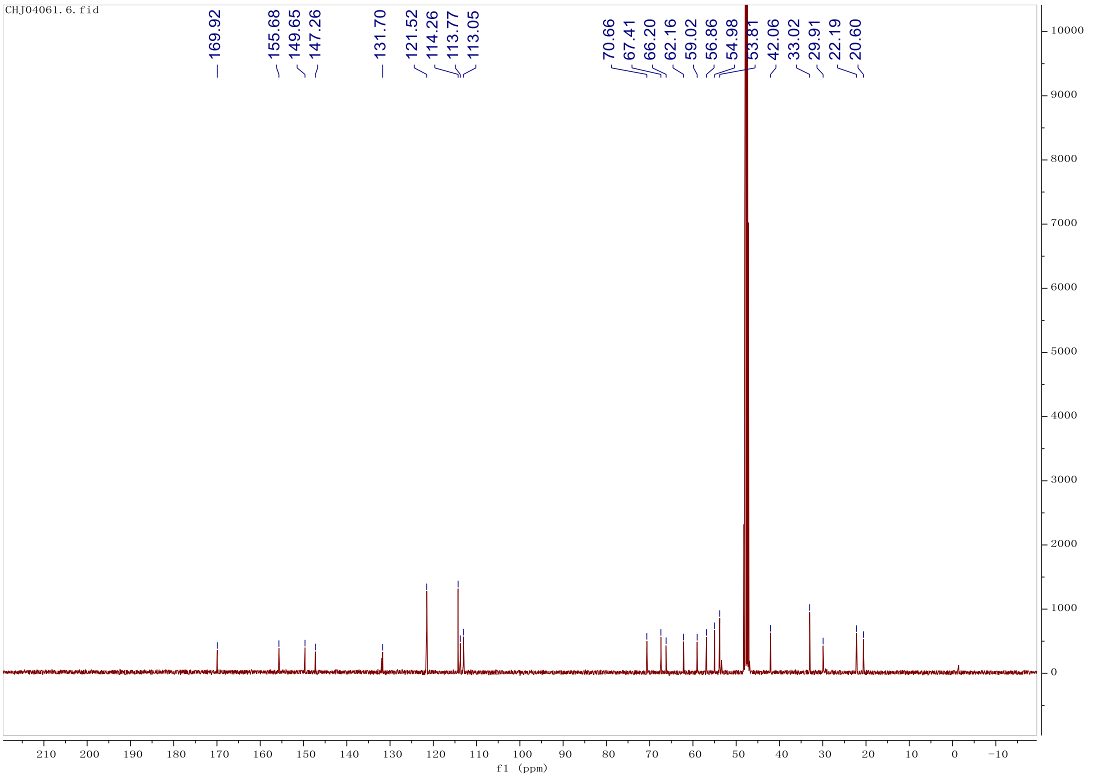

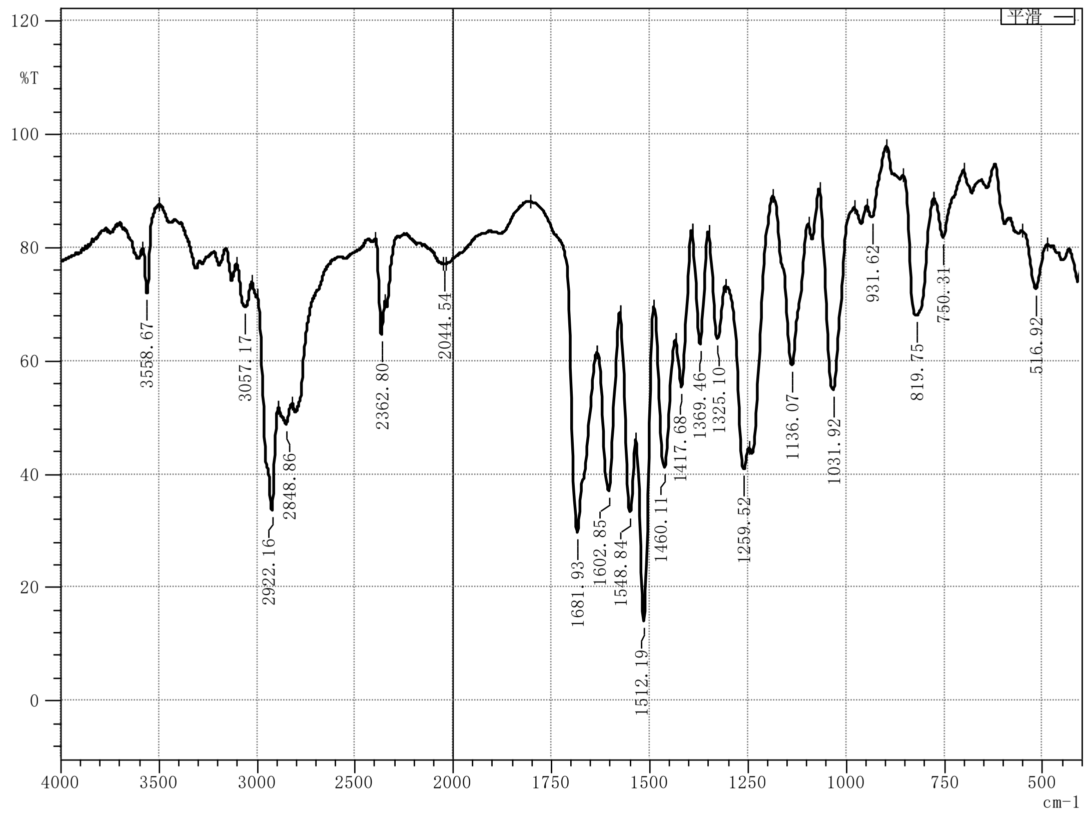

NMR (^{1}H and ^{13}C) and IR (KBr) of Compound CHJ04082:

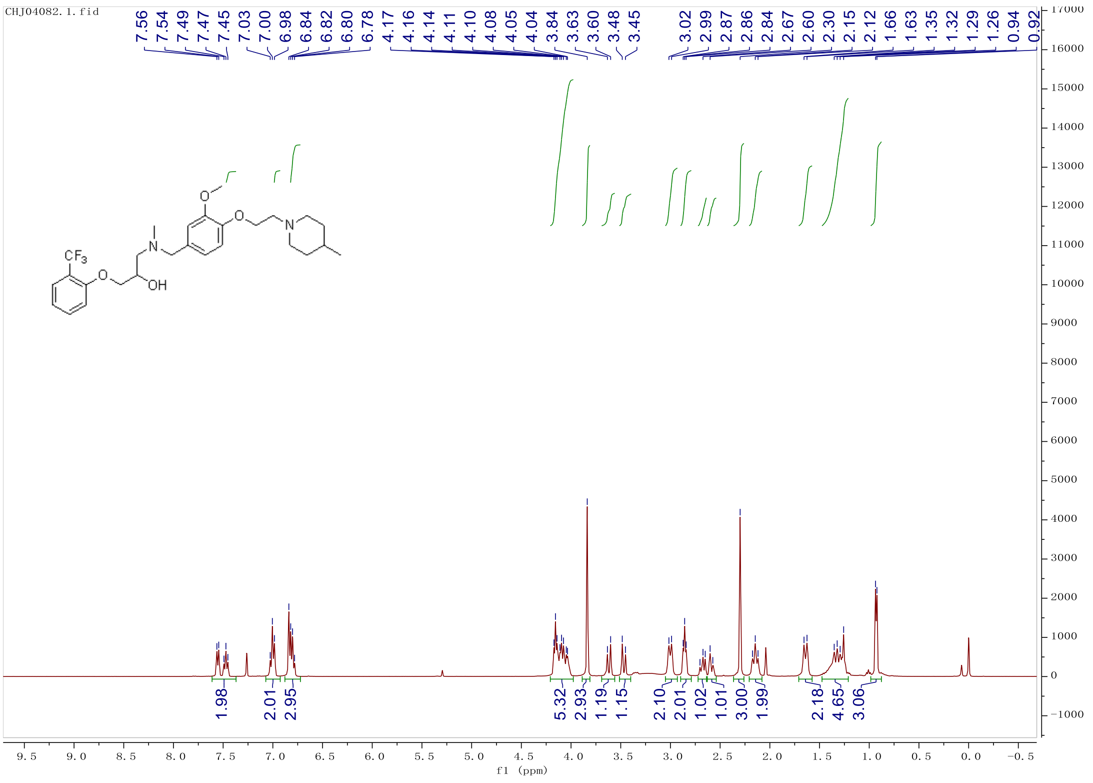

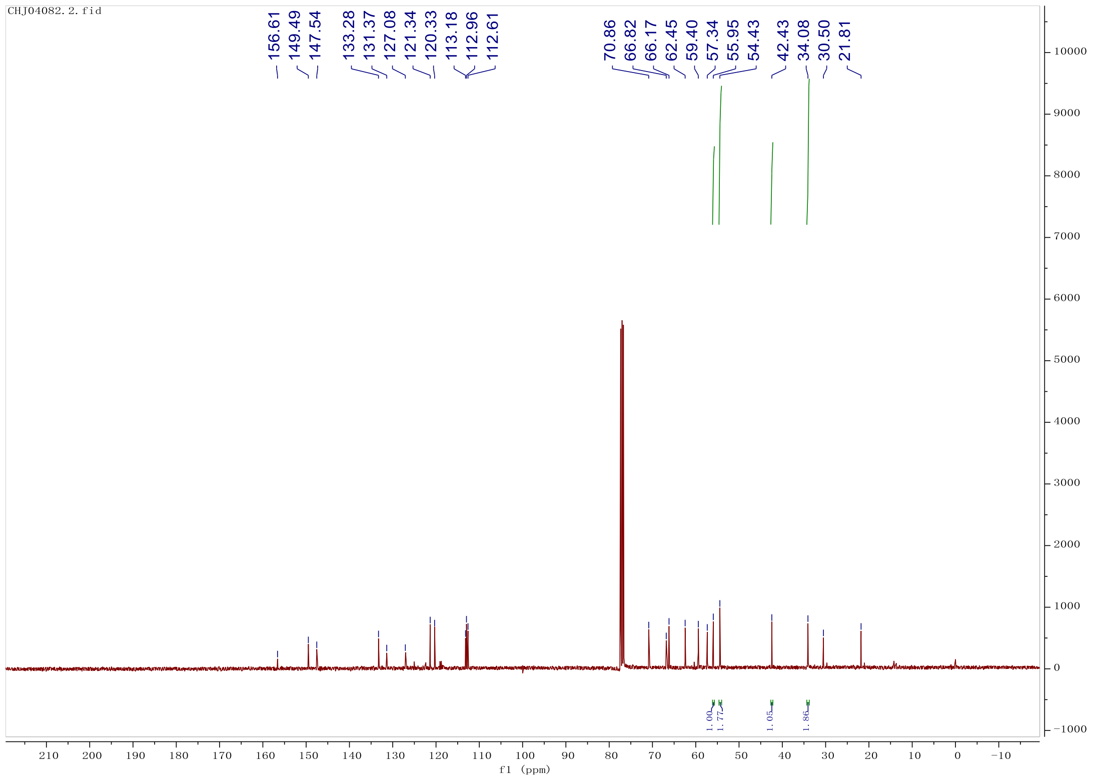
CHJ04082.2.fid
156.61
149.49
147.54
133.28
131.37
127.08
121.34
120.33
113.18
112.96
112.61
70.86
66.82
66.17
62.45
59.40
57.34
55.95
54.43
42.43
34.08
30.50
21.81
f1 (ppm)

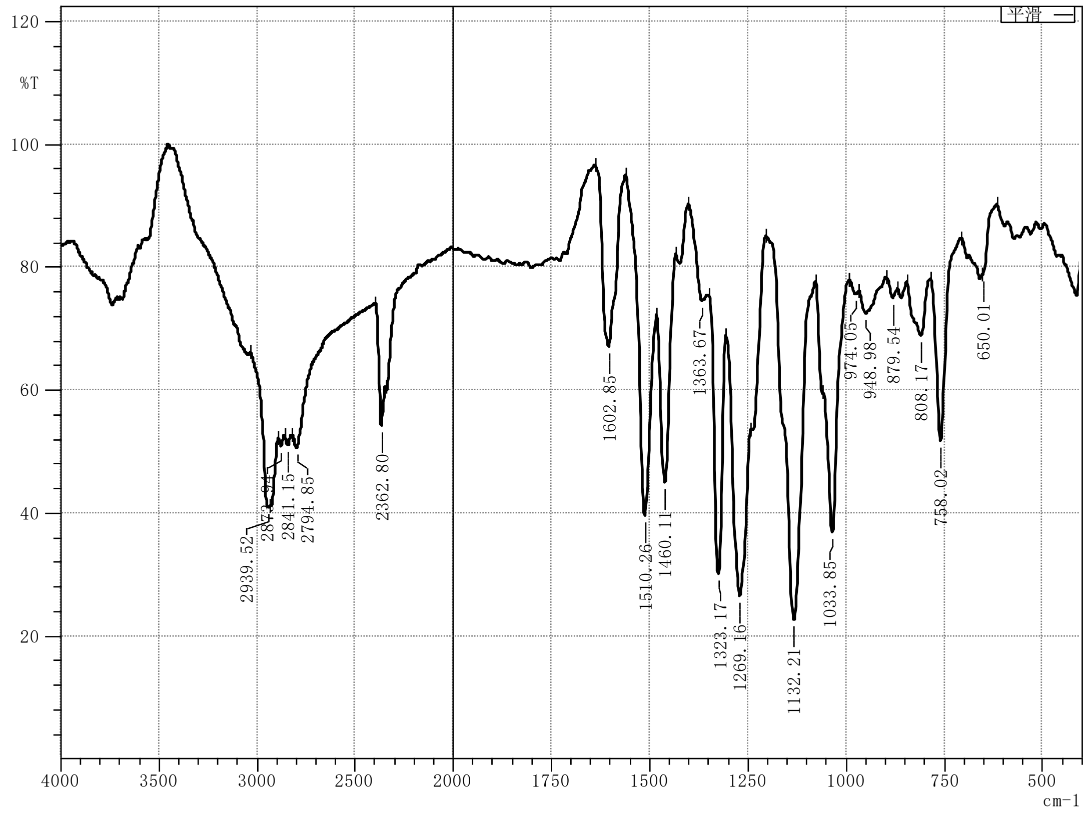
%T
cm-1
2939.52
2841.15
2794.85
2362.80
1602.85
1510.26
1460.11
1363.67
1323.17
1269.16
1132.21
1033.85
974.05
948.98
879.54
808.17
758.02
650.01

NMR (^{1}H and ^{13}C) and IR (KBr) of Compound CHJ04083:

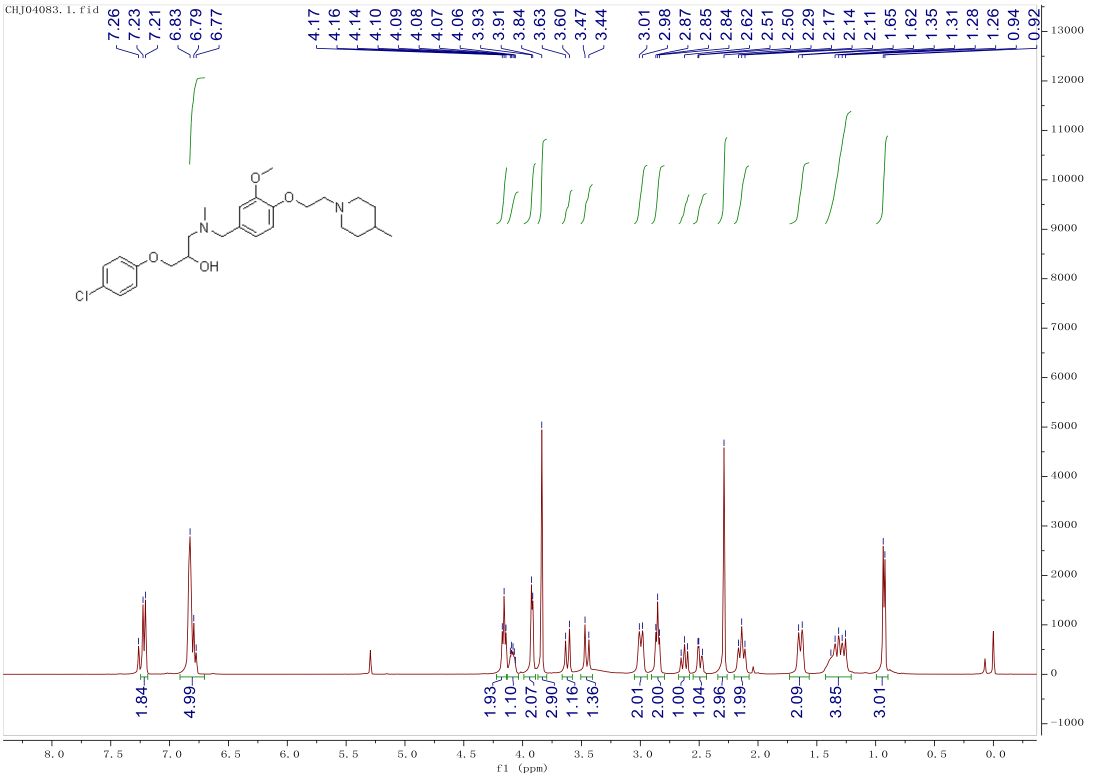

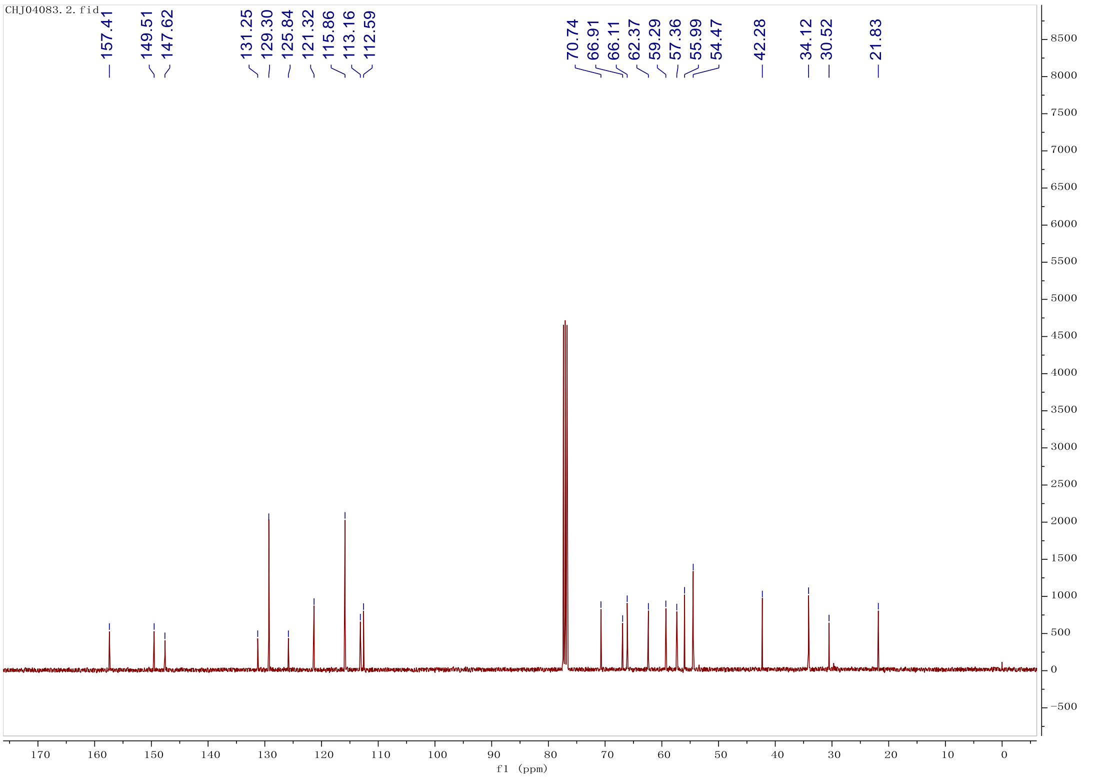

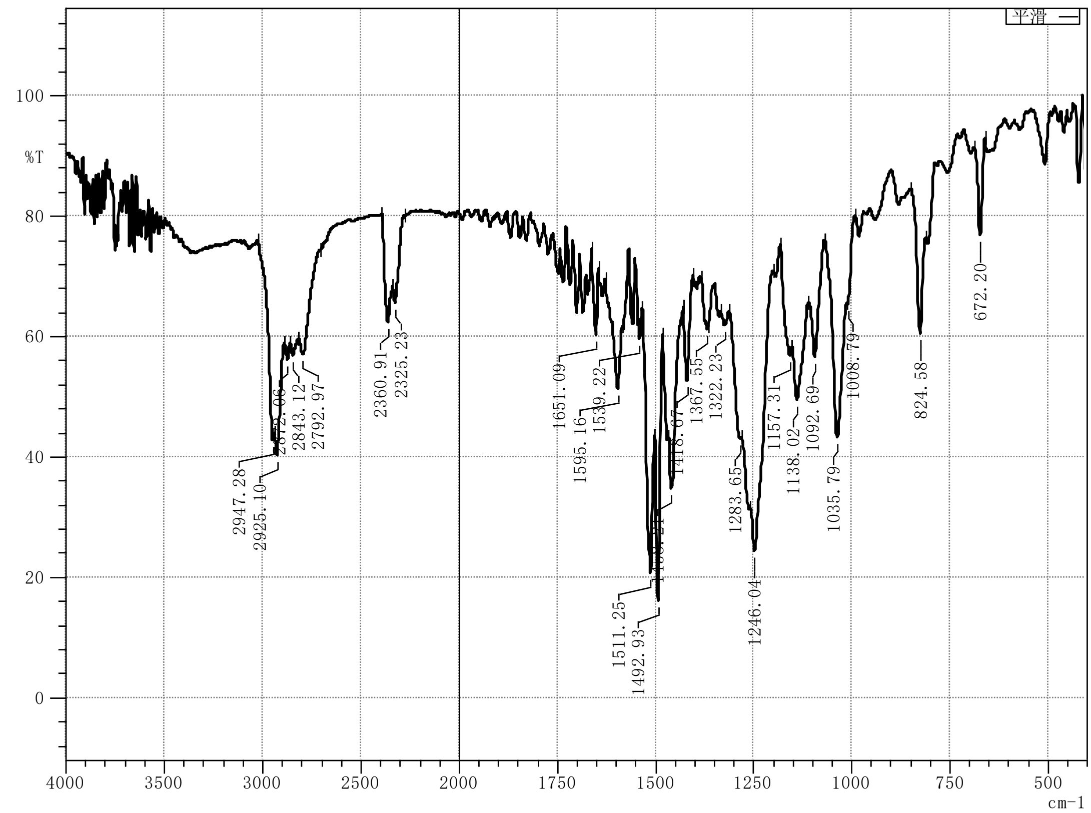

NMR (^{1}H and ^{13}C) and IR (KBr) of Compound CHJ04084:

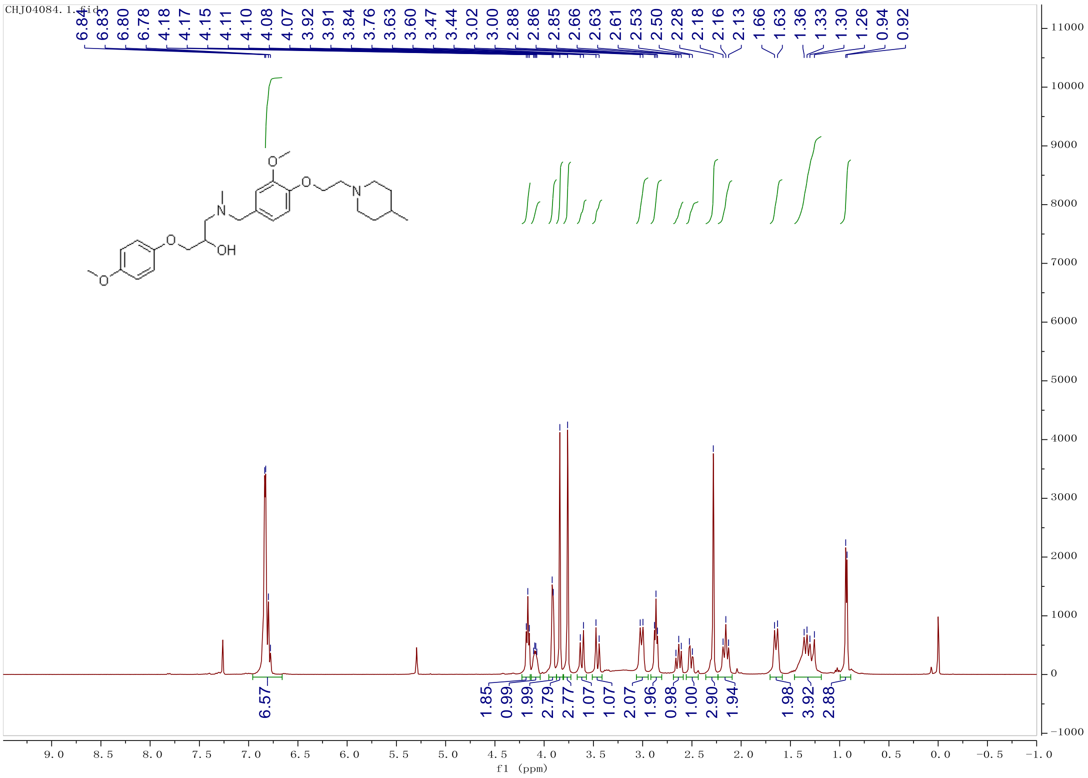

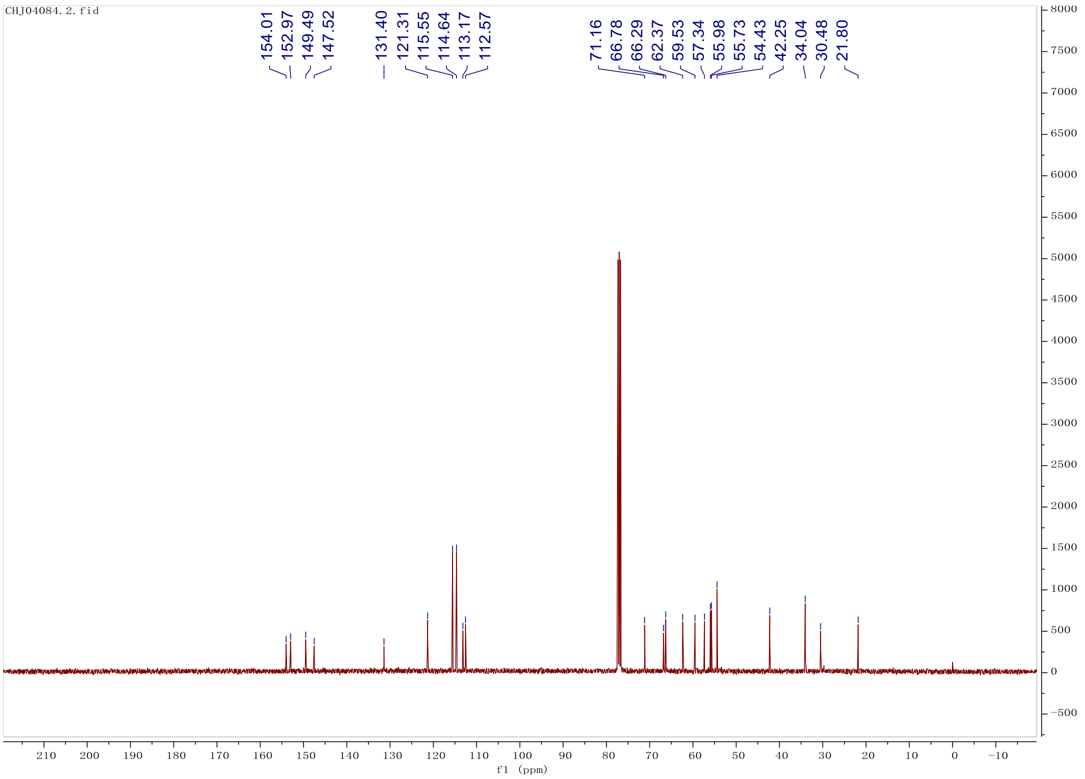
CHJ04084.2.fid
154.01
152.97
149.49
147.52
131.40
121.31
115.55
114.64
113.17
112.57
71.16
66.78
66.29
62.37
59.53
57.34
55.98
55.73
54.43
42.25
34.04
30.48
21.80
f1 (ppm)

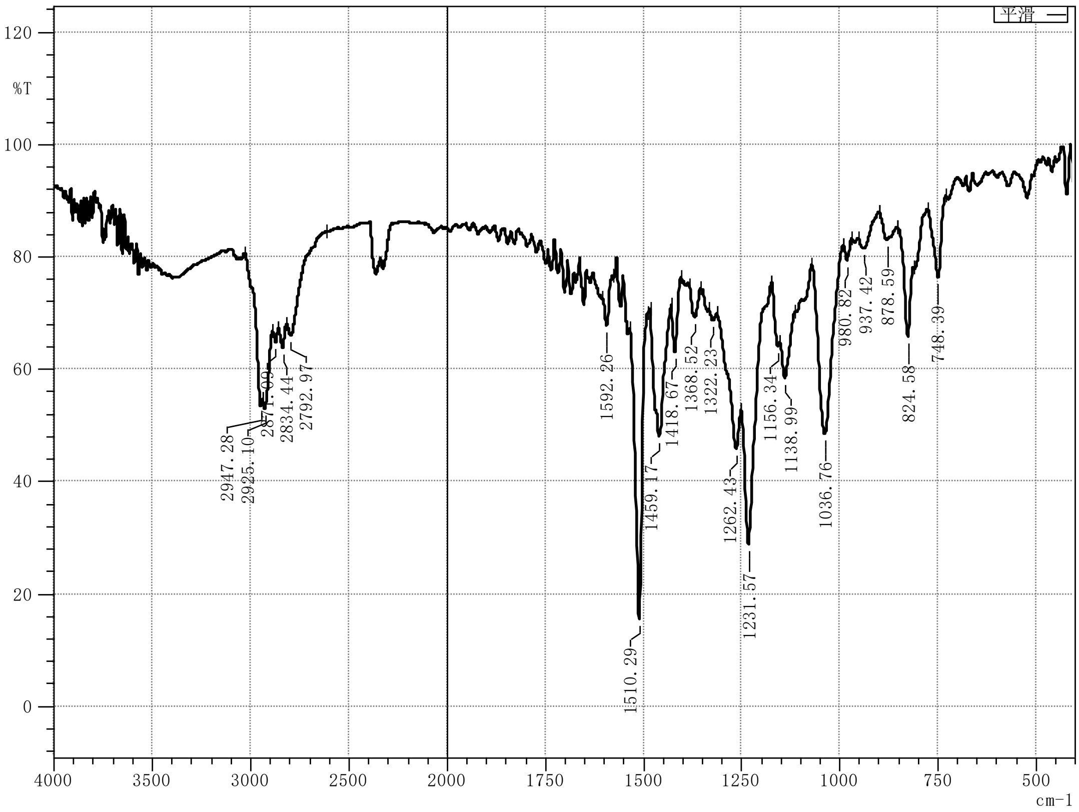
%T
cm-1
平滑
2947.28
2925.10
2871.09
2834.44
2792.97
1592.26
1510.29
1459.17
1418.67
1368.52
1322.23
1262.43
1231.57
1156.34
1138.99
1036.76
980.82
937.42
878.59
824.58
748.39

NMR (^{1}H and ^{13}C) and IR (KBr) of Compound CHJ04085:

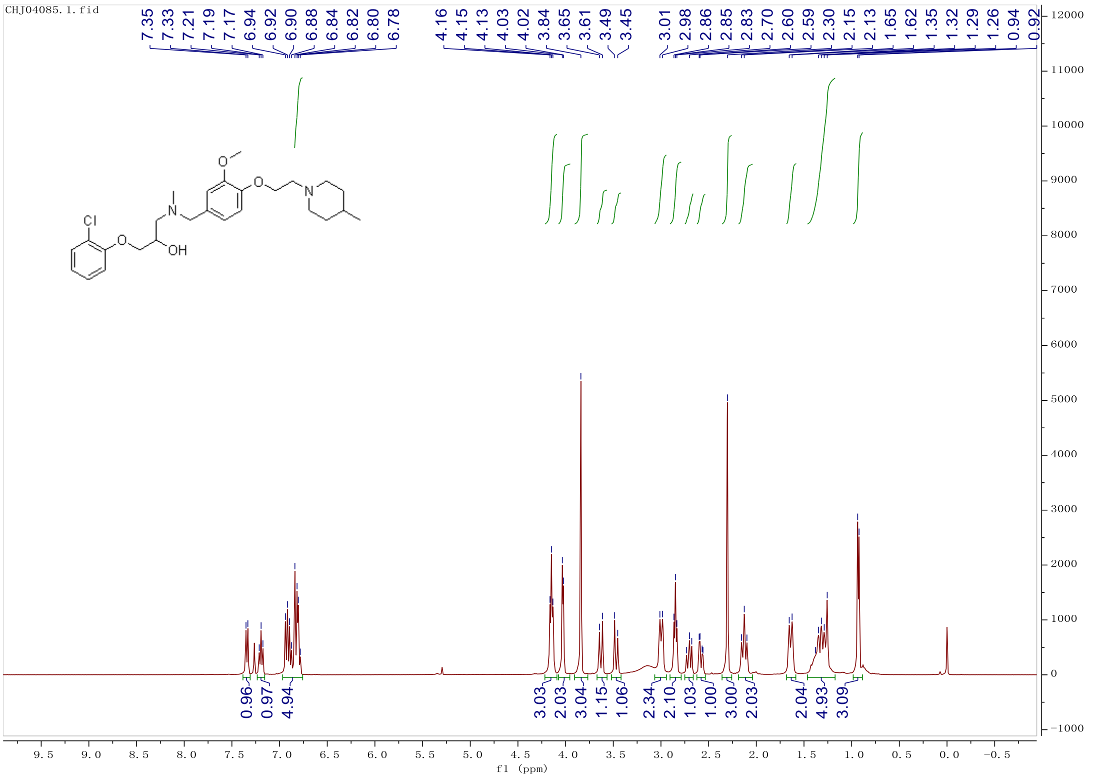

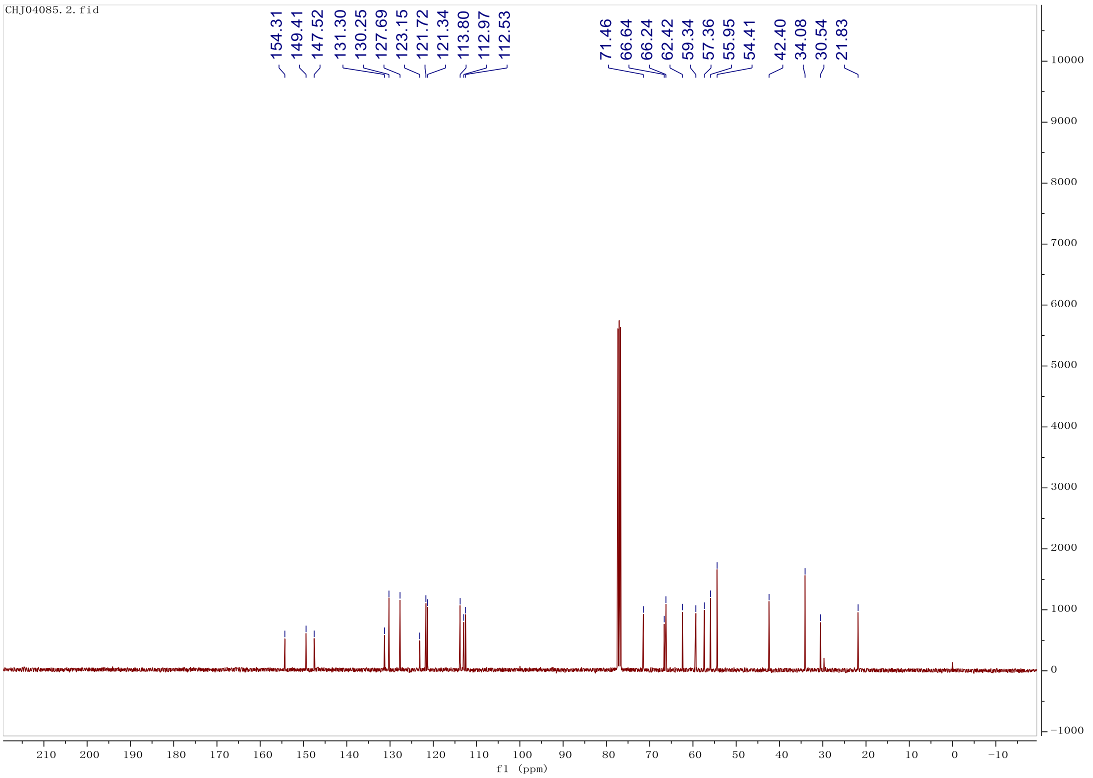

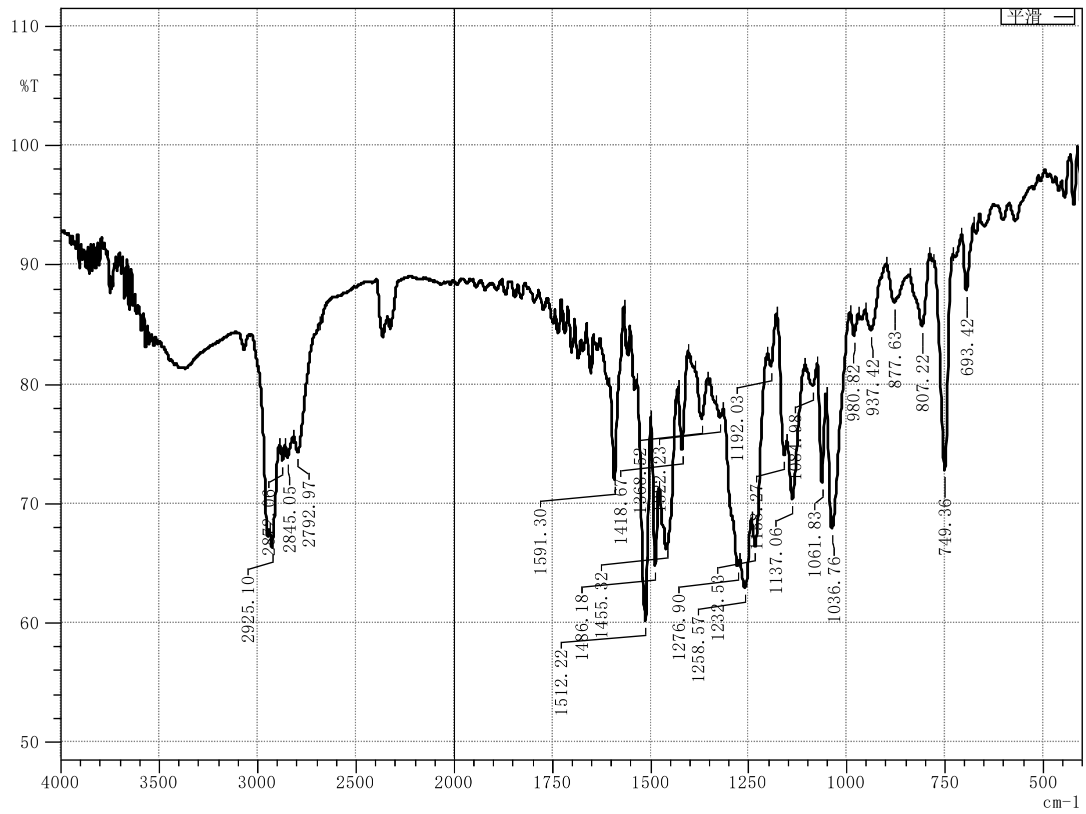

NMR (^{1}H and ^{13}C) and IR (KBr) of Compound CHJ04086:

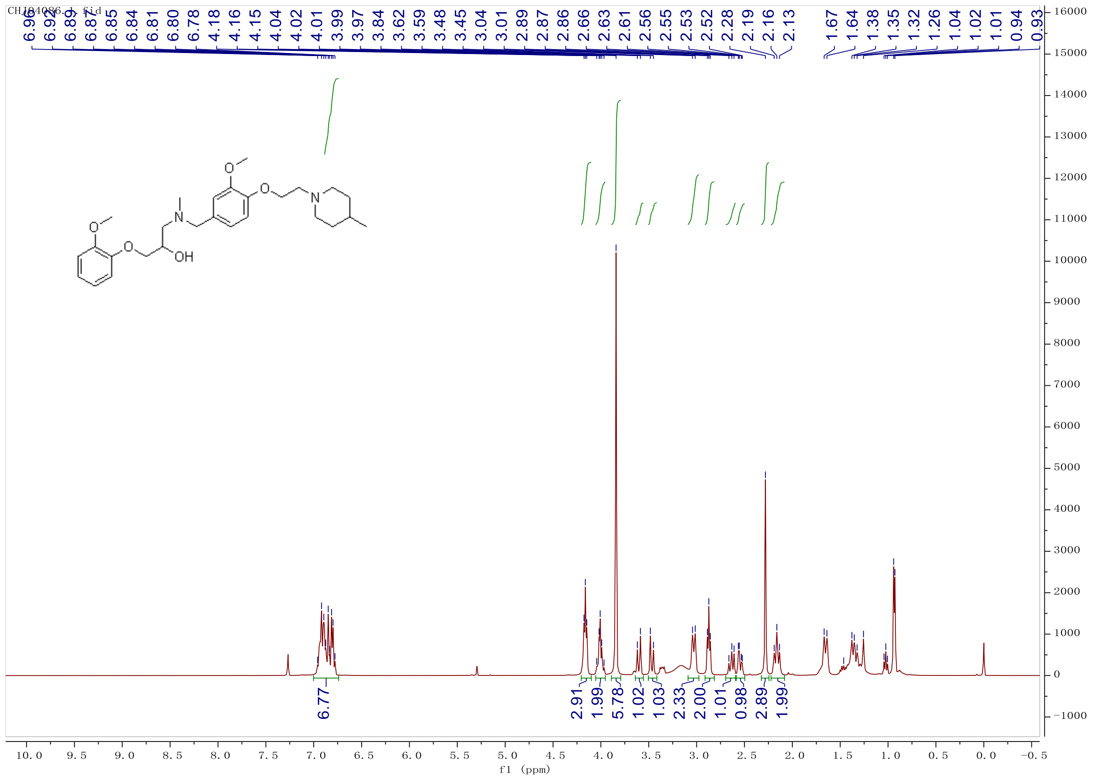

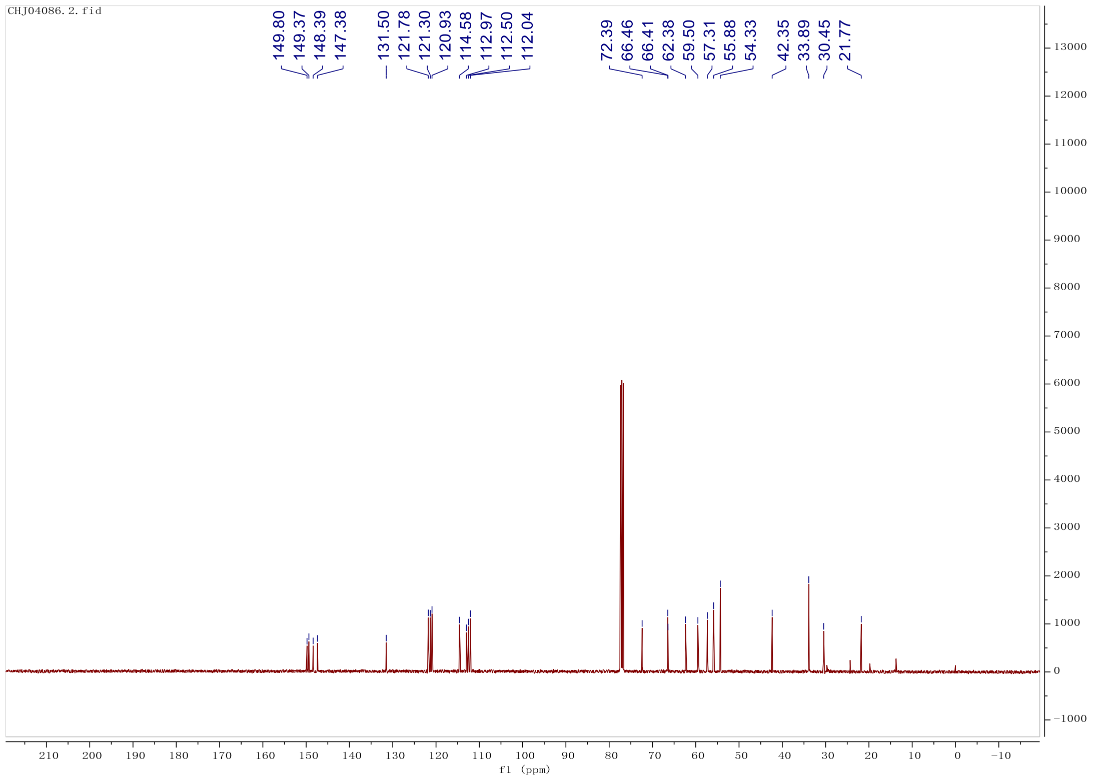
CHJ04086.2.fid
149.80
149.37
148.39
147.38
131.50
121.78
121.30
120.93
114.58
112.97
112.50
112.04
72.39
66.46
66.41
62.38
59.50
57.31
55.88
54.33
42.35
33.89
30.45
21.77
f1 (ppm)

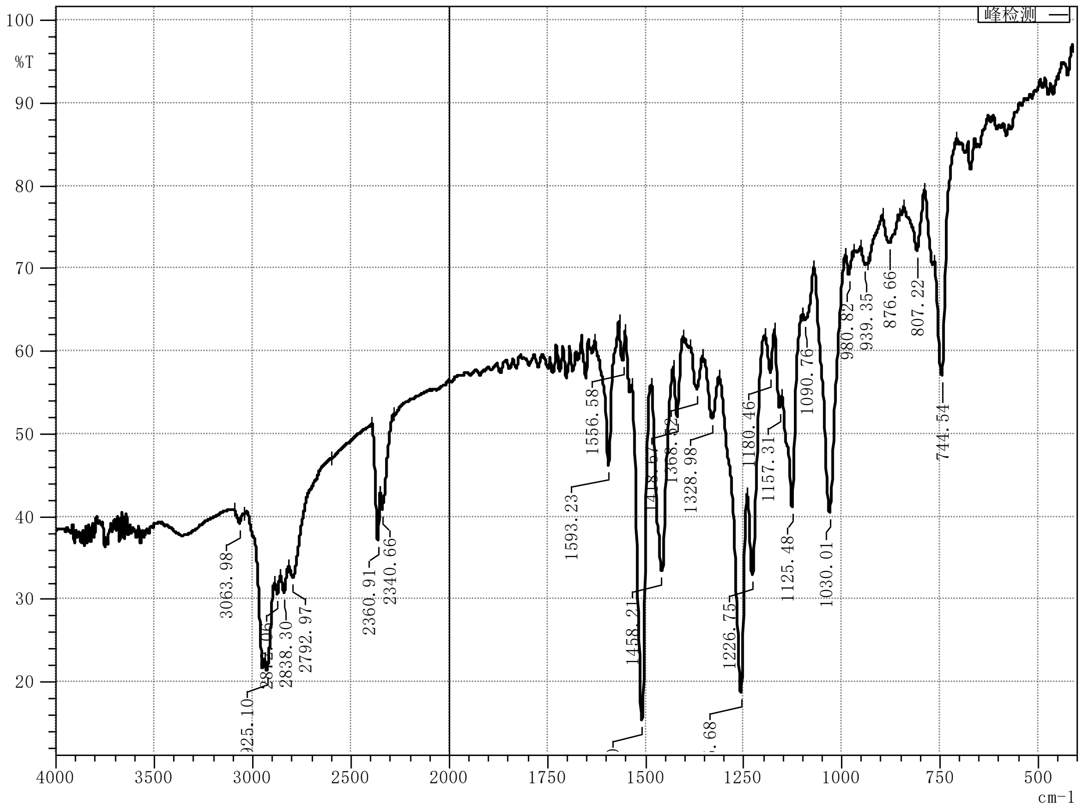
峰检测
%T
cm-1
3063.98
2838.30
2792.97
2360.91
2340.66
1593.23
1556.58
1458.21
1328.98
1226.75
1180.46
1157.31
1125.48
1090.76
1030.01
980.82
939.35
876.66
807.22
744.54

NMR (^{1}H and ^{13}C) and IR (KBr) of Compound CHJ04064:

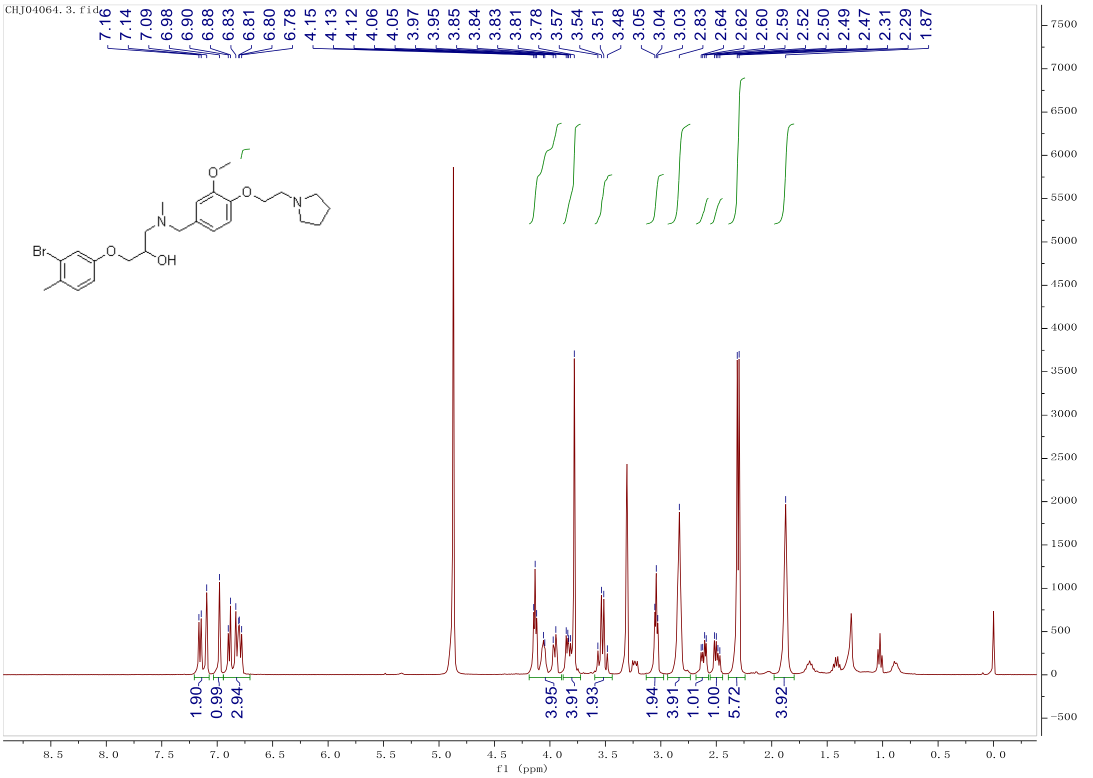

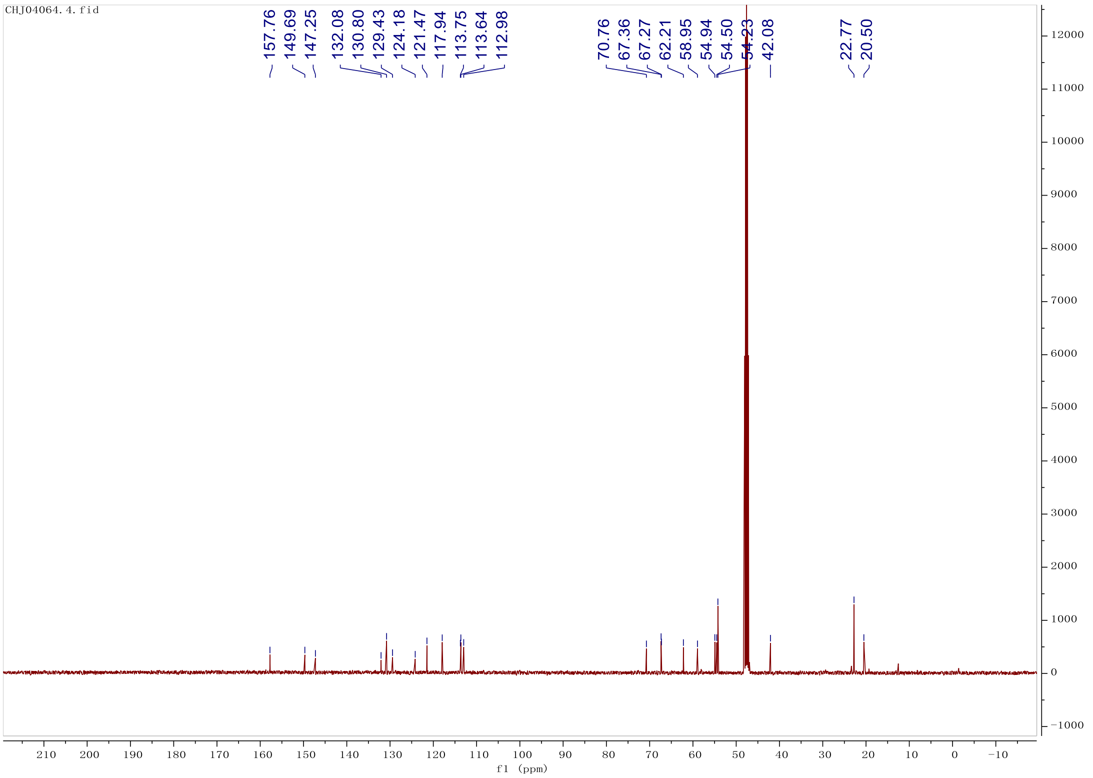

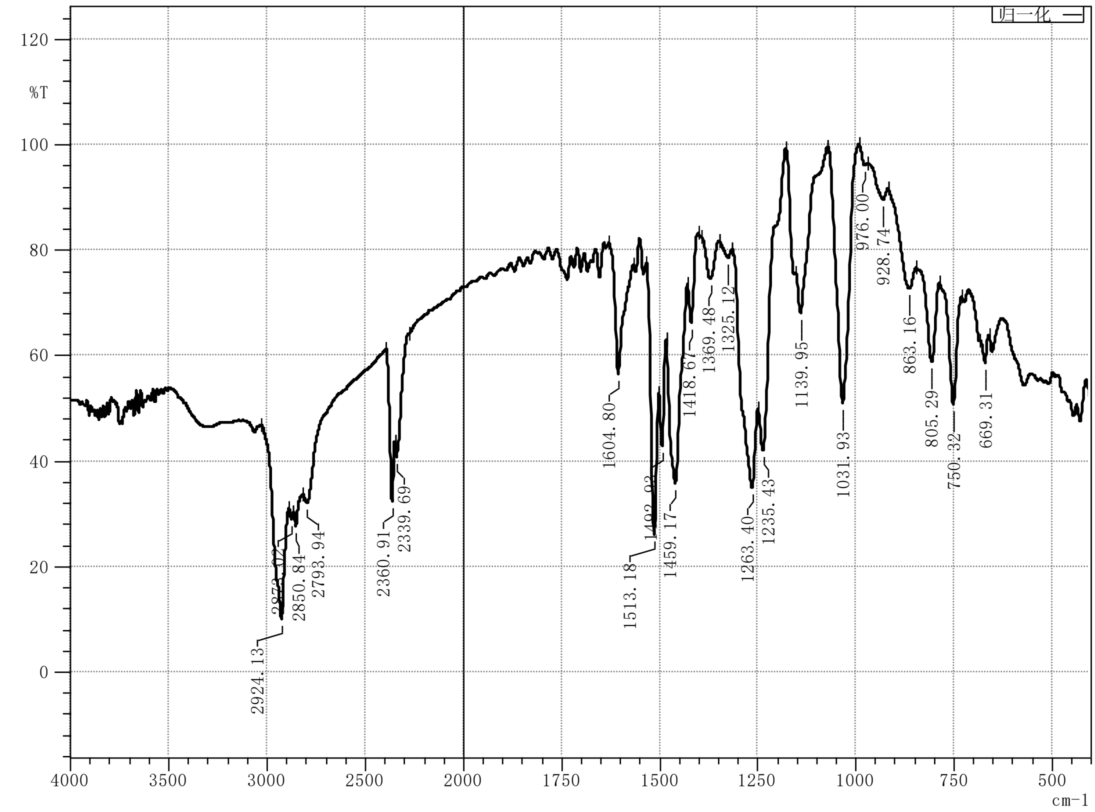

NMR (^{1}H and ^{13}C) and IR (KBr) of Compound CHJ04065:

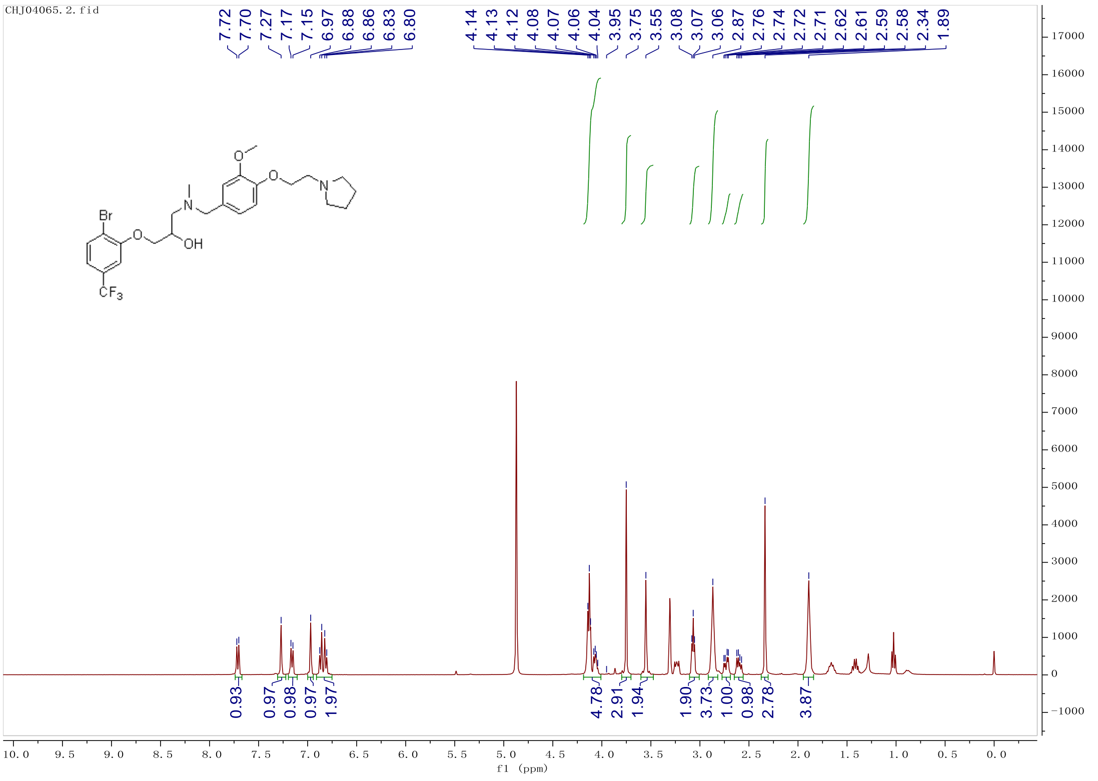

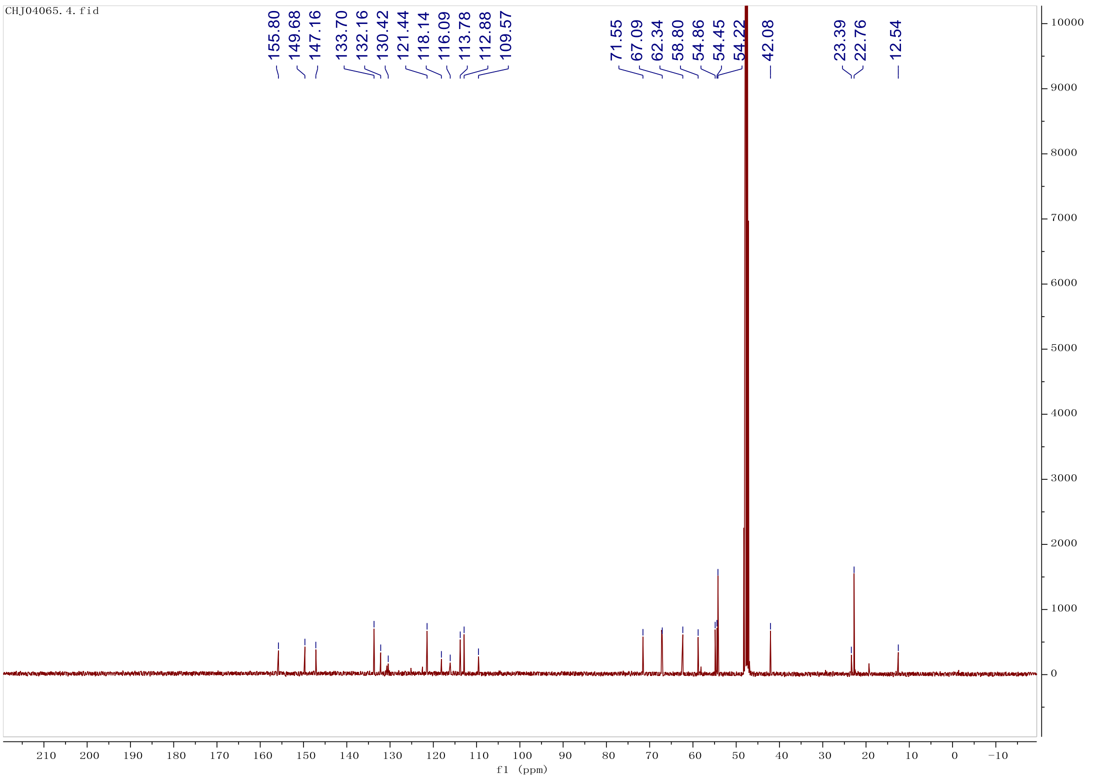
CHJ04065.4.fid
155.80
149.68
147.16
133.70
132.16
130.42
121.44
118.14
116.09
113.78
112.88
109.57
71.55
67.09
62.34
58.80
54.86
54.45
54.22
42.08
23.39
22.76
12.54
f1 (ppm)

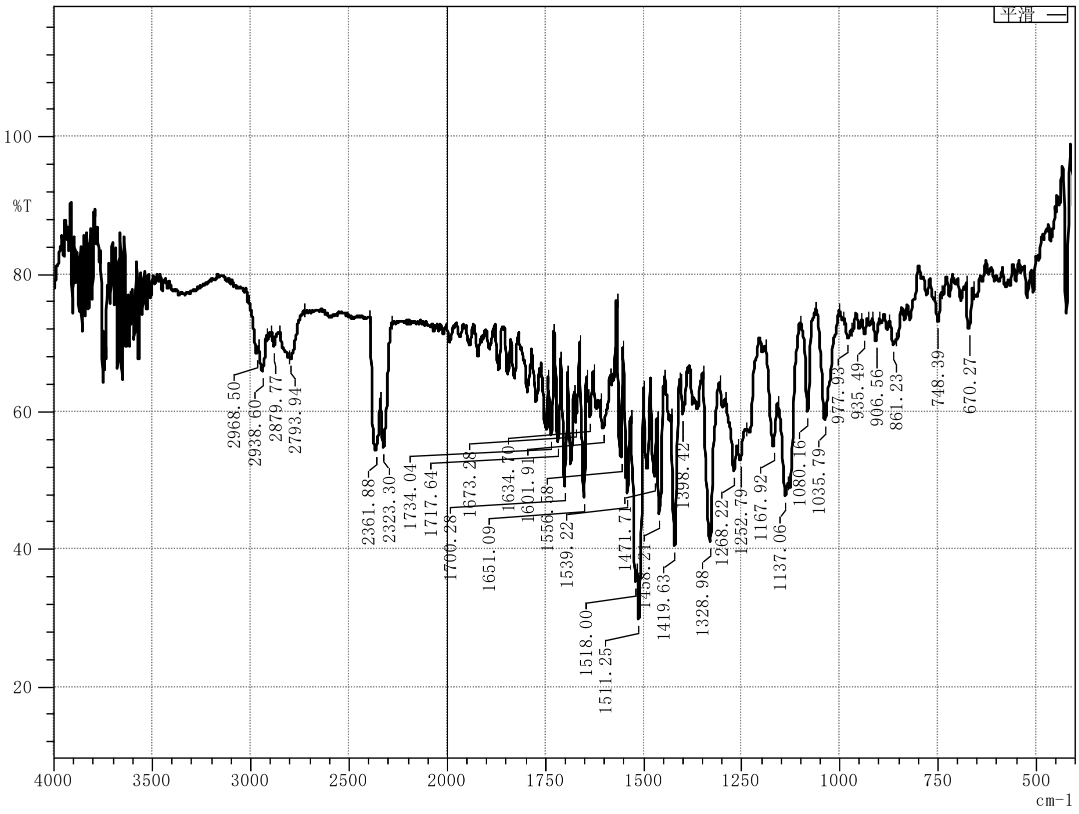
平滑
%T
cm-1
2968.50
2938.60
2879.77
2793.94
2361.88
2323.30
1734.04
1717.64
1700.28
1673.28
1651.09
1634.70
1601.91
1556.58
1539.22
1518.00
1511.25
1471.71
1458.21
1419.63
1398.42
1328.98
1268.22
1252.79
1167.92
1137.06
1080.16
1035.79
977.93
935.49
906.56
861.23
748.39
670.27

NMR (^{1}H and ^{13}C) and IR (KBr) of Compound CHJ04066:

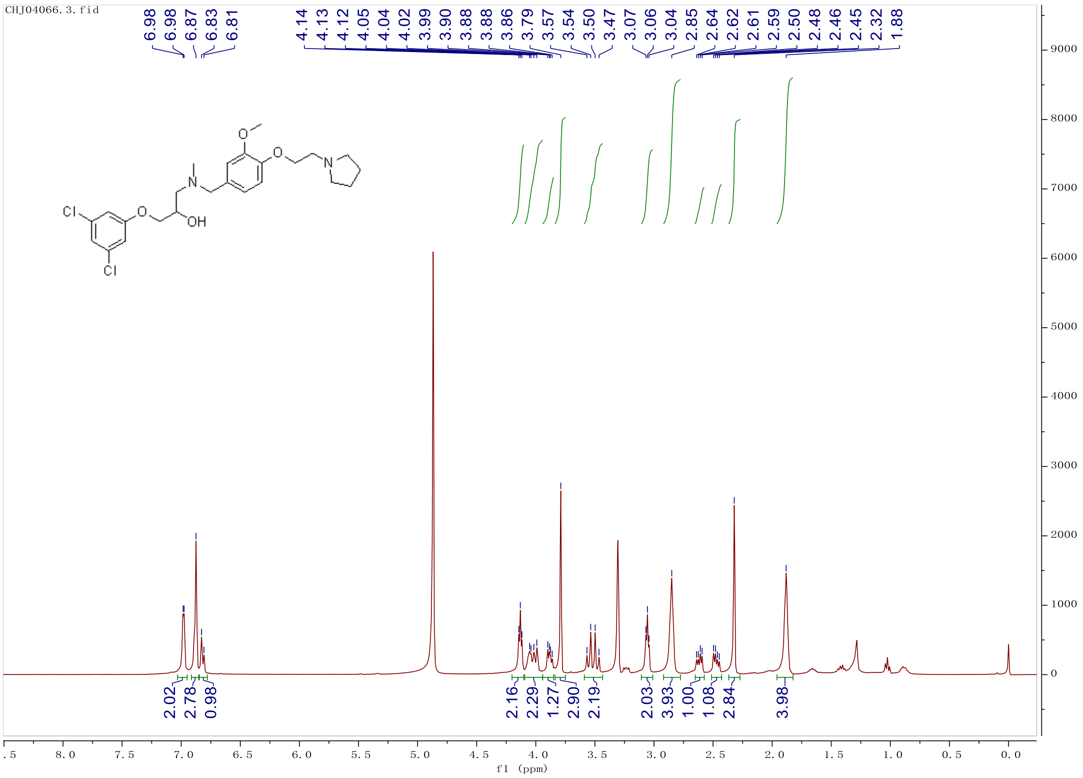

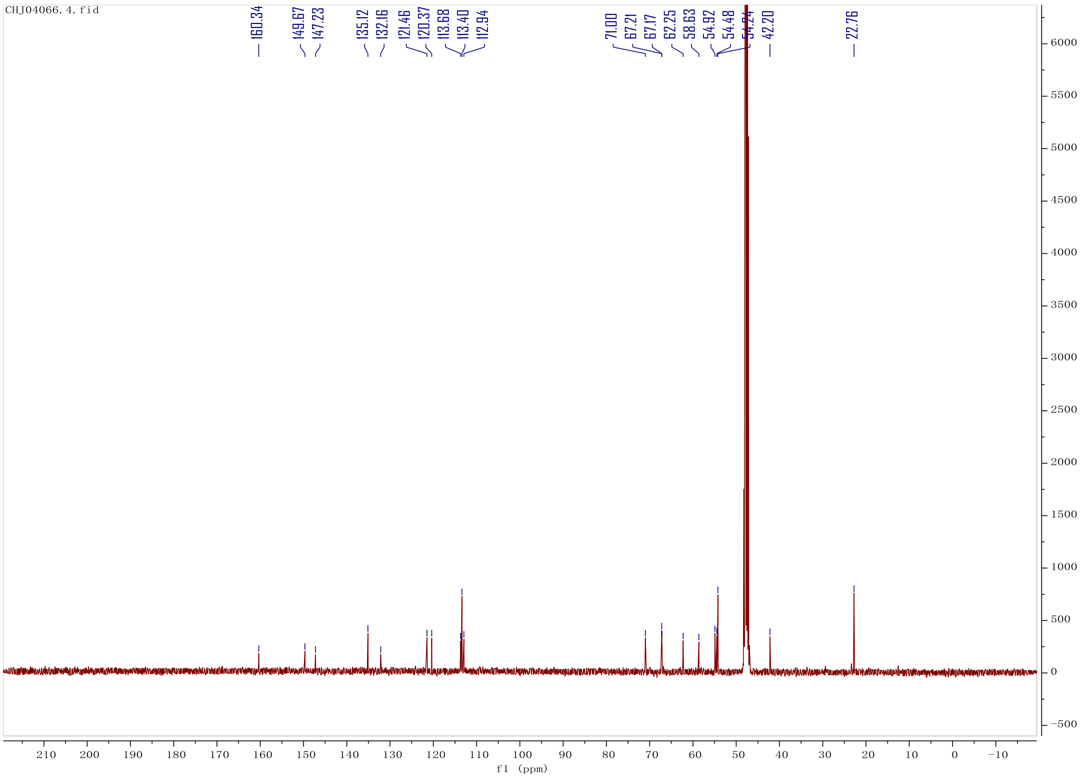

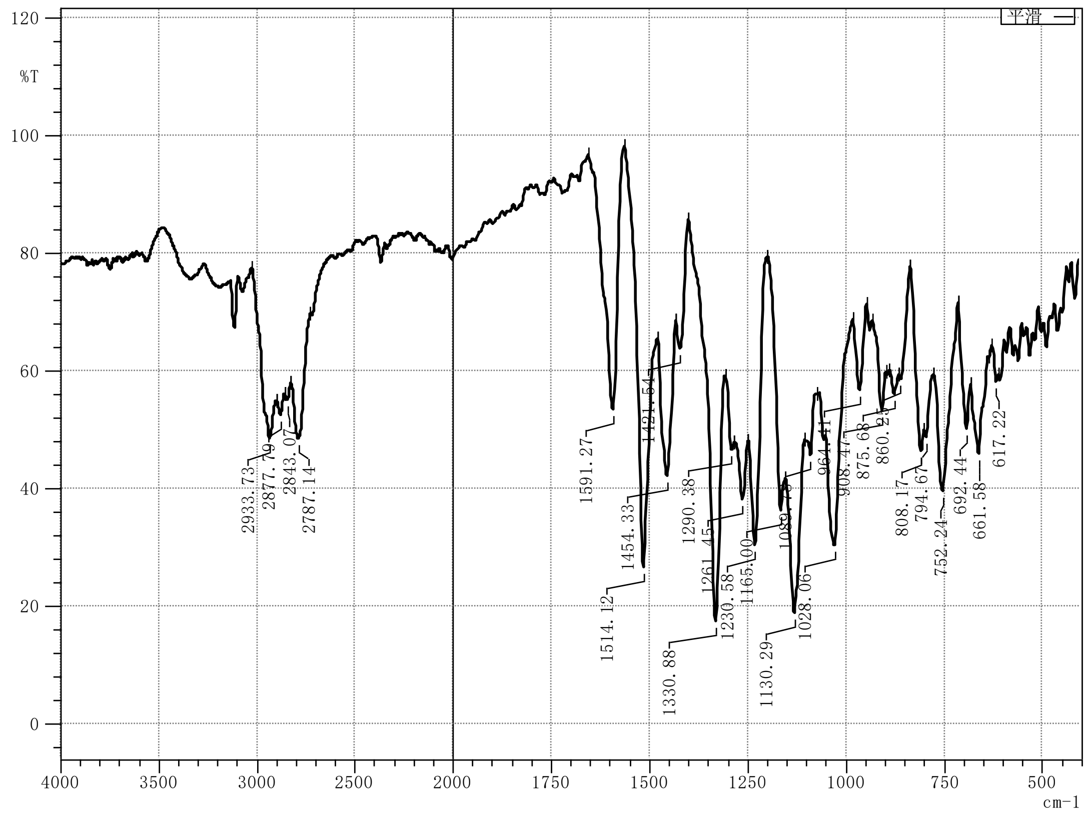

NMR (^{1}H and ^{13}C) and IR (KBr) of Compound CHJ04068:

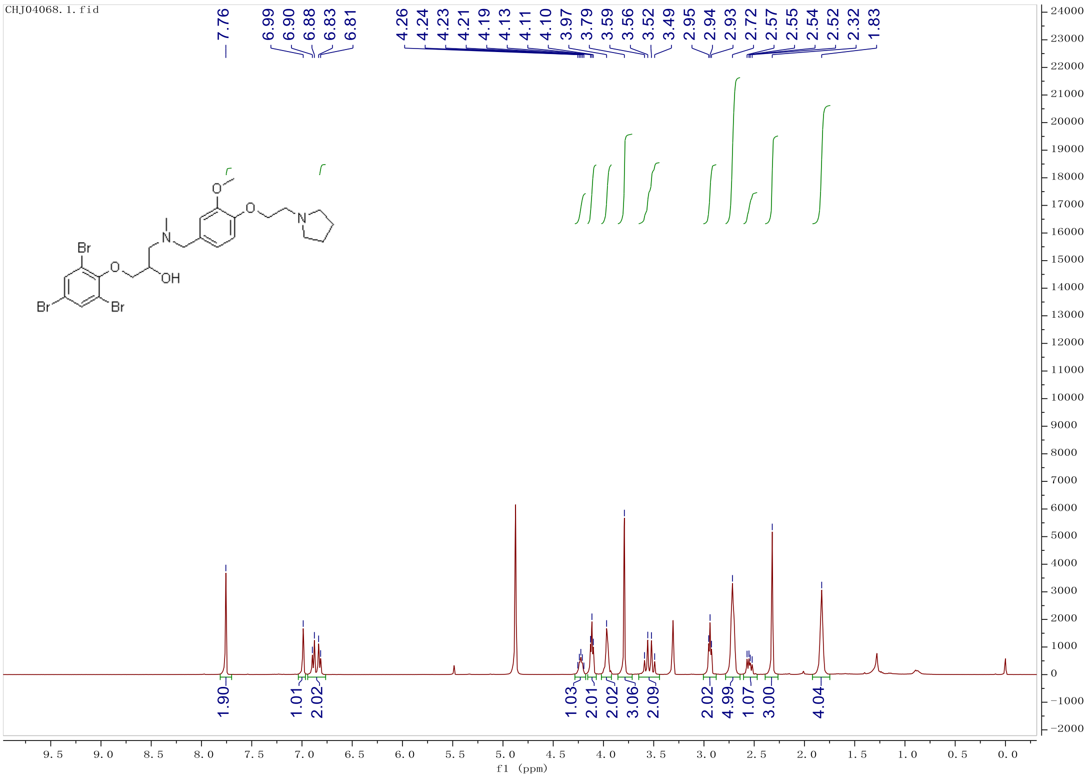

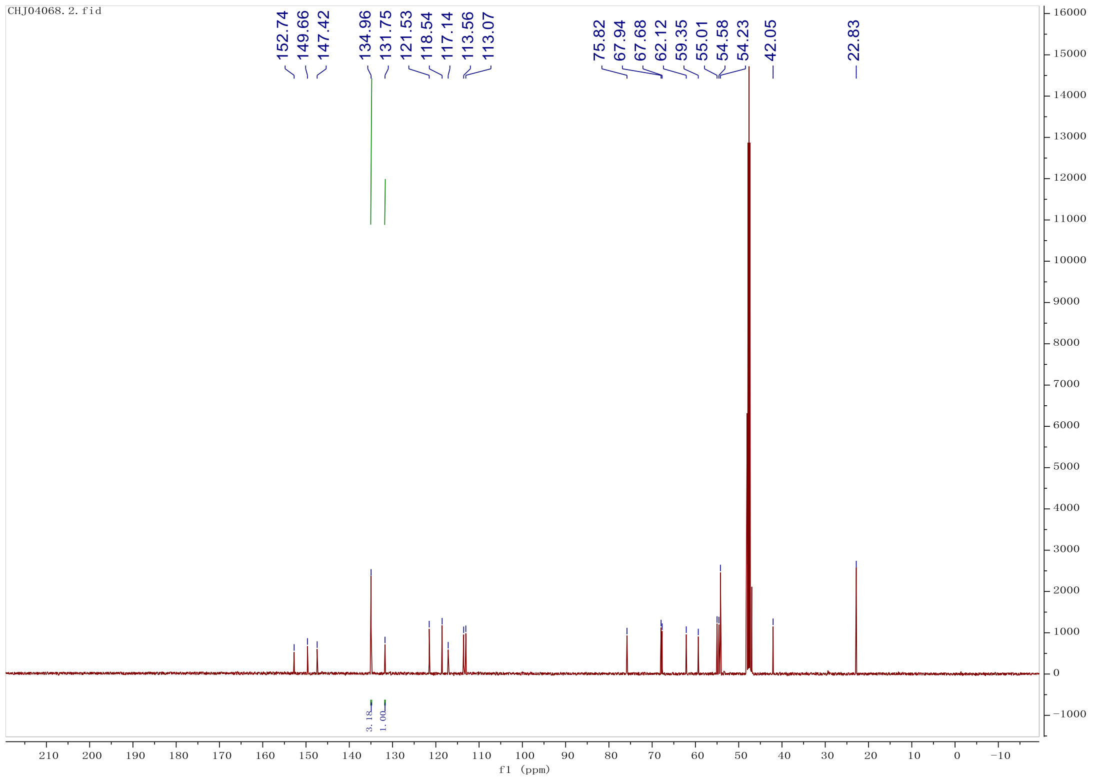

CHJ04068.2.fid
152.74
149.66
147.42
134.96
131.75
121.53
118.54
117.14
113.56
113.07
75.82
67.94
67.68
62.12
59.35
55.01
54.58
54.23
42.05
22.83
3.18
1.00
f1 (ppm)

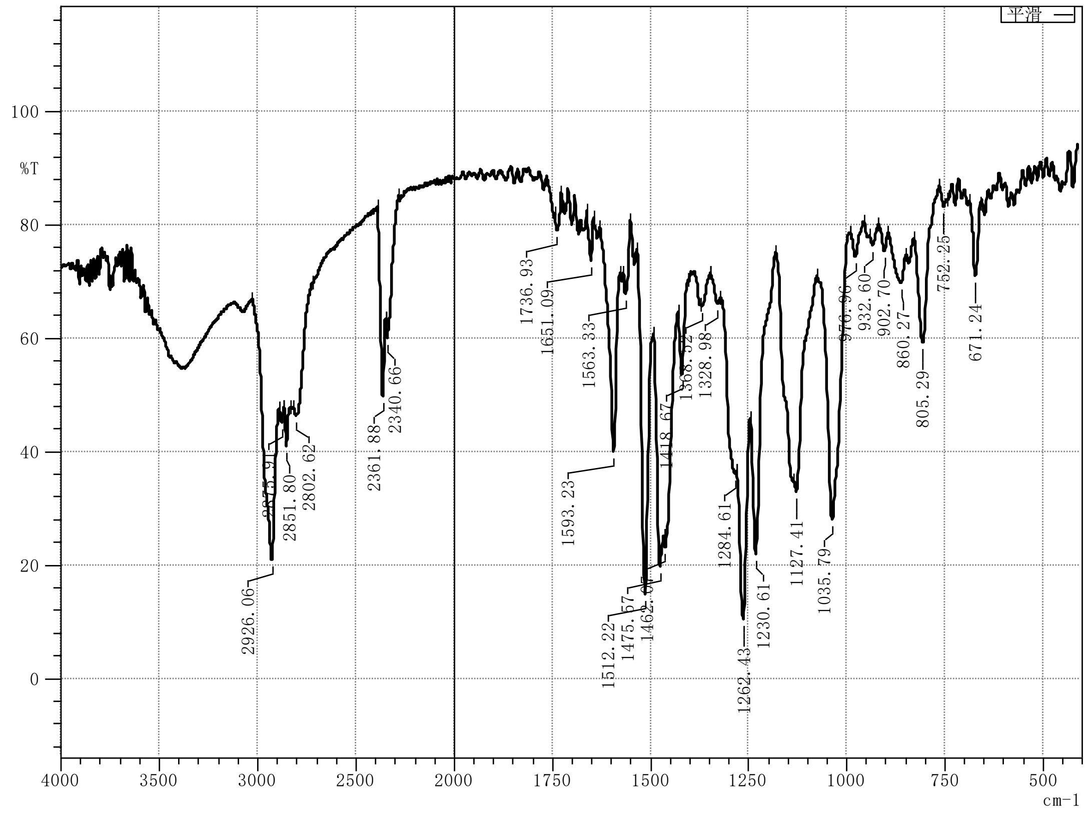

平滑
%T
2926.06
2851.80
2802.62
2361.88
2340.66
1736.93
1651.09
1563.33
1593.23
1512.22
1475.57
1328.98
1284.61
1262.43
1230.61
1127.41
1035.79
932.60
902.70
860.27
805.29
752.25
671.24
cm-1

NMR (^{1}H and ^{13}C) and IR (KBr) of Compound CHJ04072:

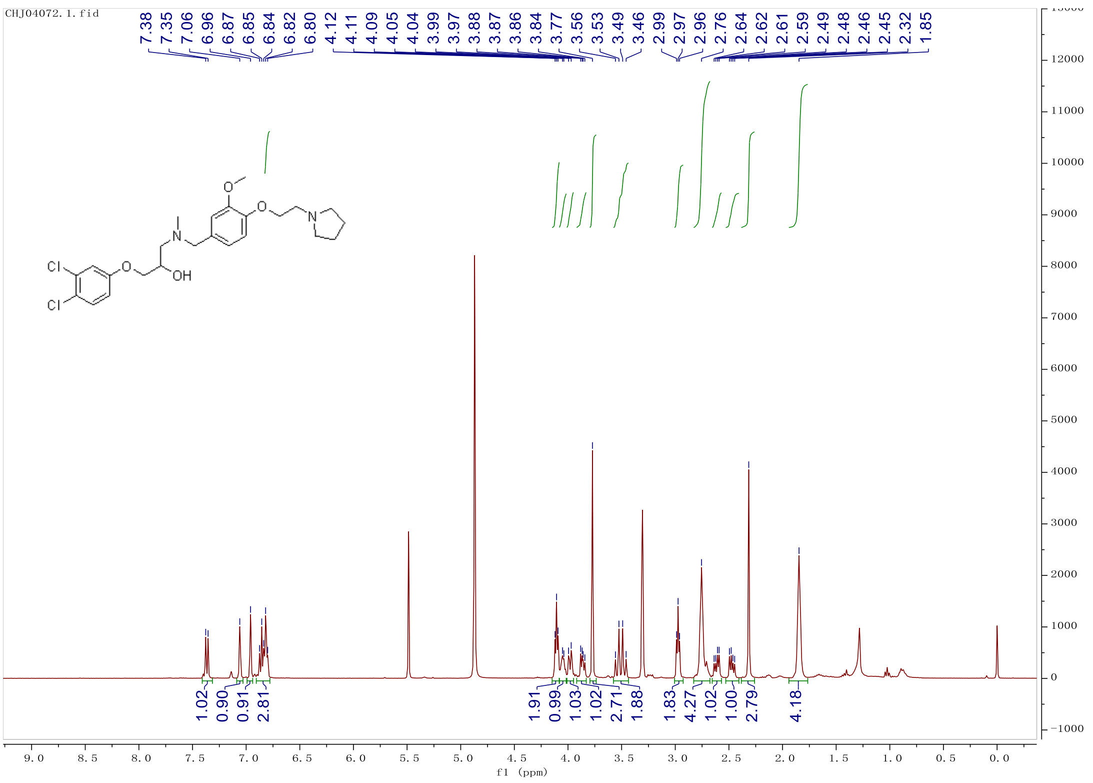

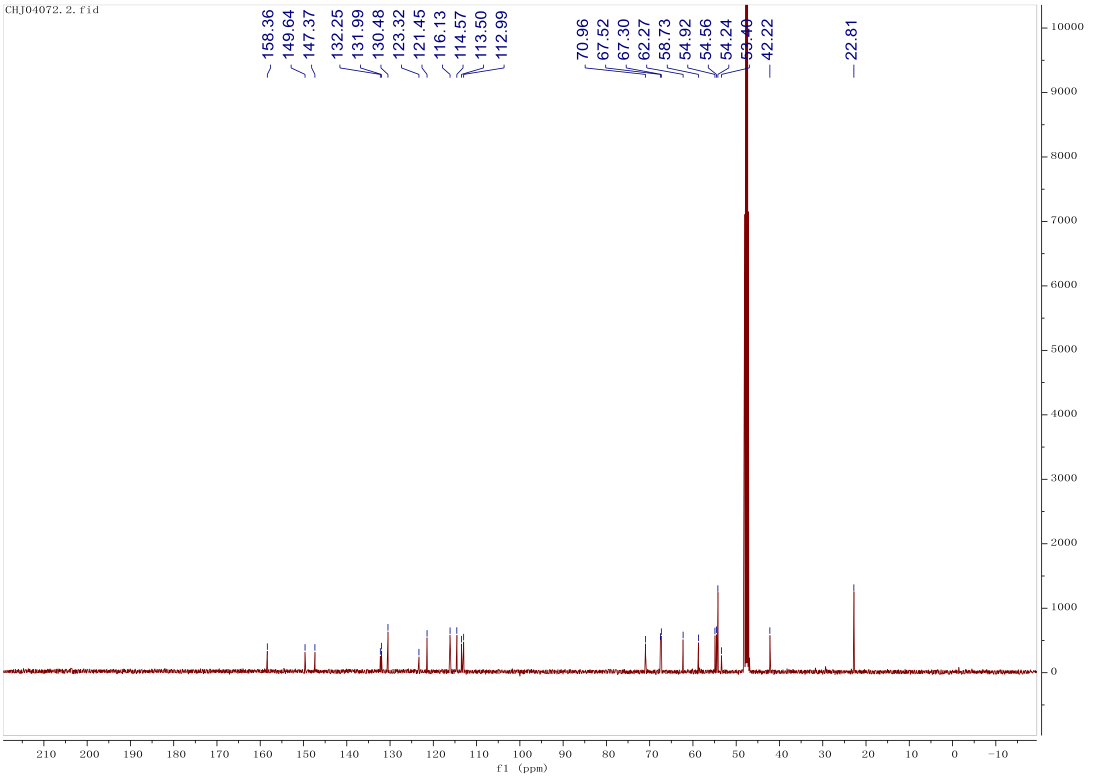

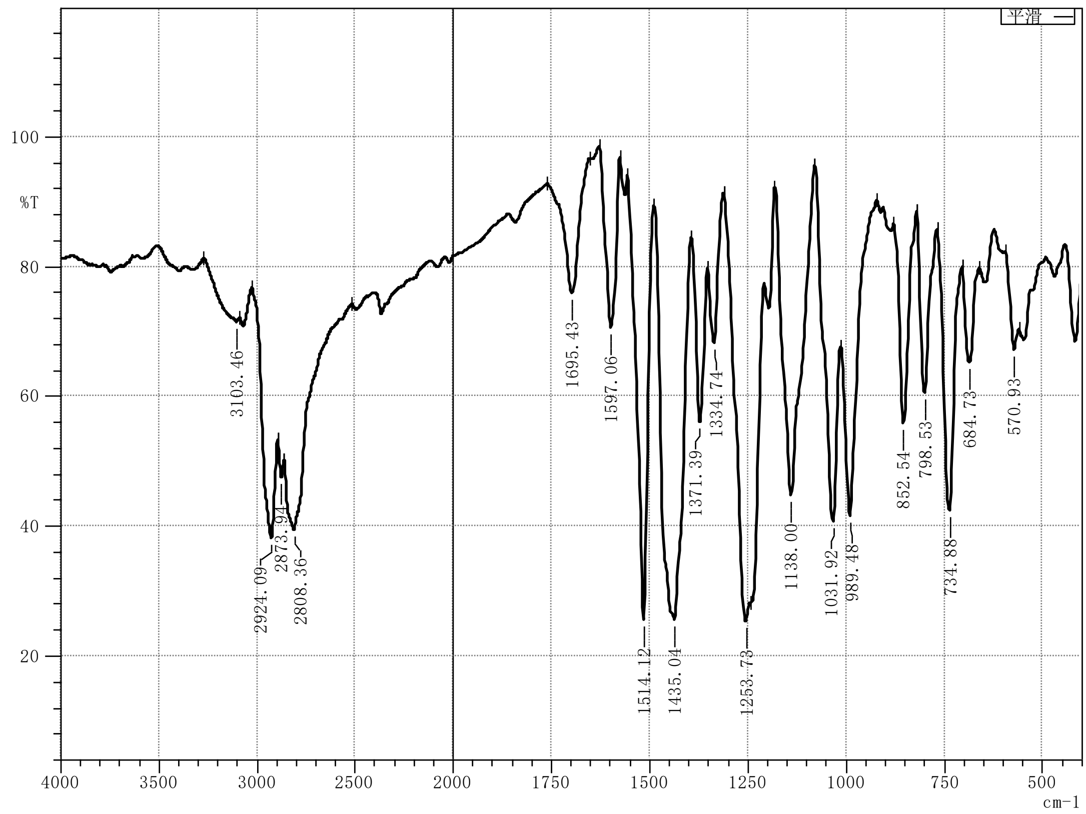

NMR (^{1}H and ^{13}C) and IR (KBr) of Compound CHJ04089:

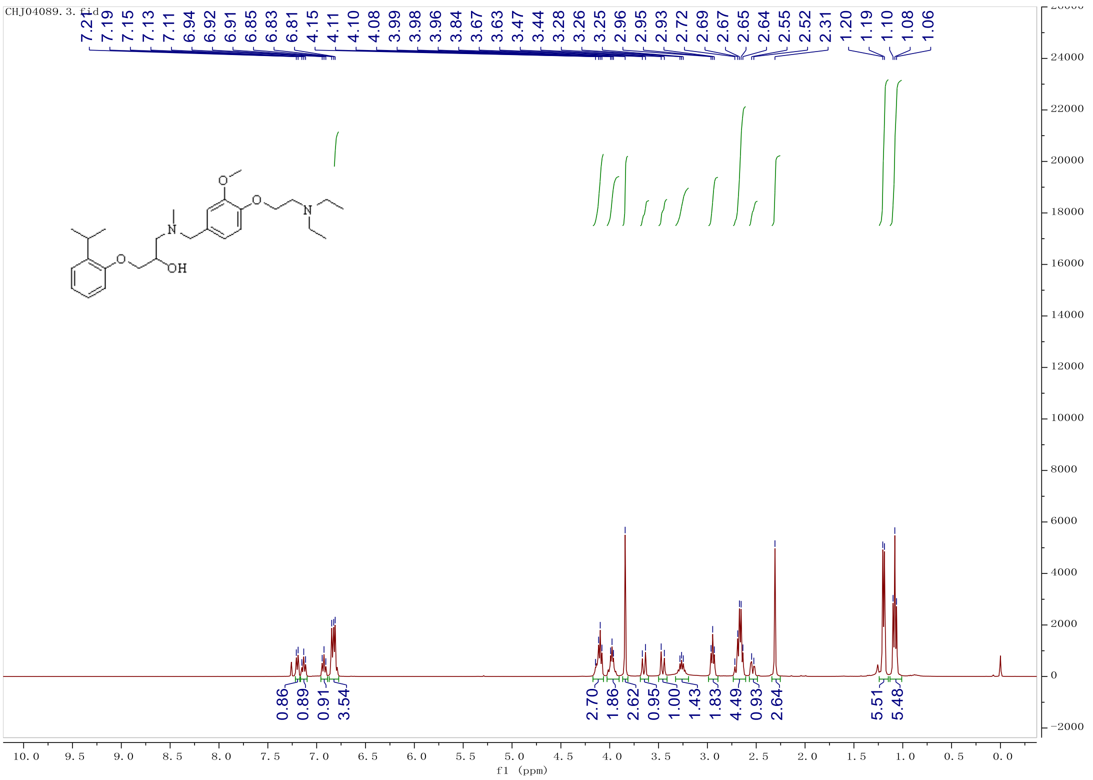

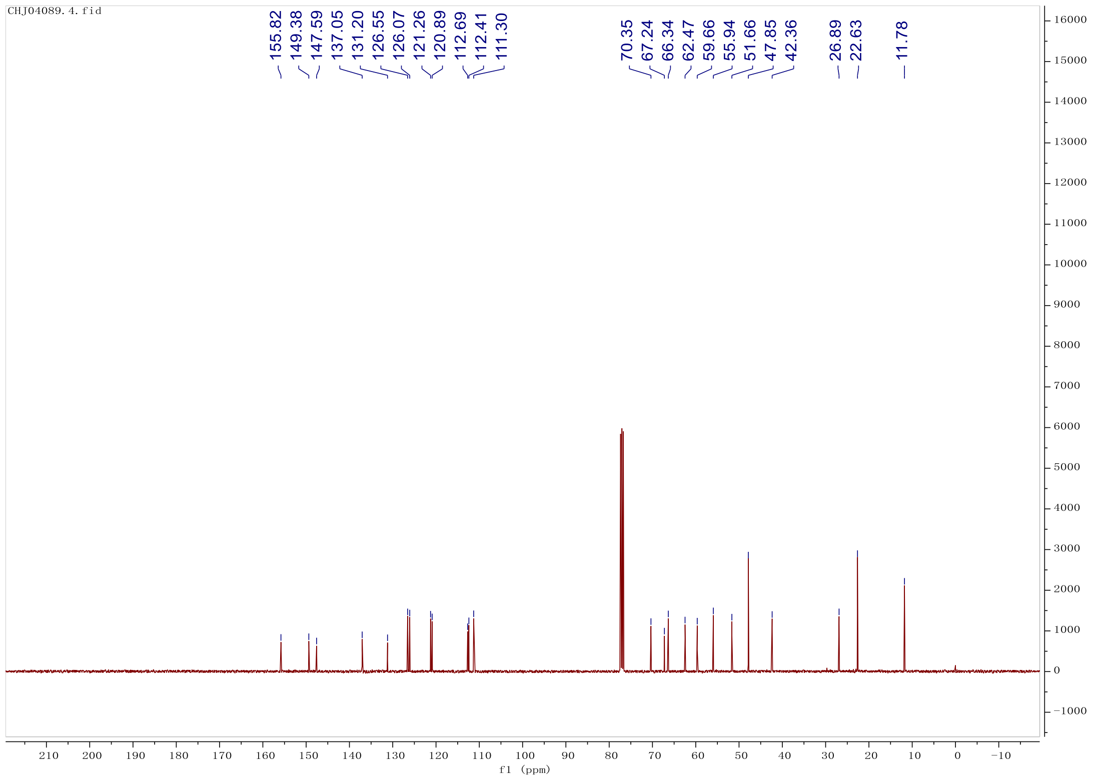
CHJ04089.4.fid
155.82
149.38
147.59
137.05
131.20
126.55
126.07
121.26
120.89
112.69
112.41
111.30
70.35
67.24
66.34
62.47
59.66
55.94
51.66
47.85
42.36
26.89
22.63
11.78
f1 (ppm)

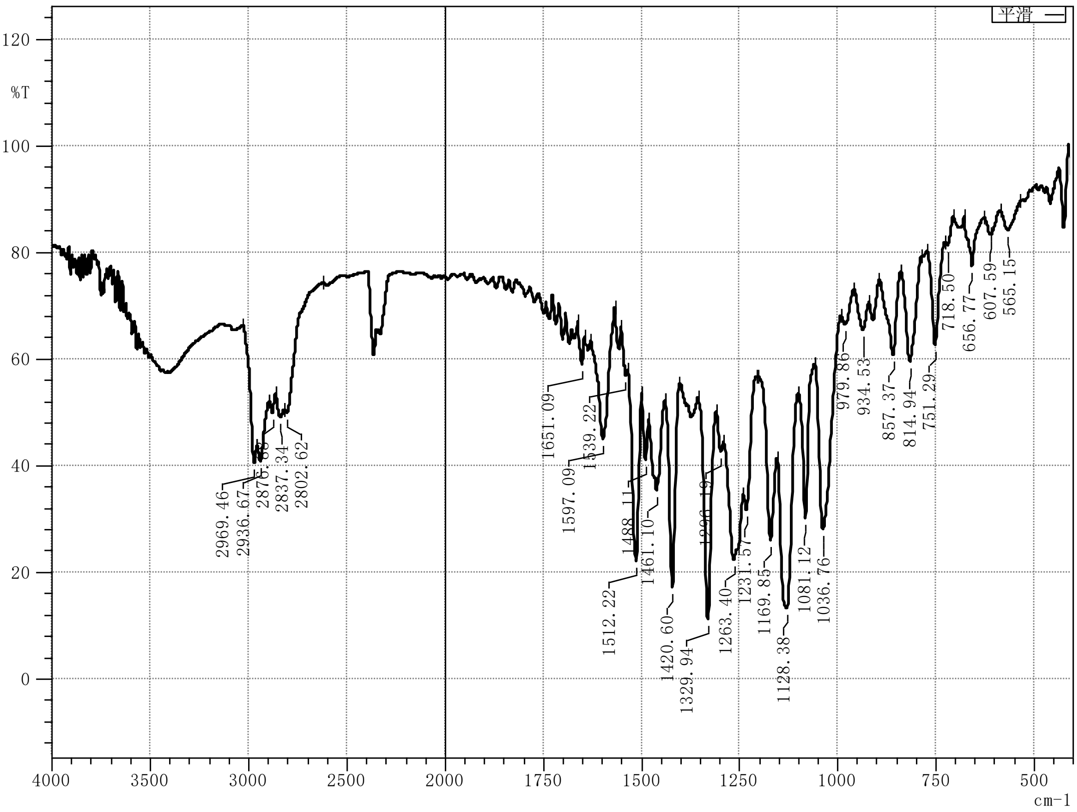
%T
cm-1
2969.46
2936.67
2837.34
2802.62
1651.09
1597.09
1512.22
1461.10
1420.60
1329.94
1263.40
1231.57
1169.85
1128.38
1081.12
1036.76
979.86
934.53
857.37
814.94
751.29
718.50
656.77
607.59
565.15

NMR (^{1}H and ^{13}C) and IR (KBr) of Compound CHJ04090:

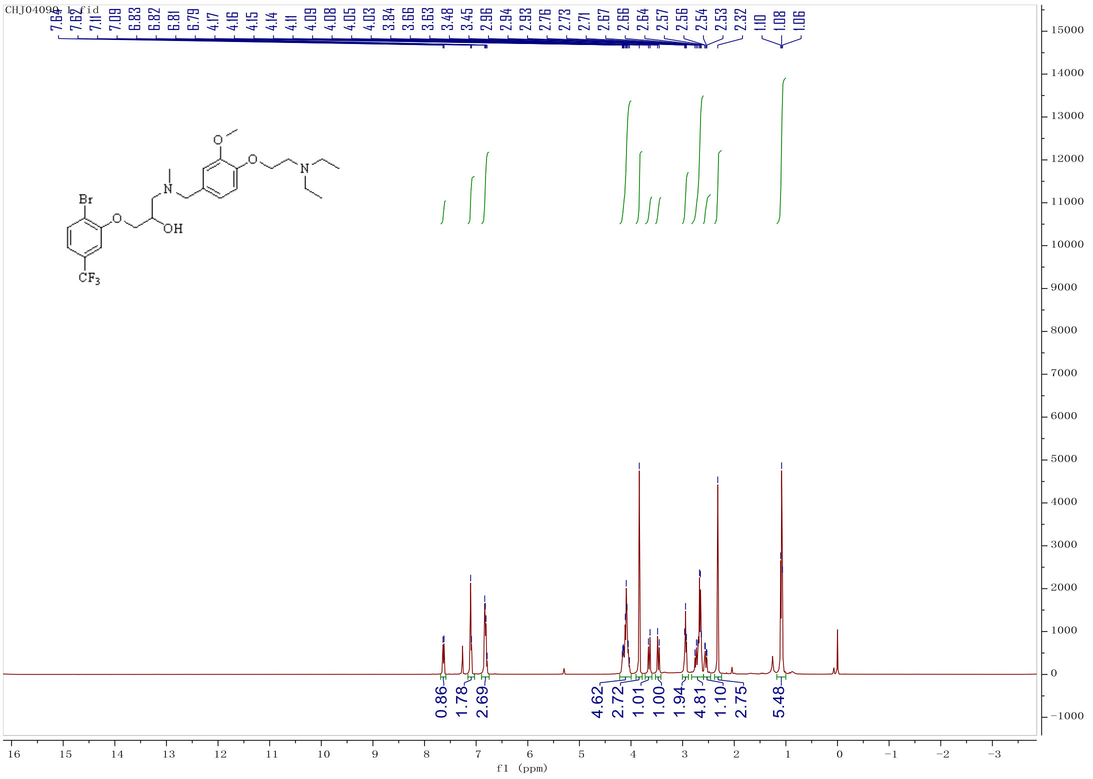

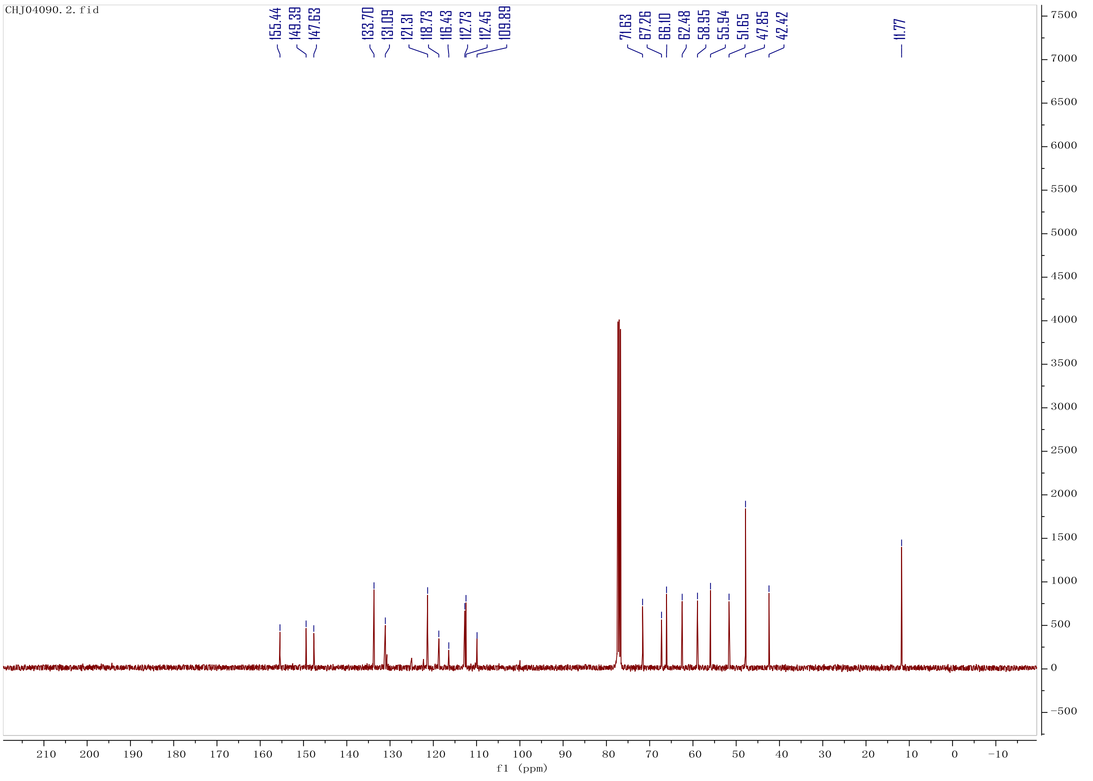

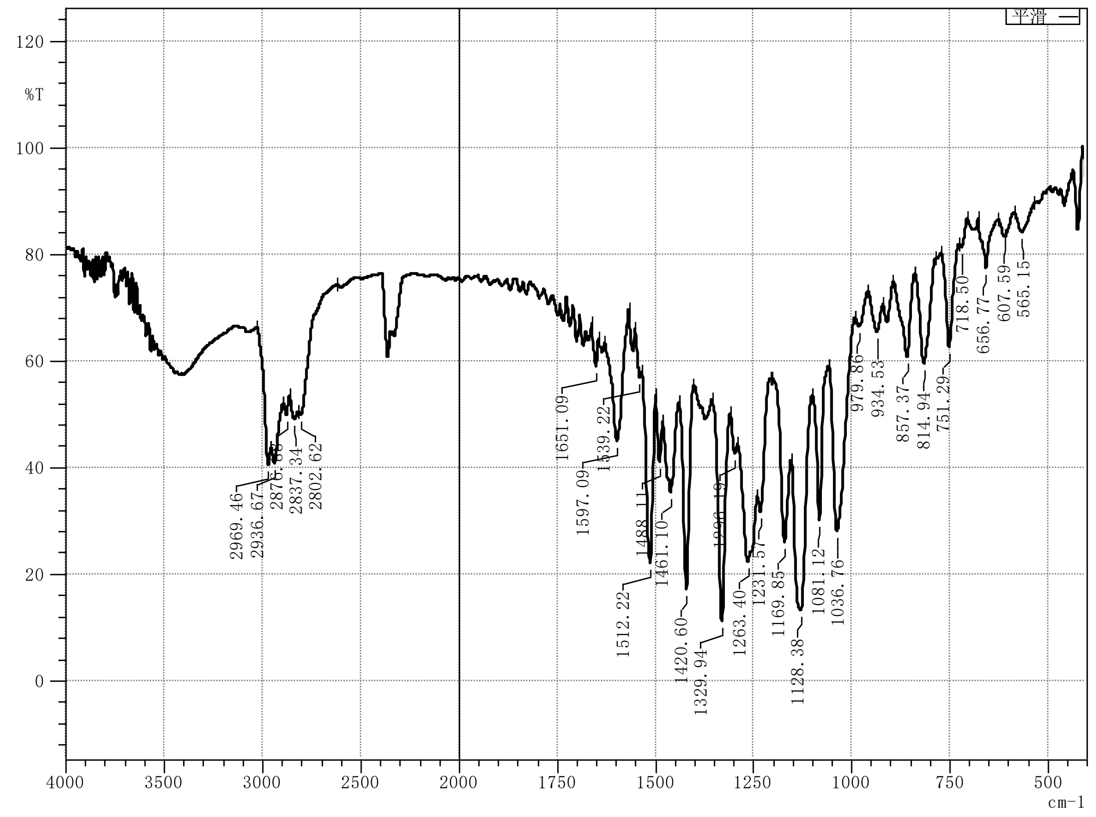

NMR (^{1}H and ^{13}C) and IR (KBr) of Compound CHJ04091:

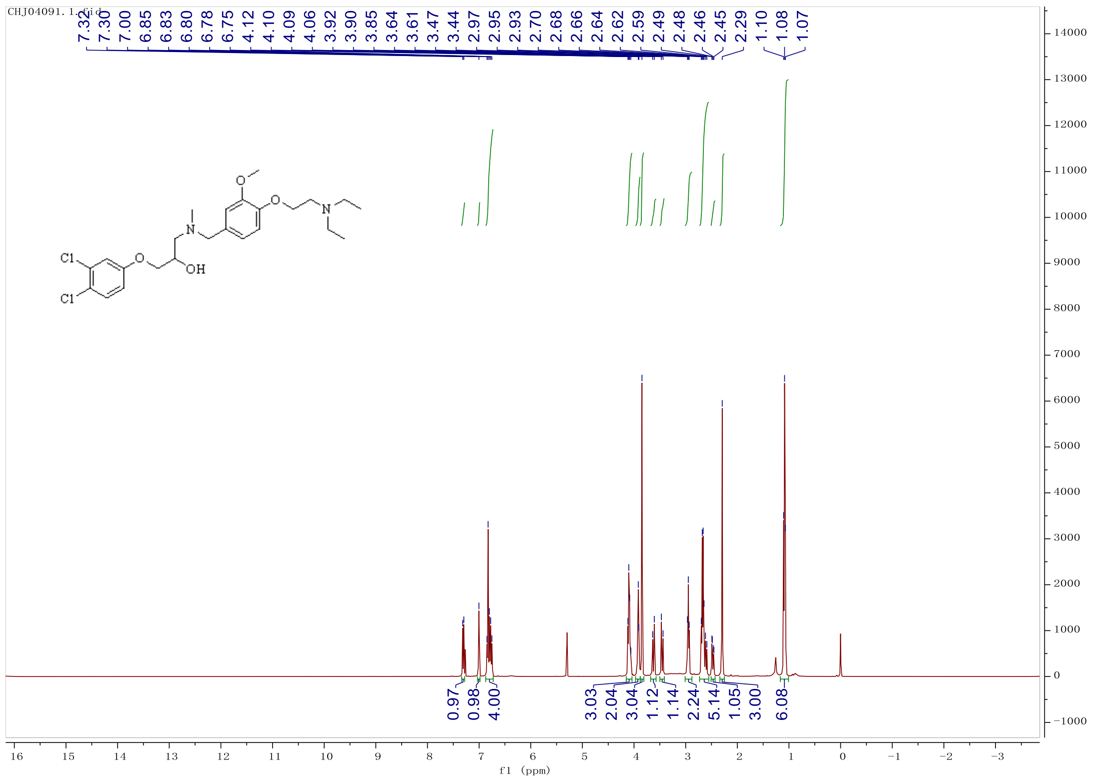

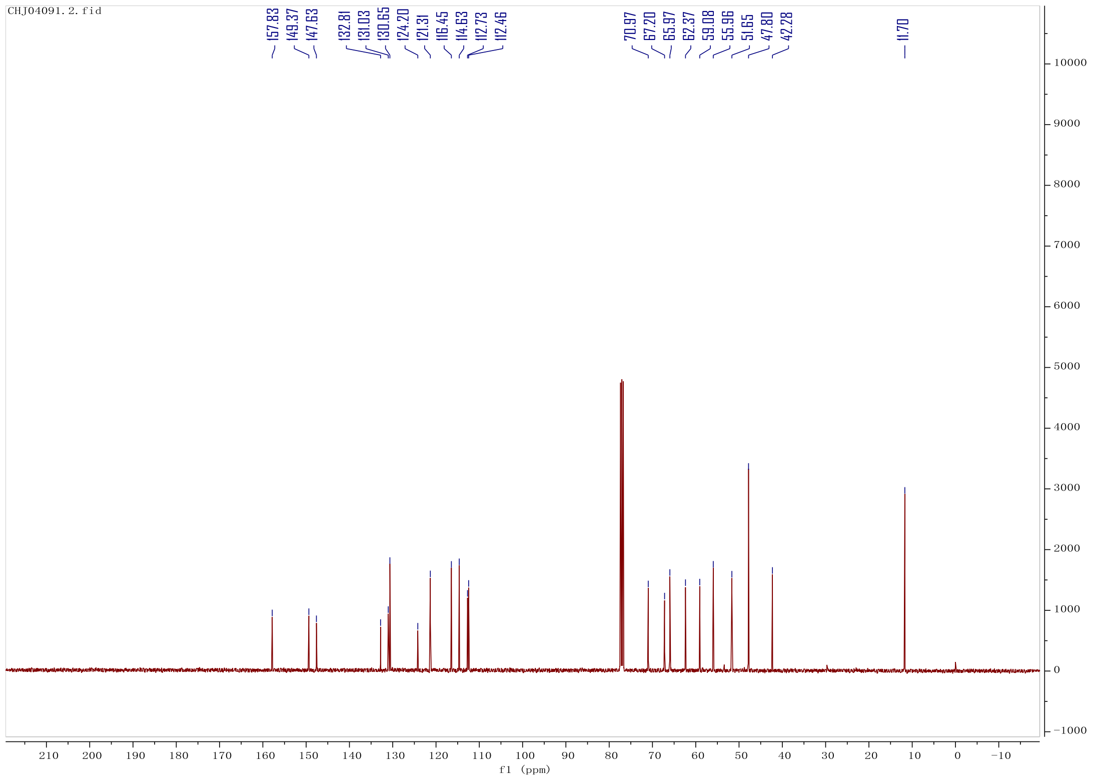
CHJ04091.2.fid
157.83
149.37
147.63
132.81
131.03
130.65
124.20
121.31
116.45
114.63
112.73
112.46
70.97
67.20
65.97
62.37
59.08
55.96
51.65
47.80
42.28
11.70
f1 (ppm)

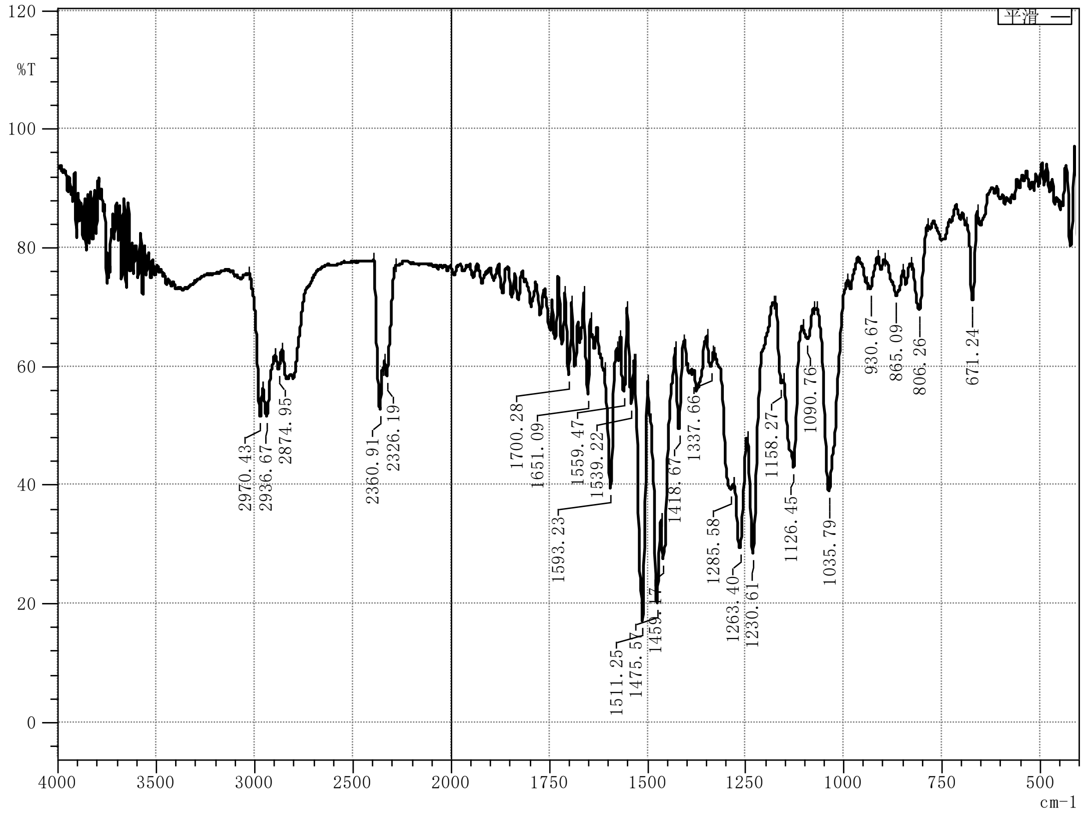
%T
平滑
2970.43
2936.67
2874.95
2360.91
2326.19
1700.28
1651.09
1559.47
1539.22
1593.23
1511.25
1475.57
1418.67
1337.66
1285.58
1263.40
1230.61
1158.27
1126.45
1090.76
1035.79
930.67
865.09
806.26
671.24
cm-1

NMR (^{1}H and ^{13}C) and IR (KBr) of Compound CHJ04092:

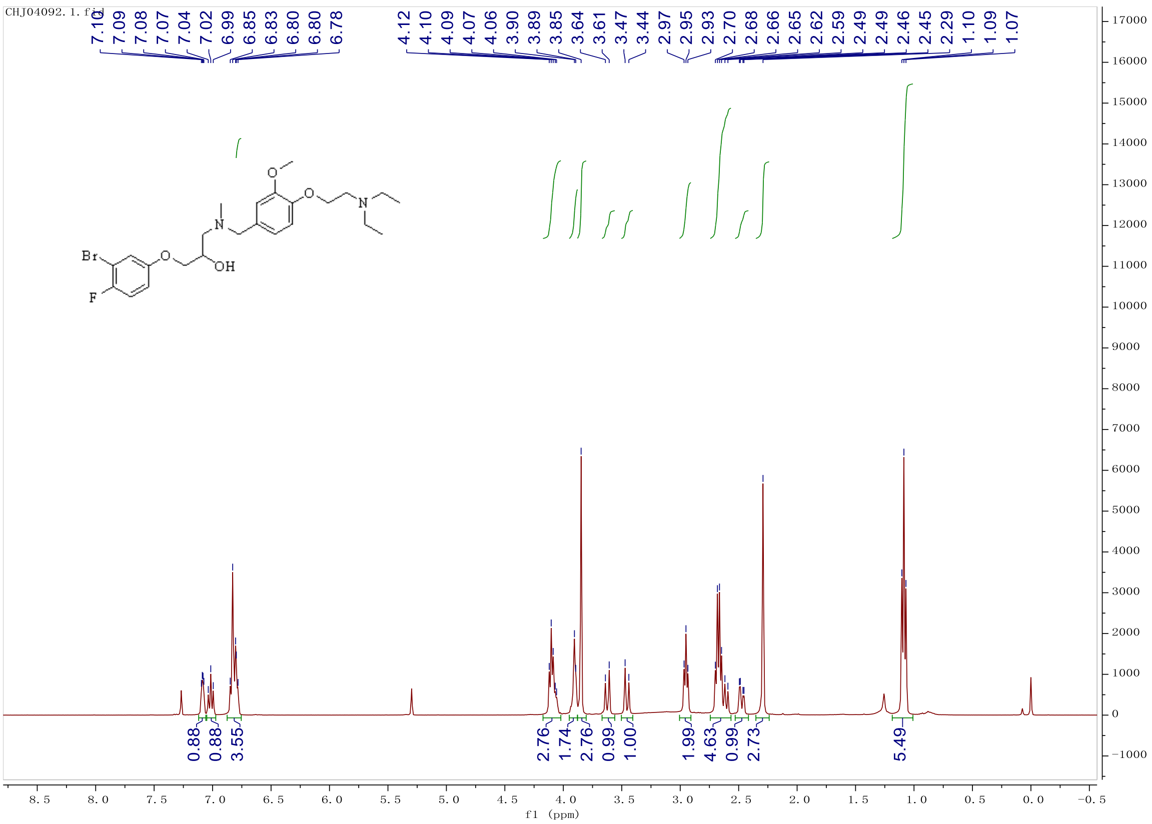

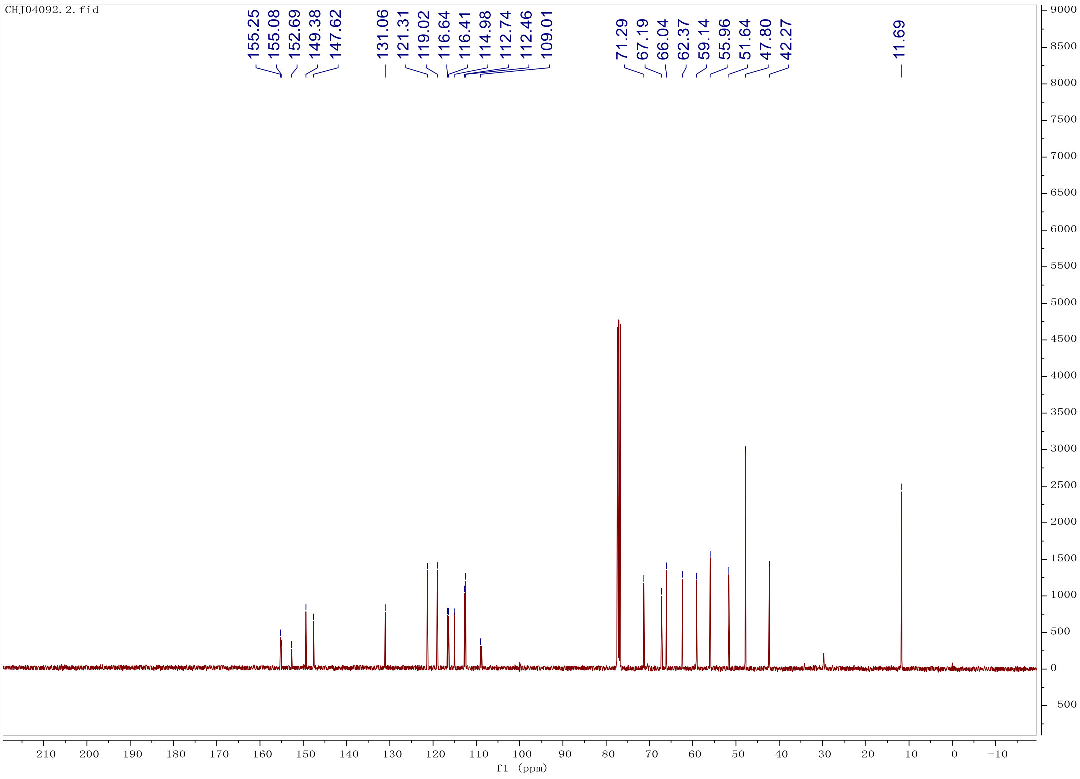

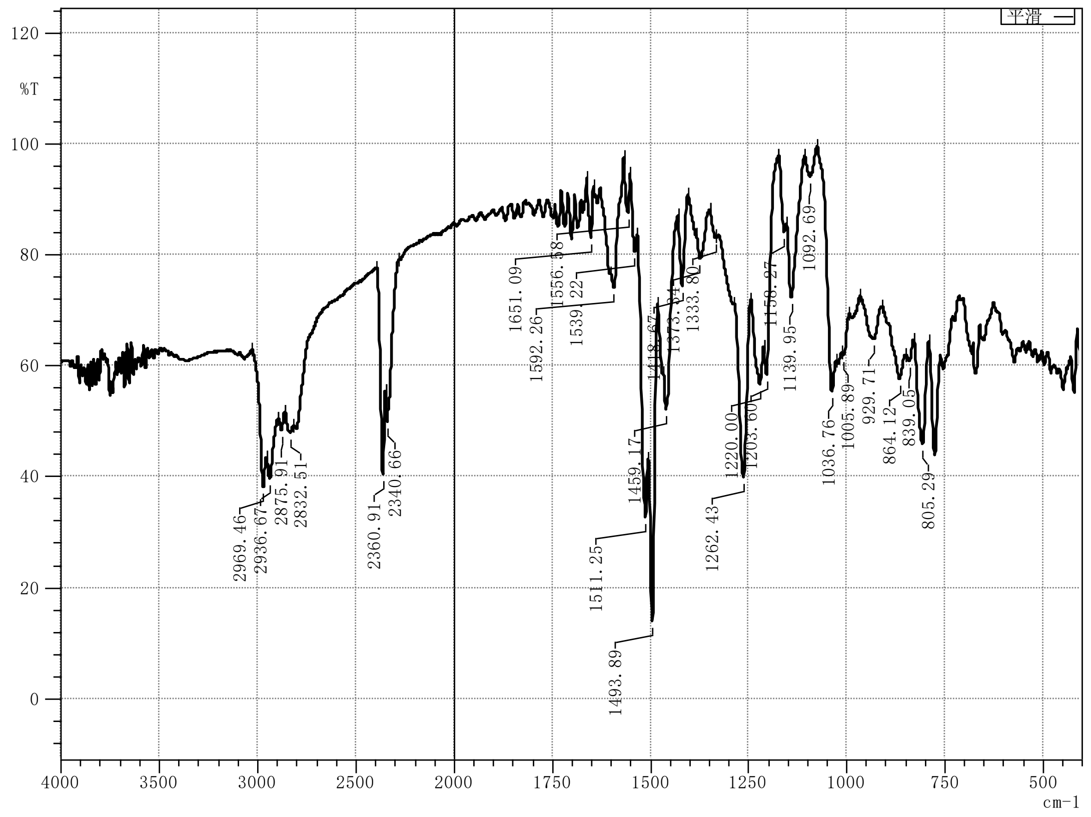

NMR (^{1}H and ^{13}C) and IR (KBr) of Compound CHJ04093:

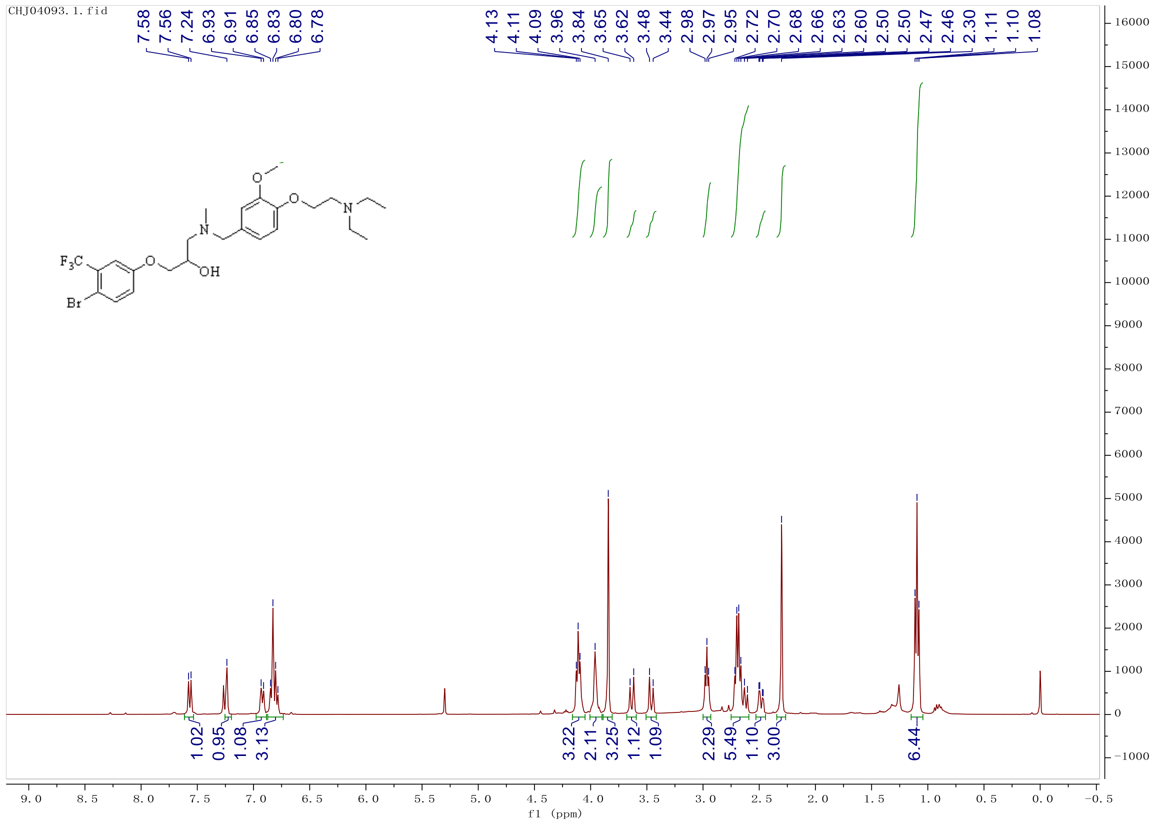

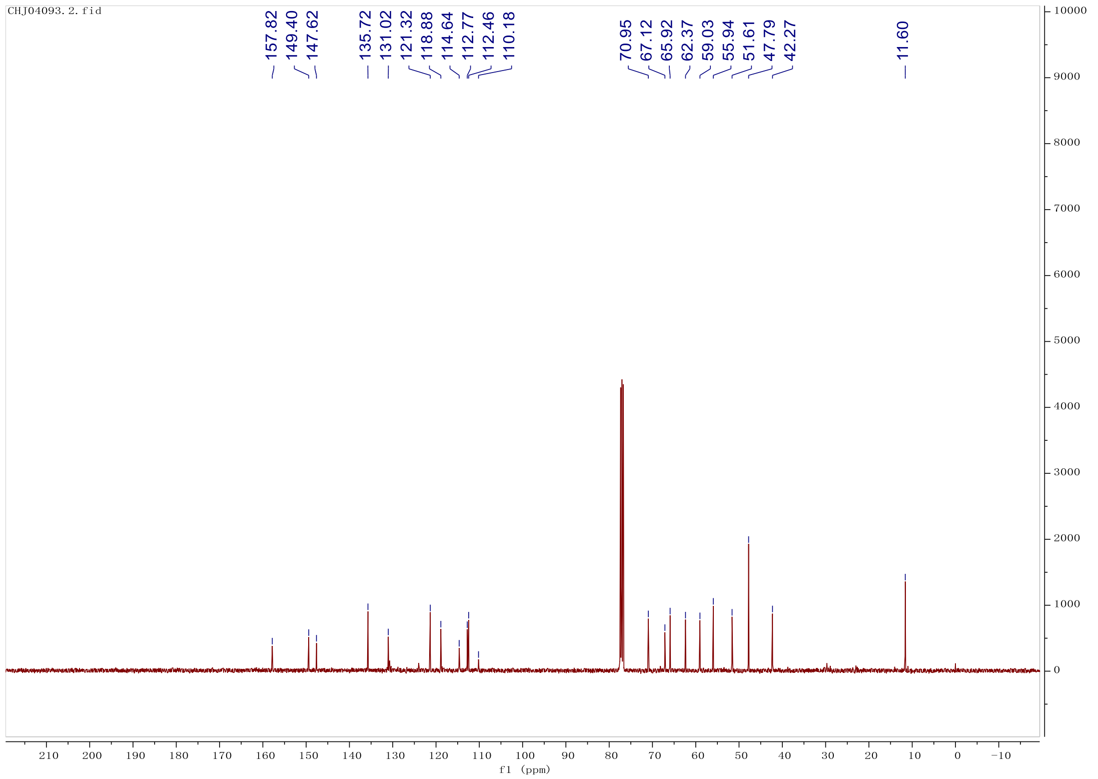
CHJ04093.2.fid
157.82
149.40
147.62
135.72
131.02
121.32
118.88
114.64
112.77
112.46
110.18
70.95
67.12
65.92
62.37
59.03
55.94
51.61
47.79
42.27
11.60
f1 (ppm)

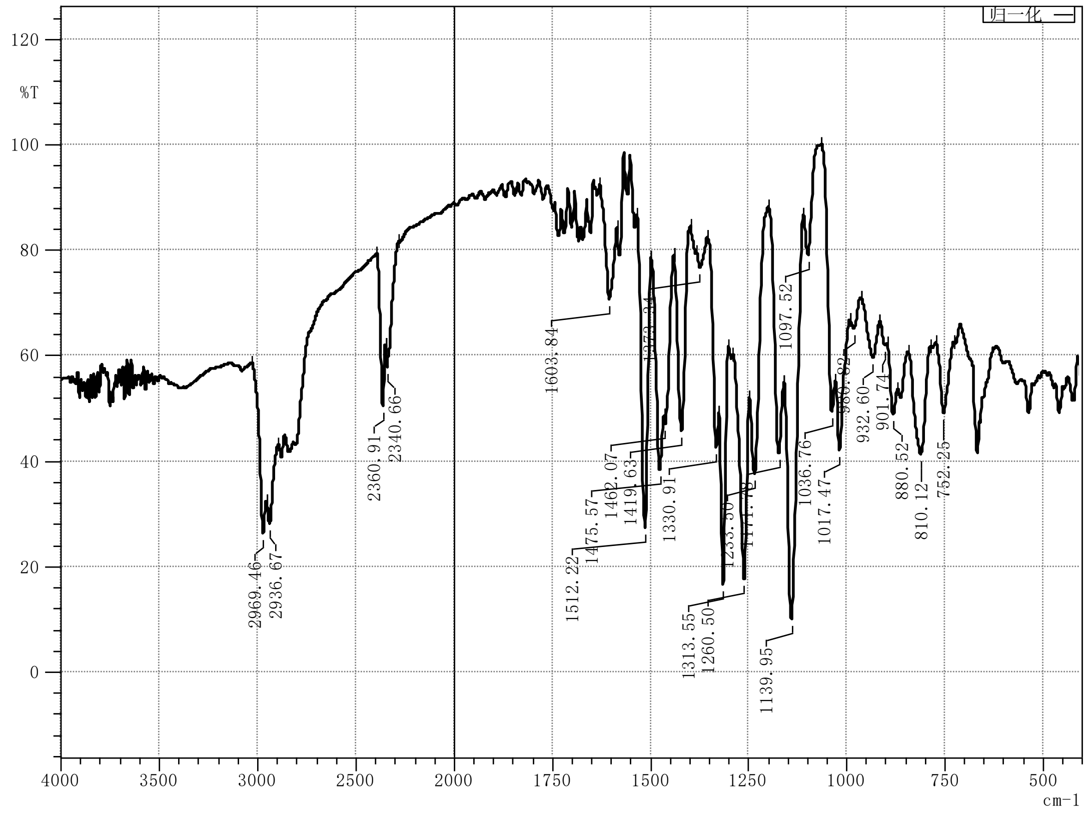
归一化
%T
cm-1
2969.46
2936.67
2360.91
2340.66
1603.84
1512.22
1475.57
1419.63
1330.91
1313.55
1260.50
1139.95
1097.52
1036.76
1017.47
932.60
901.74
880.52
810.12
752.25

NMR (^{1}H and ^{13}C) and IR (KBr) of Compound CHJ04094:

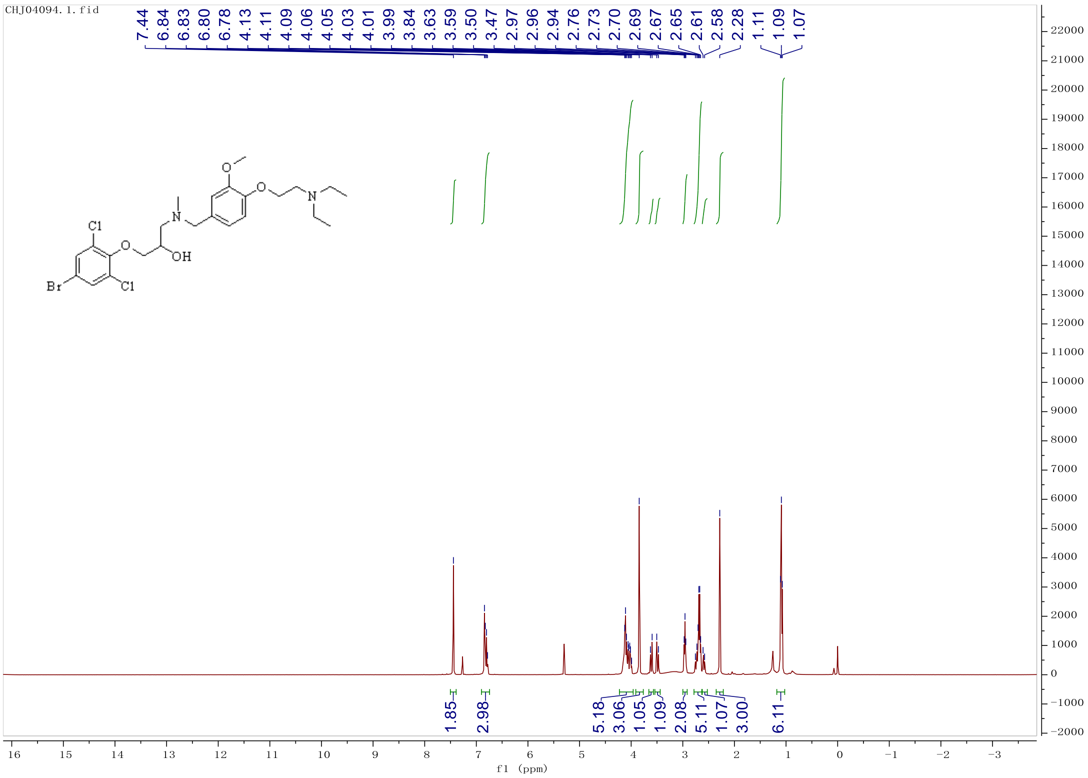

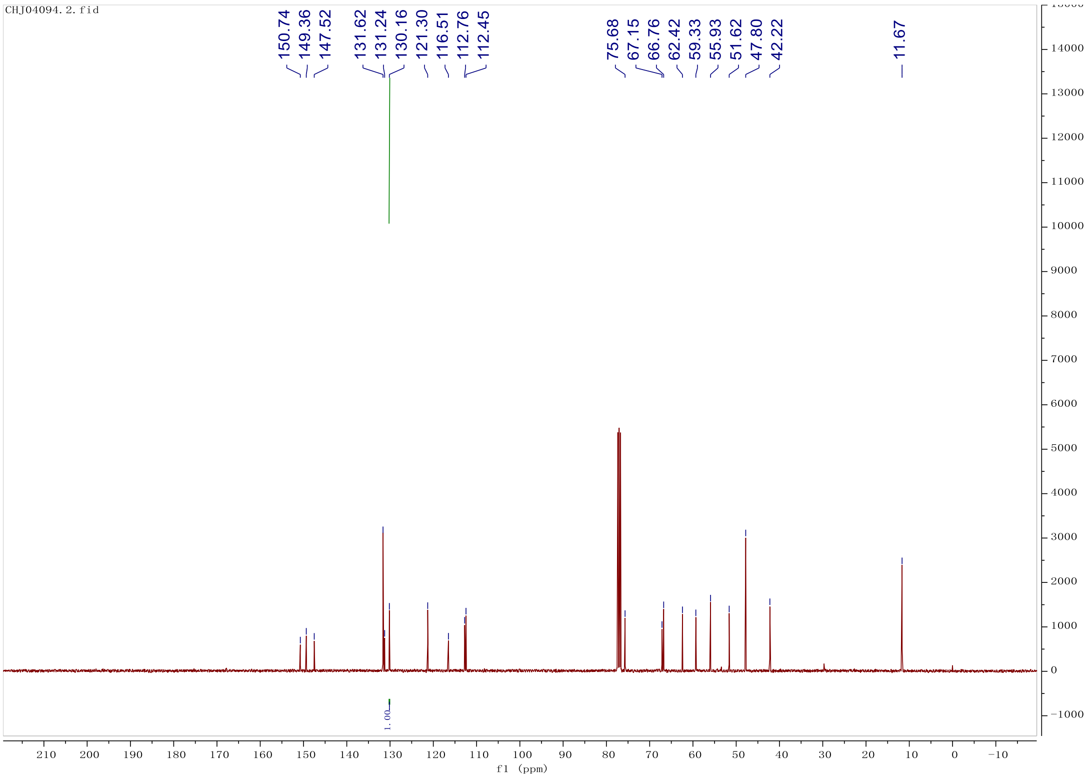

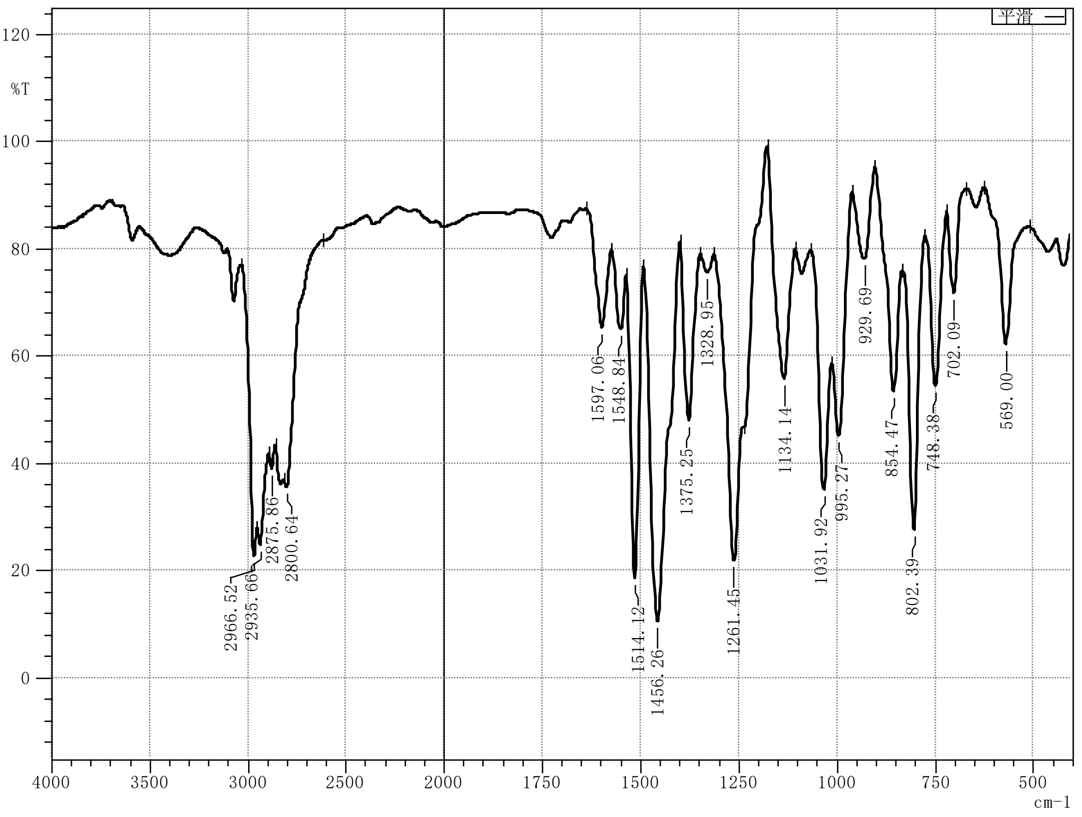

NMR (^{1}H and ^{13}C) and IR (KBr) of Compound CHJ04097:

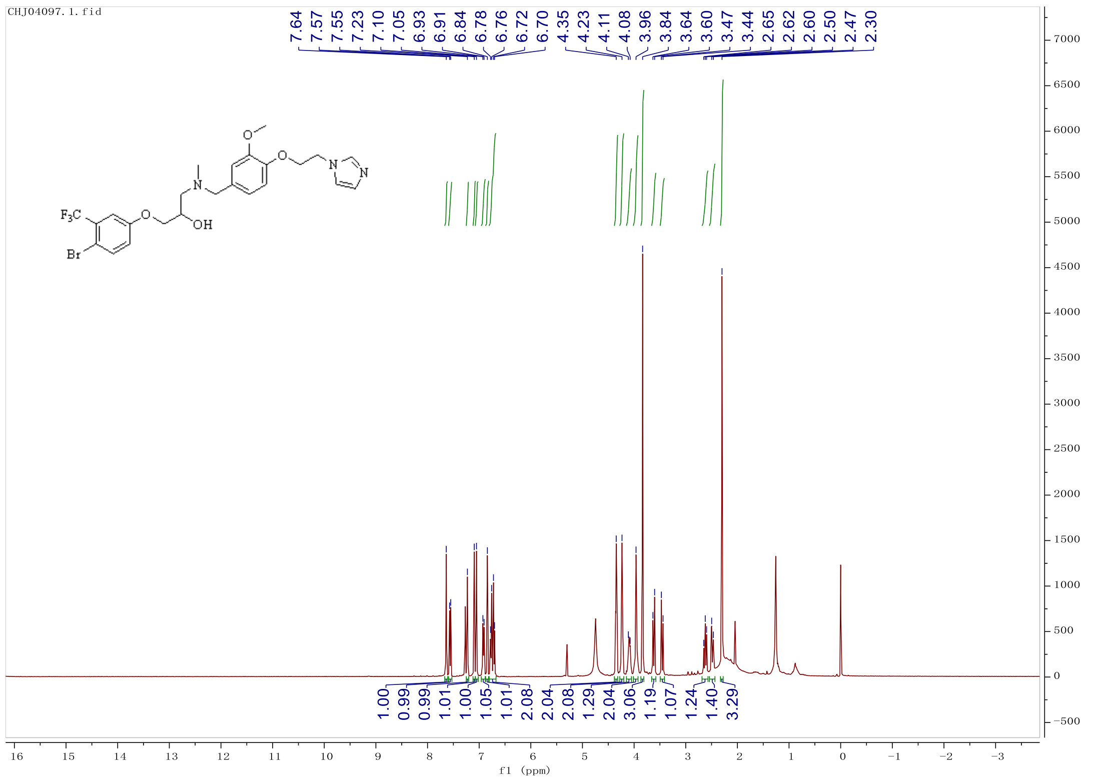

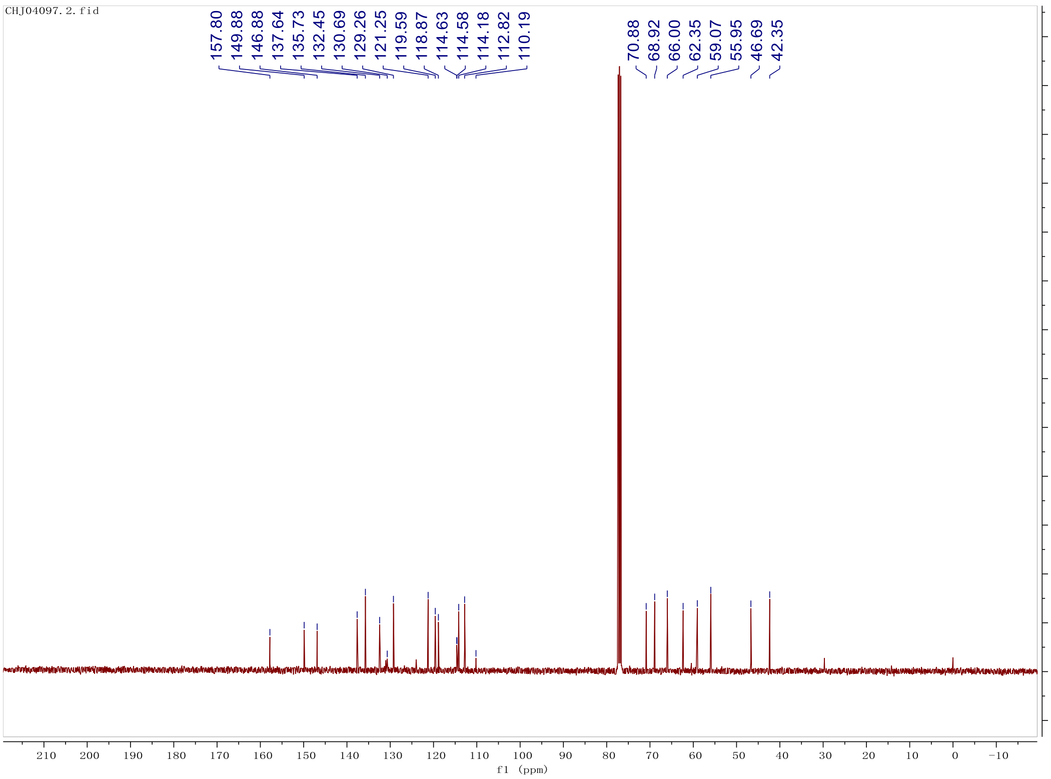
CHJ04097.2.fid
157.80
149.88
146.88
137.64
135.73
132.45
130.69
129.26
121.25
119.59
118.87
114.63
114.58
114.18
112.82
110.19
70.88
68.92
66.00
62.35
59.07
55.95
46.69
42.35
f1 (ppm)

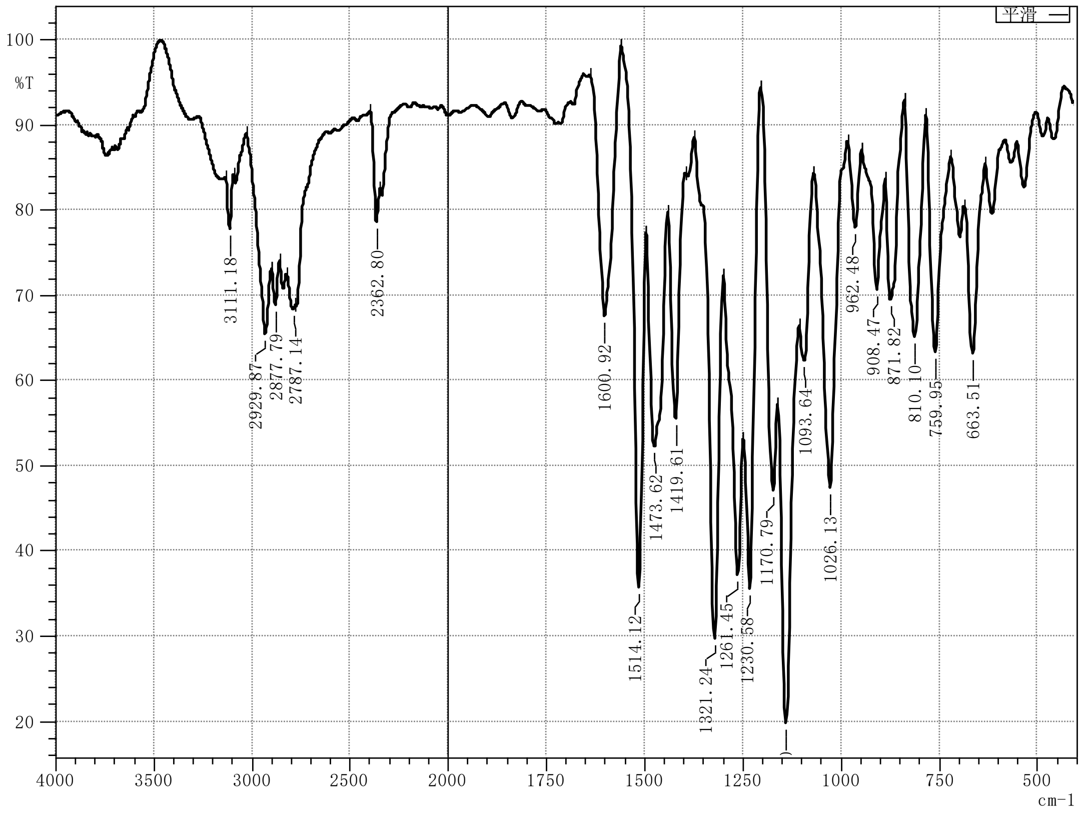
%T
3111.18
2929.87
2877.79
2787.14
2362.80
1600.92
1514.12
1473.62
1419.61
1321.24
1261.45
1230.58
1170.79
1093.64
1026.13
962.48
908.47
871.82
810.10
759.95
663.51
cm-1

NMR (^{1}H and ^{13}C) and IR (KBr) of Compound CHJ04099:

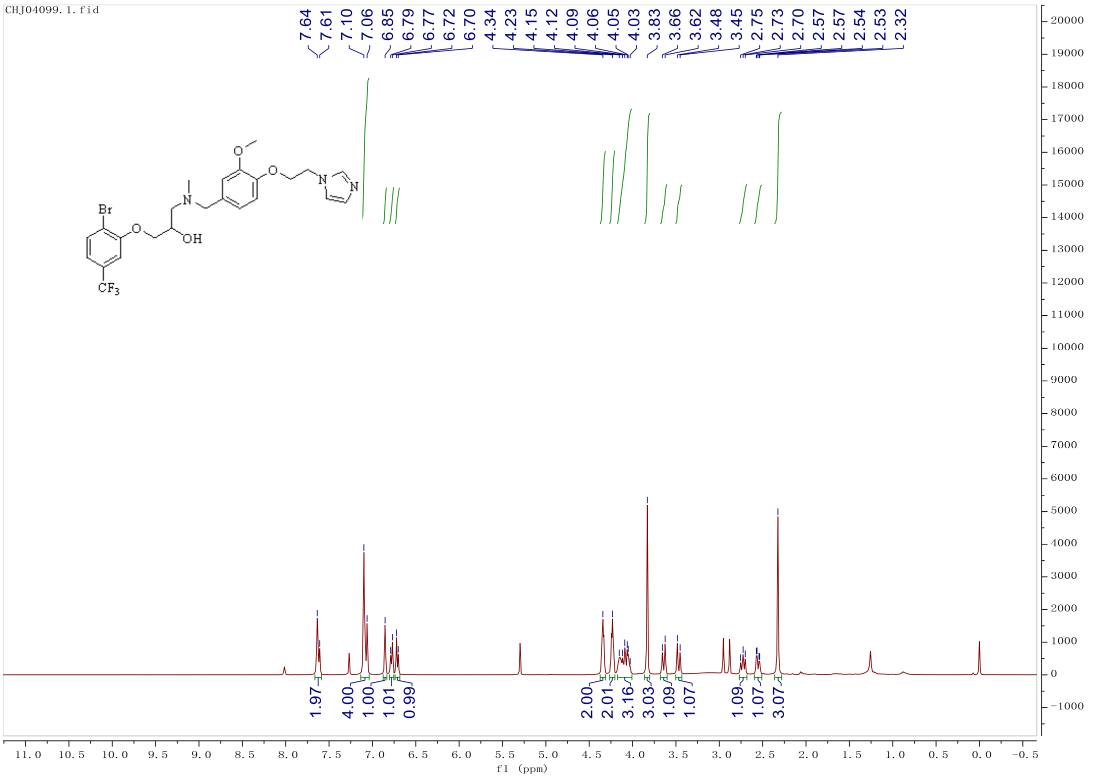

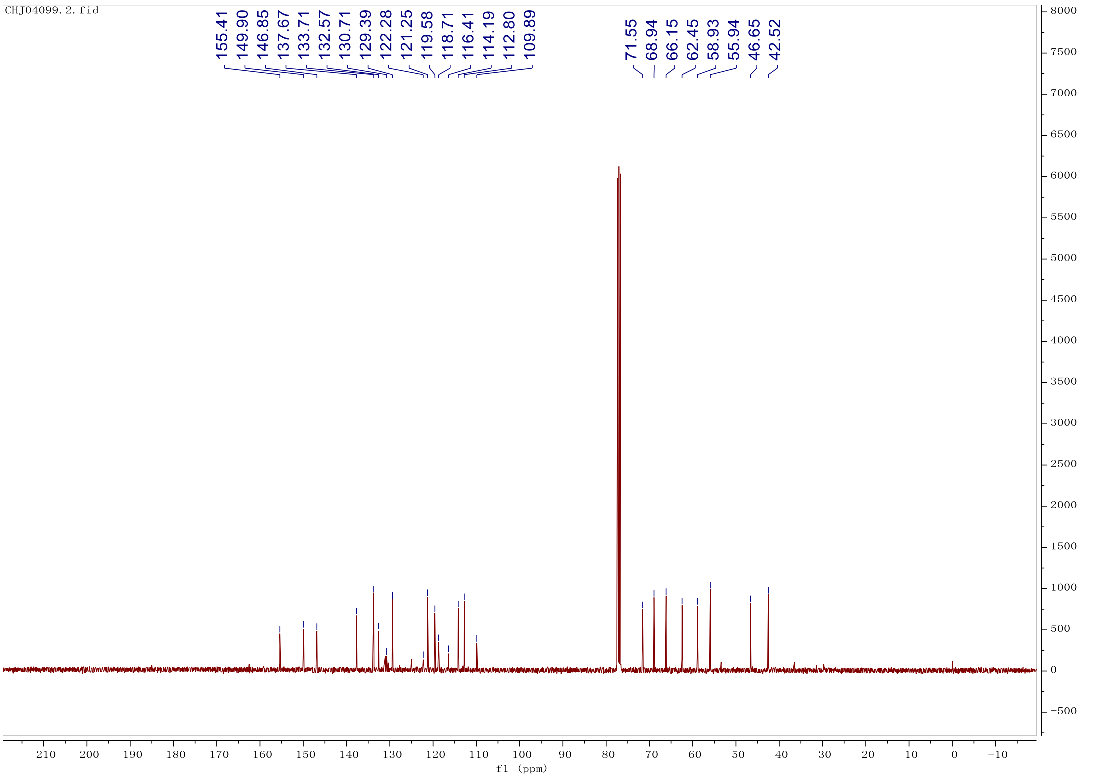

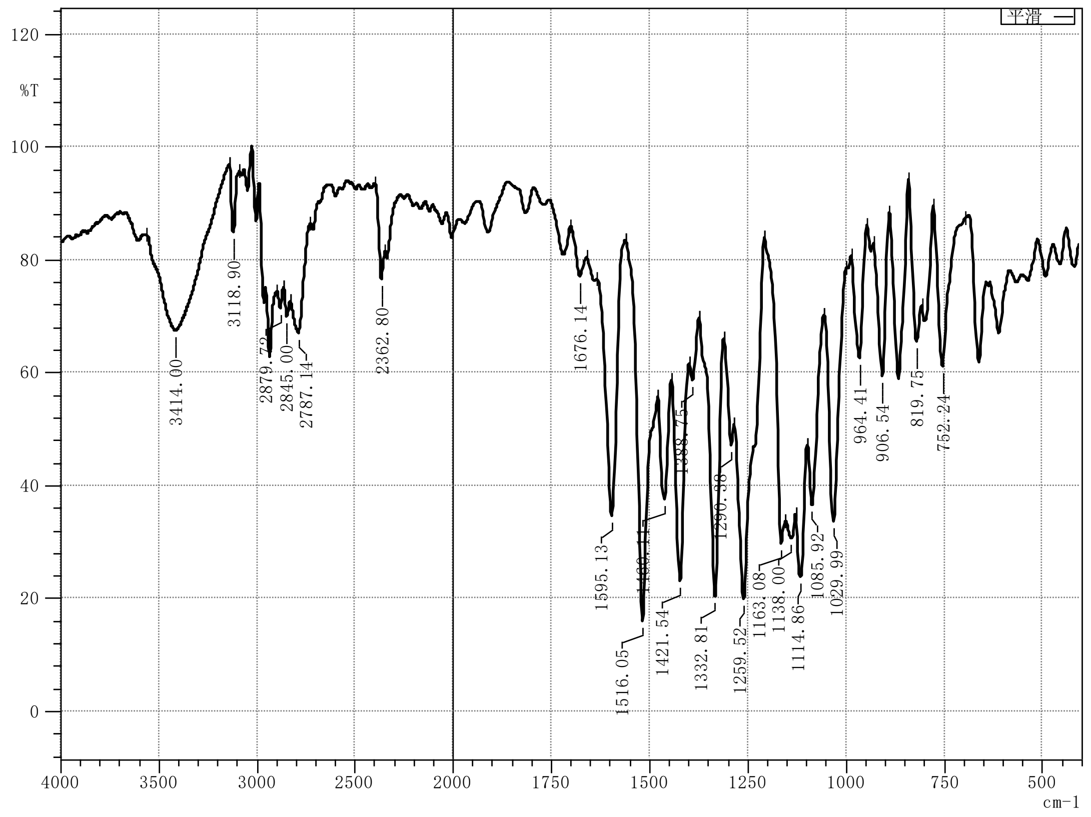

NMR (^{1}H and ^{13}C) and IR (KBr) of Compound CHJ05001:

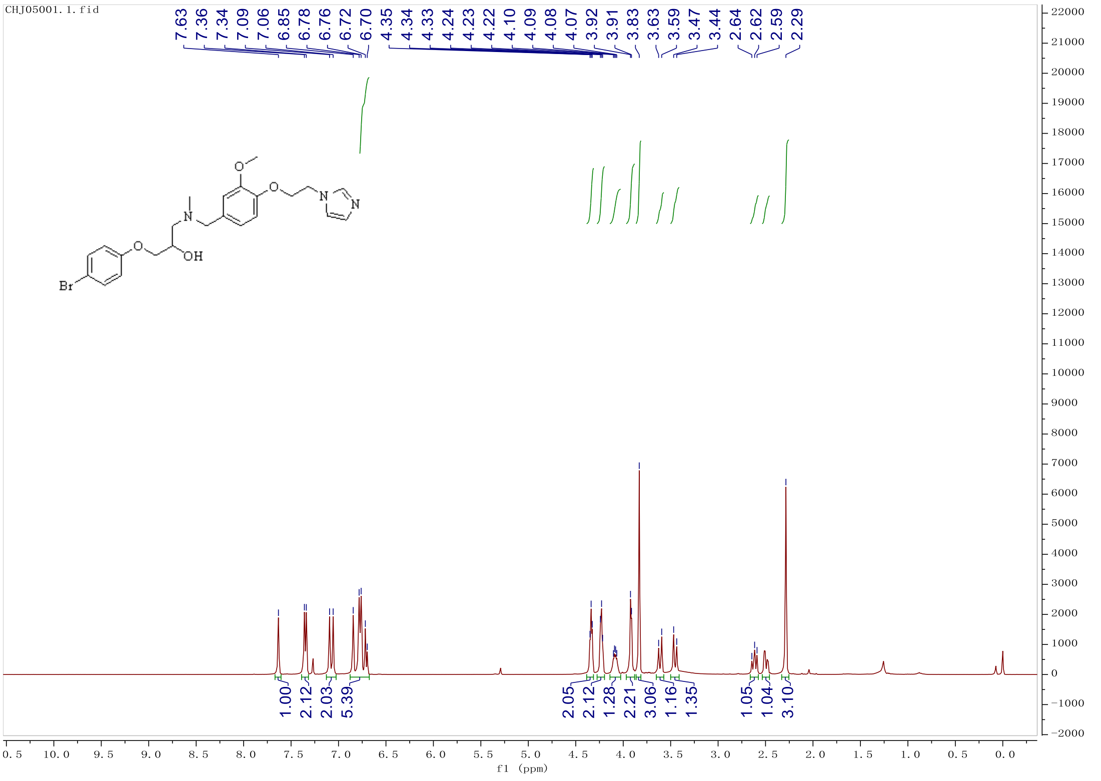

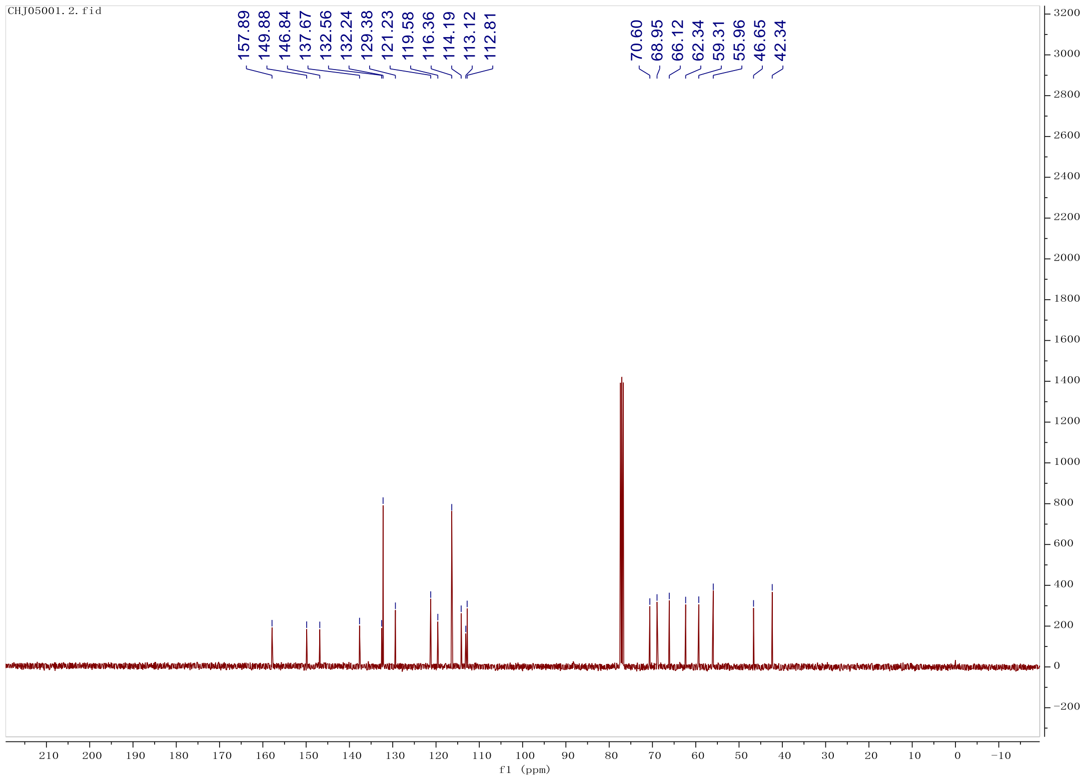
CHJ05001.2.fid
157.89
149.88
146.84
137.67
132.56
132.24
129.38
121.23
119.58
116.36
114.19
113.12
112.81
70.60
68.95
66.12
62.34
59.31
55.96
46.65
42.34
f1 (ppm)

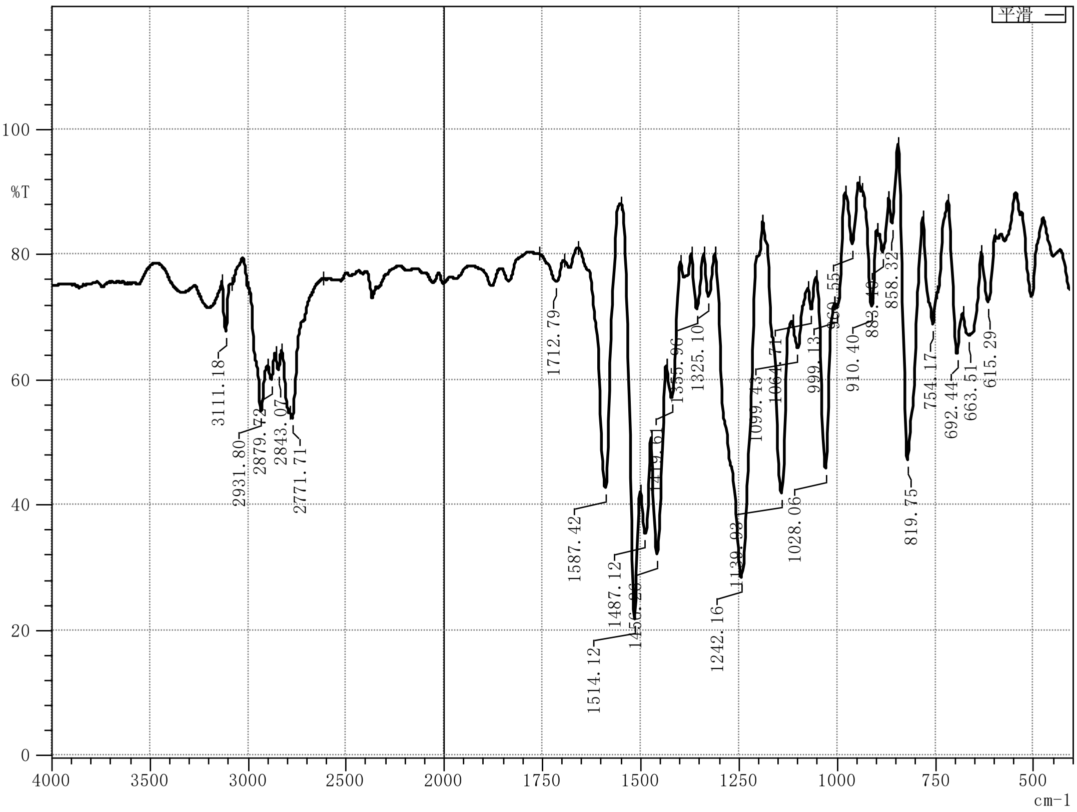
%T
3111.18
2931.80
2843.07
2771.71
1712.79
1587.42
1487.12
1514.12
1325.10
1242.16
1028.06
910.40
858.32
819.75
754.17
692.44
663.51
615.29
cm-1

NMR (^{1}H and ^{13}C) and IR (KBr) of Compound CHJ05002:

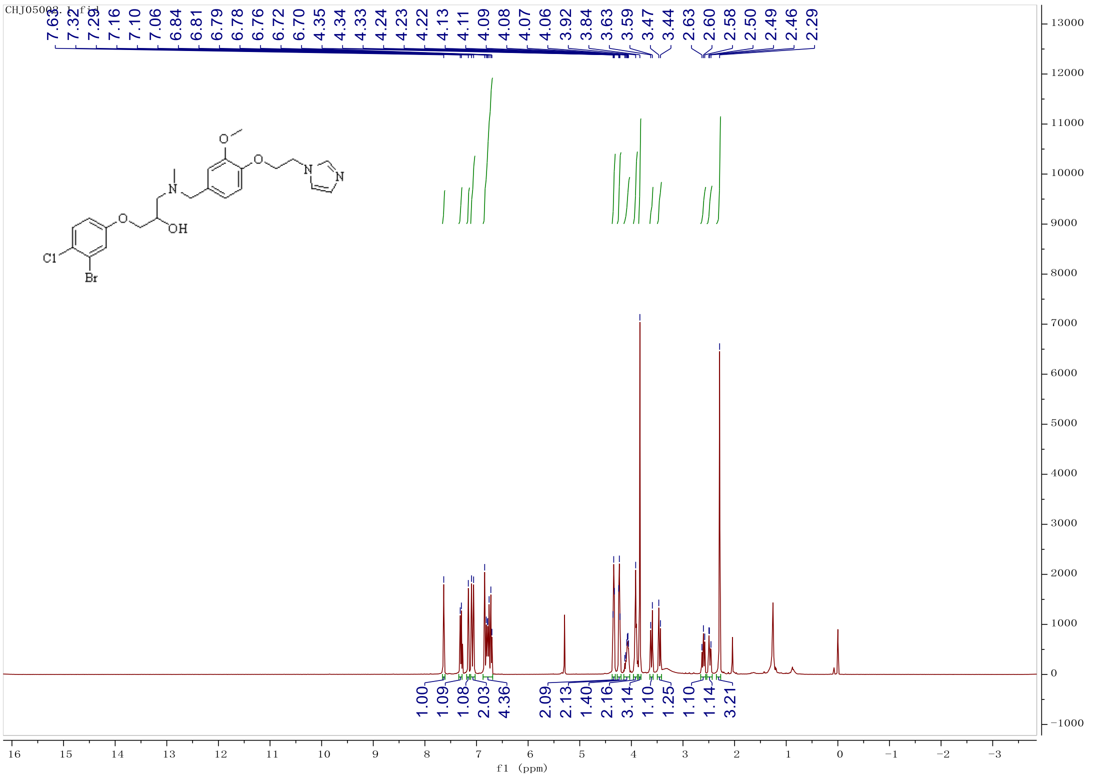

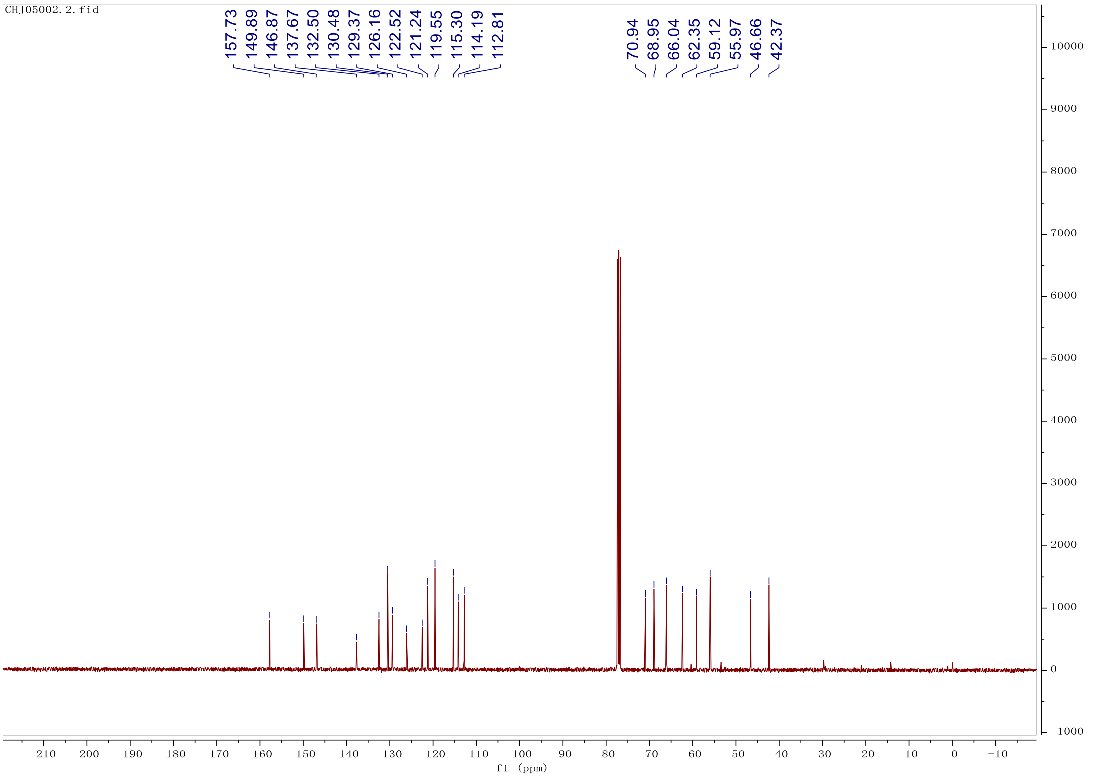

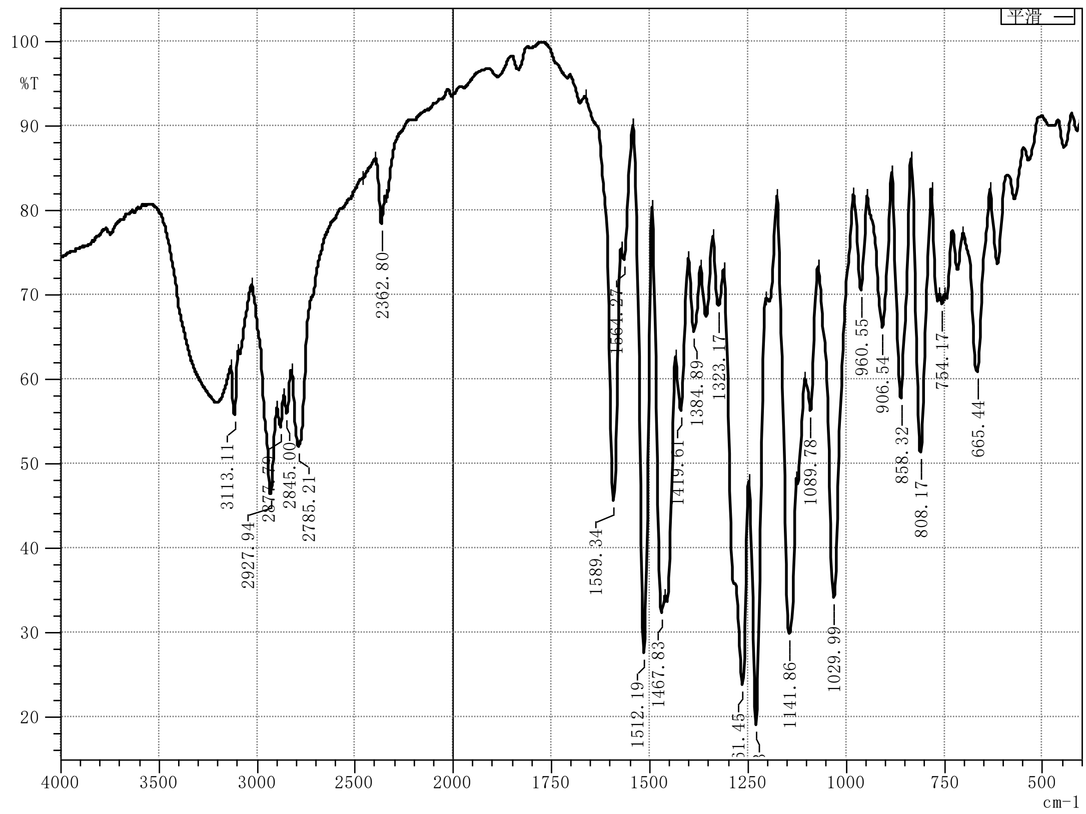

NMR (^{1}H and ^{13}C) and IR (KBr) of Compound CHJ05003:

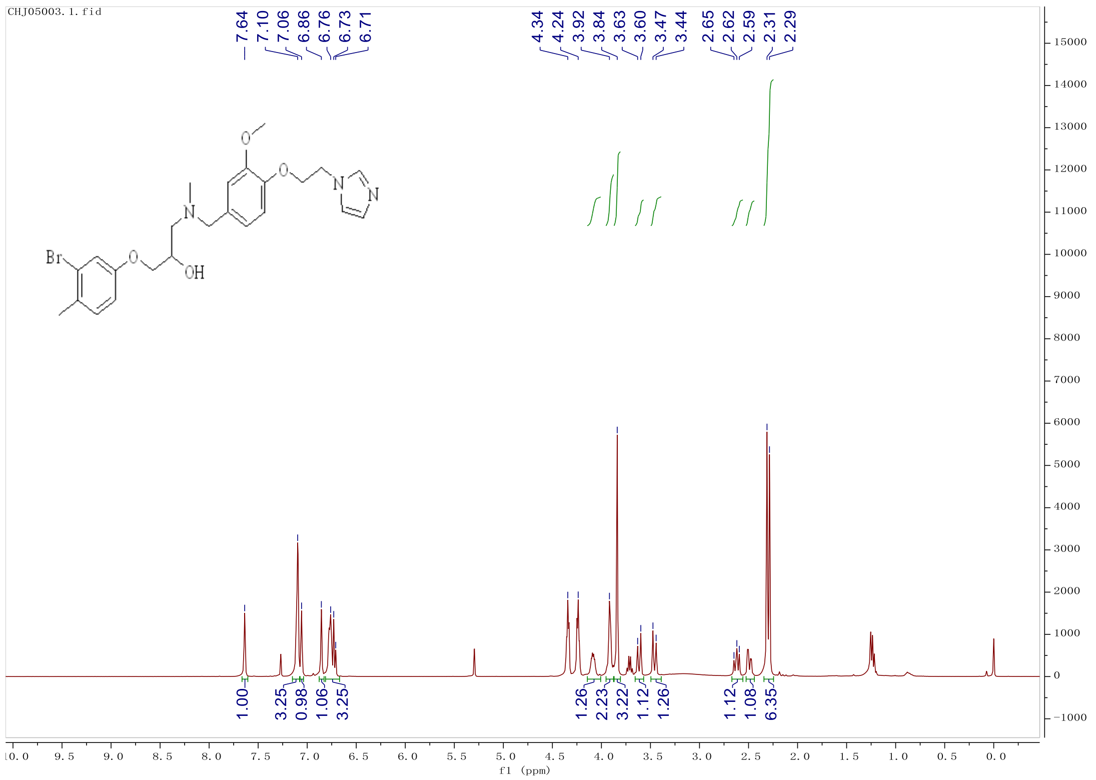

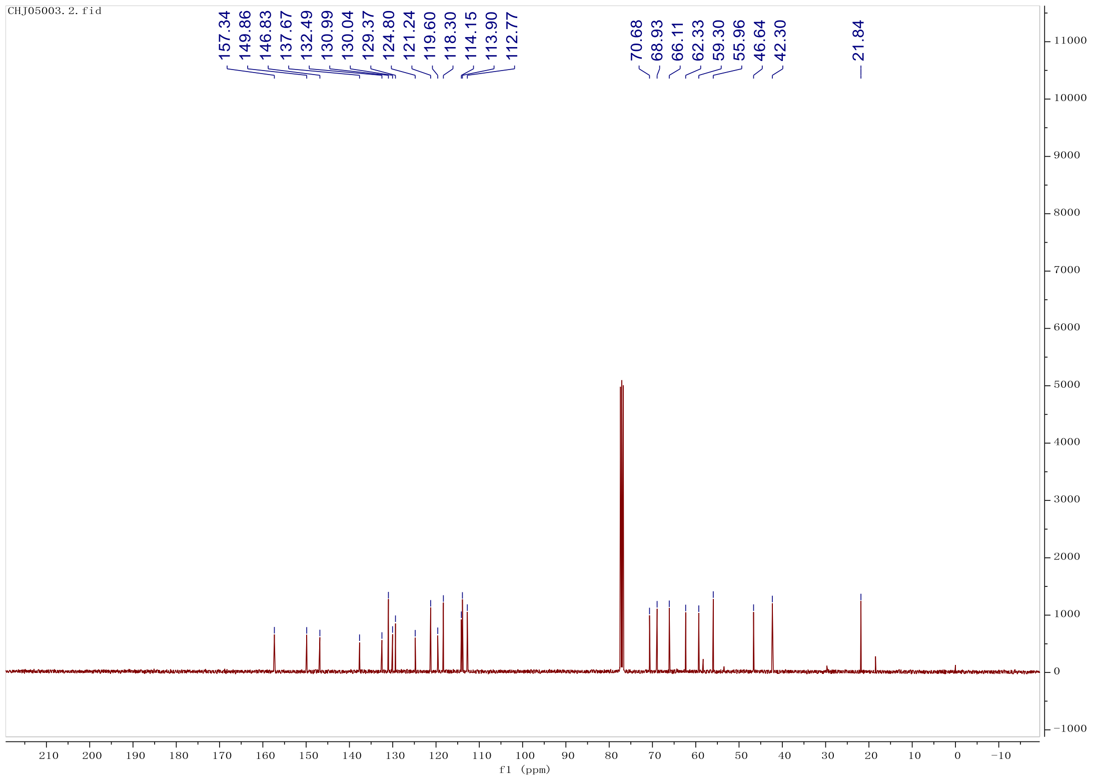
CHJ05003.2.fid
157.34
149.86
146.83
137.67
132.49
130.99
130.04
129.37
124.80
121.24
119.60
118.30
114.15
113.90
112.77
70.68
68.93
66.11
62.33
59.30
55.96
46.64
42.30
21.84
f1 (ppm)

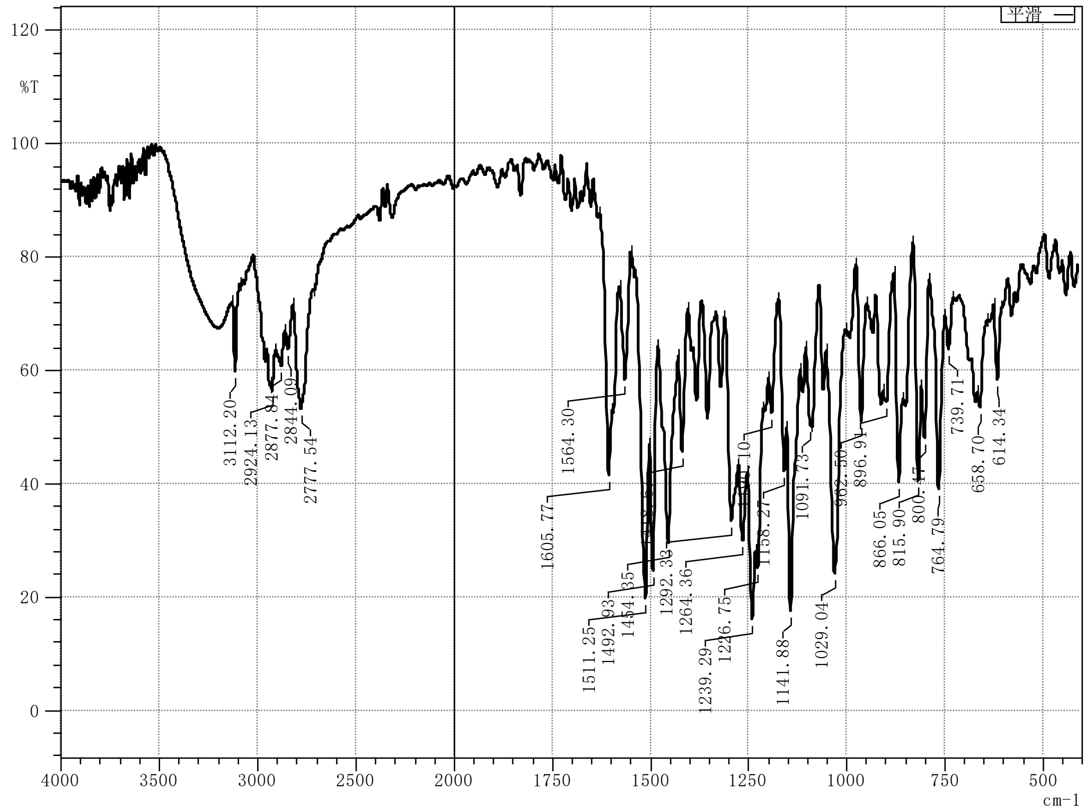
%T
cm-1
3112.20
2924.13
2877.84
2844.09
2777.54
1564.30
1605.77
1511.25
1492.93
1454.35
1292.33
1264.36
1239.29
1226.75
1158.27
1141.88
1091.73
1029.04
962.50
896.91
866.05
815.90
764.79
739.71
658.70
614.34
